# Molecular glues that inhibit deubiquitylase activity and inflammatory signaling

**DOI:** 10.1038/s41594-025-01517-5

**Published:** 2025-03-17

**Authors:** Francesca Chandler, Poli Adi Narayana Reddy, Smita Bhutda, Rebecca L. Ross, Arindam Datta, Miriam Walden, Kieran Walker, Stefano Di Donato, Joel A. Cassel, Michael A. Prakesch, Ahmed Aman, Alessandro Datti, Lisa J. Campbell, Martina Foglizzo, Lillie Bell, Daniel N. Stein, James R. Ault, Rima S. Al-awar, Antonio N. Calabrese, Frank Sicheri, Francesco Del Galdo, Joseph M. Salvino, Roger A. Greenberg, Elton Zeqiraj

**Affiliations:** 1 Astbury Centre for Structural Molecular Biology, School of Molecular and Cellular Biology, Faculty of Biological Sciences, University of Leeds, Leeds, UK; 2 Medicinal Chemistry, Molecular and Cellular Oncogenesis (MCO) Program and The Wistar Cancer Center Molecular Screening, The Wistar Institute, Philadelphia, PA USA; 3 Department of Cancer Biology, Penn Center for Genome Integrity, Basser Center for BRCA, Perelman School of Medicine, University of Pennsylvania, Philadelphia, PA USA; 4 Leeds Institute of Rheumatic and Musculoskeletal Medicine, Faculty of Medicine and Health, University of Leeds, Leeds, UK; 5 NIHR Leeds Biomedical Research Centre, Leeds Teaching Hospitals, NHS Trust, Chapel Allerton Hospital, Leeds, UK; 6 Drug Discovery Program, Ontario Institute for Cancer Research, Toronto, Ontario Canada; 7 Leslie Dan Faculty of Pharmacy, University of Toronto, Toronto, Ontario Canada; 8 Department of Agricultural, Food, and Environmental Sciences, University of Perugia, Perugia, Italy; 9 Department of Pharmacology and Toxicology, University of Toronto, Toronto, Ontario Canada; 10 Centre for Systems Biology, Lunenfeld-Tanenbaum Research Institute, Sinai Health System, Toronto, Ontario Canada; 11 Department of Molecular Genetics, University of Toronto, Toronto, Ontario Canada; 12 Department of Biochemistry, University of Toronto, Toronto, Ontario Canada

**Keywords:** Drug discovery, Cryoelectron microscopy, Proteases, Immunology, Multienzyme complexes

## Abstract

Deubiquitylases (DUBs) are crucial in cell signaling and are often regulated by interactions within protein complexes. The BRCC36 isopeptidase complex (BRISC) regulates inflammatory signaling by cleaving K63-linked polyubiquitin chains on type I interferon receptors (IFNAR1). As a Zn^2+^-dependent JAMM/MPN (JAB1, MOV34, MPR1, Pad1 N-terminal) DUB, BRCC36 is challenging to target with selective inhibitors. Here, we discover first-in-class inhibitors, termed BRISC molecular glues (BLUEs), which stabilize a 16-subunit human BRISC dimer in an autoinhibited conformation, blocking active sites and interactions with the targeting subunit, serine hydroxymethyltransferase 2. This unique mode of action results in selective inhibition of BRISC over related complexes with the same catalytic subunit, splice variants and other JAMM/MPN DUBs. BLUE treatment reduced interferon-stimulated gene expression in cells containing wild-type BRISC and this effect was abolished when using structure-guided, inhibitor-resistant BRISC mutants. Additionally, BLUEs increase IFNAR1 ubiquitylation and decrease IFNAR1 surface levels, offering a potential strategy to mitigate type I interferon-mediated diseases. Our approach also provides a template for designing selective inhibitors of large protein complexes by promoting rather than blocking protein–protein interactions.

## Abstract (web summary)

The BRCC36 isopeptidase complex (BRISC) is a deubiquitylase that stabilizes interferon receptors, driving inflammation. We discovered ‘BRISC molecular glue’ inhibitors (BLUEs) that selectively inactivate BRISC, promoting interferon receptor ubiquitylation and degradation to dampen immune responses.

## Main

Over 100 human deubiquitylases (DUBs) regulate cellular signaling by controlling protein activity, localization or stability^1–5^. Their dysfunction is implicated in autoimmune disorders, cancers, metabolic diseases and neurodegeneration^6–8^. Consequently, DUBs remain prominent therapeutic targets in drug discovery^9,10^.

BRCC36 is a JAMM/MPN (JAB1, MOV34, MPR1, Pad1 N-terminal) metallo-DUB that selectively cleaves K63-linked ubiquitin chains^11,12^. It exists in two complexes: the cytoplasmic BRCC36 isopeptidase complex (BRISC) and the nuclear Abraxas 1-regulated isopeptidase complex (ARISC). BRISC regulates type I interferon (IFN) signaling by stabilizing type I IFN receptors (IFNAR1), while ARISC interacts with breast cancer type 1 susceptibility protein (BRCA1) to facilitate DNA damage repair^13–15^. BRCC36 (MPN^+^) requires a pseudo-DUB partner (MPN^−^) for activity: Abraxas 1 in the nucleus or Abraxas 2 in the cytoplasm^12,16–18^. Both BRISC and ARISC also contain BRCC45 and MERIT40, forming 2:2:2:2 heterotetramers^16–19^. BRISC partners with serine hydroxymethyltransferase 2 (SHMT2) for IFNAR1 targeting and ARISC recruits BRCA1, BRCA1-associated RING domain 1 and receptor-associated protein 80 for double-strand break repair^13–15^. Cryo-electron microscopy (cryo-EM) structures of BRISC–SHMT2 revealed a U-shaped assembly bridging two BRCC45–MERIT40 ‘arms’, similar to ARISC structures^19–21,44^.

BRISC-mediated deubiquitylation of IFNAR1 receptors enhances Janus kinase (JAK)–signal transducer and activator of transcription (STAT) signaling and IFN-stimulated gene (ISG) expression ^22^. Elevated ISGs are linked to autoimmune diseases such as systemic lupus erythematosus^23^, rheumatoid arthritis^24^ and systemic sclerosis (SSc)^25^. BRISC-deficient mice are protected from excessive IFN signaling and inflammation^22^, highlighting BRISC inhibition as a strategy to alleviate chronic inflammation and autoimmune disease driven pathology.

Notable strides have been made in selectively targeting DUBs from the ubiquitin-specific protease (USP) family^26–30^, offering therapeutic potential and tools to probe DUB biology. However, most JAMM/MPN DUB inhibitors are broad-spectrum zinc chelators and no selective inhibitors exist for BRCC36 complexes^11,31^. Capzimin, a quinoline-8-thiol derivative, targets the proteasomal Rpn11 active-site zinc but also inhibits BRCC36 and AMSH^32^. Inhibitors of COP9 signalosome subunit 5 (CSN5) deneddylase likewise bind the catalytic zinc but show specificity for CSN5 over AMSH and PSMD14 (ref. ^33^). Despite advances in inhibitor development for many DUBs^34^, current JAMM/MPN inhibitors target the conserved zinc-binding pocket, posing challenges for selective inhibitor design.

Molecular glues (MGs) are small molecules that stabilize protein–protein interactions^35,36^, exemplified by immunosuppressants (for example, cyclosporin A^37,38^ and rapamycin) and natural degraders (for example, auxin in plants^36^). Immunomodulatory drugs such as thalidomide function as MGs by stabilizing an interaction between the E3 ligase cereblon and neosubstrates, promoting their degradation^39^. MGs, thus, offer a means to regulate protein stability but none have been reported for DUBs.

We describe first-in-class selective BRISC inhibitors and define a new mechanism for DUB inhibition. Cryo-EM structures reveal how these small molecules act as MGs without binding the active-site zinc. Instead, they stabilize a BRISC conformer that blocks BRCC36 from cleaving ubiquitin chains. Structure-guided mutagenesis and cell-based studies confirm target engagement and we validate the inhibitor mechanism in IFN-stimulated human cells and participant samples. These findings highlight the therapeutic potential of MG compounds that induce specific protein–protein interactions for selective inhibition of macromolecular complexes.

## Results

### Identification of first-in-class selective BRISC inhibitors

**Fig. 1:**
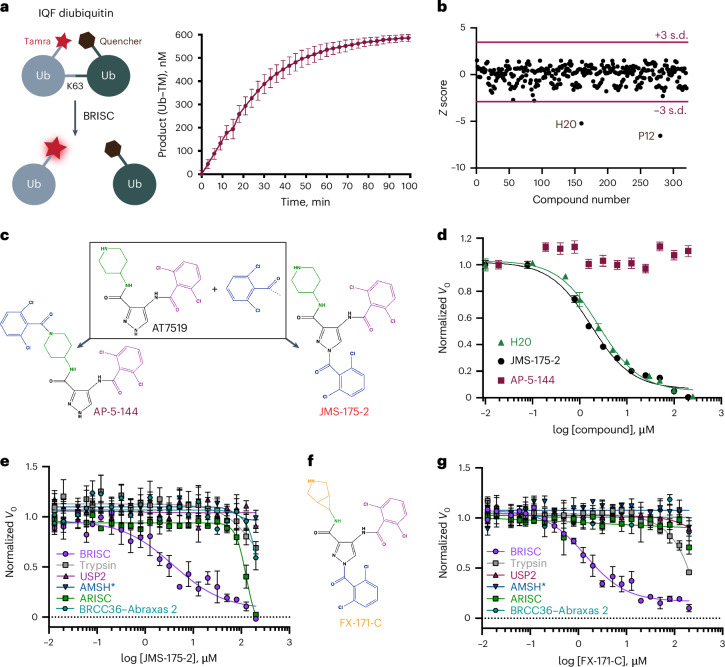
Fluorescence-based screen to identify first-in-class JAMM inhibitors. **a**, Left, schematic of a TAMRA-linked IQF diubiquitin substrate. Ub, ubiquitin. Right, reaction progress curve of BRISC DUB activity. Data points are the mean ± s.e.m. of two independent experiments carried out in technical duplicate. **b**, *Z*-score normalization of 320 compounds from an in-house kinase-directed inhibitor library and identification of hit compounds in wells H20 and P12. **c**, Chemical structures of AT7519 and two isomers with an additional 2,6-dichlorobenzoyl moiety. **d**, Dose–response inhibition of BRISC activity by the H20 compound and the two potential isomers, AP-5-144 and JMS-175-2. **e**, Dose–response inhibition of trypsin, USP2 and JAMM/MPN DUB enzymes AMSH*, BRISC, ARISC and BRCC36–Abraxas 2 by JMS-175-2. **f**, Chemical structure of the FX-171-C compound. **g**, As in **e**, but for FX-171-C. Data points in **d**,**e**,**g** are the mean ± s.e.m. of three independent experiments carried out in technical duplicate. Source data

**Extended Data Fig. 1:**
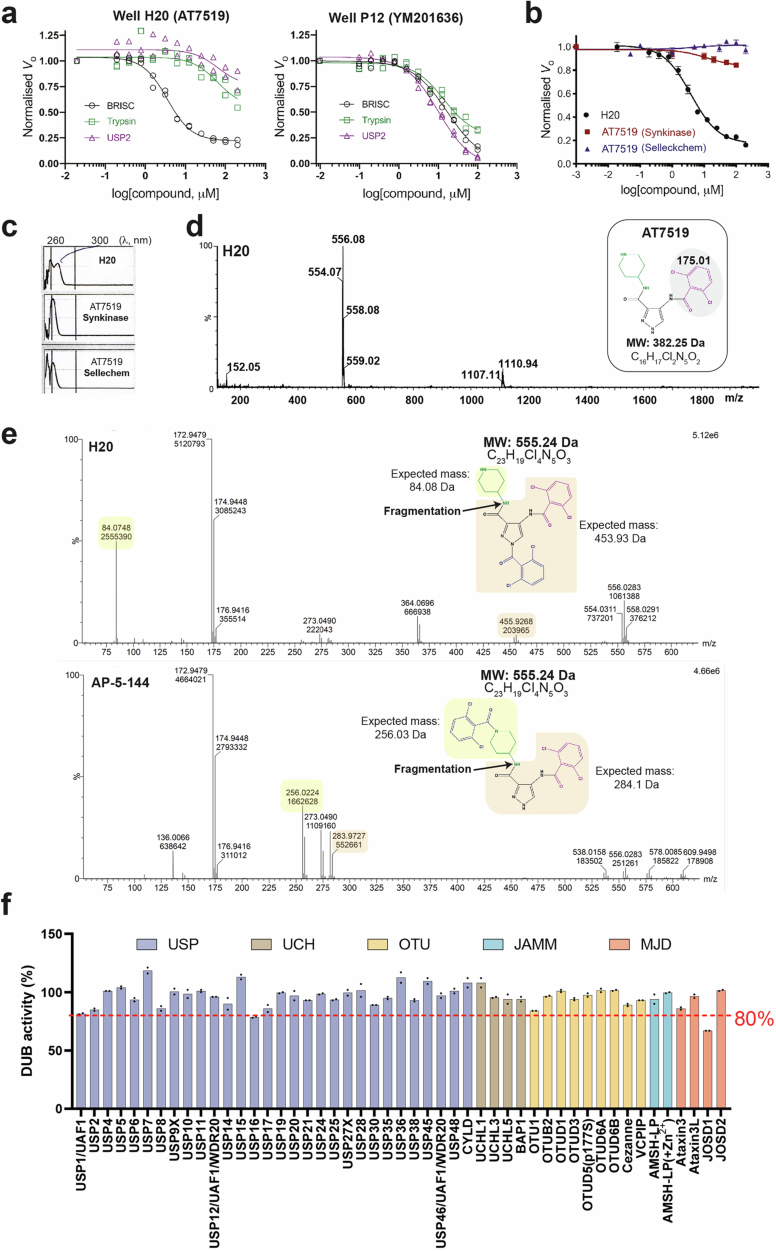
Validation of hit compounds. **a**, Dose response curves for hit compounds against BRISC (1 nM), USP2 (100 nM) and trypsin (125 nM) using the internally-quenched fluorescence di-ubiquitin assay described in Fig. 1a. Data points from two independent experiments are plotted. **b**, Re-testing of purchased H20 hit compound presumed to be AT7519. Data points are mean ± SEM from three independent experiments. **c**, UV-vis profile of compound in well H20 and purchased AT7519 compounds from Synkinase and Selleckchem. **d**, Liquid-chromatography mass spectrometry (LC-MS) spectra of H20 compound. *Inset*, AT7519 structure. The difference between the H20 compound and AT7519 is 173 Da, which corresponds to the mass of a dichlorobenzoyl group. **e**, MS fragmentation analyses for H20 compound and a synthesised isomer, AP-5-144. **f**, Profiling JMS-175-2 activity (5 µM) against a panel of 48 available DUBs using a ubiquitin-rhodamine(110)-glycine enzymatic assay. Data points are from two independent experiments. Source data

We designed a biochemical screen to identify BRISC small-molecule inhibitors by measuring activity of a commercial K63-linked diubiquitin substrate with an internally quenched fluorophore (IQF) (Fig. 1a, left). Increased fluorescence was detected over time, enabling continuous readout of DUB activity (Fig. 1a, right). We screened an in-house compound library of 320 published and custom-made kinase inhibitors and identified compounds AT7519 (well H20) and YM201636 (well P12) as hits (Fig. 1b). Compound selectivity was assessed against the broad-spectrum DUB USP2 and the serine protease trypsin, which cleave K63-ubiquitin substrate under the same assay conditions. YM201636 (well P12) inhibited BRISC, trypsin and USP2, suggesting that it is a nonspecific inhibitor, whereas what we presumed to be compound AT7519 (well H20) showed selective inhibition of BRISC DUB activity (Extended Data Fig. 1a). To further validate the compound in well H20, we purchased AT7519 from two commercial vendors, Synkinase and Selleckchem. Curiously, neither inhibited BRISC DUB activity in the IQF assay (Extended Data Fig. 1b). Ultraviolet–visible light (UV–vis) spectroscopy analyses showed a different spectrum for the compound in well H20 compared to the purchased AT7519 compounds (Extended Data Fig. 1c), suggesting that the compound in well H20 was different to AT7519. Liquid chromatography–mass spectrometry (LC–MS) revealed that the H20 compound was pure, with a mass of 555.55 Da instead of the expected mass of 382.25 Da (ref. ^40^) (Extended Data Fig. 1d). This mass difference is consistent with the addition of a 2,6-dichlorobenzoyl group, which we reasoned could have been inadvertently added during chemical synthesis at either the piperidine or the pyrazole ring. We synthesized two possible isomers: AP-5-144 and JMS-175-2 (Fig. 1c) and tested their inhibitory effects against BRISC. We found that JMS-175-2 matched the profile of the compound in well H20, inhibiting BRISC with a half-maximal inhibitory concentration (IC_50_) of 3.8 μM (Fig. 1d). Consistent with the JMS-175-2 structure, MS fragmentation analyses showed that compound H20 contains the 2,6-dichlorobenzoyl modification at the pyrazole ring and not the piperidine ring (Extended Data Fig. 1e). The AP-5-144 isomer did not inhibit BRISC and, using fragmentation analyses, we confirmed that AP-5-144 did not match the chemical structure of compound H20 (Extended Data Fig. 1e). These data confirmed the chemical structure of the compound in well H20 and led to the serendipitous identification of the BRISC inhibitor JMS-175-2.

We next determined inhibitor selectivity for BRISC beyond USP2 and trypsin. AMSH is a related JAMM/MPN DUB that, like BRCC36, selectively cleaves K63-linked polyubiquitin chains^41,42^. JMS-175-2 did not inhibit AMSH* (a STAM2–AMSH fusion)^43^ (Fig. 1e), showing that it is selective for BRISC over other zinc-dependent DUBs. Remarkably, JMS-175-2 did not inhibit the nuclear ARISC complex, which shares three of the four BRISC subunits, including the catalytic subunit BRCC36 (Fig. 1e). A related analog, FX-171-C (Fig. 1f), had a moderately improved IC_50_ of 1.4 μM compared to JMS-175-2 (IC_50_ = 3.8 μM) and retained selectivity for BRISC against other JAMM/MPN DUBs (Fig. 1g). These data confirm that the JMS-175-2 series of compounds are selective BRISC inhibitors and suggest that the specificity is conferred, in part, by the Abraxas 2 subunit that is substituted for Abraxas 1 in the ARISC complex.

To fully explore the selectivity profile of JMS-175-2, we evaluated its inhibitory effects on 48 DUBs spanning five DUB families. JMS-175-2 did not fully inhibit any of the DUBs present in the panel, including AMSH-LP (Extended Data Fig. 1f).

**Extended Data Fig. 2:**
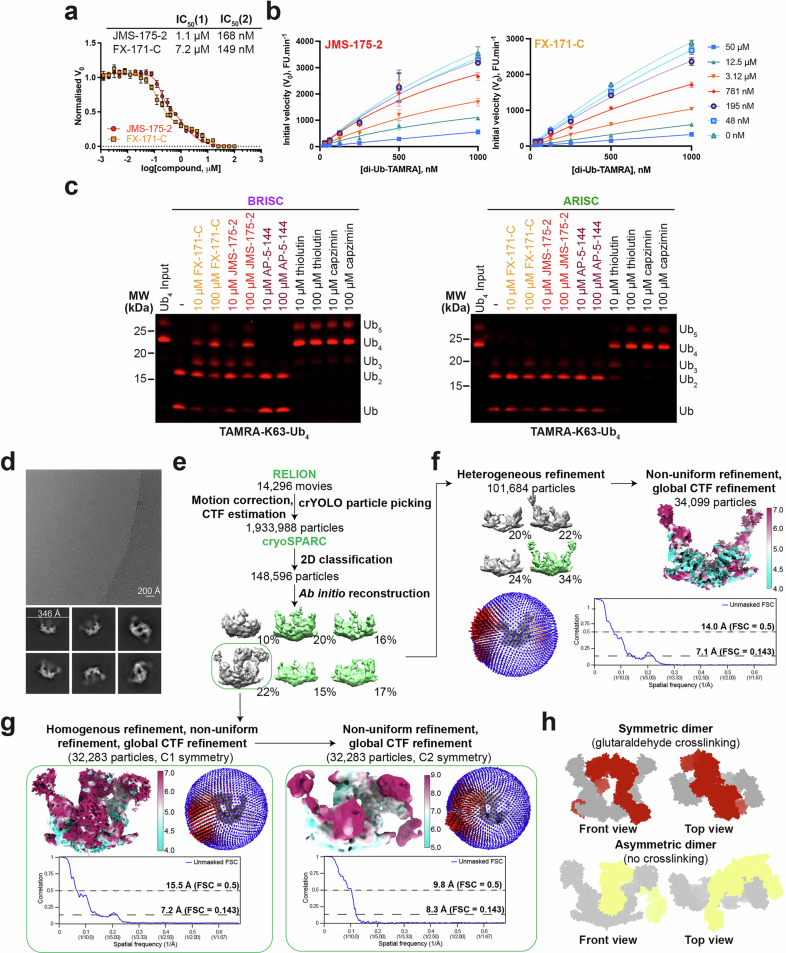
Identification of a higher-order BRISC conformation. **a**, 32-point dose-response inhibition assay with JMS-175-2 and FX-171-C, with a biphasic curve fitted. Data points are mean ± SEM of two independent experiments carried out in technical duplicate. **b**, Michaelis-Menten plots of BRISC activity against a K63-linked di-ubiquitin fluorogenic substrate with increasing concentrations of JMS-175-2 and FX-171-C. Data points are mean ± SEM of three independent experiments carried out in technical duplicate. **c**, In-gel DUB assay comparing cleavage of a TAMRA-labelled K63-linked tetraubiquitin substrate by BRISC (*left*) and ARISC (*right*) with indicated compounds. Uncropped gels are in Source Data Extended Fig. 2. The gels shown are representative of three independent experiments. **d**, Representative micrograph from 14,296 movies (BRISC dataset) and corresponding 2D class averages generated in cryoSPARC. **e**, **f**, Cryo-EM processing workflow for BRISC **f**, monomer and **g**, dimer. Green indicates selected classes for 3D refinement in cryoSPARC. **f**, Final monomer cryo-EM density map coloured by local resolution and Euler angular distribution (*left*). Rod heights are proportional to the number of particles in each direction. Unmasked FSC curves with resolution calculated using the gold standard FSC cut-off at 0.143 and 0.5 frequency. **g**, Final dimer maps with C1 and C2 symmetry applied, coloured by local resolution. Euler angular distribution shown with rods, and unmasked FSC curves, as in **f**. **h**, *Top*, surface model of ARISC dimers observed in negative stain EM from grids prepared using the GraFix cross-linking method. The same conformation is reported for BRISC dimers from nsEM grids prepared using GraFix. *Bottom*, an asymmetric BRISC dimer conformation observed in cryo-EM without cross-linking. Source data

Curiously, JMS-175-2 and FX-171-C did not inhibit the minimally active BRCC36–Abraxas 2 complex, which indicates that the arm regions containing BRCC45 and MERIT40 also contribute to the inhibitor selectivity profile (Fig. 1e,g). We noticed a biphasic mode of inhibition (Extended Data Fig. 2a) and enzyme activity inhibition plots at different substrate concentrations suggested that JMS-175-2 and FX-171-C were not competitive inhibitors (Extended Data Fig. 2b). The strong selectivity of the JMS-175-2 and FX-171-C compounds and the noncompetitive mode of inhibition indicate that these inhibitors do not target the Zn^2+^ active site, unlike previously described JAMM/MPN inhibitors^31–33^.

We used a commercially available fluorescently labeled K63-linked tetraubiquitin substrate to demonstrate that both JMS-175-2 and FX-171-C inhibit BRISC-mediated cleavage of polyubiquitin chains (Extended Data Fig. 2c). The other possible JMS-175-2 stereoisomer, AP-5-144, did not inhibit BRISC cleavage of tetraubiquitin chains, consistent with the diubiquitin fluorescence assay (Fig. 1d). Importantly, JMS-175-2 and FX-171-C did not inhibit ARISC activity against tetraubiquitin (Extended Data Fig. 2c).

These experiments identify the first selective BRISC inhibitors and suggest a unique mechanism of action whereby the Abraxas 2 pseudo-DUB subunit and the BRCC45–MERIT40 arms contribute to selective inhibition.

### BRISC inhibitors stabilize an autoinhibited dimer

**Fig. 2:**
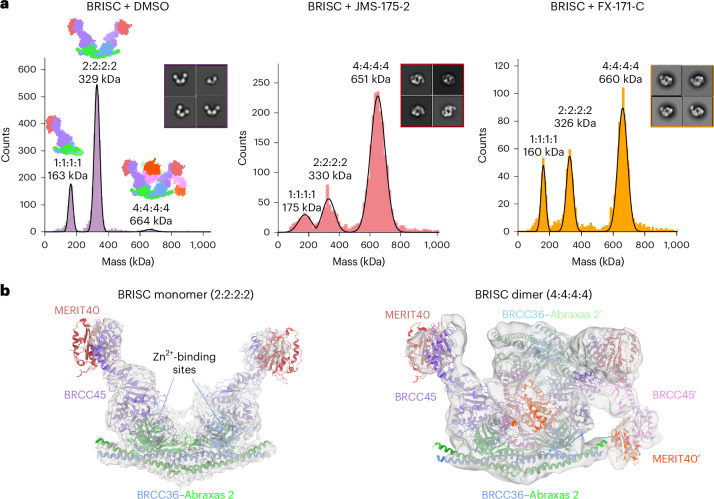
Inhibitors stabilize a BRISC dimer. **a**, Mass photometry histograms of purified BRISC in absence (DMSO, left) and presence (JMS-175-2, middle; FX-171-C, right) of inhibitors. Insets, corresponding negative-stain EM 2D classes of BRISC mixed with DMSO or inhibitors. **b**, Left, cryo-EM density map of a BRISC monomer with a BRISC model (PDB 6H3C) rigid-body fitted (dust cleaning size: 7.4, map threshold: 0.0907). Right, cryo-EM density map of a BRISC dimer with two BRISC models rigid-body fitted. Maps are outputs from nonuniform refinement in cryoSPARC.

To understand the molecular basis of BRISC inhibition by the JMS-175-2 inhibitor series and to determine the small-molecule-binding site, we characterized the complex by mass photometry and cryo-EM. Single-molecule mass photometry measurements in the absence of any inhibitors revealed three populations of purified BRISC. The major population corresponded to a single BRISC complex with four subunits at a 2:2:2:2 ratio (Fig. 2a, left), consistent with negative-stain EM two-dimensional (2D) class averages (Fig. 2a, left inset) and with previous studies^17,19,44^. We also observed a population at 163 kDa, which may correspond to a dissociated 1:1:1:1 complex or the minimally active BRCC36–Abraxas 2 super dimer^18^. Surprisingly, we also observed a third population, consisting of 2–5% of the particles, with an estimated molecular weight of ~664 kDa. This corresponds to the mass of two BRISC ‘monomer’ complexes with a predicted 4:4:4:4 stoichiometry.

Consistent with these measurements, we observed a higher-molecular-weight BRISC species in the cryo-EM data. We observed both BRISC monomer and BRISC dimer complexes in 2D class averages and in ab initio reconstructed maps (Extended Data Fig. 2d,e). The majority of particles corresponded to a monomeric complex, consistent with the BRISC–SHMT2 structure^20,44^ (Fig. 2b, Extended Data Fig. 2e,f and Table 1). In addition, the low-resolution cryo-EM reconstruction was consistent with a BRISC complex dimer (Fig. 2b, Extended Data Fig. 2g and Table 1). The conformation of this dimeric species was different from the symmetric BRISC and ARISC dimers previously reported in glutaraldehyde crosslinked samples imaged by negative-stain EM^19,20^ (Extended Data Fig. 2h). These observations suggest that BRISC has a propensity to dimerize, raising the possibility that low-level dimers may be regulated or stabilized by ligand binding.

**Table 1:** Cryo-EM data collection, refinement and validation statistics

	BRISC∆N∆C + FX-171-C	BRISC∆N∆C + JMS-175-2	BRISC (FL)
	EMD-17980, PDB 8PVY	EMD-18009, PDB 8PY2	
**Data collection and processing**			
Microscope	FEI Titan Krios G2		
Detector	Thermo Fisher Scientific Falcon 4	Thermo Fisher Scientific Falcon 4	Thermo Fisher Scientific Falcon 4
Energy filter	Thermo Fisher Scientific Selectris X		Thermo Fisher Scientific Selectris X
Energy filter slit (eV)	10		10
Magnification	×165,000	×96,000	×165,000
Voltage (kV)	300	300	300
Spot size	7	6	8
Illuminated area (µm)	0.8	1	0.53
Pixel size (Å)	0.71	0.82	0.74
Defocus range (µm)	−1.6 to −2.5	−1.7 to −3.1	−0.9 to −2.7
Total electron dose (e^−^ per Å^2^)	34.97	39.84	40.46
Exposure (s)	3.43	5.99	2.78
Number of frames	44	40	40
Electron dose per frame (e^−^ per Å^2^)	0.8	0.99	1
Videos collected	16,750	7,768	14,573
Acquisition mode	Counting	Counting	Counting
Symmetry imposed	*C*_1_	*C*_1_	*C*_1_, *C*_2_
Initial particle images (no.)	2,458,785	1,616,457	1,933,988
Final particle images (no.)	632,988	371,872	34,099 (monomer)
			32,283 (dimer)
Map resolution (Å)	3.02	3.32	7.1 (monomer)
			7.2 (dimer, *C*_1_)
			8.3 (dimer, *C*_2_)
FSC threshold	0.143	0.143	0.143
Map resolution range (Å)	2.8–5.4	3.1–7.8	4.0–7.0 (monomer)
			4.0–7.0 (dimer, *C*_1_)
			5.0–9.0 (dimer, *C*_1_)
**Refinement**			
Initial models used (PDB code)	6R8F, 6H3C	6R8F, 6H3C	
Model resolution (Å)	2.7	3.1	
FSC threshold	0.143	0.143	
Model composition			
Nonhydrogen atoms	35,736	35,730	
Protein residues	4,436	4,436	
Ligands	Zn: 4	Zn: 4	
	FX-171-C: 2	JMS-175-2: 2	
*B* factors (Å)			
Protein	60.66	22.53	
Ligands	18.46	11.95	
Root-mean-square deviation from ideal geometry			
Bond lengths (Å)	0.003	0.002	
Bond angles (°)	0.72	0.519	
**Validation**			
MolProbity score	1.97	1.94	
Clashscore	9.94	11.14	
Ramachandran plot			
Favored (%)	92.85	94.47	
Allowed (%)	7.15	5.53	
Disallowed (%)	0	0	
Rotamer outliers (%)	0.13	0.33	
Cβ outliers (%)	0	0	
*cis-*proline (%)	0	0	
CaBLAM outliers (%)	3.95	3.31	

**Extended Data Fig. 3:**
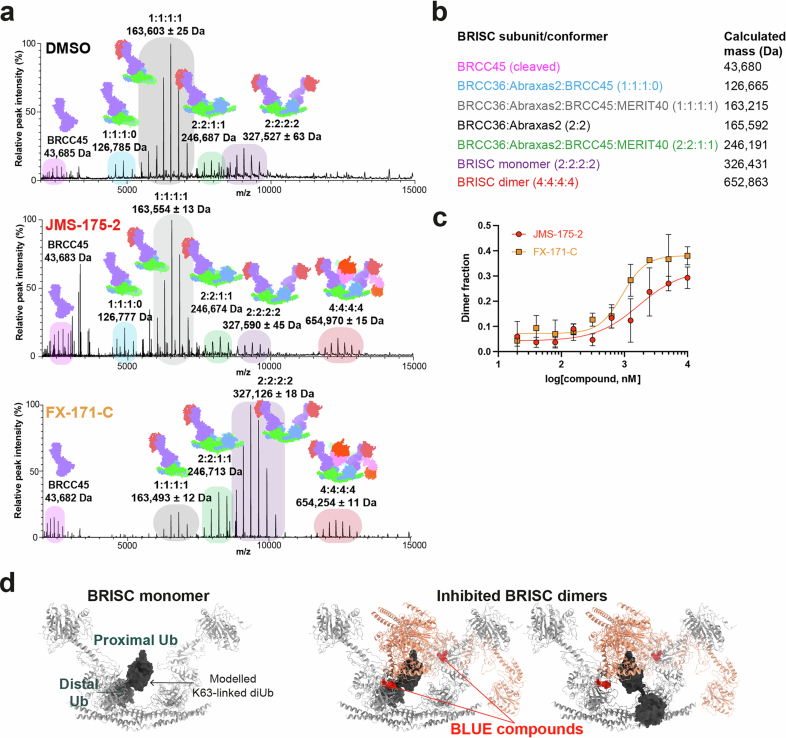
Identification of BRISC dimers in mass photometry and native mass spectrometry. **a**, Native mass spectra of BRISC mixed with DMSO (control), JMS-175-2, or FX-171-C. BRISC complexes and subcomplexes are highlighted. **b**, Table of calculated masses for different BRISC subcomplexes and super complexes. **c**, Mass photometry measurements of BRISC dimer at increasing inhibitor concentrations. Counts corresponding to BRISC dimer as a fraction of total counts are plotted. Data points are mean ± SEM from three independent experiments. **d**, *Left*, K63-linked diUb (dark grey) modelled on the MPN^+^ domain of BRCC36 in BRISC (light grey), based on the AMSH LP-diUb structure (PDB: 2ZNV). *Right*, Upon dimer formation, the second BRISC monomer sterically clashes with the proximal ubiquitin when it is bound to either BRCC36 active site. Source data

Interestingly, incubating purified BRISC with JMS-175-2 and FX-171-C resulted in a considerable mass shift to the 4:4:4:4 complex, which suggests that the inhibitor promotes BRISC dimer formation (Fig. 2a, middle and right). Negative-stain EM confirmed the oligomeric state on inhibitor addition, with 2D class averages resembling two U-shaped BRISC assemblies (Fig. 2a, middle and right insets). Using native MS, we confirmed the inhibitor-induced mass corresponds to a dimeric BRISC complex and BRISC dimers with 4:4:4:4 stoichiometry were detected after the addition of JMS-175-2 and FX-171-C (Extended Data Fig. 3a,b). Importantly, we also observed a dose-dependent increase in dimer formation by mass photometry for both JMS-175-2 and FX-171-C (Extended Data Fig. 3c). These data suggest an unexpected mode of action where inhibitor binding promotes a stable BRISC dimer complex of 16 subunits and molecular weight of 655 kDa.

### Cryo-EM structures reveal that BRISC inhibitors act as MGs

**Fig. 3:**
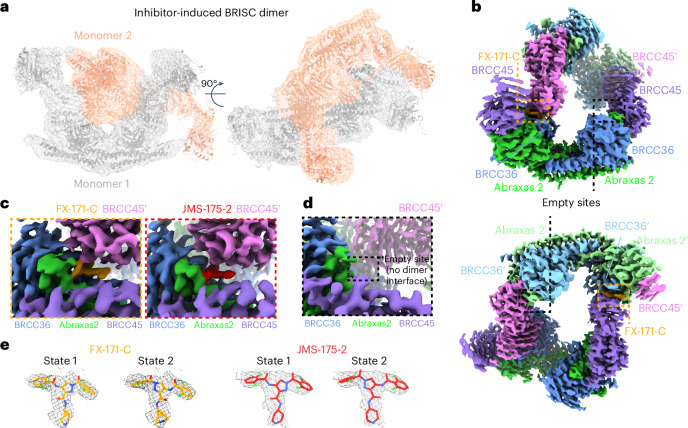
Cryo-EM structures of inhibited BRISC dimers. **a**, Cryo-EM density map of BRISC–FX-171-C costructure at 3.0 Å. BRISC monomers are shown as gray and salmon cartoon models and fitted to the cryo-EM map shown as a transparent surface at a 0.00224 threshold. The C termini of BRCC45 (residues 275–383) and MERIT40 are rigid-body fitted into the density. **b**, BRISC–FX-171-C cryo-EM density map at a 0.0165 threshold. BRISC subunits are colored by chain. The density corresponding to FX-171-C is colored orange and highlighted in orange boxes. The maps shown in **a**–**c** are locally filtered maps generated using RELION local resolution estimation. **c**, Close-up views of the indicated inhibitor density comparing binding sites for FX-171-C (left) and JMS-175-2 (right). **d**, Cryo-EM density at the equivalent sites of BRCC36, Abraxas 2 and BRCC45′ in the BRISC–FX-171-C costructure, where there is no dimer interface and no additional density corresponding to FX-171-C. The maps in **a**–**d** had dust cleaning (size 7.1) applied in ChimeraX. **e**, Structures of FX-171-C and JMS-175-2 modeled in state 1 and state 2. Cryo-EM density of the inhibitor after focused refinement represented as a mesh and displayed using the surface zone tool (FX-171-C, radius = 2.6; JMS-175-2, radius = 2.2) in ChimeraX.

**Extended Data Fig. 4:**
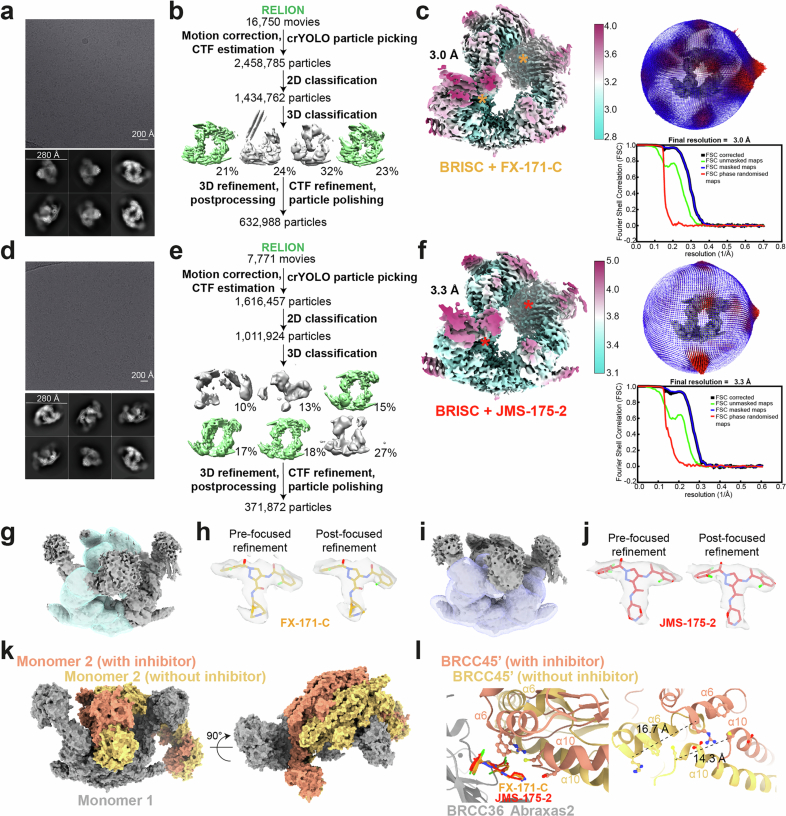
Cryo-EM processing of the BRISC-inhibitor co-complex. Figures **a**–**c** correspond to the BRISC-FX-171-C cryo-EM dataset. Figures **d**–**f** correspond to the BRISC-JMS-175-2 dataset. **a**, **d**, Representative micrographs (from 16,750 (FX-171-C) and 7,771 movies (JMS-175-2)) and 2D class averages. **b**, **e**, Image processing workflow. Green maps indicate selected classes used for 3D refinement. **c**, **f**, *Left*, cryo-EM density maps after 3D refinement for the final reconstructions used for model building. Asterisks indicate BLUE compound binding sites. *Right*, final maps with corresponding Euler angular distribution with rod heights proportional to the number of particles in each direction. FSC curves with resolution calculated using the gold standard FSC cut-off at 0.143 frequency. **g**, Mask used for focused refinement of the BRISC-FX-171-C map. **h**, Chemical structure of FX-171-C fitted into EM density before (*left*) and after (*right*) focused refinement. Cryo-EM density visualised using the surface zone tool in ChimeraX; left, radius 2.04, right, radius 2.60. **i**, Mask applied during refinement of BRISC-JMS-175-2 map. **j**, Chemical structure of JMS-175-2 fitted into EM density before (*left*) and after (*right*) focused refinement. Cryo-EM density visualised using the surface zone tool in ChimeraX; left, radius 2.20, right, radius 2.41. **k**, Overlay of two BRISC dimers aligned on one BRISC molecule (grey) for comparison. Models are represented as surfaces. Orange, model fitted to the BRISC-FX-171-C structure shown in **c**, yellow, BRISC models rigid-body fitted in the cryo-EM density of the asymmetric dimer shown in Extended Data Fig. 2g. The yellow molecule is shifted relative to the orange molecule. **l**, Models described in **k**, focussed on the small molecule binding site highlighting the shift in the BRCC45’ α6 and α10 helices.

To determine the precise mechanism by which a small molecule induces the formation of a multimeric DUB complex, we solved two costructures of BRISC complexes bound to FX-171-C and JMS-175-2. We observed a high proportion (>95%) of BRISC dimers after incubation with each inhibitor. After three-dimensional (3D) refinement and postprocessing, we obtained cryo-EM maps at resolutions of 3.0 Å (FX-171-C) and 3.3 Å (JMS-175-2) (Fig. 3a, Extended Data Fig. 4a–f and Table 1). The BRISC–inhibitor structures consist of a BRISC dimer of 16 subunits (stoichiometry 4:4:4:4), where the BRCC45–MERIT40 arms of one BRISC monomer hook around the BRCC45–MERIT40 arm of a neighboring BRISC molecule (BRISC′), bridging the BRCC36–Abraxas 2 super dimer (Fig. 3b). Modeling of K63-linked diubiquitin substrate in this conformation suggests that the recruitment of a second BRISC occludes the BRCC36 active sites by sterically blocking chain binding and catalysis (Extended Data Fig. 3d).

We observe the highest resolution (2.8–3.6 Å) in the core of the BRISC dimer structures, consisting of the BRCC36–Abraxas 2 super dimer and the BRCC45′ subunit that participates in the dimer interface (Fig. 3b and Extended Data Fig. 4c, f). The resolution is lower (7–12 Å) for the extreme C termini of BRCC45 and MERIT40 (arm regions), thus limiting accurate model building of these regions. This is likely because of the flexible nature of the arm regions and is consistent with our previous observations of the BRISC–SHMT2 cryo-EM structure^44^. Because of the lower resolution of the map beyond the second ubiquitin E2 variant (UEV) domain of BRCC45 and for MERIT40, we rigid-body fitted BRCC45 UEV-C (residues 275–383) and MERIT40 from previous BRISC–SHMT2 structures^20,44^.

The binding interface formed by BRCC36, Abraxas 2 and BRCC45′ is also formed at the opposite site of the dimer structure (BRCC36′, Abraxas 2′, BRCC45). At both interfaces, we observe additional density that is not attributed to either BRISC monomer; this density has the size and shape expected for each inhibitor (Fig. 3c). Importantly, the equivalent BRCC36–Abraxas 2 surface that is not in contact with BRCC45′ from an opposing BRISC monomer does not contain additional cryo-EM density (Fig. 3d). The extra density is present in the same location for both the FX-171-C and JMS-175-2 maps (Fig. 3c), indicating a similar binding mode for both compounds. Because of the slight tilting of the BRISC′ monomer resulting in an asymmetric dimer, there is one inhibitor bound per BRISC molecule and two inhibitors per BRISC dimer (4:4:4:4:2 stoichiometry).

Focused refinement using a mask comprising the core of the BRISC dimer moderately improved the density for the FX-171-C compound (Extended Data Fig. 4g,h). Likewise, applying a mask on the highest resolution half of the JMS-175-2 map also improved the density for JMS-175-2 (Extended Data Fig. 4i,j). Because of the presence of two dichlorobenzoyl rings in each compound, we could not unambiguously determine the orientation of the dichlorobenzoyl moieties in the cryo-EM densities and, thus, modeled the ligands in two orientations: state 1 and state 2 (Fig. 3e).

**Extended Data Fig. 5:**
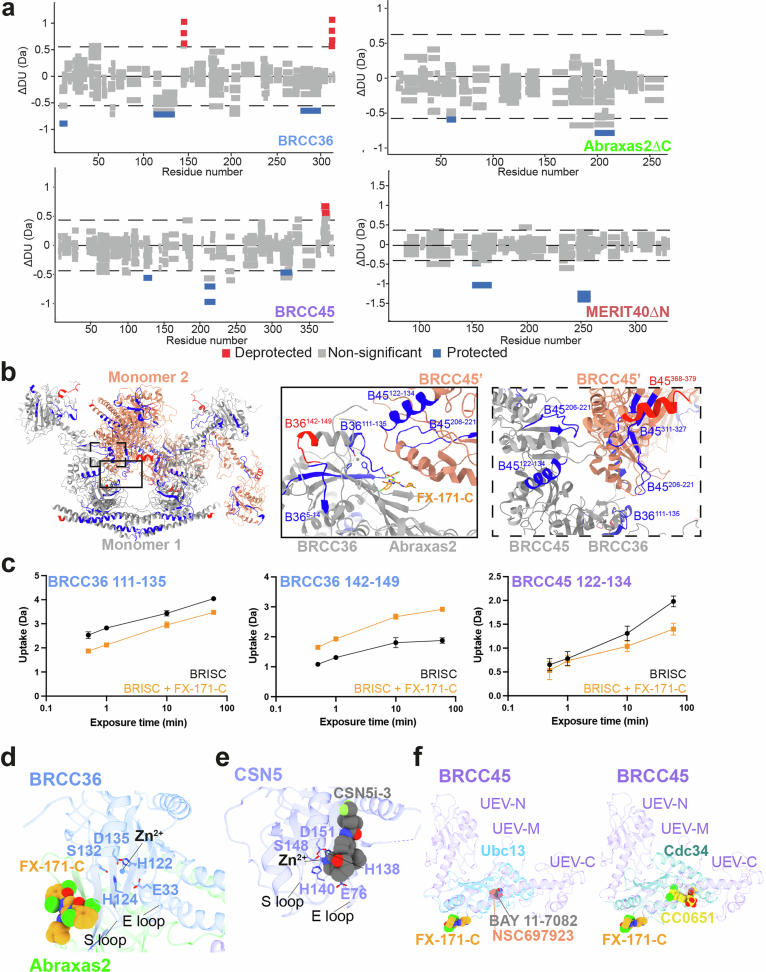
Observed changes in BRISC subunit solvent accessibility and secondary structure in the presence of FX-171-C by HDX-MS. **a**, Wood’s plots generated with Deuteros showing the differences in deuterium uptake over all four HDX timepoints from three technical replicates, comparing BRISC in the absence and presence of FX-171-C. Regions highlighted in grey indicate peptides with no significant change, calculated using a 99% confidence interval, between the two conditions. The dashed line indicates the 99% confidence limit. Peptides are coloured in red to indicate deprotection in the presence of inhibitor, and blue to indicate protection. **b**, Peptides mapped onto BRISC dimer structure, highlighting peptides near BLUE binding site and at the interface of two BRCC45 subunits. B36 = BRCC36; B45 = BRCC45. **c**, Example deuterium uptake curves in the absence and presence of FX-171-C. Data points are mean ± SEM from three technical replicates. **d**, BLUE compounds are allosteric inhibitors and do not disrupt the BRCC36 Zn^2+^ binding site. **e**, CSN5 active site in complex with inhibitor CSN5i-3 (PDB: 5J0G). **f**, *Left*, BRCC45 UEV-M bound to FX-171-C aligned to Ubc13 in complex with BAY 11-7082 (PDB: 4ONN) and NSC697923 (PDB: 4ONM). *Right*, BRCC45 UEV-M bound to FX-171-C aligned to Cdc34 in complex with CC0651 (PDB: 3RZ3).

Next, we examined the conformational changes induced by FX-171-C using differential hydrogen deuterium exchange (HDX)–MS analysis. Measuring differences in deuterium uptake, detected at the peptide level, in the absence and presence of FX-171-C enabled us to analyze the structural changes after inhibitor binding (Extended Data Fig. 5a). For example, regions of protection upon FX-171-C addition were identified in BRCC36 (residues 111–135) and BRCC45 (residues 122–134), which are consistent with the small-molecule-binding site and interaction interfaces identified in our cryo-EM structures (Extended Data Fig. 5b,c). We also observed deprotection of a BRCC36 peptide (142–149), indicative of a change in solvent accessibility near the enzyme active site. Protected peptides in BRCC45 (residues 206–221 and 311–327) suggest further interactions between BRCC45 subunits from opposing BRISC monomers (Extended Data Fig. 5b). Moreover, deprotected peptides in the BRCC36–Abraxas 2 coiled-coil and the C termini of BRCC45 and MERIT40 subunits indicate additional and far-reaching conformational changes induced by inhibitor binding (Extended Data Fig. 5b).

Collectively, these analyses establish that the inhibitors are BRISC MGs (BLUEs) that stabilize two BRISC octamers to form a BRISC dimer with 16 subunits. BLUEs bind at a composite site of three interacting proteins: BRCC36 and Abraxas 2 from one BRISC monomer and BRCC45′ from a second BRISC monomer. The inhibitor-induced dimer is an inactive conformation, whereby ubiquitin chain binding and processing is blocked.

### The BLUE-binding pocket

BLUE compounds bind near the catalytic zinc but do not engage it or BRCC36 active-site residues (Extended Data Fig. 5d), consistent with noncompetitive enzyme inhibition (Extended Data Fig. 2b). BLUE compounds are examples of noncompetitive JAMM/MPN DUB inhibitors, in contrast to JAMM/MPN DUB inhibitors that target the zinc-binding site^31–33,45^ (Extended Data Fig. 5e). In addition to using an unexploited binding site for JAMM/MPN DUBs, BLUE compound engagement of the middle BRCC45 UEV domain highlights another unexpected compound-binding surface in E2 folds. Unlike BAY 11-7082 and NSC697923 (inhibitors of Ubc13), BLUEs neither engage the UEV pseudocatalytic site^46^ nor bind to an allosteric site exemplified by the Cdc34 E2 inhibitor, CC0651 (ref. ^47^) (Extended Data Fig. 5f).

**Fig. 4:**
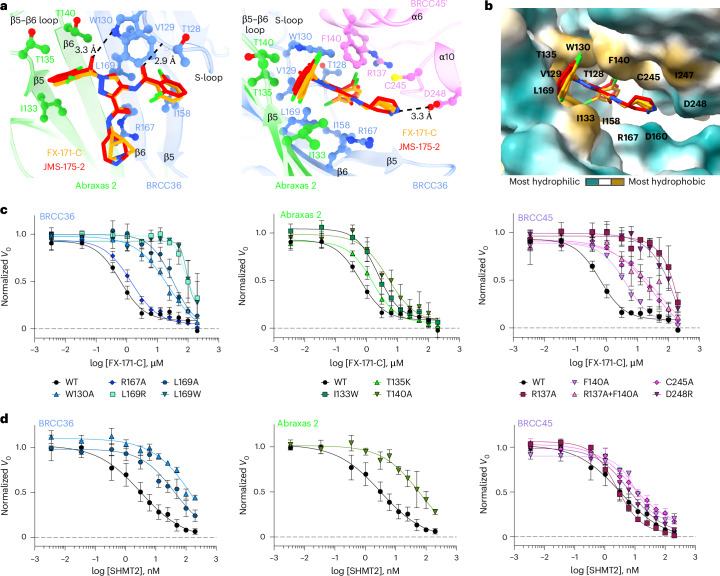
Analysis of the BLUE compound-binding site. **a**, Ball-and-stick model of FX-171-C and JMS-175-2 binding to BRCC36, Abraxas 2 and BRCC45. Hydrogen bonds are shown as black dashed lines and residues studied by mutagenesis are indicated. **b**, The BLUE compound-binding pocket shown as a surface and colored by hydrophobicity. **c**, FX-171-C inhibition of BRISC DUB activity with BRCC36 (left), Abraxas 2 (middle) and BRCC45 (right) mutants. **d**, SHMT2 inhibition of the same BRISC mutants as in **c**. Data in **c**,**d** are the mean ± s.e.m. of three independent experiments carried out in technical duplicate. Source data

The local resolution of our cryo-EM structures at the dimer interface is ~2.8 Å (FX-171-C map, Fig. 3c), which is sufficient to identify residues from each BRISC monomer that contribute to the inhibitor-binding pocket. In BRCC36, BLUEs bind between the S-loop (β4–α3) and the β5–β6 strands. In Abraxas 2, BLUE compounds interact with the β5 strand and β5–β6 loop (Fig. 4a, left). Two α-helices (α6 and α10) from the BRCC45′ subunit also line the inhibitor-binding pocket (Fig. 4a, right). The two dichlorobenzoyl moieties of JMS-175-2 and FX-171-C sit in a hydrophobic groove formed by BRCC36 S-loop residues T128 and W130, as well as residues I158 and L169 (Fig. 4b). Abraxas 2 I133 and BRCC45′ F140, C245 and I247 also contribute to the hydrophobic binding pocket. The JMS-175-2 piperidine ring and FX-171-C pyrrolidine ring extend into a hydrophilic region encompassing BRCC36 D160 and R167 and BRCC45′ D248.

**Extended Data Fig. 6:**
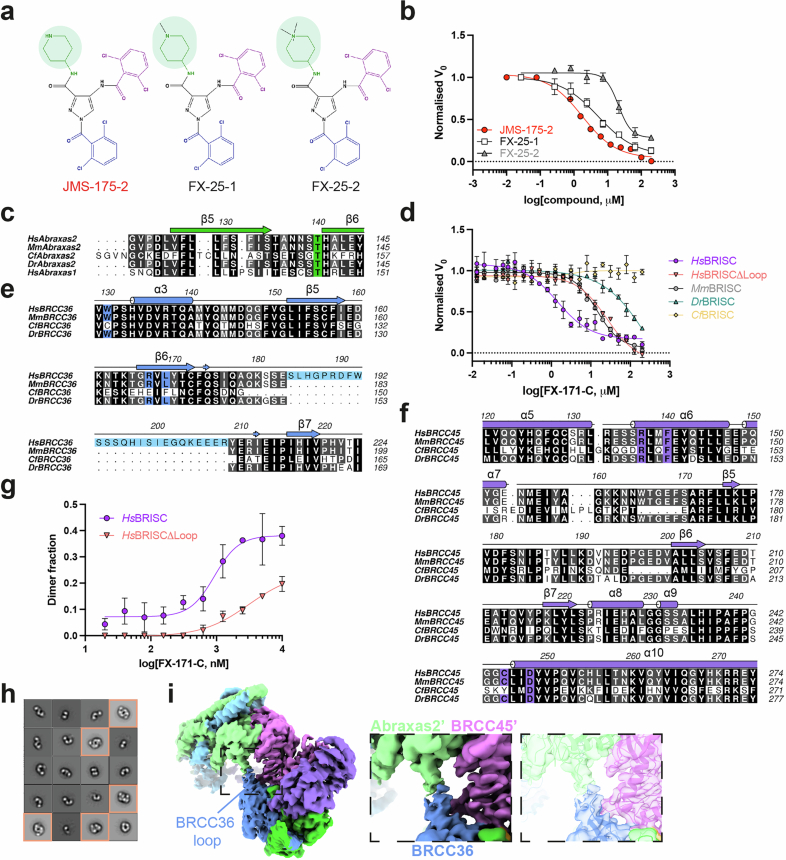
BLUE compounds are allosteric inhibitors and selective for human BRISC. **a**, Chemical structure of JMS-175-2 and analogues FX-25-1, FX-25-2, which have substitutions in the piperidine ring (highlighted in green). **b**, Dose-response inhibition of BRISC by indicated compounds. IC_50_ values: JMS-175-2 = 3.8 µM, FX-25-1 = 5.2 µM, FX-25-2 = 21 µM. Data points are mean ± SEM of three independent experiments carried out in technical duplicate. **c**, Multiple sequence alignment (black = conserved, white = not conserved) of Abraxas1 and Abraxas2 from indicated species. Coloured boxes indicate BLUE interacting residues. **d**, FX-171-C inhibition of different BRISC orthologues. *Hs - H. sapiens, Mm - M. musculus, Dr - D. rerio, Cf - C. floridanus*. Data points are mean ± SEM of three independent experiments carried out in technical duplicate. **e**, **f**, Multiple sequence alignment of **e**, BRCC36 and **f**, BRCC45 from indicated BRISC orthologues. Residues are coloured as in **c**. **g**, Mass photometry analyses of dimer formation with FX-171-C for *Hs*BRISCΔNΔC and *Hs*BRISCΔLoop. Fraction of counts corresponding to BRISC dimer are plotted. Data points are mean ± SEM of three independent experiments. **h**, Negative stain EM 2D class averages of *Hs*BRISCΔLoop incubated with FX-171-C. 22% of particles in the 2D class averages correspond to BRISC dimers. **i**, BRISC-FX-171-C cryo-EM density map highlighting an extended loop in BRCC36 (dust cleaning size 7.1, map threshold 0.0044). Source data

BRCC36 forms two hydrogen bonds with the BLUE compounds. In both state 1 and state 2, the amide backbone of V129 and the W130 side chain (BRCC36 S-loop) form hydrogen bonds with the two amide oxygens either side of the central pyrazole ring. BRCC45′ F140 forms aromatic stacking interactions with the central pyrazole ring, and the BRCC45′ D248 forms a hydrogen bond with the amine group in the JMS-175-2 piperidine or FX-171-C pyrrolidine ring (Fig. 4a). Consistent with this interaction, analogs containing methyl substitutions of the piperidine ring showed reduced inhibition of BRISC activity (Extended Data Fig. 6a,b). BRCC45′ R137 forms a hydrogen bond with the BRCC45′ loop containing C245 to stabilize the BRCC45′ α10 helix that lines the compound-binding site.

Interestingly, we do not observe the same compound-binding pocket in the asymmetric (no inhibitor) conformation (Fig. 2b). We rigid-body fitted two BRISC molecules into the cryo-EM density of the asymmetric dimer conformation and observe a shifted BRISC′ molecule relative to the BRISC–FX-171-C model (Fig. 2b and Extended Data Fig. 4k). The BRCC45′ α6 and α10 helices that line the compound-binding site are shifted in the compound-bound conformation (Extended Data Fig. 4l). These structural insights suggest that MG compound binding not only induces BRISC dimerization but also alters the conformation of pre-existing (and low-level) BRISC dimers.

### A human-specific BRCC36 loop promotes BRISC dimer formation

BLUE compounds are highly selective for BRISC over other JAMM/MPN DUBs, including the closely related ARISC complex that shares the BRCC36 catalytic subunit (Fig. 1e,g). Cryo-EM structures reveal that BLUEs directly engage the Abraxas 2 subunit. Sequence alignment of Abraxas 1 (ARISC) and Abraxas 2 (BRISC) illustrated divergence in the primary amino acid sequence near the BLUE compound-binding site (β5–β6 loop) (Extended Data Fig. 6c), which likely contributes to the selectivity of BLUEs for BRISC over ARISC.

To further probe compound selectivity, we tested FX-171-C inhibition of BRISC complexes from metazoan orthologs: mouse (*Mus musculus)*, zebrafish (*Danio rerio)* and ant (*Camponotus floridanus)*. FX-171-C had the highest potency toward human BRISC over mouse and zebrafish BRISC, whilst there was no inhibition of ant BRISC (Extended Data Fig. 6d). Analyzing the BRISC–BLUE interaction interface explains the high specificity of BLUE compounds for human BRISC over ant BRISC. BRCC36 W130 and L169 and the Abraxas 2 β5–β6 strands, which line the inhibitor-binding pocket (Fig. 4a), are not conserved in *Cf*BRCC36 and *Cf*Abraxas 2 (Extended Data Fig. 6c,e). BRCC45 C245, which contributes to compound binding, is also not conserved in *Cf*BRISC (Extended Data Fig. 6f).

Analyzing the selectivity between human and mouse BRISC suggested a possible contributor of dimer formation. Human and mouse BRISC share over 97% sequence identity, yet FX-171-C is approximately ten times more potent as an inhibitor of human BRISC. The major difference is an extended loop region of 25 amino acids in human BRCC36 (residues 184–208) (Extended Data Fig. 6e). Deletion of this loop in human BRISC reduced inhibitor sensitivity approximately tenfold to a similar IC_50_ for mouse BRISC lacking the same loop (Extended Data Fig. 6d). Consistent with the idea that the loop region mediates dimer formation, we observed fewer dimers for the human BRISCΔLoop construct upon inhibitor addition in mass photometry and negative-stain EM (Extended Data Fig. 6g,h). The cryo-EM density corresponding to the BRCC36 loop extends toward an Abraxas 2′ subunit from the opposing BRISC molecule, further supporting a role for this loop in mediating BRISC dimerization (Extended Data Fig. 6i). Interestingly, humans have two BRCC36 isoforms, with one lacking this loop region, suggesting that it is possible to design MGs that display selectivity not only within the same enzyme family but also across orthologous species and splice variants.

### Residues in three BRISC subunits contribute to inhibition

**Extended Data Fig. 7:**
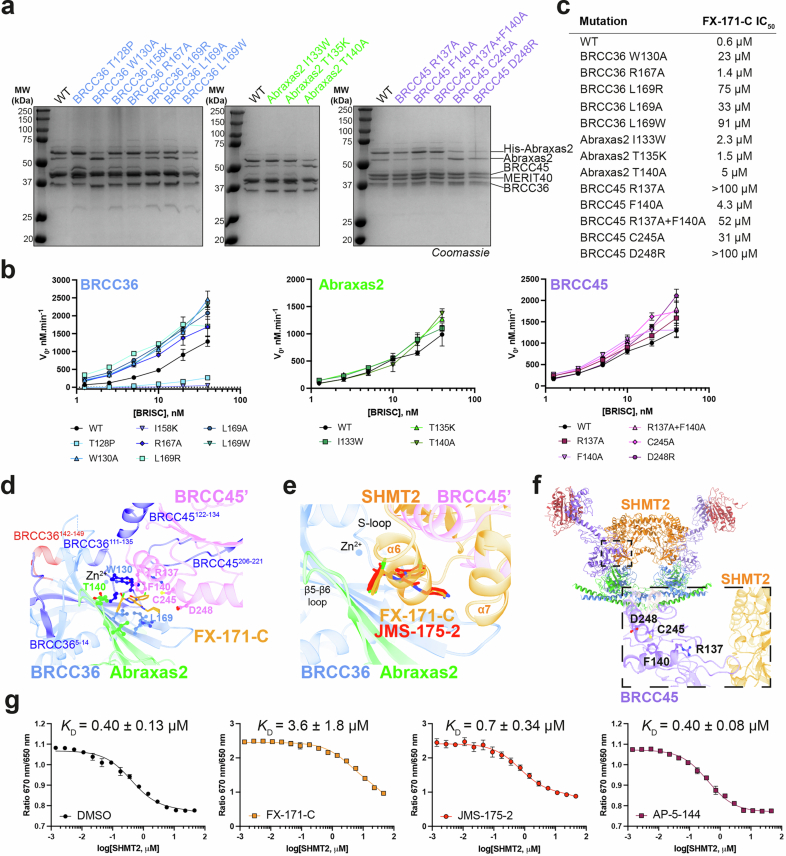
Determining the DUB activity, inhibitor sensitivity, and SHMT2 inhibition of structure-guided mutants. **a**, SDS-PAGE analysis of purified BRISC mutants. Each purified protein sample was generated from one purification. Uncropped gels are in Source Data Extended Fig. 7. **b**, Activity of BRISC mutants against an IQF di-ubiquitin substrate. Data points are mean ± SEM of three independent experiments carried out in technical duplicate. **c**, FX-171-C IC_50_ values from inhibition assays shown in Fig. 4. **d**, Protected and deprotected peptides from HDX-MS mapped onto the FX-171-C binding site. Peptides are coloured blue to indicate protection and red to indicate deprotection, after incubation with FX-171-C. **e**, Superimposition of the SHMT2 dimer from BRISC-SHMT2 structure (PDB: 6R8F) onto BRISC-FX-171-C dimer structure. SHMT2 α6 helix clashes with the BLUE binding site. **f**, Mutated residues in BRCC45 are not in close proximity to the SHMT2 binding site in the BRISC-SHMT2 structure (PDB: 6R8F). **g**, Spectral Shift (Dianthus) assays measure the binding of SHMT2(A285T) to labelled His-BRISC in the absence and presence of compounds. *K*_D_ is calculated by plotting the ratio of the fluorescence intensities at 650 nm and 670 nm against SHMT2 concentration, with a GraphPad Prism equation for one-site total binding. Data points are mean ± SEM of three independent experiments. Source data

The cryo-EM structures of BRISC in complex with MGs allowed us to make specific substitutions to probe the BRISC–BLUE interaction site and to assess the contribution of each interacting residue for inhibition. We substituted residues from BRCC36, Abraxas 2 and BRCC45 and purified 15 mutant BRISC complexes from insect cells (Extended Data Fig. 7a). Because of the proximity of some residues to the BRCC36 active site, we assessed BRISC DUB activity against a fluorogenic diubiquitin substrate. Two BRCC36 mutants (T128P and I158K) were inactive and we determined which substitutions conferred a reduction in inhibitor sensitivity for the remaining 13 active mutant complexes (Extended Data Fig. 7b).

BRCC36 W130A and L169A/R/W mutants showed severely reduced inhibition by FX-171-C (>100-fold over wild-type (WT) complex), whilst BRCC36 R167A remained inhibitor sensitive (Fig. 4c and Extended Data Fig. 7c). Abraxas 2 mutant T140A was moderately affected, exhibiting an IC_50_ tenfold higher than BRISC WT, while Abraxas 2 I133W and T135K had moderate to little effect on inhibitor sensitivity (Fig. 4c and Extended Data Fig. 7c). We also substituted BRCC45 residues (R137A, F140A, C245A and D248R) and all had reduced sensitivity to inhibition when compared to WT BRISC complexes (Fig. 4c and Extended Data Fig. 7c). These data validate the BLUE compound-binding sites identified by cryo-EM and the reduced sensitivity observed for the BRCC45 mutants confirmed the MG mechanism of inhibition.

The reduced inhibition after substituting residues surrounding the inhibitor-binding pocket is consistent with regions of change in solvent accessibility observed by HDX–MS after incubation with FX-171-C. BRCC36 W130 lies within a protected loop (residues 111–135) and the W130A mutant has reduced inhibitor sensitivity (Fig. 4c and Extended Data Fig. 7d). Substitutions of BRCC45 residues R137, F140, C245 and D248 led to reduced FX-171-C inhibition and these residues are in close proximity to protected regions of BRCC45: residues 122–134 and 206–221 (Fig. 4c and Extended Data Fig. 7d). In contrast, BRCC36 L169 and Abraxas 2 T140 are required for inhibition but do not exhibit changes in solvent accessibility in HDX–MS.

### MGs and SHMT2 share a binding pocket

The metabolic enzyme SHMT2 interacts with BRISC to regulate IFNAR1 signaling^44^. Interestingly, the SHMT2-binding site on BRISC overlaps with the BLUE compound-binding site (Extended Data Fig. 7e). Indeed, some of the residues we substituted to validate inhibitor binding also contribute to the SHMT2 interaction interface. As SHMT2 is a potent endogenous inhibitor of BRISC DUB activity^44^, we assessed whether BRISC mutants were still inhibited by SHMT2 (Fig. 4d). BRCC36 W130A and L169A and Abraxas 2 T140A showed reduced BRISC inhibition by SHMT2, indicating that these substitutions also disrupt SHMT2 binding to BRISC. By contrast, the BRCC45 mutants were inhibited by SHMT2 with a similar IC_50_ to BRISC WT, which is consistent with these BRCC45 residues being distant from the BRISC–SHMT2 binding interface in the context of the BRISC monomer (Extended Data Fig. 7f). Therefore, both the BLUE compounds and the endogenous inhibitor SHMT2 share a common interaction site.

To investigate SHMT2 and BLUE compound competition with BRISC, we used a spectral shift assay (Dianthus) to determine the *K*_D_ of the BRISC–SHMT2 interaction in the absence and presence of FX-171-C. We measured a *K*_D_ of 0.4 ± 0.1 μM for SHMT2 with labeled BRISC (Extended Data Fig. 7g). The *K*_D_ of SHMT2 for BRISC was reduced to 3.6 ± 1.8 μM after incubation with FX-171-C and 0.7 ± 0.3 μM after incubation with JMS-175-2. There was no change in affinity with the negative control compound AP-5-144 (Extended Data Fig. 7g). These data demonstrate direct competition between BLUEs and SHMT2 and show that BLUEs can reduce SHMT2 binding to BRISC, which, despite being an endogenous inhibitor, is required for immune signaling in cells^44^.

### BLUE compounds reduce IFN signaling

**Extended Data Fig. 8:**
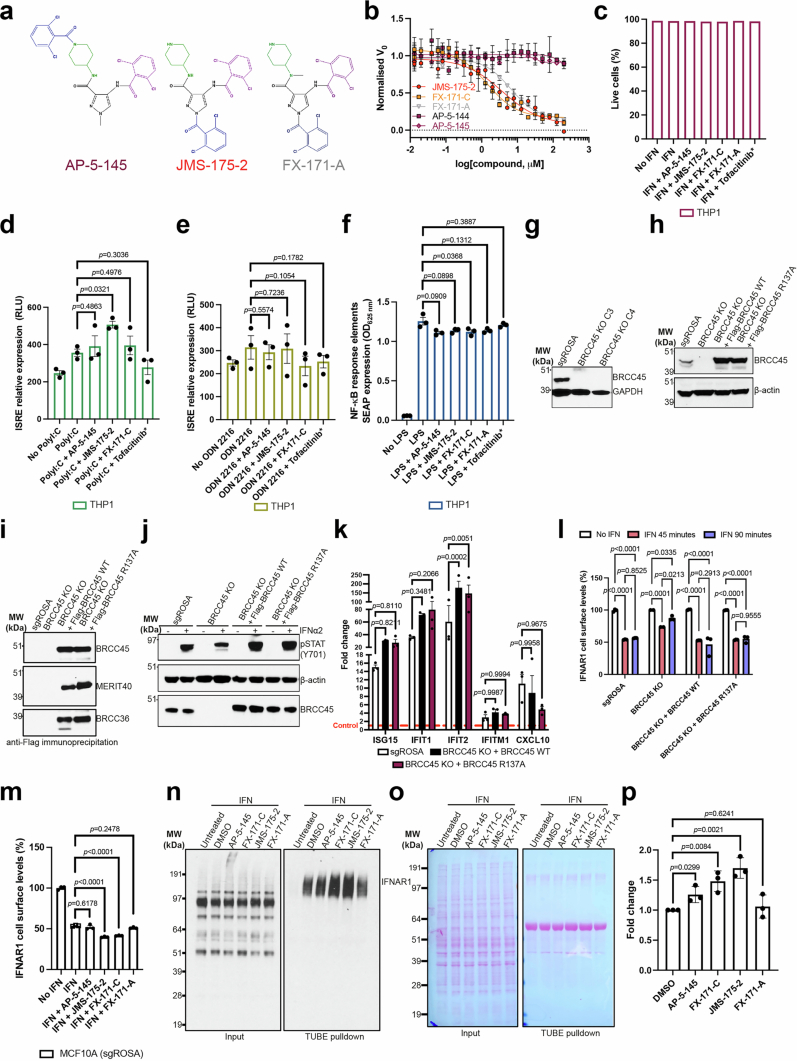
Establishing the effect of BLUE compound treatment on immune signalling pathways, IFNAR1 surface levels, and IFNAR1 ubiquitylation. **a**, Chemical structures of compounds AP-5-145, JMS-175-2, and FX-171-A. **b**, Dose-response inhibition of BRISC by indicated compounds. Data points are mean ± SEM from at least two independent experiments carried out in duplicate. **c**, Bar chart representing percentage of live cells across all conditions for ISRE expression and FACS analysis in THP-1 cells shown in Fig. 5a, h. THP-1 cells were treated with/without hIFNα2 (25 ng/mL) and either 4 μM inhibitor (JMS-175-2, FX-171-C, FX-171-A), 4 μM AP-5-145 negative control, DMSO control (0.1%), or JAK/STAT inhibitor Tofacitinib (*0.4 μM) for 16 h. Bars represent the means from three independent experiments. **d**, **e**, Luciferase analysis of the ISRE in THP-1 supernatant after stimulation with **d**, polyI:C (1 µg/mL), or **e**, ODN 2216 (1 µM) and treatment with either 4 μM inhibitor (JMS-175-2, FX-171-C, FX-171-A), 4 μM AP-5-145 negative control, or JAK/STAT inhibitor Tofacitinib (*0.4 μM) for 16 h. **f**, NF-κB pathway activity analysed by SEAP activity in THP-1 supernatants. Optical density measured at 625 nm. Data points in **d**–**f** are from three independent experiments. **g**, BRCC45 protein levels in selected clones after knock out in MCF10A *Cas*9 cells. sgROSA was used as a CRISPR *Cas*9 control. **h**, BRCC45 expression in whole cell lysates from MCF10A *Cas*9 cells expressing CRISPR control (sgROSA), BRCC45 WT, and BRCC45 R137A. **i**, Anti-Flag co-immunoprecipitation performed in indicated MCF10A cell lines. BRISC complex subunits were detected using specific antibodies. **j**, MCF10A cell lines were treated with and without hIFNα2 (75 ng/mL) for 1 h. STAT1 Tyr701 phosphorylation, BRCC45 and total protein levels (β-actin) were detected using specific antibodies. **k**, MCF10A *Cas*9 cells were treated with /without hIFNα2 for 4 h. Expression of interferon-induced genes *ISG15*, *IFIT1*, *IFIT2*, *IFITM1*, and *CXCL10* were normalised to *RNA18S* and presented as fold change to own no IFN treated control. Data points are from three independent experiments. **l**, MCF10A cells (sgROSA, BRCC45 KO, BRCC45 WT and BRCC45 R137A) were treated with/without hIFN-Iα (50 ng/mL) for either 45 or 90 min. **m**, SgROSA MCF10A cells were treated with/without hIFN-Iα (50 ng/mL) and either 5 μM inhibitor (JMS-175-2, FX-171-C, FX-171-A), 5 μM negative control AP-5-145 or DMSO (0.1%) for 90 min. In **l**, **m**, IFNAR1 cell surface levels (%) were quantified using FACS analysis and calculated as a percentage of no IFN stimulation, and data points are from three independent experiments. **n**, Anti-IFNAR1 immunoblots of TUBE-pulldown. *Left*, input samples after stimulation with IFNα2, treatment with BLUE inhibitors and cell lysis. *Right*, ubiquitylated IFNAR1 isolated with agarose-TUBE beads after IFNα2 stimulation. The blot shown is representative of three biological replicates. Raw, uncropped Western blots are in Source Data Extended Fig. 8. **o**, Ponceau stained membrane of blots shown in **n**, prior to antibody incubation. **p**, Densitometry quantification of Western blot shown in **n**, and two other biological replicates (n = 3). In **d**–**f**, paired, two-tailed *t*-tests were used to compare compound treated cells with DMSO control cells. In **k**, a two-way ANOVA with Tukey’s multiple comparisons test was used to gene expression levels for five genes in both BRCC45 WT and BRCC45 R137A cells to the sgROSA MCF10A cells. In **l**, a two-way ANOVA with Tukey’s multiple comparisons test was performed to compare no IFN vs. IFN 45 min vs. IFN 90 min. Error bars represent ± SEM. In **m**, **p**, statistical analyses were performed using unpaired, two-tailed *t*-tests to compare compound treated cells with DMSO control cells. Source data

To investigate the effects of BLUE compounds on IFN signaling, we used a THP-1 cell line with a stably integrated, inducible luciferase reporter for the IFN-regulatory factor pathway. For additional controls alongside FX-171-C and JMS-175-2, we tested three further compounds: FX-171-A, AP-5-145 and tofacitinib, a JAK inhibitor. FX-171-A has a similar chemical structure to JMS-175-2 but is a less potent BRISC inhibitor (IC_50_ = 6.8 μM) (Extended Data Fig. 8a,b). AP-5-145 is an N-methylated analog of AP-5-144, the other possible stereoisomer synthesized at the beginning of this study, and has no inhibitory effect against BRISC (Fig. 1c and Extended Data Fig. 8b). We added a methyl group to the pyrazole ring to avoid potential cyclin-dependent kinase 2 inhibition in cells and to increase cell permeability when used as a negative control. We observed no cell death with any of the compounds tested up to 4 μM (Extended Data Fig. 8c).

**Fig. 5:**
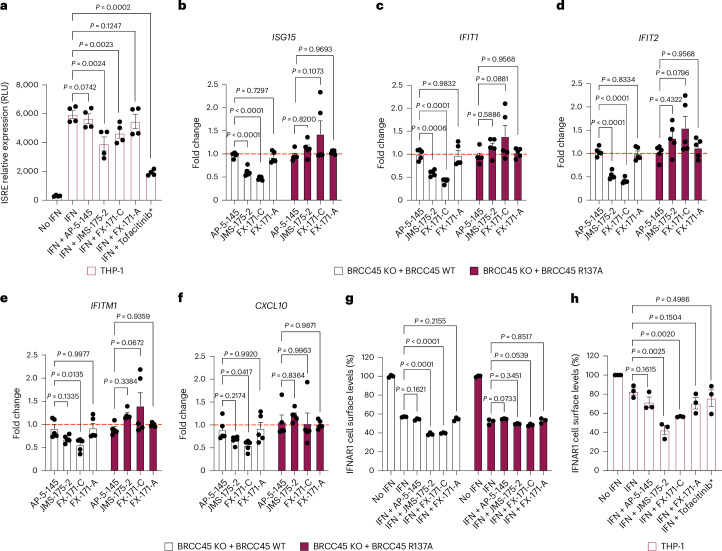
BLUE compounds reduce ISG expression and IFNAR1 internalization in cells. **a**, THP-1 cells were treated with or without human (h)IFNα2 (25 ng ml^−1^) and 4 μM inhibitor (JMS-175-2, FX-171-C or FX-171-A), 4 μM negative control (AP-5-145), DMSO control (0.1%) or JAK–STAT inhibitor tofacitinib (0.4 μM) for 16 h. Luciferase analysis of the ISRE in THP-1 supernatant in relative light units (RLU). Data points are from four independent experiments. **b**–**f**, MCF10A Cas9 cells expressing BRCC45 WT and BRCC45 R137A were treated with hIFNα2 (75 ng ml^−1^) and 2.5 μM inhibitor (JMS-175-2, FX-171-C or FX-171-A), 2.5 μM negative control (AP-5-145) or DMSO control (0.1%) for 4 h. Expression of indicated IFN-induced genes (*ISG15* (**b**), *IFIT1* (**c**), *IFIT2* (**d**), *IFITM1* (**e**) and *CXCL10* (**f**)) normalized to *RNA18S* is presented as the fold change to a control treated with IFN + DMSO. Data points are from four independent experiments. **g**, MCF10A cells (BRCC45 WT and BRCC45 R137A) were treated with or without hIFNα1 (50 ng ml^−1^) and 5 μM inhibitor (JMS-175-2, FX-171-C or FX-171-A), 5 μM negative control (AP-5-145) or DMSO control (0.1%) for 90 min. IFNAR1 cell surface levels (%) were quantified using FACS analysis and calculated as a percentage of no IFN stimulation. Data points are from three independent experiments. **h**, THP-1 cells were treated with or without hIFNα2 (25 ng ml^−1^) and 4 μM inhibitor (JMS-175-2, FX-171-C or FX-171-A), 4 μM negative control (AP-5-145), DMSO control (0.1%) or JAK–STAT inhibitor tofacitinib (0.4 μM) for 16 h. IFNAR1 surface levels were quantified using FACS analysis and the median fluorescence intensity of allophycocyanine (APC)–IFNAR1 was calculated as a percentage of no IFN stimulation. Data points are from three independent experiments. Statistical analyses in **a** were performed using paired, two-tailed *t*-tests to compare compound-treated cells to DMSO control cells. In **b**–**g**, a one-way ANOVA with Dunnett’s multiple-comparisons test was used to compare statistical significance between AP-5-145 and BLUE compound treatment. In **h**, unpaired, two-tailed *t*-tests were used to compare compound-treated cells to DMSO control cells. Error bars represent ±s.e.m. Source data

We first evaluated the impact of BLUE treatment on the activation of IFN-stimulated response elements (ISRE) in response to IFNα2 stimulation. Both JMS-175-2 and FX-171-C reduced ISRE relative expression compared to the AP-5-145 and FX-171-A controls, with tofacitinib having a potent effect (Fig. 5a).

Next, we stimulated THP-1 cells with different agonists to determine whether BLUE treatment affected other signaling pathways. We stimulated THP-1 cells with TLR3 and TLR9 agonists, polyinosinic:polycytidylic acid (polyI:C) and ODN 2216. These treatments only minimally induced ISRE relative expression, which was not reduced by BLUE or tofacitinib treatment (Extended Data Fig. 8d,e). In addition, we used a nuclear factor (NF)-κB pathway reporter assay and observed no BLUE effect on lipopolysaccharide-induced NF-κB pathway activity compared to the control compounds (Extended Data Fig. 8f).

To probe the effect of BLUE compounds in cells, we generated BRCC45 knockout (KO) MCF10A cells using CRISPR–Cas9-mediated genomic deletion (Extended Data Fig. 8g) and complemented BRCC45 KO with either Flag–BRCC45 WT or Flag–BRCC45 R137A (Extended Data Fig. 8h). In DUB activity assays, the BRCC45 R137A substitution reduced FX-171-C inhibition (IC_50_ > 100 µM) without affecting SHMT2 inhibition (Fig. 4c,d and Extended Data Fig. 7c). We confirmed by coimmunoprecipitation that interactions with BRISC subunits BRCC36 and MERIT40 were maintained in the BRCC45 WT and BRCC45 R137A cell lines (Extended Data Fig. 8i).

MCF10A cells were challenged with IFNα2 to stimulate IFNAR1 signaling and ISG expression. An increase in STAT1 phosphorylation was observed in the single guide (sg)ROSA (control sgRNA) cells, BRCC45 WT and BRCC45 R137A cells and less STAT1 phosphorylation was observed in BRCC45 KO cells (Extended Data Fig. 8j). Following IFNα2 stimulation, we used reverse transcription (RT)–qPCR to measure changes in gene expression for five ISGs: *ISG15*, *IFIT1*, *IFIT2*, *IFITM1* and *CXCL10*. The differences in gene expression across sgROSA, WT and R137A cell lines were nonsignificant, except for *ISG15*, which had higher gene expression in BRCC45 WT cells but still comparable to BRCC45 R137A (Extended Data Fig. 8k).

To measure the effect of BLUEs on ISG expression, we compared BRCC45 WT and BRCC45 R137A cells with active and inactive control compounds. Treatment of BRCC45 WT cells with 2.5 μM JMS-175-2 and FX-171-C reduced gene expression for all five ISGs (Fig. 5b–f). Critically, a reduction in ISG expression was not observed in cells harboring the BRCC45 R137A substitution, which reduces BLUE inhibition of BRISC, indicating on-target BLUE activity in cells.

BRISC regulates IFN signaling through IFNAR1 deubiquitylation, likely limiting IFNAR1 internalization and subsequent degradation^22^. We used fluorescence-activated cell sorting (FACS) to determine the effect of BLUE compounds on IFNAR1 surface levels. MCF10A cells were challenged with IFNα2, which resulted in reduced IFNAR1 surface levels, indicating increased IFNAR1 internalization in response to IFN stimulation (Extended Data Fig. 8l). We treated the sgROSA and BRCC45 WT cells with IFNα2 and the compound panel and observed a reduction in IFNAR1 cell surface levels with FX-171-C and JMS-175-2 but not with FX-171-A and AP-5-145 (Fig. 5g and Extended Data Fig. 8m). Importantly, IFNAR1 surface levels were not reduced in the BRCC45 R137A cells, indicating that reduced IFNAR1 levels in sgROSA and BRCC45 WT cells were because of BRISC inhibition (Fig. 5g and Extended Data Fig. 8m). Similarly, we stimulated the monocyte cell line THP-1 with IFNα2 treated with the inhibitor panel and analyzed IFNAR1 levels using FACS. We observed a reduction in IFNAR1 surface levels after treatment with JMS-175-2 and FX-171-C, whereas no significant reduction was observed with AP-5-145, FX-171-A or JAK–STAT inhibitor tofacitinib (Fig. 4h).

To evaluate the impact of BLUE compound treatment and BRISC inhibition on IFNAR1 ubiquitylation, we used tandem ubiquitin-binding entities (TUBEs) and western blotting. Following stimulation with IFNα2 in MCF10A cells, we isolated ubiquitylated IFNAR1 using TUBE pulldowns. We observed elevated levels of IFNAR1 ubiquitylation with FX-171-C and JMS-175-2 treatments compared to treatment with DMSO, AP-5-145 and FX-171-A (Extended Data Fig. 8n–p).

**Fig. 6:**
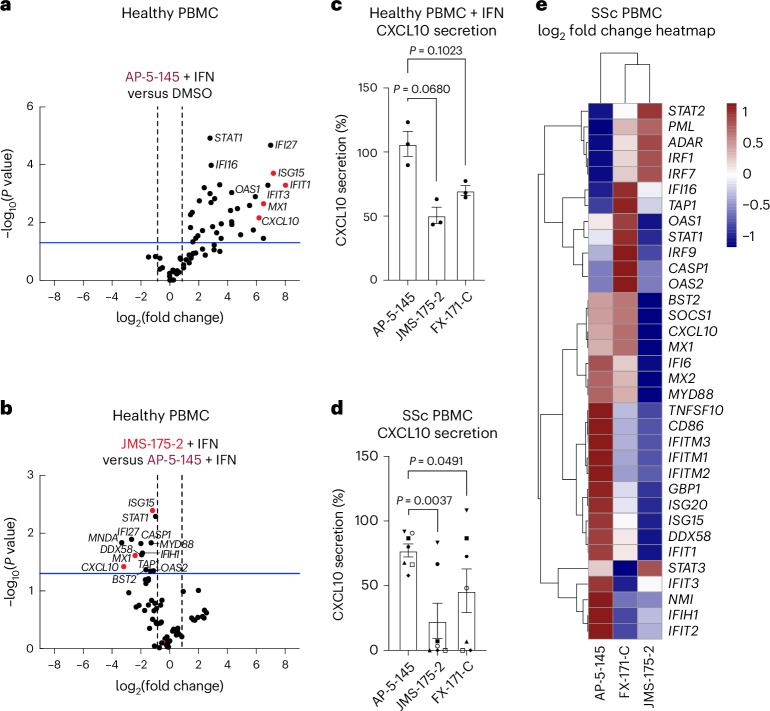
BLUE compounds reduce ISG expression in PBMCs. **a**,**b**, Type I IFN signaling gene expression analysis (67 genes normalized for housekeeping genes: *ACTB*, *GAPDH*, *HPRT1* and *RPLP0*) of healthy control PBMCs stimulated with IFNα2. Volcano plot of genes increased upon addition of IFNα2 with negative control AP-5-145 compared to DMSO (no IFN) condition (**a**). Effect of JMS-175-2 + IFN stimulation compared to AP-5-145 + IFN (**b**). The blue line indicates *a P* value of 0.05. Data points are the means from three independent experiments. **c**, CXCL10 protein levels in supernatant from IFNα2-stimulated healthy PBMCs (*n* = 3) quantified by ELISA and shown as a percentage of a control treated with IFN + DMSO (100%). The bar graph shows the average of three independent experiments. **d**, PBMCs were isolated from participants with SSc and treated with DMSO, AP-5-145, FX-171-C or JMS-175-2 for 16 h without IFN stimulation. Secreted CXCL10 in supernatant is shown as a percentage of the DMSO control. The bar graph shows the average of data from seven SSc donors. **e**, Type I IFN signaling gene expression analysis of unstimulated SSc PBMCs, treated with 2 µM AP-5-145, JMS-175-2 or FX-171-C. Heat map showing log_2_ mean fold change in ISG expression by treatment, compared to AP-5-145, according to qPCR SuperArray. Δ*C*_*t*_ was calculated against the geometric mean of four housekeeping genes, followed by the ΔΔ*C*_*t*_ (fold change) relative to AP-5-145 and log_2_ transformation. Heat maps represent the mean fold change from nine SSc donors. *P* values were calculated using a Student’s *t*-test (two-tailed distribution and equal variances between the two samples). In **a**–**d**, paired, two-tailed Student’s *t*-tests were used to compare between treatment conditions for statistical significance. Error bars represent ±s.e.m. Source data

**Extended Data Fig. 9:**
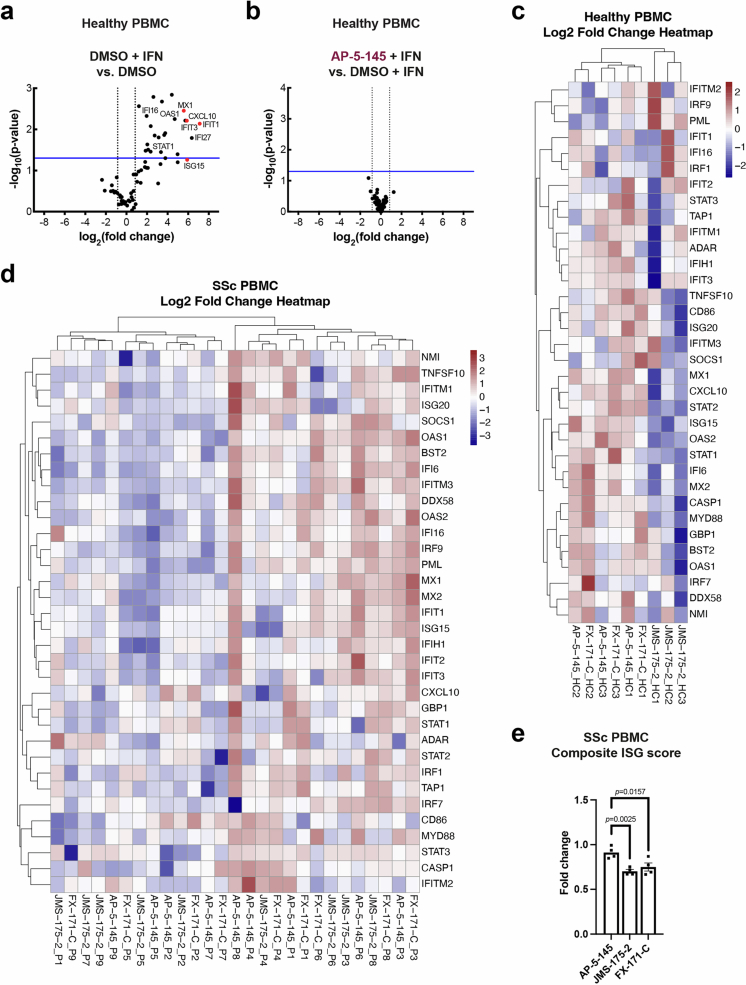
BLUE compounds reduce interferon-stimulated gene expression in stimulated healthy and unstimulated SSc PBMCs. **a**–**c**, Type I IFN signalling gene expression analysis of healthy control PBMCs treated with/without IFNα2 (20 ng/mL) and DMSO control (0.1 %) or 2 µM AP-5-145, JMS-175-2, or FX-171-C for 16 h (n = 3). **a**, Volcano plot illustrating genes increased with addition of IFN + DMSO vs. DMSO only. **b**, Volcano plot illustrating no change in gene expression with negative control AP-5-145 + IFN vs. DMSO + IFN only. In **a**, and **b**, data points are the means from three independent experiments. *P* values were calculated using a Student’s *t*-test (two-tailed distribution and equal variances between the two samples). **c**, Heatmap of each ISG expression levels relative to each donors housekeeping gene expression levels (geometric mean of *ACTB*, *GAPDH*, *HPRT1*, *RPLP0*), shown as Log2 fold change to grouped AP-5-145. Data shown for each individual donor, HC = healthy control. Heat map represents the mean fold change from three healthy donors. **d**, Type I IFN signalling gene expression analysis of unstimulated SSc PBMCs from nine patients, treated with 2 µM AP-5-145, JMS-175-2, or FX-171-C for 16 h. ISG relative expression to each donors housekeeping genes, shown as Log2 fold change relative to grouped AP-5-145, as in **a**. P refers to patient number that is P1 = patient 1. Heat map represents the mean fold change from nine SSc donors. **e**, PBMCs were isolated from patients and treated with DMSO (0.1%), 2 µM AP-5-145, FX-171-C or JMS-175-2 for 16 h without IFN stimulation. Composite ISG score (including *CXCL10*, *IFIT1*, *ISG15* and *MX1*) gene expression analysis between conditions relative to each donor DMSO control. Error bars represent ± SEM. Individual data points represent the mean fold change for each gene for 20 donors. Statistical analysis was performed using a paired, two-tailed *t*-test. Source data

We next studied the effects of BLUE compound treatment in peripheral blood mononuclear cells (PBMCs) from healthy volunteers upon IFNα stimulation. For a comprehensive analysis of how BLUE compounds modulate the IFN gene signature, we measured the expression of 67 ISGs, including *ISG15*, *IFIT1*, *IFIT2*, *IFITM1*, *CXCL10* and *MX1*. A total of 34 ISGs were significantly upregulated by IFN, with no difference in ISG expression profile between DMSO and AP-5-145 (Fig. 6a and Extended Data Fig. 9a,b). Treatment with JMS-175-2 suppressed the IFN-induced signature compared to AP-5-145 in 13 of the 34 ISGs (Fig. 6b and Extended Data Fig. 9c). Interestingly, for FX-171-C-treated PBMCs, there was less reduction in ISG expression compared to the AP-5-145 control when used at 2 μM (Extended Data Fig. 9c). We also measured IFN-inducible CXCL10 secretion levels and observed lower CXCL10 protein secretion in BLUE-treated PBMCs (Fig. 6c).

To assess the activity of BLUE compounds in the context of autoimmune disease associated with aberrant type I IFN activation, we also measured ISG expression and CXCL10 secretion levels of PBMCs from participants affected by SSc^25^ (Supplementary Table 2). Treatment of basal, nonstimulated SSc PBMCs with JMS-175-2 or FX-171-C reduced CXCL10 secretion (Fig. 6d). Next, we used RT–qPCR to analyze the effect of BLUE compound treatment on 34 upregulated ISGs (identified in healthy PBMCs) (Extended Data Fig. 9a). In SSc PBMCs (*n* = 9), 22 ISGs were reduced after treatment with JMS-175-2 compared to AP-5-145, with FX-171-C having a lesser effect (Fig. 6e and Extended Data Fig. 9d). Additionally, we used a composite ISG score including *CXCL10*, *IFIT1*, *ISG15* and *MX1* relative to *GAPDH* (ref. ^48^) to measure the effect on ISG expression for a larger group of participants (*n* = 20). BLUE treatment of unstimulated SSc PBMCs reduced the composite ISG score for JMS-175-2 and FX-171-C relative to AP-5-145 (Extended Data Fig. 9e).

## Discussion

Deubiquitylating enzymes are prime therapeutic targets because of their roles in cancer, neurodegeneration, inflammation and immunity^10,49–51^. The BRISC DUB complex, which regulates type I IFN signaling, is a key candidate for ameliorating autoimmune conditions^23–25^. However, high conservation among JAMM/MPN DUB active sites complicates selective inhibitor discovery. Moreover, the same catalytic subunit (BRCC36) exists in two complexes with distinct functions. Through biochemical screening and structural and molecular biology approaches, we identified first-in-class, selective inhibitors of the cytoplasmic BRISC DUB complex over related DUBs.

BRISC inhibitors act as MGs that stabilize an inhibited BRISC dimer. We verified target engagement in cells using an inhibitor-resistant BRCC45 R137A mutant. BLUE compounds lowered ISG expression in MCF10A cells, THP-1 cells and PBMCs from healthy participants and those with SSc. Although JMS-175-2 reduced ISGs on average (Fig. 6e), participant variability masked individual effects (Extended Data Fig. 9d). A key finding is that BLUEs reduce IFN receptor surface levels rather than directly blocking IFN signaling, thus providing an alternative to JAK inhibition for reducing inflammatory signaling. The modest inhibition compared to clinical JAK inhibitors reflects current compound potency, which could be improved. Nonetheless, partial pathway inhibition could be exploited to normalize rather than fully suppress type I IFN activity in disease.

**Fig. 7:**
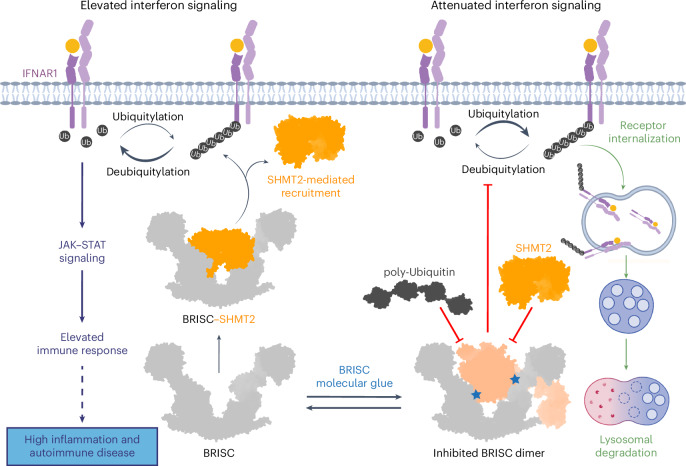
Proposed model of BLUE compound mode of action. Interferon binding to IFNAR1 receptors triggers JAK–STAT signaling and an elevated immune response. IFN also initiates IFNAR1 receptor ubiquitylation (K63-linked), receptor internalization and lysosomal degradation. The BRISC–SHMT2 complex is required for deubiquitylation of IFNAR1. BRISC is recruited to IFNAR1 and IFNAR2 through interactions with SHMT2 to promote sustained IFN signaling and inflammation. BLUE compounds (blue stars) promote the formation of a BRISC dimer complex, which sterically hinders SHMT2 and polyubiquitin binding. Some graphics in this image were generated with BioRender.com.

On the basis of reports that IFNAR1 is a BRISC substrate^22,44^, we propose that reduced BRISC activity causes hyperubiquitylation of IFNAR1, accelerating receptor degradation and lowering inflammatory signaling (Fig. 7). By combining structural analyses of BRISC–SHMT2 and BRISC–ubiquitin models, we further suggest that BLUEs stabilize an autoinhibited BRISC dimer.

DUBs are regulated by autoinhibited conformations, protein–protein interactions and pseudo-DUB partners. The DUB MG described herein offers a strategy to selectively inhibit DUBs or other macromolecular complexes by promoting interactions rather than disrupting them. Such glues stabilize autoinhibited conformations, leveraging natural regulatory mechanisms, as is the case for NX-1607, an intra-MG that traps Cbl-b in an inactive state^52,53^. Therefore, this approach enables the identification of selective inhibitors for DUBs with conserved active sites that function as dimers (for example, USP25 and USP28)^54,55^.

Several USP inhibitors block catalysis by stabilizing an inactive enzyme conformation. For example, USP7 inhibitors FT671 and FT827 (ref. ^27^) and the cross-reactive USP11–USP15 inhibitor mitoxantrone^56,57^ misalign active-site residues. Other USP7 inhibitors (GNE-6640 and GNE-6676) block ubiquitin binding rather than disrupting the catalytic site^28^. Likewise, BLUEs inhibit BRCC36 without engaging its catalytic site, whereas other JAMM/MPN inhibitors described to date are presumed to target the catalytic zinc (Extended Data Fig. 5d,e).

BLUE compounds gain selectivity by binding a three-protein interface (Fig. 3c). Most MGs contact two proteins, although CsA and FK506 stabilize interactions among three proteins to disrupt calcineurin signaling^37^. Both MGs contact calcineurin A and B; CsA also engages cyclophilin and FK506 engages FK-binding protein^37,58,59^. BLUEs similarly interact with a DUB (BRCC36), a pseudo-DUB (Abraxas 2) and a UEV domain (BRCC45), targeting a previously unexplored UEV site.

Many DUBs rely on protein–protein interactions for enzymatic regulation, localization and substrate recognition. BRCC36 subcellular function depends on pseudo-DUB partners Abraxas 1 and Abraxas 2 (ref. ^16^). We show that BRISC activity is inhibited through higher-order dimerization. The presence of BRISC dimers without small molecules suggests that MGs may stabilize a low-affinity BRISC–BRISC interaction. Unlike bifunctional proteolysis-targeting chimeras^60^, MGs enhance existing low-affinity binding and this is true for BRISC dimer stabilization. The asymmetric dimer occludes the BRCC36 active site and predicted polyubiquitin-binding regions (Extended Data Fig. 3d). Moreover, the BLUE-binding pocket overlaps with the binding interface of the endogenous inhibitor SHMT2 (Extended Data Fig. 7e). This dimer likely represents an autoinhibited BRISC state sampled at low levels in unstimulated cells. Elucidating the functional relevance and regulation of these BRISC dimers is a key area for future study.

Testing BLUE inhibition of BRISC orthologs revealed a human-specific BRCC36 loop critical for inhibitor sensitivity and dimer formation. A human splice isoform lacking this loop (residues 184–208) and cryo-EM maps showed that this flexible loop extends toward the opposite BRISC molecule, stabilizing the dimer (Extended Data Fig. 6i). These findings suggest that an autoinhibited state is more prominent in human BRISC. Further studies with more potent, drug-like inhibitors will clarify BRISC dimer regulation in cells and animals. Differences between mouse and human forms require careful consideration in murine models and humanized mouse models may be necessary to evaluate BRISC inhibitors in vivo.

## Methods

Our research complies with all relevant ethical regulations. All participants enrolled provided written informed consent according to a protocol approved by the Medicine and Health Regulatory agency (STRIKE NRES-011NE to F.D.G., IRAS 15/NE/0211; reviewing body: North East—Newcastle and North Tyneside 2 Research Ethics Committee; sponsor: University of Leeds).

### Expression and purification of DUB complexes

Genes encoding the four-subunit human BRISC and ARISC complexes (full-length BRISC (FL), ARISC (FL), BRISCΔNΔC (MERIT40ΔN, Abraxas 2ΔC), BRCC36–Abraxas 2, BRISCΔLoop (Δ184–208), *Dr*BRISCΔNΔC and *Cf*BRISCΔNΔC) were cloned using the MultiBac system and coexpressed in *Spodoptera frugiperda* (*Sf*9) insect cells^61^. Genes encoding BRISC mutants and *Mm*BRISC were cloned into pFastBac-HTB vectors in the Bac-to-Bac system (Thermo Fisher Scientific), baculoviruses were generated in *Sf*9 cells and used for coinfection of *Trichoplusia ni* cells. All BRISC complexes were purified as previously described^18,44^.

Plasmids encoding N-terminally His-tagged USP2 (plasmid 36894) and N-terminally His-tagged AMSH* (pOPINB-AMSH*, plasmid 66712)^43^ were purchased from Addgene. SHMT2ΔN(A285T) (residues 18–504) was expressed and purified as previously described^44^. His–USP2 and His–AMSH* were expressed in *Escherichia coli* BL21(DE3) cells. Cells were grown at 37 °C in Terrific Broth medium, induced with 0.5 mM IPTG and grown overnight at 18 °C. For purification, cell pellets were resuspended in lysis buffer containing 50 mM Tris-HCl pH 7.6, 300 mM NaCl, 20 mM imidazole, 5% glycerol, 0.075% β-mercaptoethanol, 1 mM benzamidine, 0.8 mM PMSF and 0.3 mg ml^−1^ lysozyme. Cells were lysed by sonication (1 s on, 1 s off for a total of 16 min) and cleared by centrifugation at 18,000*g*. The clarified lysate was incubated with Ni-NTA beads (Cytiva) for 1 h at 4 °C, before washing with wash buffer containing 50 mM Tris-HCl pH 7.6, 300 mM NaCl, 20 mM imidazole, 5% glycerol, 0.075% β-mercaptoethanol and 1 mM benzamidine and a high-salt buffer containing 500 mM NaCl. The protein was eluted with elution buffer containing 50 mM Tris-HCl pH 7.6, 300 mM NaCl, 120 mM imidazole, 5% glycerol, 0.075% β-mercaptoethanol and 1 mM benzamidine. The elutions containing His–USP2 or His–AMSH* were dialyzed overnight with thrombin (His–USP2) or 3C PreScission protease (His–AMSH*). After dialysis, cleaved samples were incubated with Ni-NTA beads and washed with wash buffer. The cleaved fractions were concentrated and loaded onto a Superdex 75 10/300 column (Cytiva) equilibrated with 25 mM HEPES pH 7.5, 150 mM NaCl and 1 mM TCEP.

### Compound library screening

BRISC DUB activity was measured at room temperature using 1 nM BRISC and 500 nM IQF diubiquitin K63 substrate (LifeSensors, DU6303) in the presence of DMSO and compounds at 10 μM (final concentrations). Assays were performed in 384-well black flat-bottom low-flange plates (Corning, 35373) in buffer containing 50 mM HEPES–NaOH pH 7.0, 100 mM NaCl, 1 mg ml^−1^ BSA, 1 mM DTT and 0.03% v/v Brij-35. Then, 10 μl of twofold concentrated enzyme stock was dispensed, followed by transfer of 200 nl of compounds (1 mM stock) using a 384-pin tool and a 15-min incubation at room temperature. Then, 10 μl of twofold concentrated substrate stock was added and the reaction was monitored by measuring fluorescence intensity (excitation, 540 nm; emission, 580 nm) after 20-min incubation at room temperature and ~50% of substrate was consumed. Orthogonal assays for verification of H20 and P12 hit compounds using USP2 (100 nM) and trypsin (125 nM) were performed in identical conditions.

### LC–MS and MS/MS analysis

LC–MS/MS analysis was carried out in an ultrahigh-performance (UPLC) BEH C18 (2.1 × 50 mm, 1.7 µm) column using the Acquity UPLC II system. The mobile phase was 0.1% formic acid in water (solvent A) and 0.1% formic acid in acetonitrile (solvent B). A gradient starting at 95% solvent A going to 5% in 4.5 min, holding for 0.5 min, going back to 95% in 0.5 min and equilibrating the column for 1 min was used. A Waters Synapt G2S quadrupole time-of-flight MS instrument equipped with an electrospray ionization source was used for MS analysis. MassLynx (version 4.1) was used for data analysis. The parameters for LC–MS analysis were as follows: frequency, 15 s; cone voltage, 25 V; capillary voltage, 3 kV. For MS/MS spectra, an MS/MS range of *m*/*z* 50–900, scan time of 0.1 s and collision energy ramp of 30–60 V were used. Compound identifications were performed using accurate mass analysis and MS/MS fragmentation analysis. Absorbance spectra were measured using a UV–vis Nanodrop spectrophotometer N8000 (Thermo Fisher Scientific).

### Synthesis of BRISC inhibitors

Reaction schemes, methods and validation of the synthesis of JMS-175-2, FX-171-C, AP-5-145,and FX-171-A are outlined in Supplementary Protocol 2.

### DUB activity assays (IQF)

IQF assays were performed in DUB reaction buffer containing 50 mM HEPES–NaOH pH 7.0, 100 mM NaCl, 0.1 mg ml^−1^ BSA, 1 mM DTT and 0.03% v/v Brij-35. To assess inhibitor potency, inhibitors were diluted in DMSO up to a final concentration of 200 μM and incubated with the target enzyme for 15 min at room temperature. The final concentration of BRISC WT, all BRISC mutants, *Hs*BRISCΔLoop, *Mm*BRISC and *Dr*BRISCΔNΔC was 1 nM. Other enzymes were tested at the following concentrations: 5 nM ARISC (FL), 10 nM *Cf*BRISCΔNΔC, 250 nM AMSH* (ref. ^43^), 500 nM USP2 and 1 μM trypsin (Sigma, 9002-07-7). For Michaelis–Menten experiments, 1 nM BRISC with 0–1 μM IQF K63-linked diubiquitin substrate was used. Michaelis–Menten analyses and Lineweaver–Burk plots were calculated using GraphPad Prism (version 9.0).

To assess enzymatic activity, BRISC complexes were diluted to concentrations between 1 nM and 50 nM. For SHMT2 assays, SHMT2(A285T) was diluted in 20 mM MES pH 6.5, 500 mM NaCl and 2 mM TCEP up to 200 nM. SHMT2 was incubated with enzyme for 15 min at room temperature before adding substrate. Then, 20-μl enzyme reactions were carried out in 384-well black flat-bottom low-flange plates (Corning, 35373). DUB activity was measured using IQF K63-linked diubiquitin (LifeSensors, DU6303) at 50 nM. Cleaved diubiquitin was monitored by measuring fluorescence intensity (excitation, 544 nm; emission, 575 nm; dichroic mirror, 560 nm). Fluorescence intensity was measured every minute for 30 min at 30 °C. Fluorescence intensity units were plotted against time to generate a linear reaction progress curve, where the initial velocity (*V*_0_) corresponds to the gradient of the curve (typically achieved within 15 min). IC_50_ values were calculated using the GraphPad Prism (version 9.0) built-in dose–response equation for inhibitor concentration versus response (variable slope).

### DUB selectivity profiling

Biochemical selectivity profiling (DUBprofiler) was performed by Ubiquigent with a panel of 48 purified DUBs and ubiquitin–rhodamine(110)–glycine as a fluorescent substrate. Single-dose inhibition (5 μM) was determined for JMS-175-2 after a 15-min preincubation at room temperature.

### Deubiquitylation assay using fluorescently labeled polyubiquitin chains

The inhibitor capzimin (Merck) was diluted in 1 mM DTT and incubated for 30 min at room temperature to reduce disulfide bonds required for inhibitor activity. Then, 5 nM BRISC (FL) or 20 nM ARISC (FL) was incubated with 0.5% DMSO or 10 μM or 100 μM inhibitor (FX-171-C, JMS-175-2, AP-5-144, thiolutin or capzimin) for 15 min at room temperature in DUB reaction buffer containing 50 mM HEPES–NaOH pH 7.0, 100 mM NaCl, 0.1 mg ml^−1^ BSA, 1 mM DTT and 0.03% v/v Brij-35. DUBs were incubated with 750 nM TAMRA-labeled K63-tetraubiquitin (LifeSensors, SI6304T) for 10 min at 30 °C. Reactions were quenched by adding 2.5 μl of 4× SDS–PAGE loading dye (240 mM Tris-HCl pH 6.8, 40% v/v glycerol, 8% w/v SDS, 0.04% w/v bromophenol blue and 5% v/v β-mercaptoethanol). The samples were resolved on 4–12% NuPAGE Bis–Tris gels (Thermo Fisher Scientific). Gels were scanned using an iBright FL 1500 (Thermo Fisher Scientific) (excitation, 515–545 nm; emission, 568–617 nm; exposure, 500 ms).

### Mass photometry

For single-point measurement of BRISC and inhibitors, 1 μM BRISC (FL) was mixed with DMSO, JMS-175-2 or FX-171-C at 330 μM and incubated for 15 min on ice. Immediately before mass photometry measurement, the BRISC–inhibitor mix was diluted in 25 mM HEPES pH 7.5, 150 mM NaCl and 1 mM TCEP to 10 nM BRISC (0.05% v/v DMSO). Then, 12 μl of the diluted sample was used for the final mass photometry measurement, following autofocus stabilization. For measurements with increasing concentrations of JMS-175-2 and FX-171-C, twofold dilutions of inhibitor in 100% v/v DMSO generated a dilution series with concentrations of inhibitor from 800 μM to 0 μM. Next, 0.5 μl of inhibitor was mixed with 19.5 μl of 50 nM BRISCΔNΔC (2.5% v/v DMSO) and incubated at room temperature for 15 min. The BRISC–inhibitor mix was used directly for mass photometry measurement using buffer-free autofocus stabilization.

Microscope coverslips were prepared according to an established protocol^62^. Experiments were performed using a OneMP mass photometer (Refeyn). Videos were recorded for 60 s using AcquireMP (Refeyn) and processed using DiscoverMP (Refeyn). Mass photometry image processing was previously described^62^. Briefly, contrast-to-mass (C2M) calibration was performed using protein standards (66–669 kDa) diluted in buffer (25 mM HEPES pH 7.5, 150 mM NaCl and 1 mM TCEP). The output from each individual video resulted in a list of particle contrasts that were converted to mass using the C2M calibration. The mass distribution from each run was in the form of a histogram, where the count refers to each landing event and a Gaussian sum is fitted to the data. The relative amount of each species was calculated as the area of each Gaussian, where *σ* refers to the s.d. of the fitted Gaussian. The dimer fraction refers to the percentage of the total counts corresponding to the 564-kDa BRISCΔNΔC dimer complex. Curves and half-maximal effective concentration values were fitted and calculated using the GraphPad Prism (version 9.0) built-in dose–response equation for concentration of agonist versus response (variable slope).

### Negative-stain EM

BRISC (FL) or *Mm*BRISC (FL) was mixed with inhibitor (or DMSO) on ice for 30 min (1 μM BRISC and 100 μM inhibitor). The BRISC–inhibitor mix was diluted in gel filtration buffer to 0.014 mg ml^−1^ (0.5% v/v DMSO). Carbon-coated copper grids (formvar–carbon 300-mesh Cu, Agar Scientific) were glow-discharged for 30 s at 10 mA and 0.39 mbar (PELCO easiGlow, Ted Pella). Grids were incubated for 1 min with 7 μl of sample, washed three times with H_2_O and stained twice with 2% w/v uranyl acetate for 30 s. Excess liquid was removed by blotting with filter paper. Data were collected using an FEI Tecnai F20 microscope (Thermo Fisher Scientific) at 200 keV, fitted with an FEI CETA (complementary metal–oxide–semiconductor charge-coupled device) camera. Micrographs were collected at ×29,000 magnification with a pixel size of 3.51 Å. RELION (versions 3.0 and 3.1.1) were used for processing of negative-stain EM data^63,64^. Approximately 2,000 particles were manually picked and extracted with a box size of 128 Å^2^. These particles were used for reference-free 2D class averaging to generate 2D templates for autopicking. The parameters for autopicking were optimized and 5,000–10,000 particles were extracted. Two rounds of 2D classification were used to remove junk particles and assess the stoichiometry of the BRISC complex.

### Cryo-EM grid preparation and data collection

For the BRISC only grid, Quantifoil R1.2/1.3 300-mesh copper grids were glow-discharged using a PELCO easiGlow glow discharge system (TedPella). Then, 3 μl of BRISC (FL) at 0.7 mg ml^−1^ in gel filtration buffer was loaded onto the cryo-EM grids. For the BRISC–JMS-175-2 grid, BRISCΔNΔC at 0.3 mg ml^−1^ (2 μM) was mixed with 200 μM JMS-175-2 for 30 min on ice. Quantifoil R1.2/1.3 300-mesh copper grids were glow-discharged using a GloQube (Quorum) for 30 s at 40 mA. For the BRISC–FX-171-C grid, BRISCΔNΔC at 0.7 mg ml^−1^ (5 μM) was mixed with FX-171-C at 400 μM in gel filtration buffer and loaded onto grids that were plasma-cleaned in downstream mode at a radiofrequency power of 43 W for 30 s using a Tergeo plasma cleaner (Pie Scientific). For all grids described in this manuscript, an FEI Vitrobot IV (Thermo Fisher Scientific) was equilibrated to 4 °C at 100% relative humidity. Grids were blotted at blot force 3 for 4 s and plunged into liquid ethane cooled by liquid nitrogen for vitrification.

Videos were collected on a Titan Krios G2 transmission EM instrument (Thermo Fisher Scientific) at 300 keV fitted with an FEI Falcon 4 direct electron detector (Thermo Fisher Scientific). For the BRISC (FL) and BRISC–FX-171-C, data were collected with a 10-eV Selectris energy filter (Thermo Fisher Scientific) using EPU automated acquisition software (version 3.5.1) in counting mode. More detailed data acquisition parameters are in Table 1.

### Image processing

Motion correction and contrast transfer function (CTF) estimation were performed on the fly^65^ in RELION (versions 3.0 and 3.1.1)^63,64^. Motion correction was performed using RELION’s own implementation of the MotionCor2 algorithm^66^. CTF was estimated using CTFFIND (version 4.1.14)^67^ for the BRISC (FL) and BRISC–FX-171-C datasets and using gCTF (version 1.18)^68^ for the BRISC–JMS-175-2 dataset. Extended Data Figure 2e–g outlines the data processing pipeline for the BRISC (FL) dataset. Particles were picked using crYOLO (version 1.6.1)^69^ using a model trained on 16 micrographs. Particle coordinates were imported into RELION (version 3.1.1) and 1,933,988 particles were extracted with a box size of 480 pixels. The particle stack was imported into cryoSPARC (version 4.2.1)^70,71^ and subjected to two rounds of reference-free 2D classification. A total of 148,596 particles were selected from high-quality 2D class averages for ab initio reconstruction. Four classes corresponded to a BRISC monomer and were selected for heterogeneous refinement. The best class (34% of the particles) was further refined using nonuniform refinement and global CTF refinement with beam tilt and beam trefoil fitted. To generate a map for the BRISC dimer complex, the class with additional density from ab initio reconstruction (32,283 particles) was refined using homogeneous refinement followed by nonuniform refinement and global CTF refinement with beam tilt and beam trefoil fitted. The same particles were also refined with *C*_2_ symmetry applied during nonuniform refinement. The final map resolutions were determined using the gold-standard Fourier shell correlation (FSC) criterion (FSC = 0.143) with FSC curves generated using the PDBe FSC server (EM Data Bank). Local resolutions were determined using the local resolution implementation in cryoSPARC and visualized in ChimeraX (version 1.2.3)^72^. To visualize the Euler angular distribution, the csparc2star.py and star2bild.py pyem scripts were used^73^. To model BRISC in the monomer and dimer conformations, one or two BRISC models with SHMT2 removed (Protein Data Bank (PDB) 6H3C) were rigid-body fitted using Chimera (version 1.12)^74^ and visualized using ChimeraX (version 1.2.3).

Data processing for the BRISCΔNΔC–FX-171-C dataset is outlined in Extended Data Fig. 4b. A crYOLO (version 1.6.1)^69^ model trained on particles picked from ten micrographs was used to pick 2,458,785 particles that were extracted with a box size of 192 pixels and a binning factor of 2 using RELION (version 3.1.1). Particles were subjected to one round of reference-free 2D classification. A BRISC–JMS-175-2 map was low-pass filtered and used as an initial model for 3D classification with no symmetry applied. The two best classes (632,988 particles) were selected for 3D refinement, postprocessing and three rounds of Bayesian particle polishing and CTF refinement, resulting in a final map at 3.02 Å. To improve the density around the small-molecule-binding site, a mask was applied during refinement to one half of the map (Extended Data Fig. 4g). The map resolution improved to 2.8 Å overall and 2.7 Å around the BLUE-binding site.

A schematic (Extended Data Fig. 4e) details the data processing pipeline for the BRISCΔNΔC–JMS-175-2 dataset. Particle picking was performed using crYOLO (version 1.6.1)^69^. A model was trained from manually picking 14 micrographs. The trained model picked 1,616,457 particles. Coordinates were imported into RELION (version 3.1.1) for extraction with a box size of 176 pixels and a binning factor of 2. Two rounds of reference-free 2D classification were used to remove junk particles. A reference model from a previous BRISC–JMS-175-2 dataset was applied during 3D classification of 1,011,924 particles with no symmetry applied. Three classes (371,872 particles) were re-extracted with a box size of 352 pixels. Then, 3D refinement, postprocessing and iterative rounds of per-particle CTF refinement and Bayesian polishing resulted in a 3.32-Å final map resolution. To improve the density around the small-molecule-binding site, a mask was applied during 3D refinement, encompassing only the better-resolved half of the map (Extended Data Fig. 4i). This improved the density for ‘half’ of the structure, resulting in a 3.2-Å map. Final resolutions were determined using the gold-standard FSC criterion (FSC = 0.143). Local resolution estimation was carried out using the RELION local resolution feature.

### Model building and refinement

Atomic models of the BRISC dimer in complex with either JMS-175-2 or FX-171-C were built using high-resolution cryo-EM maps. A preliminary model of the human BRCC36–Abraxas 2 super dimer was acquired from our previous BRISC–SHMT2 model (PDB 6R8F)^44^ with BRCC45 and SHMT2 removed. The BRCC36–Abraxas 2 super dimer was rigid-body fitted into the cryo-EM density using UCSF Chimera^74^ and manually modeled into the BRISC–FX-171-C map using Coot^75,76^. The super dimer was duplicated, rigid-body fitted and manually modeled into the BRCC36–Abraxas 2 in the opposite side of the map. A model for human BRCC45 and MERIT40 was acquired from a previous BRISC–SHMT2 model (PDB 6H3C)^20^ and rigid-body fitted into the cryo-EM density. The BRCC45 N termini (residues 1–275) were manually modeled using Coot; however, because of the lower resolution of the map beyond the UEV-M domain and for MERIT40, these regions were rigid-body fitted into the density on the basis of previous BRISC–SHMT2 structures^20,44^. The BRCC45–MERIT40 arms were duplicated, rigid-body fitted and modeled into the density corresponding to the second BRISC molecule. The side-chain atoms for BRCC45 (residues 275–383) and MERIT40 were set to zero occupancy because of lower resolution of the EM maps in these regions. Small-molecule chemical structures were generated in ChemDraw (PerkinElmer) and PDB and CIF files were created using the PRODRG2 server^77^ or eLBOW^78^. FX-171-C compounds were manually fitted into the density using Chimera and refined using Coot real-space refinement. The model was refined against the BRISC–FX-171-C map using Phenix real-space refinement (version 1.20)^79^. To build the BRISC–JMS-175-2 structure, FX-171-C was removed from the model and replaced with JMS-175-2. The model was rigid-body fitted into the density for a BRISC–JMS-175-2 cryo-EM map and subjected to iterative rounds of manual building in Coot. The BRISC–JMS-175-2 model was refined using Phenix real-space refinement (version 1.20).

### Sequence alignments and structure visualization

Multiple-sequence alignments were performed using MUSCLE^80^ and edited using ALINE (version 1.0.025)^81^. EM maps and structure models were visualized in UCSF Chimera (version 1.12.0)^74^ and ChimeraX (version 1.2.3)^72^.

### Native MS

BRISC (FL) at 10 μM was mixed with 1 mM inhibitor (JMS-175-2 or FX-171-C) or DMSO (2.5%) and incubated on ice for 30 min. Samples were buffer-exchanged into 500 mM ammonium acetate using Zeba Spin (7-kDa molecular weight cutoff) desalting columns (Thermo Fisher Scientific). Samples were analyzed by nanoelectrospray ionization MS using a quadrupole orbitrap MS (Q-Exactive UHMR, Thermo Fisher Scientific) using gold–palladium-coated nanospray tips prepared in-house. The MS instrument was operated in positive ion mode using a capillary voltage of 1.5 kV, capillary temperature of 250 °C and S-lens radiofrequency of 200 V. In-source trapping was used with a desolvation voltage of −200 V for 4 μs. Extended trapping was not used. The quadrupole mass range was 2,000–15,000 *m*/*z*. Nitrogen gas was used in the higher-energy collisional dissociation cell with a trap gas pressure setting of 5. Orbitrap resolution was 6,250 and detector *m/z* optimization was low. Five microscans were averaged and an automatic gain control target of 2 × 10^5^ was used. Mass calibration was performed by a separate injection of sodium iodide at a concentration of 2 μg μl^−1^. Data processing was performed using QualBrowser (version 4.2.28.14) and deconvoluted using UniDec^82^.

### HDX–MS

HDX–MS experiments were carried out using an automated HDX robot (LEAP Technologies) coupled to an M-Class Acquity LC and HDX Manager (Waters). First, 5 μM BRISCΔNΔC was mixed with 500 μM FX-171-C or DMSO (2.5% v/v) in buffer containing 25 mM HEPES pH 7.5, 150 mM NaCl and 1 mM TCEP and incubated for 30 min at 4 °C. For labeling, 5 μl of BRISC mixed with inhibitor or DMSO was diluted in 95 μl of deuterated buffer (50 mM potassium phosphate and 200 mM NaCl, pH 7.5) and incubated at 4 °C for 0, 0.5, 1, 10 or 60 min. The sample was quenched by adding quench buffer (50 mM potassium phosphate, pH 2.1) at a 1:1 ratio and dropping the temperature to 0 °C. Then, 50 μl of quenched sample was passed through an immobilized pepsin column (AffiPro) at 115 μl min^−1^ and trapped on a VanGuard Precolumn Acquity UPLC BEH C18 (1.7 µm, 2.1 mm × 5 mm; Waters) for 3 min in 0.3% v/v formic acid in water. The resulting peptic peptides were transferred to a C18 column (75 µm × 150 mm; Waters) and separated by gradient elution of 0–40% acetonitrile (0.1% v/v formic acid) in H_2_O (0.3% v/v formic acid) over 7 min at 40 µl min^−1^. Trapping and gradient elution of peptides were performed at 0 °C. The HDX system was interfaced to a Synapt G2Si MS instrument (Waters). High-definition MSE and dynamic range extension modes (data-independent acquisition coupled with ion mobility spectrometry separation) were used to separate peptides before collision-induced dissociation fragmentation in the transfer cell. HDX data were analyzed using PLGS (version 3.0.2) and DynamX (version 3.0.0) software supplied with the MS instrument. Restrictions for identified peptides in DynamX were as follows: minimum intensity, 10,000; minimum products per MS/MS spectrum, 3; minimum products per amino acid, 0.3; maximum sequence length, 18; maximum ppm error, 10; file threshold, 8/9. Following manual curation of the data, Woods and individual uptake plots were generated using Deuteros (version 2.0)^83^. A summary of the HDX–MS data, as recommended by reported guidelines^84^, is shown in Supplementary Table 1.

### Dianthus spectral shift binding assay

Site-specific labeling of His-tagged BRISC was performed using a RED-Tris-NTA second-generation labeling kit (NanoTemper Technologies) in buffer containing 25 mM HEPES pH 7.5, 150 mM NaCl, 1 mM DTT and 0.005% Tween-20. First, 100 nM His–BRISC was incubated with 25 nM RED-Tris-NTA dye and prepared according to the Nanotemper protocol. To measure the affinity of the interaction between BRISC and SHMT2, 12.5 nM labeled BRISC was mixed with SHMT2(A285T) in a 16-point, twofold dilution series from 46 μM to 1.4 nM. To measure the effect of the compounds on the BRISC–SHMT2 interaction, NTA-labeled His–BRISC was incubated with 1% DMSO or 100 μM compound (1% DMSO) for 15 min at room temperature. The BRISC–compound mix was then incubated with SHMT2(A285T) for 30 min at 25 °C. The reactions were carried out in 384-well Dianthus microplates (Nanotemper Technologies) with a 20-μl reaction volume. The measurements were performed using autoexcitation on a Dianthus NT.23 instrument at 25 °C using DI.Control software (version 2.1.1; Nanotemper Technologies). Data were analyzed using DI.Screening Analysis software (version 2.1.1; Nanotemper Technologies) and plotted in GraphPad Prism (version 10.1.0). *K*_D_ values were determined using a GraphPad Prism built-in equation for total binding (one site).

### Immunoblotting, flow cytometry, TUBE pulldown assay and gene expression analyses

Extended experimental methods for experiments in THP-1 cells, MCF10A cells and PBMCs are in Supplementary Protocol 1.

### Statistical analysis

Comparisons between two conditions were conducted using paired and unpaired Student’s *t*-tests. Comparisons between multiple conditions were conducted using a one-way analysis of variance (ANOVA) with Dunnett’s multiple-comparisons test or two-way ANOVAs. Statistical significance was defined as *a P* value less than 0.05 for all analyses. Data analysis was performed using GraphPad Prism (versions 9.5.1 and 10.3.1).

### Reporting summary

Further information on research design is available in the Nature Portfolio Reporting Summary linked to this article.

## Online content

Any methods, additional references, Nature Portfolio reporting summaries, source data, extended data, supplementary information, acknowledgements, peer review information; details of author contributions and competing interests; and statements of data and code availability are available at 10.1038/s41594-025-01517-5.

## Supplementary information

Supplementary Information Supplementary Protocols 1 and 2.

Reporting Summary

Supplementary Tables 1 and 2 HDX–MS data summary table and clinical details of participants included in the study.

## Source data

Source Data Fig. 1 Statistical source data.

Source Data Fig. 4 Statistical source data.

Source Data Fig. 5 Statistical source data.

Source Data Fig. 6 Statistical source data.

Source Data Extended Data Fig. 1 Statistical source data.

Source Data Extended Data Fig. 2 Statistical source data.

Source Data Extended Data Fig. 2 Uncropped gels.

Source Data Extended Data Fig. 3 Statistical source data.

Source Data Extended Data Fig. 6 Statistical source data.

Source Data Extended Data Fig. 7 Uncropped gels.

Source Data Extended Data Fig. 7 Statistical source data.

Source Data Extended Data Fig. 8 Uncropped western blots.

Source Data Extended Data Fig. 8 Statistical source data.

Source Data Extended Data Fig. 9 Statistical source data.

## Data Availability

Cryo-EM maps were deposited to the EM Data bank under accession codes EMD-17980 and EMD-18009. Model coordinates were deposited to the PDB under the accession codes 8PVY and 8PY2. HDX data are available through ProteomeXchange under dataset identifier PXD044584. PDB models 6H3C, 6R8F, 2ZNV, 5J0G, 4ONN, 4ONM and 3RZ3 were also used for model building or figure generation. All unique reagents are available upon request. Source data are provided with this paper.
