# Supplementary material for: Molecular glues that inhibit deubiquitylase activity and inflammatory signaling
Source: Nat Struct Mol Biol. 2025 Mar 17;32(9):1812–24. doi: 10.1038/s41594-025-01517-5 (PMC7617869; doi:10.1038/s41594-025-01517-5)
Supplement: Supplementary file 1 — Supplementary Protocols 1 and 2. [file 41594_2025_1517_MOESM1_ESM.pdf]

nature structural & molecular biology

Supplementary information https://doi.org/10.1038/s41594-025-01517-5

Molecular glues that inhibit deubiquitylase activity and inflammatory signaling

In the format provided by the authors and unedited

Supplementary Protocols 1. Experimental procedures for cell biology experiments.

Cell lysis, immunoprecipitation, and immunoblotting

Media supplements including human insulin solution (Santa Cruz Biotechnology, #sc-360248), cholera toxin from vibrio (Sigma Aldrich, #C8052-2MG), recombinant human EGF (PeproTech, #AF-100-15-100μg), hydrocortisone (Sigma-Aldrich, #H-0888) were used to grow MCF10A cells. The 4–12% Bis-Tris SDS-PAGE gels, cell culture media, horse serum and other materials for tissue culture were purchased from Invitrogen. LipoD293 (SigmaGen, #SL100668) was used for all the transfections to generate knock out cell lines.

Immortalised breast epithelial cell line MCF10A were cultured in 10 cm plates with Dulbecco's Modified Eagle Medium (DMEM) + F-12 (1:1) supplemented with 5% horse serum, 100 ng/mL cholera toxin, 10 μg/mL insulin, 20 ng/mL epidermal growth factor (EGF) and 0.5 μg/mL hydrocortisone until >80% confluence. 1.2 x10^5 cells were then seeded into 6 cm plates. Cells were left to reach ultra-confluence. Once confluent, cells were washed with phosphate buffered saline (PBS) and starved for 30 minutes in 2 mL DMEM + F-12 (1:1) supplemented with 1% horse serum. After 30 minutes cells were pre-treated with either DMSO (0.1%) (control and IFN only) or BRISC inhibitor (2.5 μM) for 15 minutes. After 15 minutes, hIFN2α (Biolegend, #592702, 75 ng/mL) was added to the media and mixed it well. Cells were treated for indicated time or otherwise 1 hour (without inhibitors) or 4 hours (with BRISC inhibitors). Cells were washed with ice cold PBS and scraped with cell scrapers into PBS and centrifuged at 900 g for 10 minutes. Cell pellets were either resuspended into radioimmunoprecipitation assay (RIPA) buffer supplemented with complete ethylenediaminetetraacetic acid (EDTA) free protease inhibitor cocktail and 25 U/mL benzonase for immunoblotting or in immunoprecipitation buffer (100 mM NaCl, 0.2% Igepal CA-630, 1 mM $MgCl_2$, 10% glycerol, 5 mM NaF, 50 mM Tris-HCl, pH 7.5), supplemented with complete EDTA free protease inhibitor cocktail and 25 U/mL benzonase for co-immunoprecipitation. Lysates were mixed with 2X sample buffer for gel loading prior to Western blot analysis. Co-immunoprecipitation was performed using Anti-Flag M2 Affinity Gel beads (Sigma-Aldrich #A2220) and eluted with 0.2 M Glycine. The following antibodies were used for immunoblotting: Anti-BRCC45 (Abcam, #ab177960), Anti-GAPDH (Cell Signaling Technology, #2118S), Anti-Merit40 (Cell Signaling Technology, #12711S), Anti-BRCC36 (Abcam, #ab108411), Anti-Phospho-Stat1 (Tyr701) (Cell Signaling Technology, #9167S), anti-IFNAR1 antibody (ab124764, Abcam).

Real-time quantitative PCR

1x10^5 MCF10A cells were cultured as described above and seeded into 6 well plates. Cells were left to reach ultra-confluence. Once confluent, cells were washed with PBS and starved for 30 minutes in 2 mL DMEM + F-12 (1:1) supplemented with 1% horse serum. After 30 minutes cells were pre-treated with either DMSO (0.1%) (Control and IFN only) or BRISC inhibitor (2.5 μM) for 15 minutes. After 15 minutes, hIFN2α (75 ng/mL) was added to the media and mixed. Cells were treated for 4 hours. Cells were washed with ice cold PBS and scraped with cell scrapers into 350 μL RNAlater buffer and RNA was isolated using RNeasy mini kit (Qiagen, #74104) and treated with on column DNase. (Qiagen, #79254). Isolated RNA was quantified using a Nanodrop spectrophotometer. RNA was converted into cDNA and the complementary DNA was used for real-time quantitative PCR analysis of expression of interferon stimulated genes (*ISG15*, *IFIT1*, *IFIT2*, *IFITM1*, *CXCL10* and *RNA18S*) using an Applied Biosystems Quantstudio 6 RT-PCR system. All experiments were carried out in triplicate.

IFNAR1 flow cytometry

To study the direct effect of BRISC DUB inhibitors, IFNAR1 surface levels were measured using flow cytometry (FACS) analysis. For this purpose, MCF10A WT and MCF10A BRCC45 KO cells expressing BRCC45 WT or BRCC45 R137A were used. Cells were pre-incubated with 5 µM FX-171-C for 30 minutes prior to the addition of 50 ng/mL of hIFN-Iα for various time points (45 minutes and 90 minutes). After IFN treatment, cells were dissociated from plate using cell dissociation Buffer (ThermoFisher Scientific, #13150016) and were stained with LIVE/DEAD™ Fixable Violet Dead Cell Stain Kit, for 405 nm excitation (ThermoFisher Scientific, #L34963) for 30 minutes, as per the kit protocol. The cells were then washed using FACS buffer (DPBS, 1% BSA, 2 mM EDTA, 0.09% sodium azide) and transferred to 96-well round bottom plates. Resuspended cells were divided into three repeats for each sample, each was surface stained for 1 hour at 4 °C with: 1. Human IFN-alpha/beta R1 Antibody (R&D systems, #MAB245), 2. Mouse IgG1 Isotype Control (R&D systems, #MAB002), 3. FACS buffer (control cells). Following incubation, cells were washed once with FACS buffer and were labelled for 30 minutes at 4 °C with either Biotin-SP-conjugated AffiniPure Donkey Anti-Mouse IgG (H+L) (Jackson Immunoresearch Laboratories) for samples 1 and 2 or with FACS buffer for sample 3. Cells were washed twice with FACS buffer and were labelled for 15 minutes at 4 °C with either R-Phycoerythrin-conjugated Streptavidin (Jackson Immunoresearch Laboratories) for samples 1 and 2 or with FACS buffer for sample 3. Cells were washed 3 times with FACS buffer and were analysed by FACS (MACSQuant, Miltenyi Biotec). For each sample from group 1 the Geo mean fluorescence intensity (MFI) of its isotype sample was reduced. IFNAR1 cell surface percentage was calculated by 100*(MFI treated sample/MFI of untreated sample). Statistical analysis was performed by one-way and two-way ANOVA.

<u>THP-1 *in vitro* experiments</u>

THP-1-Dual™ cells (InvivoGen), derived from a human monocytic cell line, isolated from a male patient with acute monocytic leukaemia, were cultured as per manufacturer's guidance. Cells were seeded at $1x10^6$ cells/mL in RPMI1640 media containing 10% fetal bovine serum (FBS), 1% penicillin-streptomycin (PS) (Gibco Laboratories) with DMSO (0.1%) (control) or in media containing JMS-175-2, FX-171-C, FX-171-A, or AP-5-145 at 4 µM for 16 hours with and without IFNα2 (25 ng/mL), LPS (100 ng/mL) (Merck Life Science), ODN 2216 (1 µM) (Miltenyi Biotec), or Poly:IC (1 µg/mL) (InvivoGen). Tofacitinib (Cambridge Biosciences) at 0.4 µM was used for comparison against a known JAK inhibitor. Cells were centrifuged at 300 g for 5 minutes. Supernatant was removed and subjected to simultaneous study of the NF-κB pathway, by monitoring the activity of secreted alkaline phosphatase reporter (SEAP), and the IRF pathway, by assessing the activity of a secreted Lucia luciferase using QUANTI-Blue™ Solution and QUANTI-Luc™ 4 Lucia/Gaussia (InvivoGen), respectively, as per manufacturer's instructions. Pelleted cells were used for FACS analysis using IFNα/β R1 APC-conjugated antibody or isotype control (Biotechne, FAB245A and IC002A), or unstained staining procedure with and without Zombie Green™ (Biolegend). Samples were analysed using CytoFlex LX (Beckman) (50,000 live cells per condition). Median fluorescence intensity for APC channel gated on single cell live cells was analysed and compared as a percentage to control (no stimulation condition).

<u>Tandem ubiquitin binding entity (TUBE) pull-down assay</u>

The TUBE pull-down experiment was performed according to the manufacturer's (UM401: TUBE 1 Agarose; LifeSensors) protocol with modifications. MCF10A cells (~20 $x10^6$) were pre-treated with the BLUE inhibitor panel (5 µM) or DMSO vehicle control for 1 hour followed by addition of recombinant IFNα2 (50 ng/mL, 592702, BioLegend). Following 1 hour incubation with IFNα2, cells were lysed in cell lysis buffer (50 mM Tris-HCl, pH 7.5, 0.15 M NaCl, 1 mM EDTA, 1% NP-40, 10% glycerol, 10 mM

NEM). Cell lysates were clarified by centrifugation at 15,000 g for 10 minutes at 4 °C. Clarified lysates were pre-cleared with control uncoupled agarose beads (UM400, LifeSensors) for 30 minutes on a rotator at 4 °C followed by centrifugation at 5,000 g for 2 minutes. Equal amount of pre-cleared lysates from individual samples were incubated with 20 µL Agarose-TUBE beads (pre-equilibrated with 1X Tris-buffered saline (TBS)) overnight on a rotator at 4 °C. Beads were collected by centrifugation at 5,000 g for 2 minutes at RT followed by four stringent washes with 1X TBST (TBS, 0.1% Tween-20). TUBE-pulldown proteins were eluted by boiling Agarose-TUBE beads in 2X LDS sample buffer supplemented with 200 µM DTT for 15 minutes. Eluted proteins were resolved on NuPAGE™ 4–12% Bis–Tris gel followed by Western blotting using anti-IFNAR1 antibody (ab124764, Abcam).

PBMCs *in vitro* experiments

Peripheral blood mononuclear cells (PBMCs) from healthy and SSc donors were separated using density gradient method (Leucosep™, Greiner Bio-One International) from EDTA anticoagulated peripheral blood; isolated cells were washed twice by PBS. PBMCs were then incubated in RPMI1640 media containing 10% FBS, 1% PS with DMSO (0.1%) (CTR) or in media containing JMS-175-2, FX-171-C, or AP-5-145 at 2 µM for 16 hours. For healthy PBMCs, cells were stimulated with 20 ng/mL of IFN (+/- compounds or DMSO). For SSc PBMCs, cells were in basal conditions with no additional IFN stimulation. Cells were centrifuged at 300 g for 10 minutes. Supernatant was removed and subjected to R&D Systems DY266 Human CXCL10/IP-10 DuoSet ELISA (Biotechne) in duplicates, according to manufacturer's guidance. Concentration of CXCL10 was determined according to a 4-parameter logistic regression standard curve. RNA was extracted from cell pellets using TRIzol™ (ThermoFisher Scientific, Waltham, MA) and processed using Quick-RNA Miniprep Kit (Zymo Research) as per the manufacturer's instruction.

Gene expression analysis

For Type I IFN signalling analysis, the human IFN I RT2 Profiler PCR Array (Qiagen, PAMM-016ZE) was performed on available RNA from healthy (n=3) and SSc (n=9) donors. RNA was converted to cDNA using RT2 First Strand Kit (Qiagen). Next, the cDNA was mixed with RT2 SYBR Green Mastermix (Qiagen, Venlo). Successful PCR performance was confirmed by assay tests for genomic DNA contamination, RNA sample quality, as well as single melt curve determination and <35 Ct for each gene, leading to validated gene expression of 67 genes. Relative expression for each gene was determined, firstly by ΔCT calculated using the geometric mean of 4 housekeeping genes (ACTB, GAPDH, HPRT1, RPLP0), followed by 2^(-ΔCT), and expressed as fold change to control, according to manufacturer's guidance. IFN2α-induced ISGs in healthy donors were determined using a Student's t-test (two-tail distribution and equal variances) on the triplicate fold change in 2^(-ΔCT) values between treatment and control group (AP-5-145+IFN2α v DMSO only conditions; 34 genes $p<0.05$ for n=3 donors). Heatmaps were generated using the "pheatmap" function in R (v4.0.2) and RStudio (v1.3.1093) applying row scaling to standardise the expression data across samples (R core team 2020. R Foundation for Statistical Computing). The IFN2α inducible genes determined in healthy donors (n=34) were included, and donors were combined into treatment groups AP-5-145, JMS-175-2 and FX-171-C. To account for Type I IFN signalling heterogeneity across samples, the mean 2^(-ΔCT) for each gene for AP-5-145 treated condition from all donors (healthy, n=3 or SSc, n=9) was used to determine relative gene expression, illustrated as a fold change and visualised in log2. The Type I IFN signalling array data for the patient samples is included in Source Data Figure 6, Extended Data Fig. 9.

For composite ISG score gene expression analysis, RNA was reverse transcribed using the high-capacity cDNA synthesis kit (Applied Biosystems). qRT-PCR were performed using SyBr Green PCR kit (ThermoFisher Scientific) with primers specific for *CXCL10* (Forward; TCCAGTCTCAGCACCATGAA Reverse; AGGTACTCCTTGAATGCCACT), *MX1* (Forward; CGACACGAGTTCCACAAATG Reverse; AAGCCTGGCAGCTCTCTACC), *IFIT1* (Forward; GACTGGCAGAAGCCCAGACT Reverse; GCGGAAGGGATTTGAAAGCT), *ISG15* (Forward; GTGGACAAATGCGACGAACC Reverse; ATTTCCGGCCCTTGATCCTG), and *GAPDH* (Forward; ACCCACTCCTCCACCTTTGA Reverse; CTGTTGCTGTAGCCAAATTCGT). The data obtained was analysed according to the ΔΔ Ct method relative to *GAPDH*. For composite IFN score, fold change in gene expression in AP-5-145, JMS-175-2 and FX-171-C treated samples (of each gene *CXCL10*, *MX1*, *IFIT1*, *ISG15*) was calculated relative to each donor DMSO control (CTR). The composite score represents grouped analysis of combined fold changes for all 4 genes.

Supplementary Protocols 2. Experimental procedures for compound synthesis

General Methods for Chemistry. All solvents and chemicals were used as purchased without further purification. The progress of all reactions was monitored on Merck precoated silica gel plates (with fluorescence indicator UV254) using the solvent system indicated. Column chromatography was performed with silica gel 60 (230−400 mesh ASTM) or performed using an automated Biotage Isolera one automated flash purification system with the solvent mixtures specified in the corresponding experiment. TLC plates were visualized by irradiation with ultraviolet light (254 nm). Proton (^{1}H) and carbon (^{13}C) NMR spectra were recorded on a Bruker AVANCE III 400 High Performance Digital NMR Spectrometer, and a 300MHz and 500MHz Varian Unity Inova NMR systems. Chemical shifts are reported in parts per million (ppm, δ) using the residual solvent line as a reference. Splitting patterns are designated using the following abbreviations: s, singlet; d, doublet; t, triplet; dd, doublet of doublet; m, multiplet; br, broad. Coupling constants (J) are reported in hertz (Hz). Compound purity was determined by LCMS and NMR. LCMS was obtained on a Waters Acquity QDa UPLC/MS mass spectrometer with an electrospray ionization (ESI) source and a PDA detector (210−400 nm). The purity of all final compounds was 95% or higher. High-resolution accurate mass LC-MS/MS data were acquired on a Thermo Q Exactive Plus mass spectrometer coupled with a Waters Nano-ACQUITY UPLC system.

Experimental procedure ^{1}H NMR & LC-MS for JMS-175-2 and FX-171C and control compounds.

Schemes

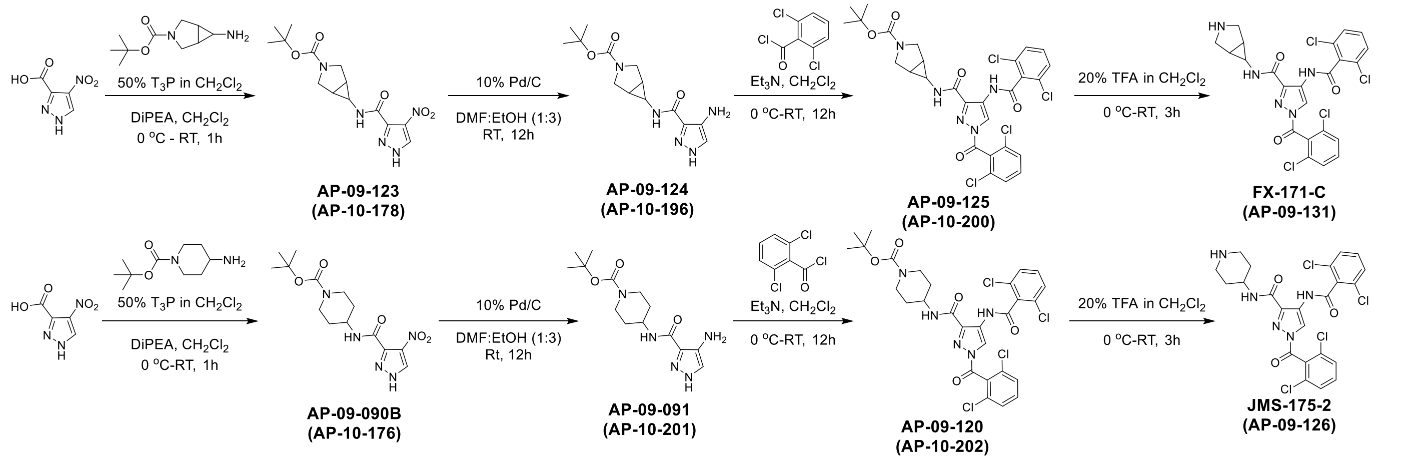

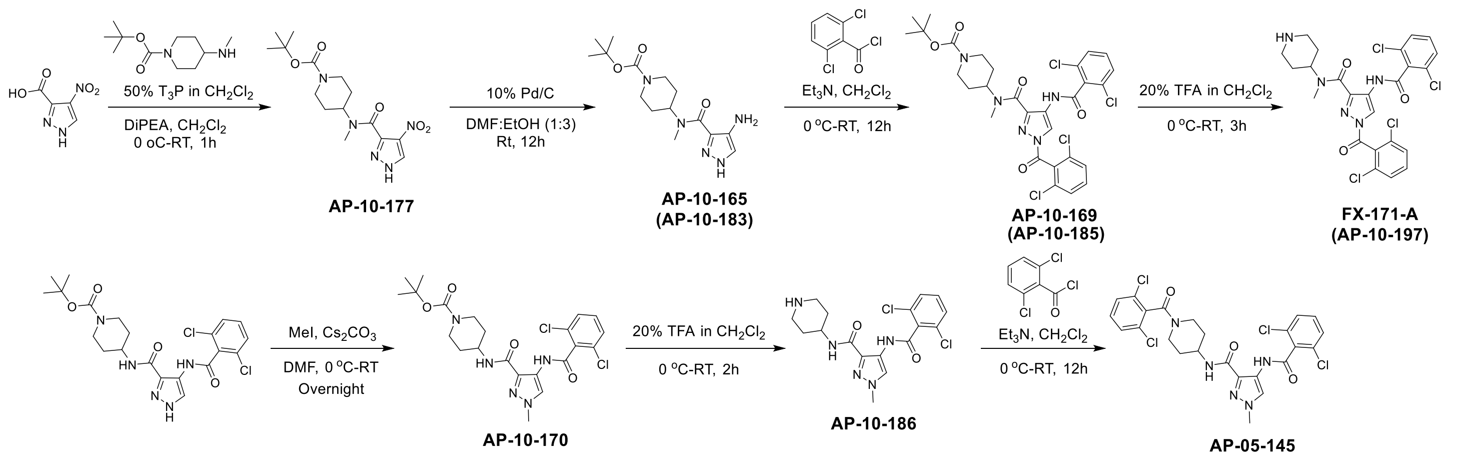

Tert-butyl 6-(4-nitro-1H-pyrazole-3-carboxamido)-3-azabicyclo [3.1.0] hexane-3-carboxylate: AP-09-123 (AP-10-178)

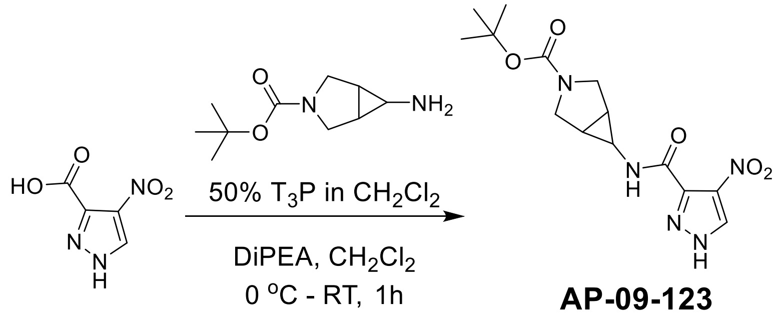

AP-09-123
(AP-10-178)

To a stirred solution of 4-nitro-1H-pyrazole-3-carboxylic acid (1.0 g; 6.36 mmol) and tert-butyl 6-amino-3-azabicyclo[3.1.0]hexane-3-carboxylate (1.325 g; 6.68 mmol) in 20 mL of dry CH_2Cl_2 at 0 °C was added diisopropyl ethyl amine (5.56 mL; 31.82 mmol) and T3P 50% solution in CH_2Cl_2 by weight (5.26 g; 8.28 mmol) simultaneously dropwise. The reaction mixture was stirred for an additional 1 hour at 0 °C. Completion of the reaction was confirmed by LC−MS. The product was extracted with CH_2Cl_2 (10 mL X 2) and washed with 1N *aq* HCl (20 mL), saturated *aq* $NaHCO_3$ (20 mL), brine solution (20 mL) and dried over anhydrous Na_2SO_4. The solvent was evaporated under reduced pressure to yield the crude product, which was purified by flash column chromatography to afford the title compound as a white solid (1.74 g; 5.16 mmol, 82%) which was confirmed by ^{1}H, ^{13}C NMR and HRMS.

^{1}H NMR (400 MHz, DMSO-*d_6*)) δ 8.82 (d, *J* = 3.9 Hz, 1H), 8.79 (s, 1H), 5.76 (DCM), 3.58 – 3.51 (m, 2H), 3.42 – 3.29 (m, 2H), 2.50 (DMSO) 2.50 – 2.47 (m, 1H), 1.78 – 1.72 (s, 2H), 1.40 (s, 9H).

^{13}C NMR (101 MHz, DMSO) δ 161.1, 154.0, 132.8, 131.8, 79.1, 47.9, 32.9, 28.6, 23.9, 22.1.

HRMS: Calcd (m/z) for $C_{14}H_{20}N_5O_5^+$ [M + H] $^+$, 338.1459; found, 338.1457.

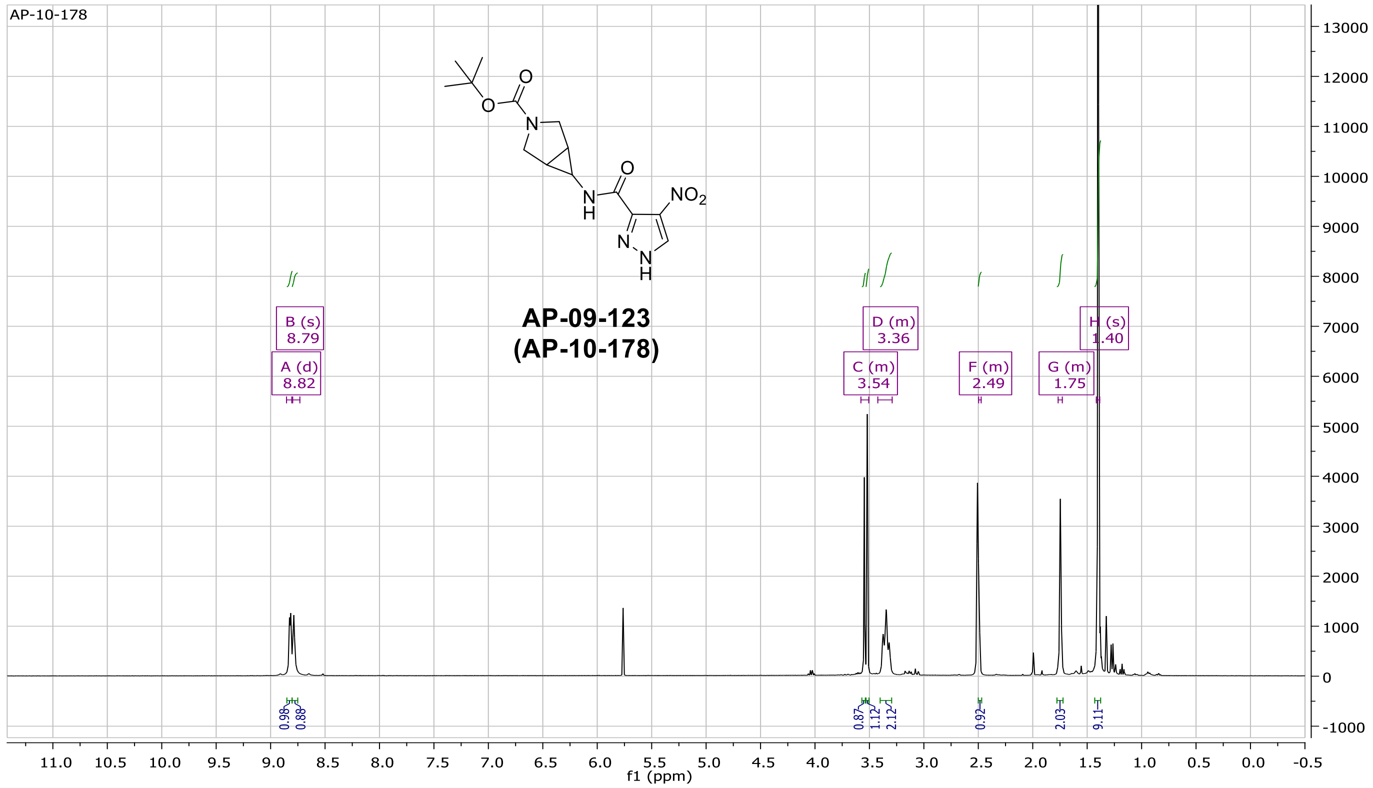
AP-10-178
AP-09-123
(AP-10-178)
NO_2
A (d) 8.82
B (s) 8.79
C (m) 3.54
D (m) 3.36
F (m) 2.49
G (m) 1.75
H (s) 1.40
0.98 0.88 0.87 1.12 2.12 0.92 2.03 9.11
f1 (ppm)

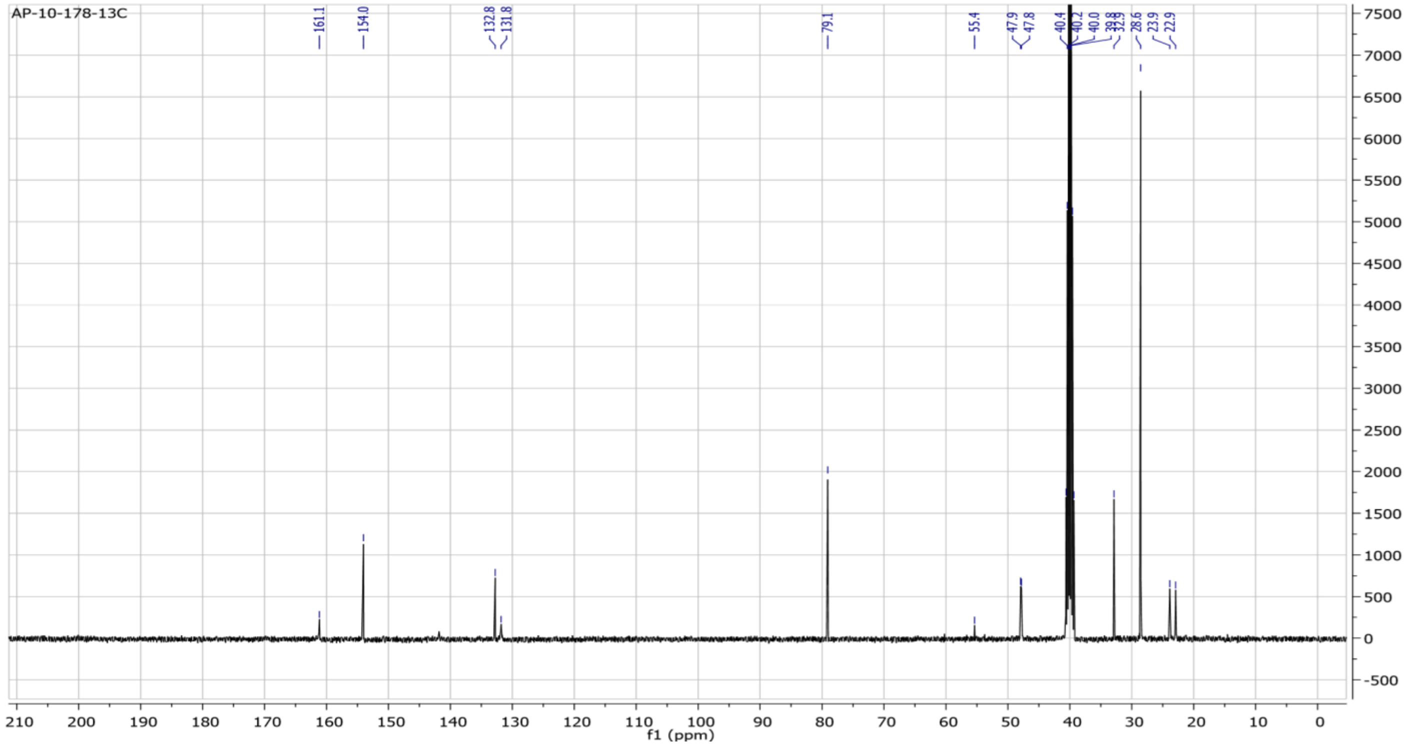
AP-10-178-13C
161.1
154.0
132.8
131.8
79.1
55.4
47.9
47.8
40.4
40.2
40.0
39.8
32.9
28.6
23.9
22.9
f1 (ppm)

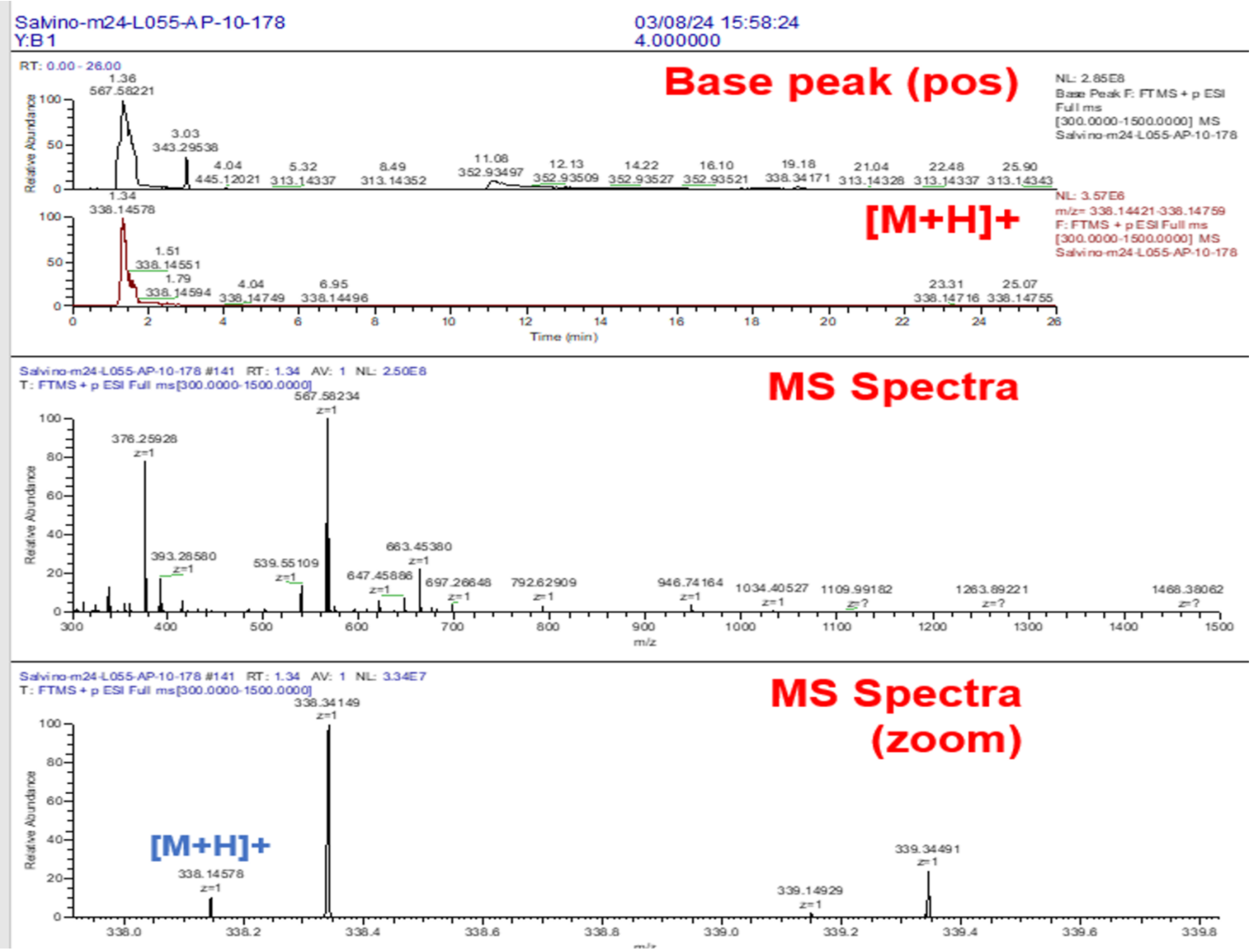

Tert-butyl 6-(4-amino-1H-pyrazole-3-carboxamido)-3-azabicyclo[3.1.0]hexane-3-carboxylate: AP-09-124 (AP-10-196)

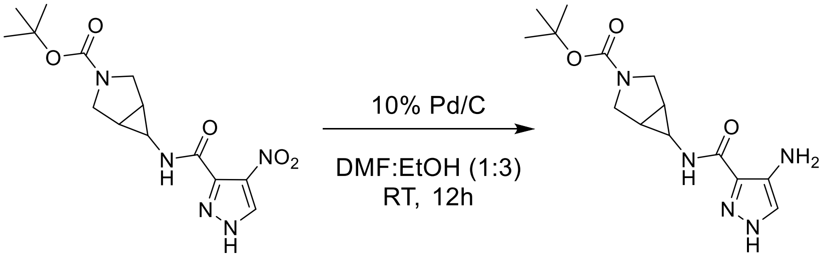

AP-09-123 (AP-10-178) → **AP-09-124 (AP-10-196)**

At room temperature, a suspension of tert-butyl 4-(4-nitro-1H-pyrazole-3-carboxamido)piperidine-1-carboxylate (1.7 g; 5.04 mmol) and Pd/C (170 mg) in DMF (10 mL) and EtOH (30 mL) was stirred for 12 h under H_2 at room temperature. Completion of the reaction was confirmed by TLC, the reaction mixture was filtered using Celite, and the filtrate was concentrated under reduced vacuum to obtain a residue which was diluted in ethyl acetate and washed with cold brine solution and dried over anhydrous

Na_2SO_4. The solvent was evaporated under reduced pressure to yield the crude product, which was purified by flash column chromatography to afford the title compound as a light brown solid (1.28 g; 4.18 mmol, 83%) which was confirmed by ^1H, ^{13}C NMR and HRMS.

^1H NMR (400 MHz, DMSO) δ 12.55 (s, 1H), 7.98 (m, 1H), 7.10 (s, 1H), 4.52 (s, 2H), 3.58-3.344 (m, 2H), 3.41-3.24 (m, 2H), 2.45 – 2.27 (m, 1H), 1.86-1.67 (m, 2H), 1.39 (s, 9H).

^{13}C NMR (101 MHz, DMSO) δ 165.4, 154.0, 133.4, 132.0, 115.2, 79.0, 47.9, 32.2, 28.6, 24.0, 23. 0

HRMS: Calcd m/z for $C_{14}H_{22}N_5O_3^+$ [M +H] +, 308.1717; found, 308.1716.

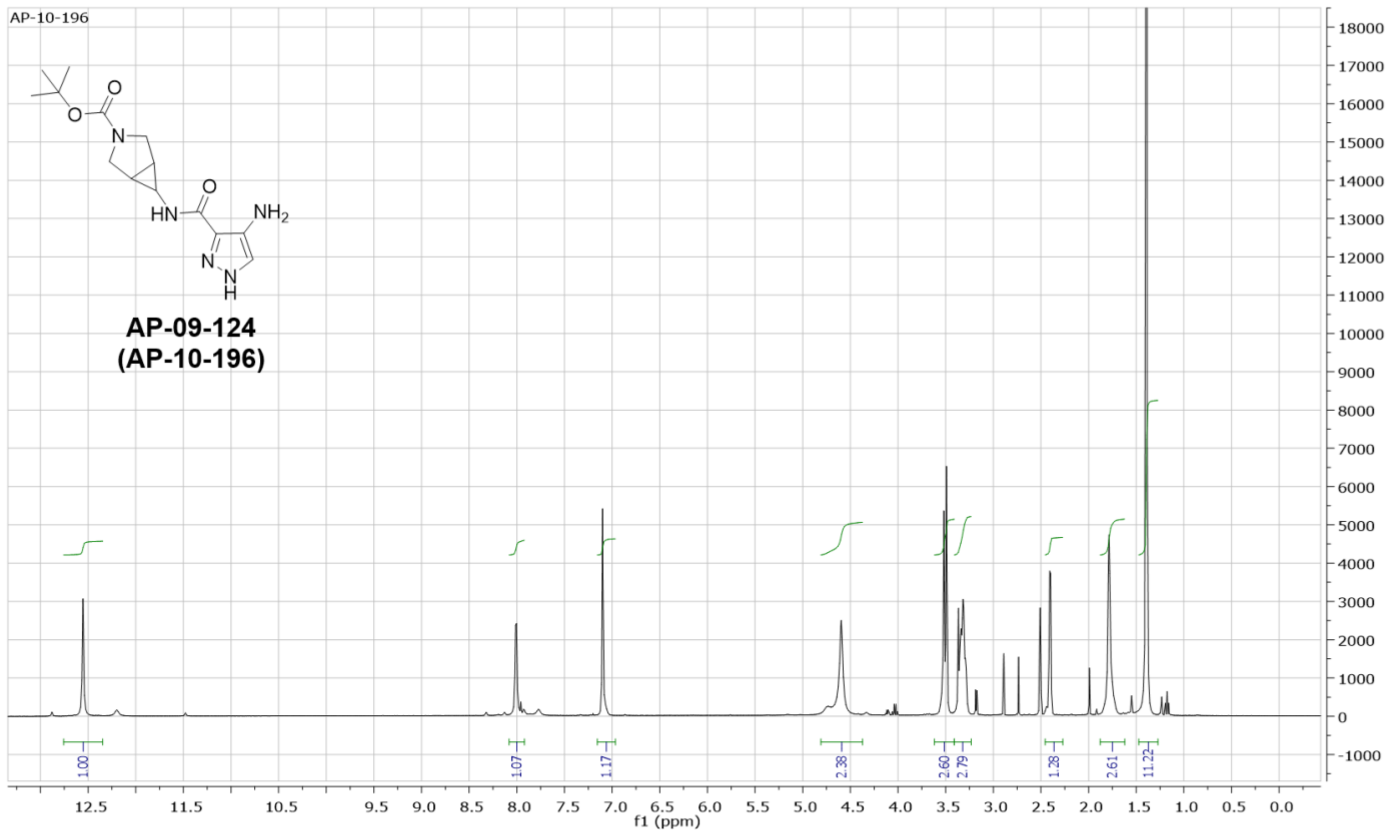

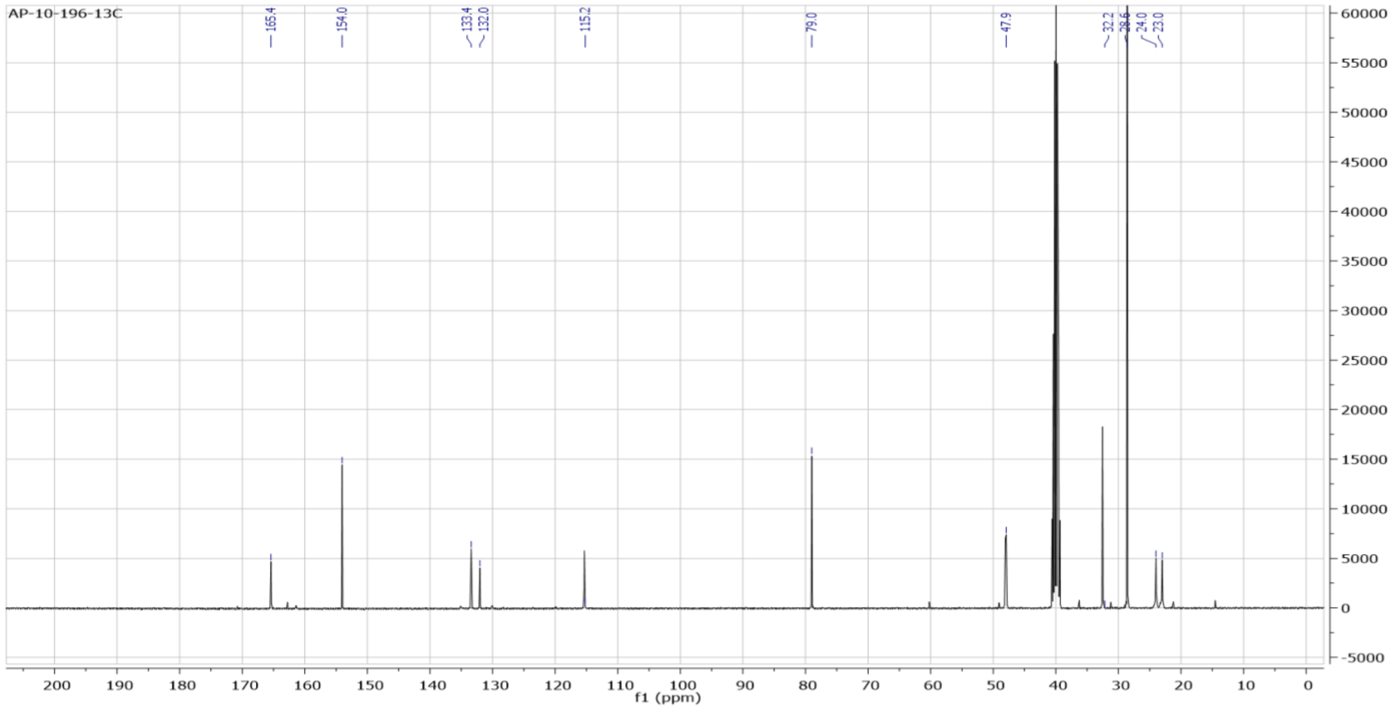
AP-10-196-13C
165.4
154.0
133.4
132.0
115.2
79.0
47.9
32.2
28.6
24.0
23.0
f1 (ppm)

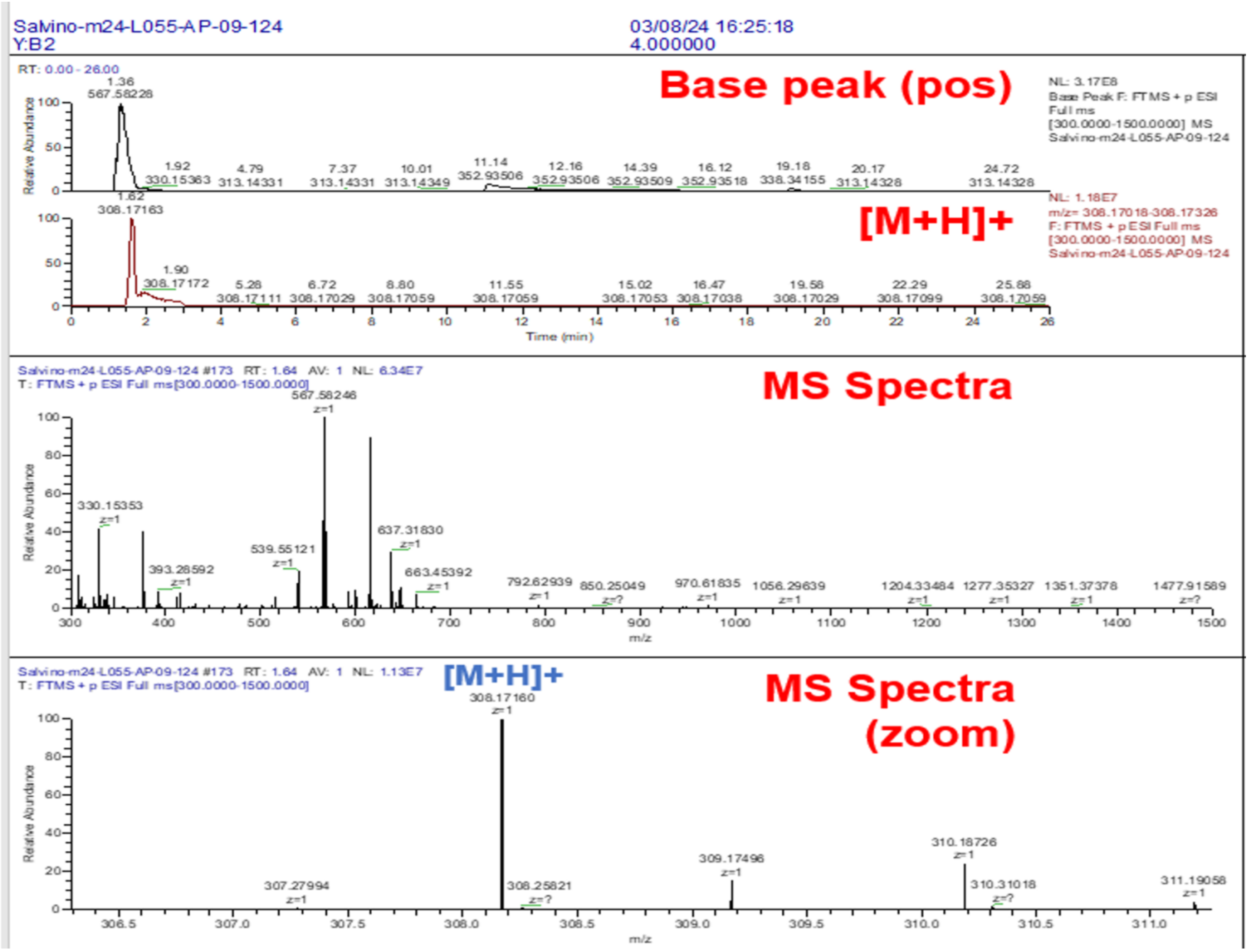
Salvino-m24-L055-AP-09-124
Y:B2
03/08/24 16:25:18
4.000000
RT: 0.00 - 26.00
Base peak (pos)
NL: 3.17E8
Base Peak F: FTMS + p ESI Full ms [300.0000-1500.0000] MS Salvino-m24-L055-AP-09-124
1.36
567.58228
[M+H]+
NL: 1.18E7
m/z= 308.17018-308.17326 F: FTMS + p ESI Full ms [300.0000-1500.0000] MS Salvino-m24-L055-AP-09-124
1.62
308.17163
Time (min)
Relative Abundance
MS Spectra
Salvino-m24-L055-AP-09-124 #173 RT: 1.64 AV: 1 NL: 6.34E7
T: FTMS + p ESI Full ms[300.0000-1500.0000]
567.58246
330.15353
637.31830
539.55121
393.28592
663.45392
m/z
MS Spectra (zoom)
Salvino-m24-L055-AP-09-124 #173 RT: 1.64 AV: 1 NL: 1.13E7
T: FTMS + p ESI Full ms[300.0000-1500.0000]
[M+H]+
308.17160
z=1
309.17496
310.18726
311.19058

Tert-butyl 6-(4-(2,6-dichlorobenzamido)-1-(2,6-dichlorobenzoyl)-1H-pyrazole-3-carboxamido)-3-azabicyclo[3.1.0]hexane-3-carboxylate: AP-09-125 (AP-10-200)

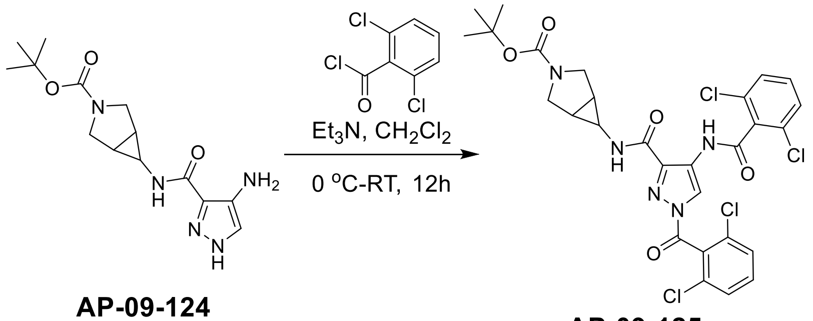

AP-09-124 (AP-10-196)

AP-09-125 (AP-10-200)

To a stirred solution of tert-butyl 6-(4-amino-1H-pyrazole-3-carboxamido)-3-azabicyclo[3.1.0]hexane-3-carboxylate (178 mg; 0.58 mmol) in 5 mL of dry CH_2Cl_2 at 0 °C was added triethyl amine (0.81 mL; 5.79 mmol) and 2,6-dichlorobenzoyl chloride (727 mg; 3.47 mmol) simultaneously dropwise. The reaction mixture was slowly brought to room temperature and stirred for 16 hours. Completion of the reaction was confirmed by LC−MS. The reaction mixture was quenched with 10 mL of cold water. The product was extracted with CH_2Cl_2 and washed with 1N *aq* HCl (10 mL), saturated *aq* $NaHCO_3$ (10 mL), brine solution (10 mL) and dried over anhydrous Na_2SO_4. The solvent was evaporated under reduced pressure to yield the crude product, which was purified by flash column chromatography to afford the title compound as a white solid (151 mg; 0.23 mmol, 40%) which was confirmed by ^{1}H, ^{13}C NMR and HRMS.

^{1}H NMR (400 MHz, DMSO) δ 10.66 (s, 1H), 9.06 (s, 1H), 8.77 (s, 1H), 7.789-7.60 (m, 3H), 7.63 – 7.49 (m, 3H), 3.55 – 3.40 (m, 2H), 3.37-3.22 (m, 2H), 2.45-2.29 (m, 1H), 1.96-1.73 (m, 2H), 1.36 (s, 9H).

^{13}C NMR (101 MHz, DMSO) δ 162.6, 162.4, 154.0, 141.9, 135.5, 132.4, 131.9, 131.8, 129.0, 128.71, 125.0, 119.2, 79.1, 47.8, 32.9, 28.5, 23.9, 22.9.

HRMS: Calcd m/z for $C_{28}H_{26}Cl_4N_5O_5^+$ [M + H] $^+$, 652.0683; found, 652.0684.

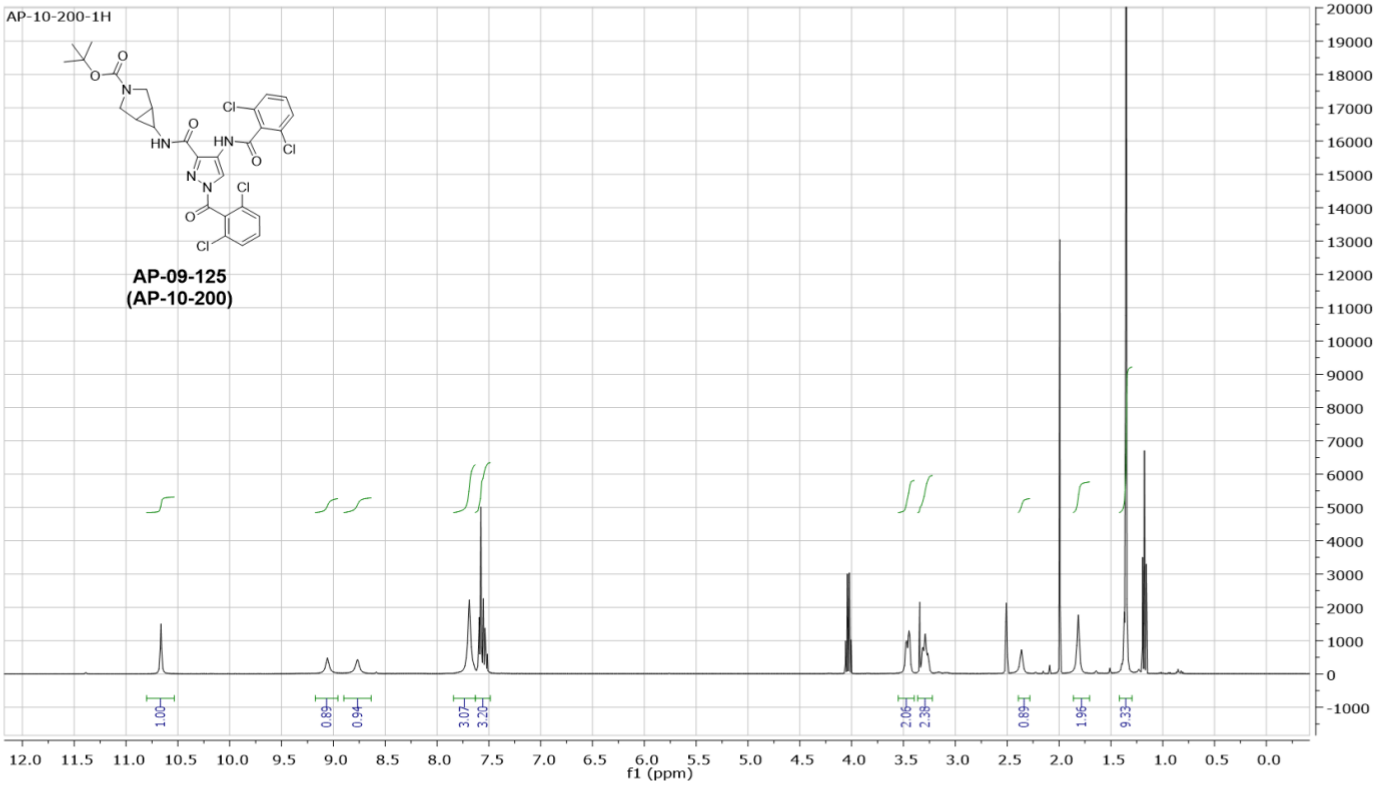
AP-10-200-1H
AP-09-125
(AP-10-200)
1.00
0.89
0.94
3.07
3.20
2.06
2.38
0.89
1.96
9.33
f1 (ppm)

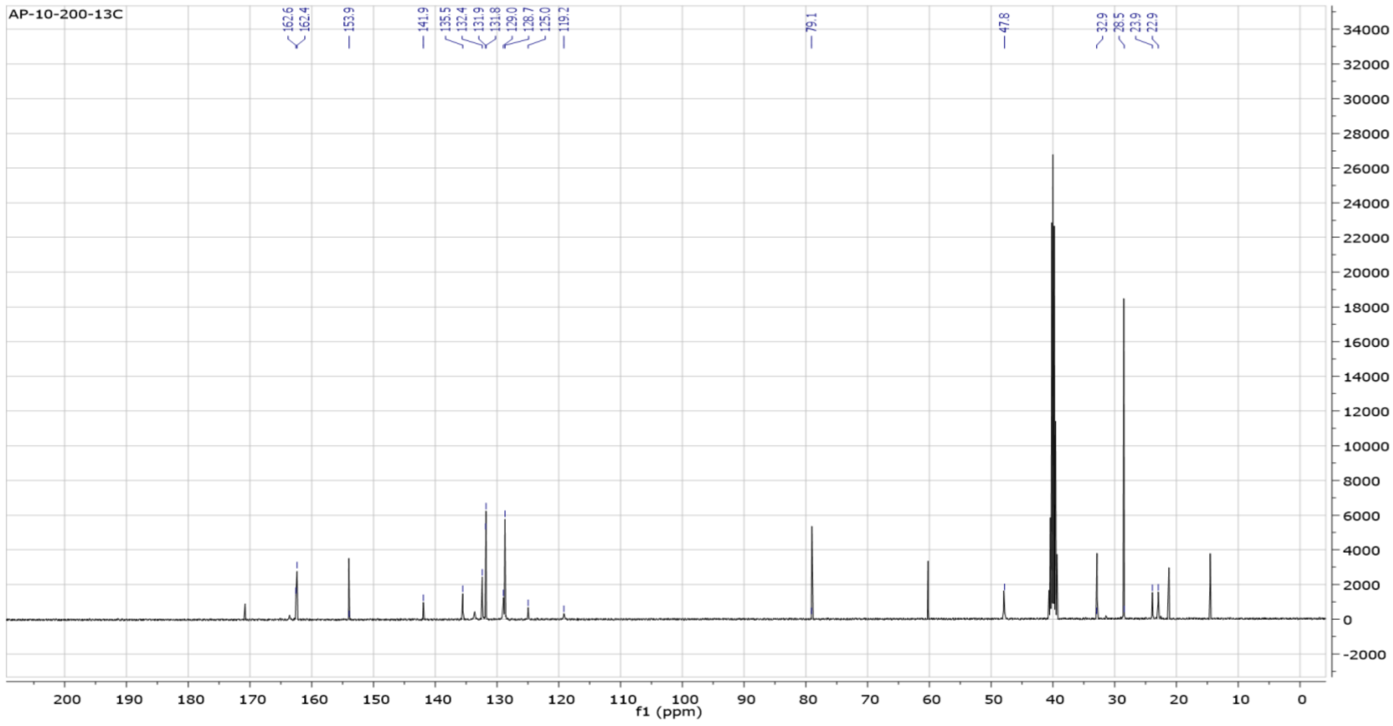
AP-10-200-13C
162.6
162.4
153.9
141.9
135.5
132.4
131.9
131.8
129.0
128.7
125.0
119.2
79.1
47.8
32.9
28.5
23.9
22.9
f1 (ppm)

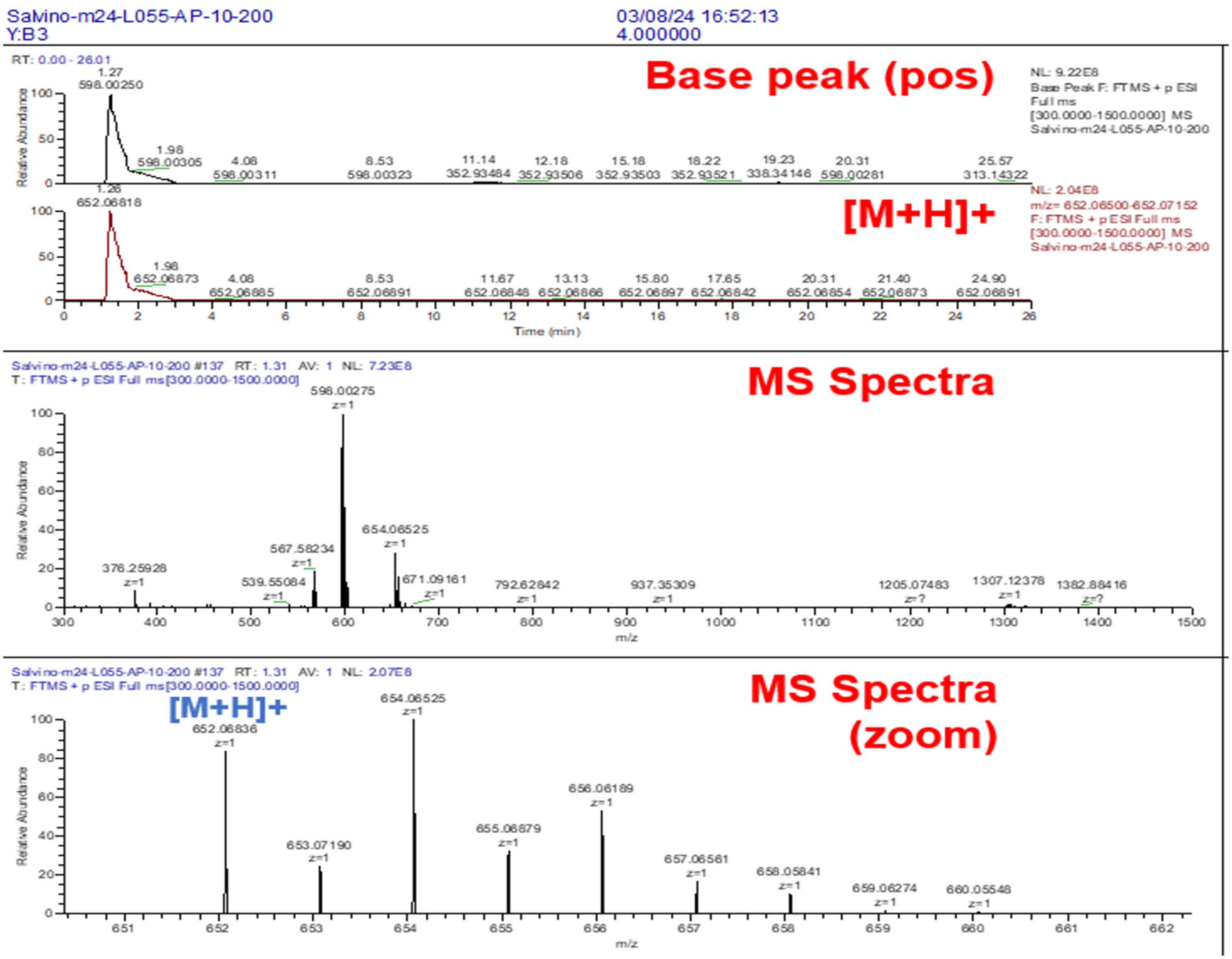

N-(3-azabicyclo[3.1.0]hexan-6-yl)-4-(2,6-dichlorobenzamido)-1-(2,6-dichlorobenzoyl)-1H-pyrazole-3-carboxamide : FX-171C (AP-09-131)

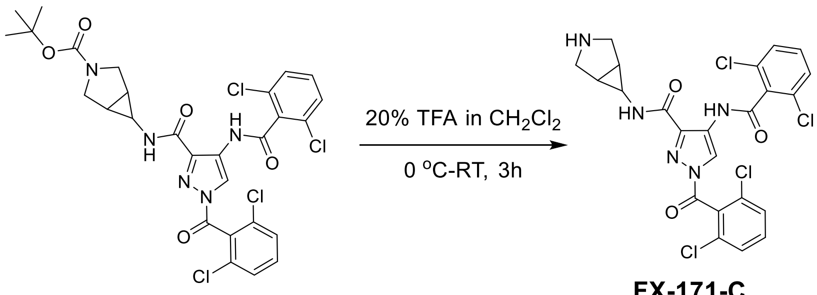

AP-09-125
AP-10-200)

FX-171-C
(AP-09-131)

To a stirred solution of tert-butyl 6-(4-(2,6-dichlorobenzamido)-1-(2,6-dichlorobenzoyl)-1H-pyrazole-3-carboxamido)-3-azabicyclo[3.1.0]hexane-3-carboxylate (60 mg; 0.09 mmol) in 2 mL CH_2Cl_2 at 0 °C was added 0.5 mL of TFA. The reaction mixture was then warmed to room temperature and stirred for 3 hours. Completion of the reaction was confirmed by LC− MS. Volatiles were evaporated under reduced pressure to yield the crude product, which was purified by flash column chromatography to afford the title compound as a white solid (51 mg; 0.08 mmol, 85 %) which was confirmed by ^{1}H, ^{13}C NMR and HRMS.

^{1}H NMR (400 MHz, CDCl3) δ 9.70 (s, 1H), 9.27 (s, 1H), 7.45 – 7.39 (m, 3H), 7.38 – 7.29 (m, 3H), 6.77 (s, 1H), 3.50 – 3.39 (m, 2H), 3.37 – 3.28 (m, 2H), 2.80-2.70 (m, 1H), 2.00-1.92 (m, 2H), 1.35 – 1.19 (pentane), 1.27-123 (m, 1H), 0.88 (pentane).

^{13}C NMR (101 MHz, MeOD) δ 165.3, 164.3, 136.3, 134.3, 134.1, 133.3, 130.0, 129.8, 126.6, 48.7, 31.9, 25.6, 1.3.

HRMS: Calcd m/z for $C_{28}H_{18}Cl_4N_3O_3^+$ [M + H]$^+$, 552.0158; found, 552.0157.

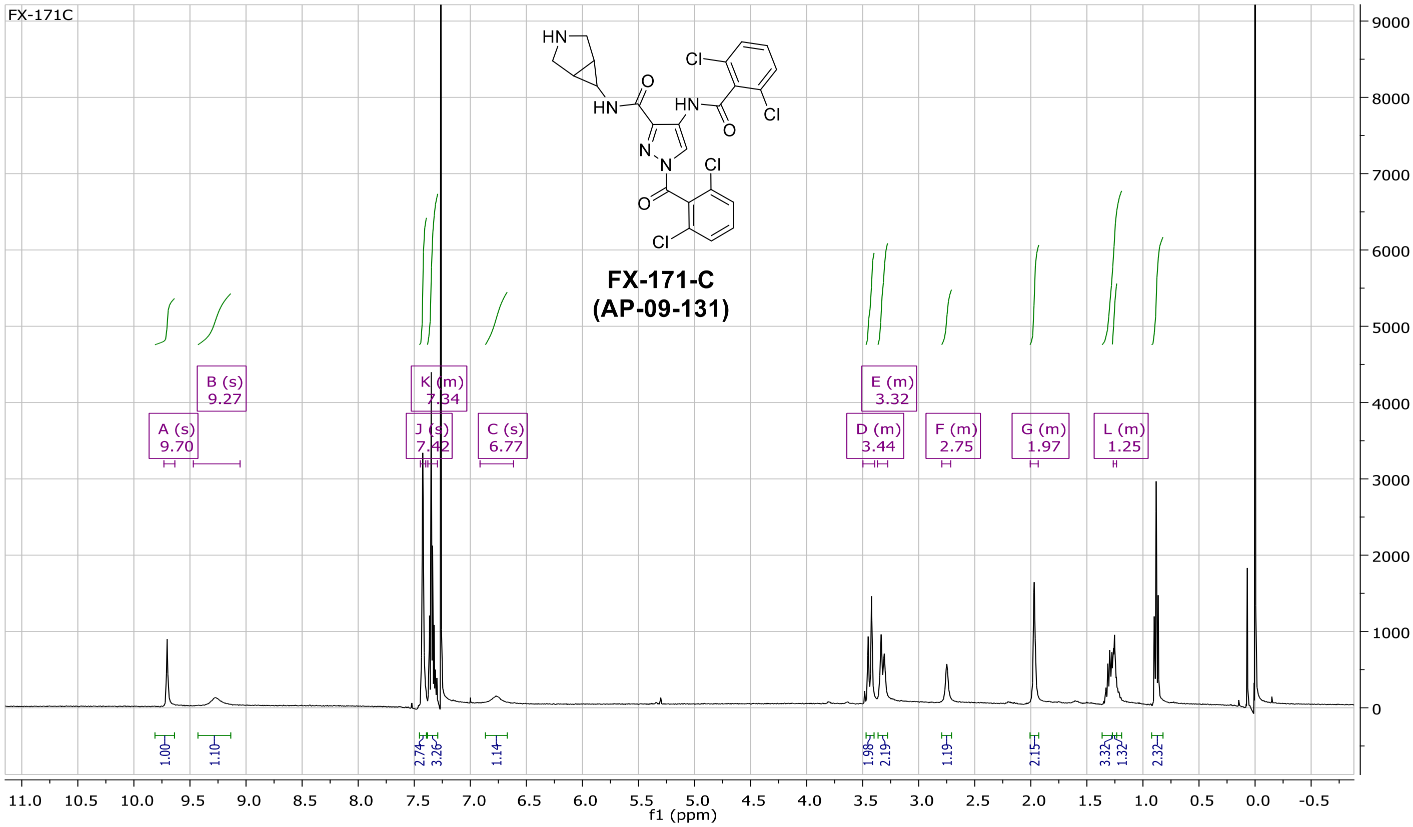

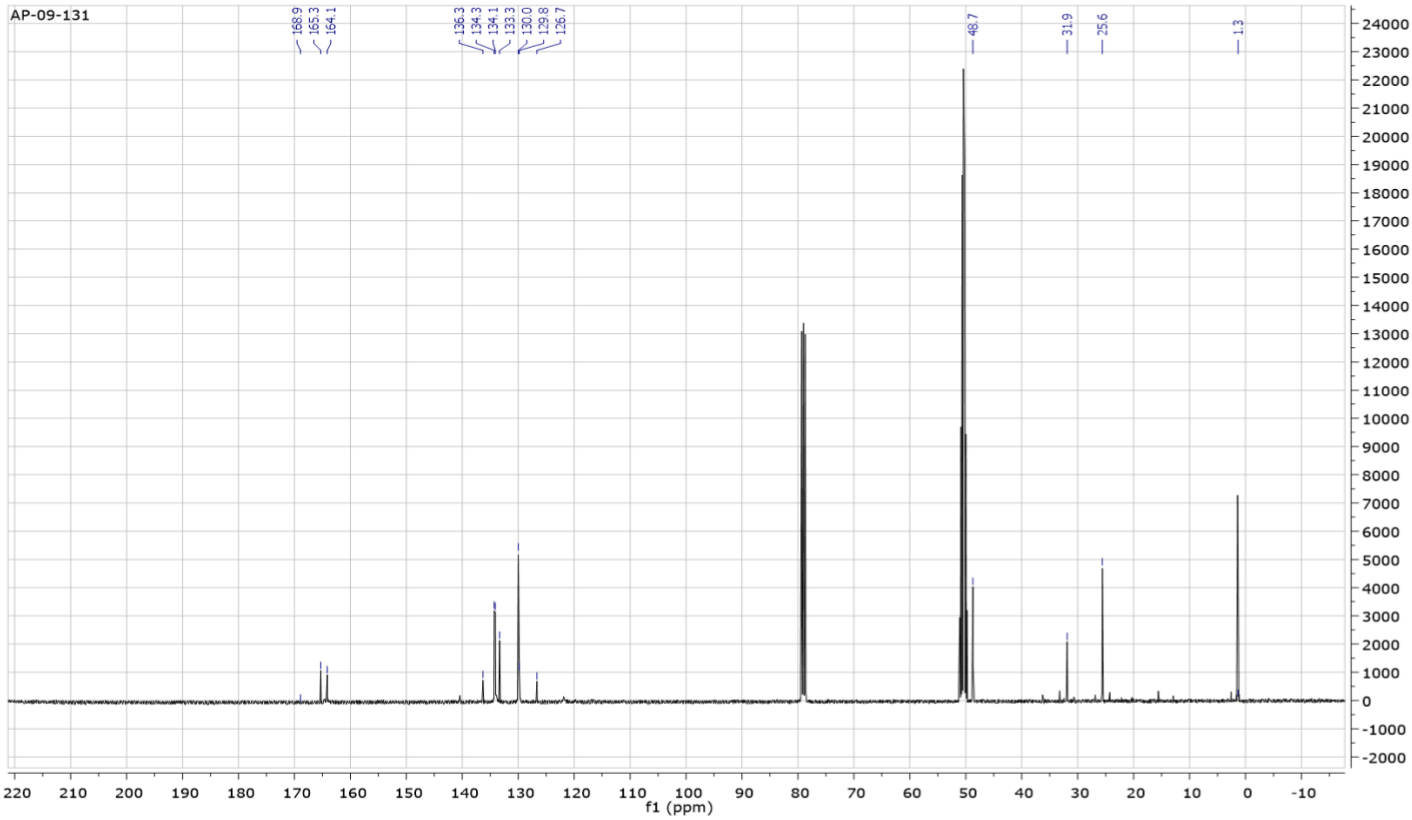

AP-09-131
168.9
165.3
164.1
136.3
134.3
134.1
133.3
130.0
129.8
126.7
48.7
31.9
25.6
1.3
f1 (ppm)

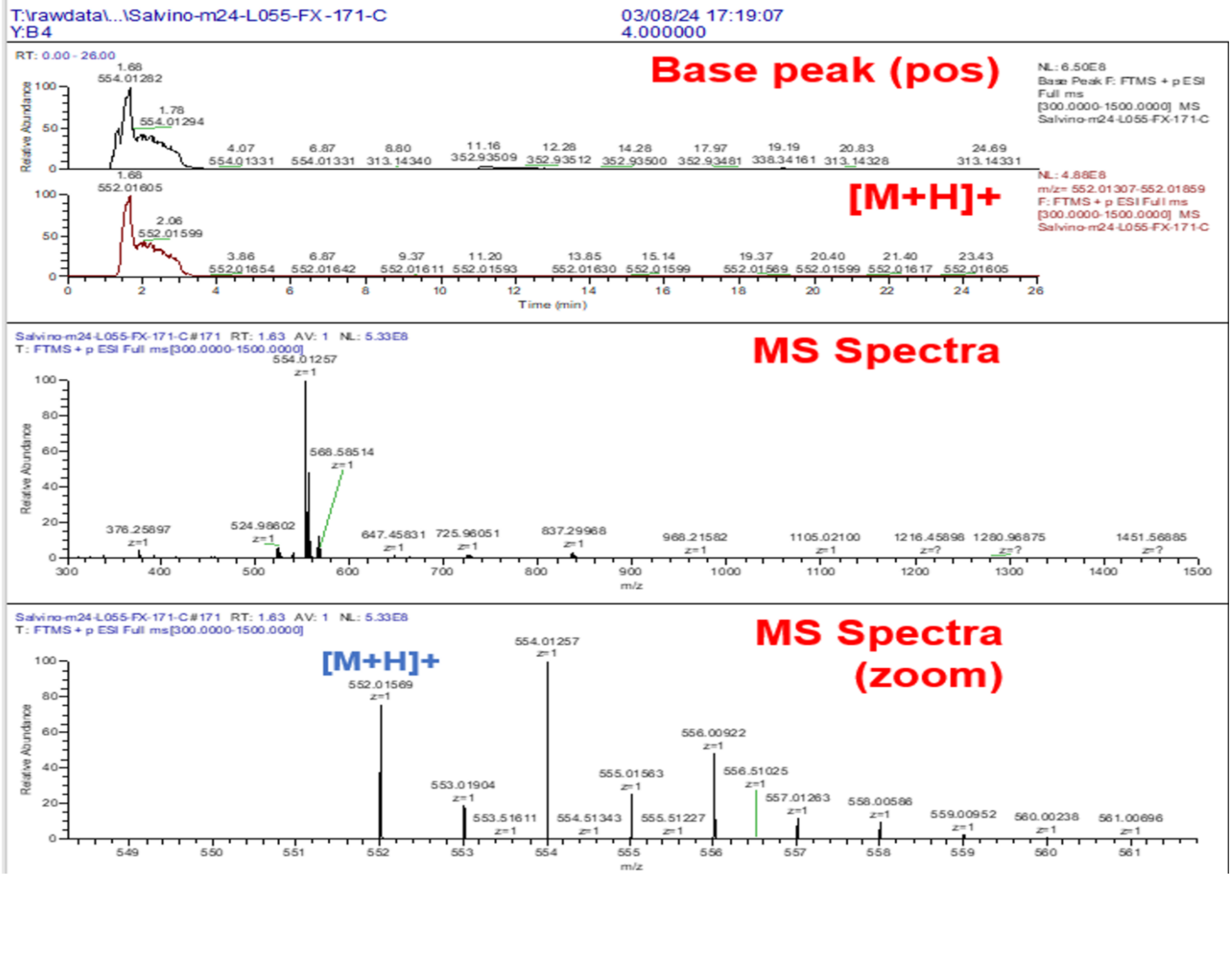

T:\rawdata\...\Salvino-m24-L055-FX-171-C
Y:B4
03/08/24 17:19:07
4.000000
RT: 0.00 - 26.00
Base peak (pos)
NL: 6.50E8
Base Peak F: FTMS + p ESI Full ms [300.0000-1500.0000] MS Salvino-m24-L055-FX-171-C
[M+H]+
NL: 4.88E8
m/z= 552.01307-552.01859
F: FTMS + p ESI Full ms [300.0000-1500.0000] MS Salvino-m24-L055-FX-171-C
Relative Abundance
Time (min)
Salvino-m24-L055-FX-171-C#171 RT: 1.63 AV: 1 NL: 5.33E8
T: FTMS + p ESI Full ms[300.0000-1500.0000]
MS Spectra
554.01257
568.58514
376.25897
524.98602
647.45831
725.96051
837.29968
968.21582
1105.02100
1216.45898
1280.96875
1451.56885
m/z
MS Spectra (zoom)
[M+H]+
552.01569
553.01904
553.51611
554.51343
555.01563
555.51227
556.00922
556.51025
557.01263
558.00586
559.00952
560.00238
561.00696

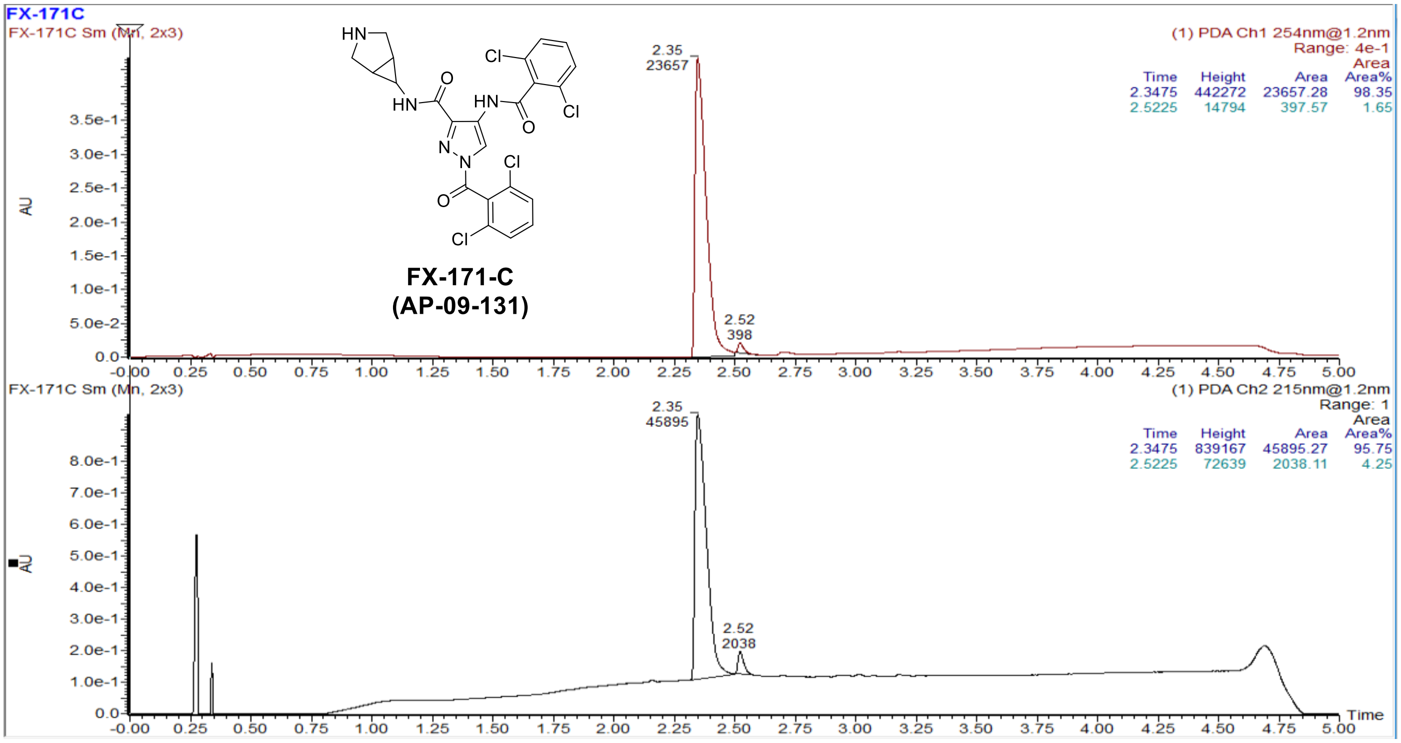

Tert-butyl 4-(4-nitro-1H-pyrazole-3-carboxamido)piperidine-1-carboxylate: AP-09-090B (AP-10-176)

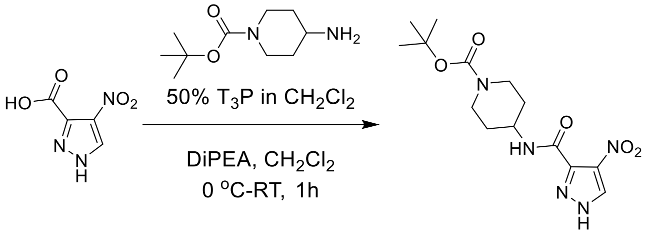

AP-09-090B
(AP-10-176)

To a stirred solution of 4-nitro-1H-pyrazole-3-carboxylic acid (1.0 g; 6.36 mmol) and tert-butyl 4-aminopiperidine-1-carboxylate (1.401 g; 7.0 mmol) in 20 mL of dry CH_2Cl_2 at 0 °C was added diisopropyl ethyl amine (4.43 mL; 25.46 mmol) and T3P 50% solution in CH_2Cl_2 by weight (4.858 g; 7.64 mmol) simultaneously dropwise. The reaction mixture was stirred for an additional 1 hour at 0 °C. Completion of the reaction was confirmed by LC−MS. The product was extracted with CH_2Cl_2 (10 mL X 2) and washed with 1N *aq* HCl (20 mL), saturated *aq* $NaHCO_3$ (20 mL), brine solution (20 mL) and dried over anhydrous Na_2SO_4. The solvent was evaporated under reduced pressure to yield the crude product, which was purified by flash column chromatography to afford the title compound as a white solid (1.685 g; 4.97 mmol, 78 %). The product was confirmed by ^{1}H, ^{13}C NMR and HRMS.

^{1}H NMR (400 MHz, CD_3OD) *δ* 8.53 (s, 1H), 4.16 – 4.09 (m, 1H), 4.09 – 4.00 (d, 2H), 3.11 – 2.94 (s, 2H), 2.05 – 1.95 (m, 2H), 1.57 – 1.49 (m, 2H), 1.48 (s, 9H).

^{13}C NMR (101 MHz, DMSO) δ 159.6, 154.3, 142.0, 132.7, 131.7, 79.0, 75.3, 46.6, 43.0, 42.2, 31.3, 28.2.

HRMS: Calcd m/z for $C_{14}H_{22}N_5O_5^+$ [M + H]$^+$, 340.1615; found, 340.1610.

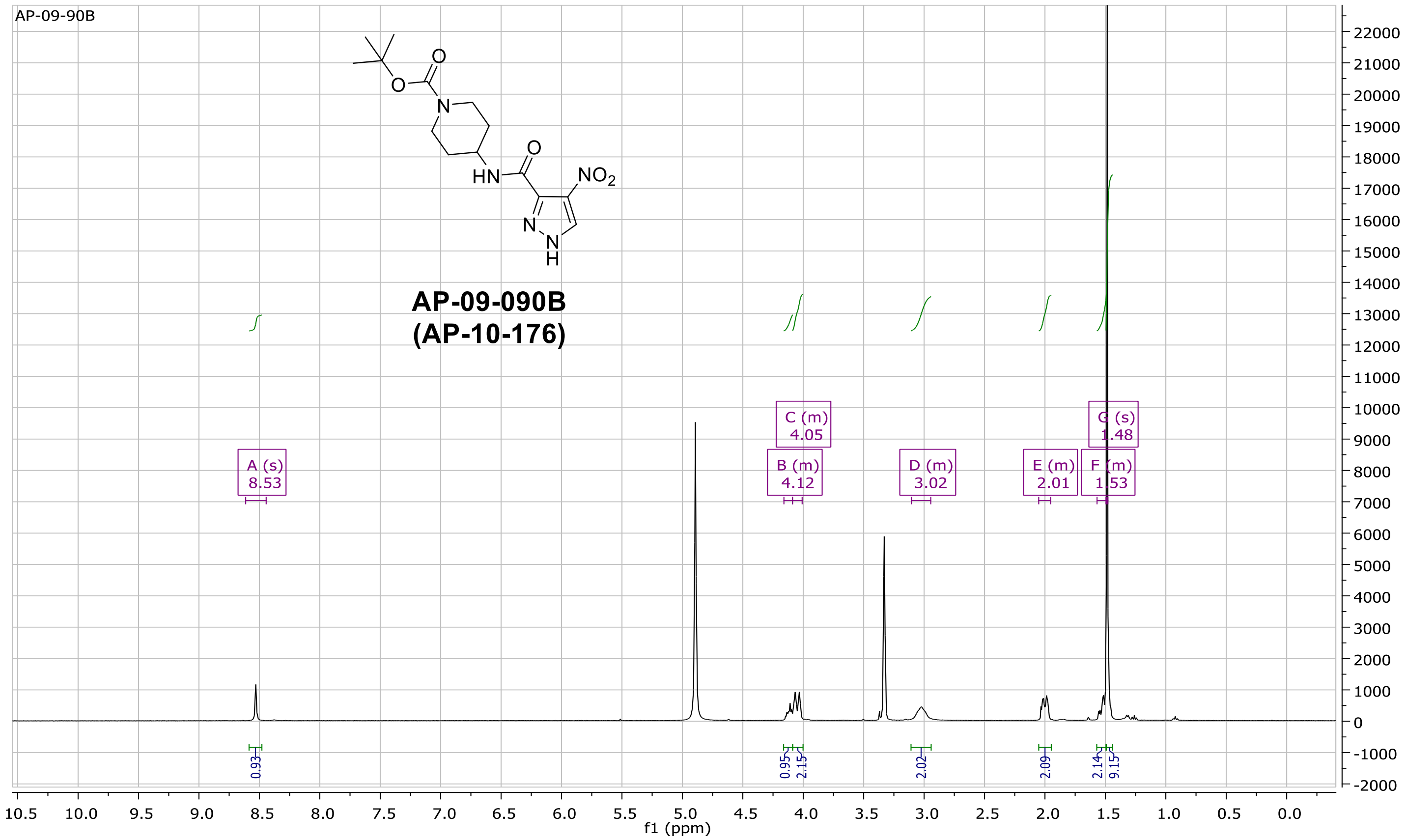

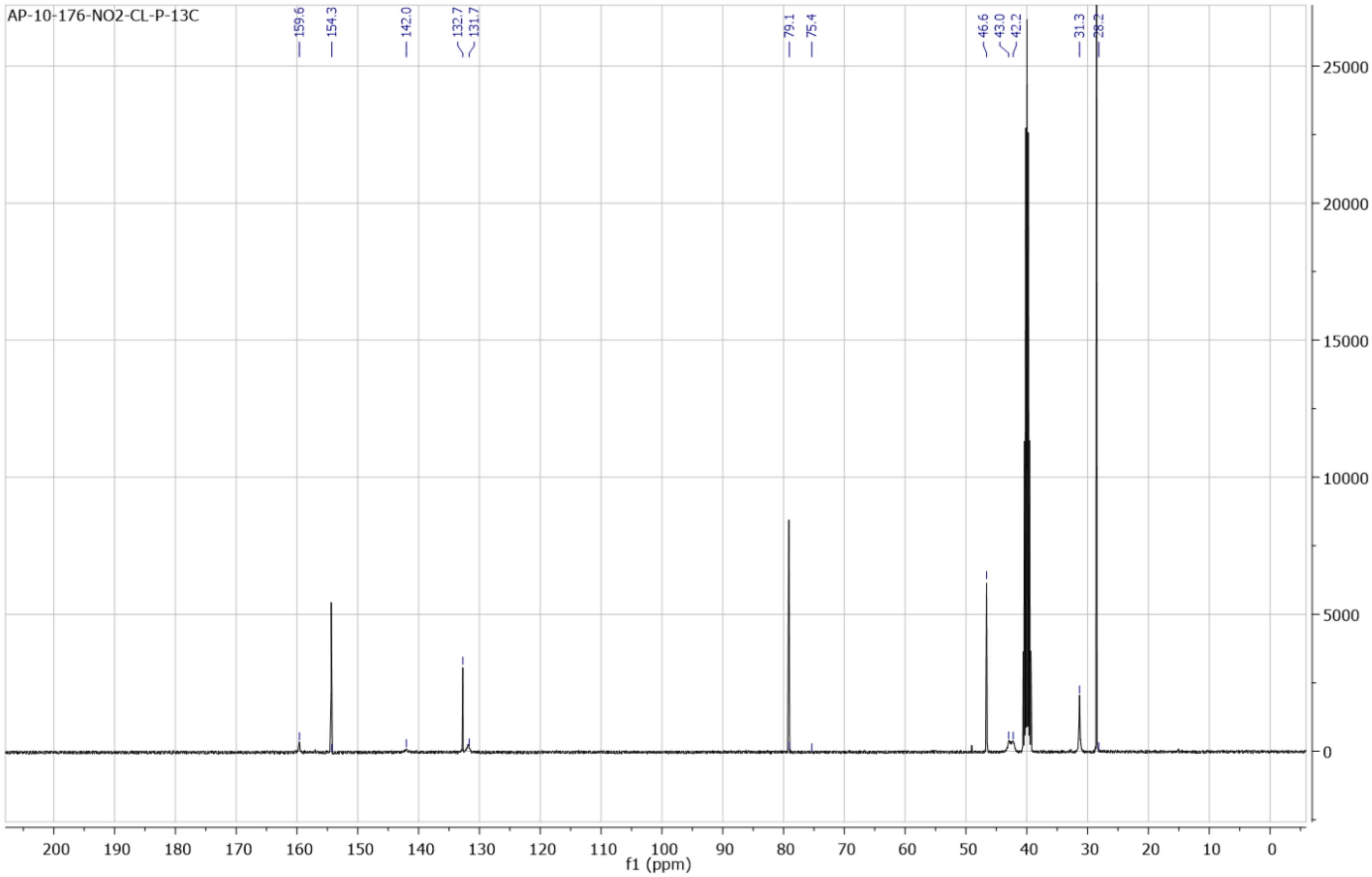
AP-10-176-NO2-CL-P-13C
159.6
154.3
142.0
132.7
131.7
79.1
75.4
46.6
43.0
42.2
31.3
28.2
25000
20000
15000
10000
5000
0
200
190
180
170
160
150
140
130
120
110
100
90
80
70
60
50
40
30
20
10
0
f1 (ppm)

AP-10-176

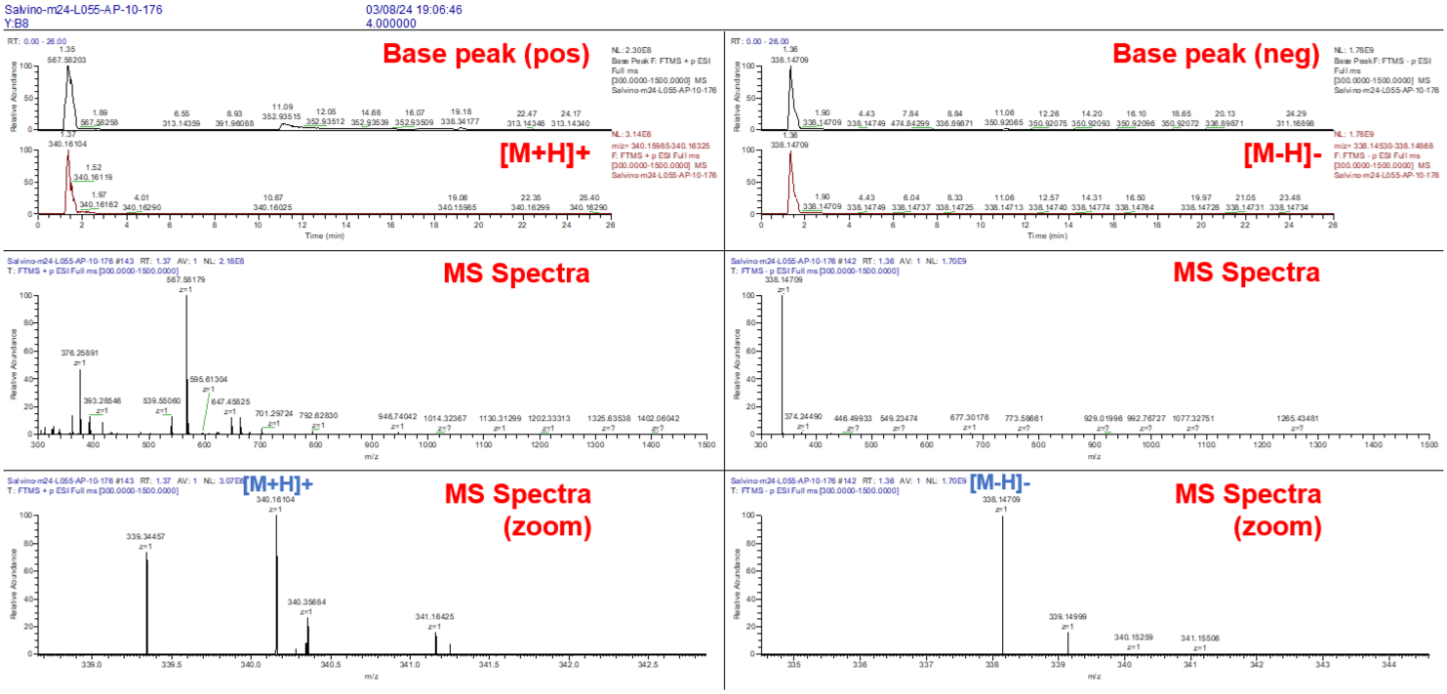
Salvino-m24-L055-AP-10-176
Y:B8
03/08/24 19:06:46
4.000000
Base peak (pos)
[M+H]+
Base peak (neg)
[M-H]-
MS Spectra
MS Spectra
[M+H]+
MS Spectra (zoom)
[M-H]-
MS Spectra (zoom)
567.58179
340.16104
339.34457
338.14709
339.14999

Tert-butyl 4-(4-amino-1H-pyrazole-3-carboxamido)piperidine-1-carboxylate: AP-09-091 (AP-10-201)

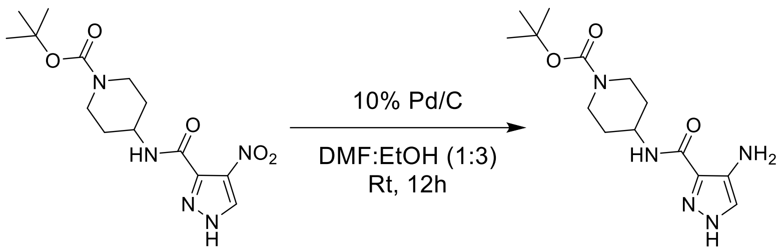

AP-09-090B (AP-10-176) **AP-09-091 (AP-10-201)**

At room temperature, a suspension of tert-butyl 4-(4-nitro-1H-pyrazole-3-carboxamido)piperidine-1-carboxylate (1.6 g, 2.27 mmol) and Pd/C (160 mg) in DMF (10 mL) and EtOH (30 mL) was stirred for 2 hours under H_2 at room temperature. Completion of the reaction was confirmed by TLC, and then the reaction mixture was filtered through Celite and the filtrate was concentrated under reduced pressure to obtain a residue which was diluted in ethyl acetate, washed with cold brine solution, and dried over anhydrous Na_2SO_4. The solvent was evaporated under reduced pressure to yield the crude product, which was purified by flash column chromatography to afford the title compound as a light brown solid (1.241 g, 4.01 mmol, 85%). The product was confirmed by ^1H, ^{13}C NMR and HRMS.

^1H NMR (400 MHz, CD_3OD) δ 7.20 (s, 1H), 5.49 (DCM), 4.08 – 4.05 (m, 1H), 4.05 – 3.96 (m, 2H), 3.03 – 2.85 (s, 2H), 2.01 (ethyl acetate), 1.96 – 1.87 (m, 2H), 1.53 – 1.42 (m, 11H), 1.24 (ethyl acetate).

^{13}C NMR (101 MHz, DMSO) δ 163.0, 154.3, 133.7, 125.3, 125.2, 79.0, 45.8, 31.9, 28.6.

HRMS: Calcd m/z for $C_{14}H_{24}N_5O_3^+$ [M + H] $^+$, 310.1874; found, 310.1872.

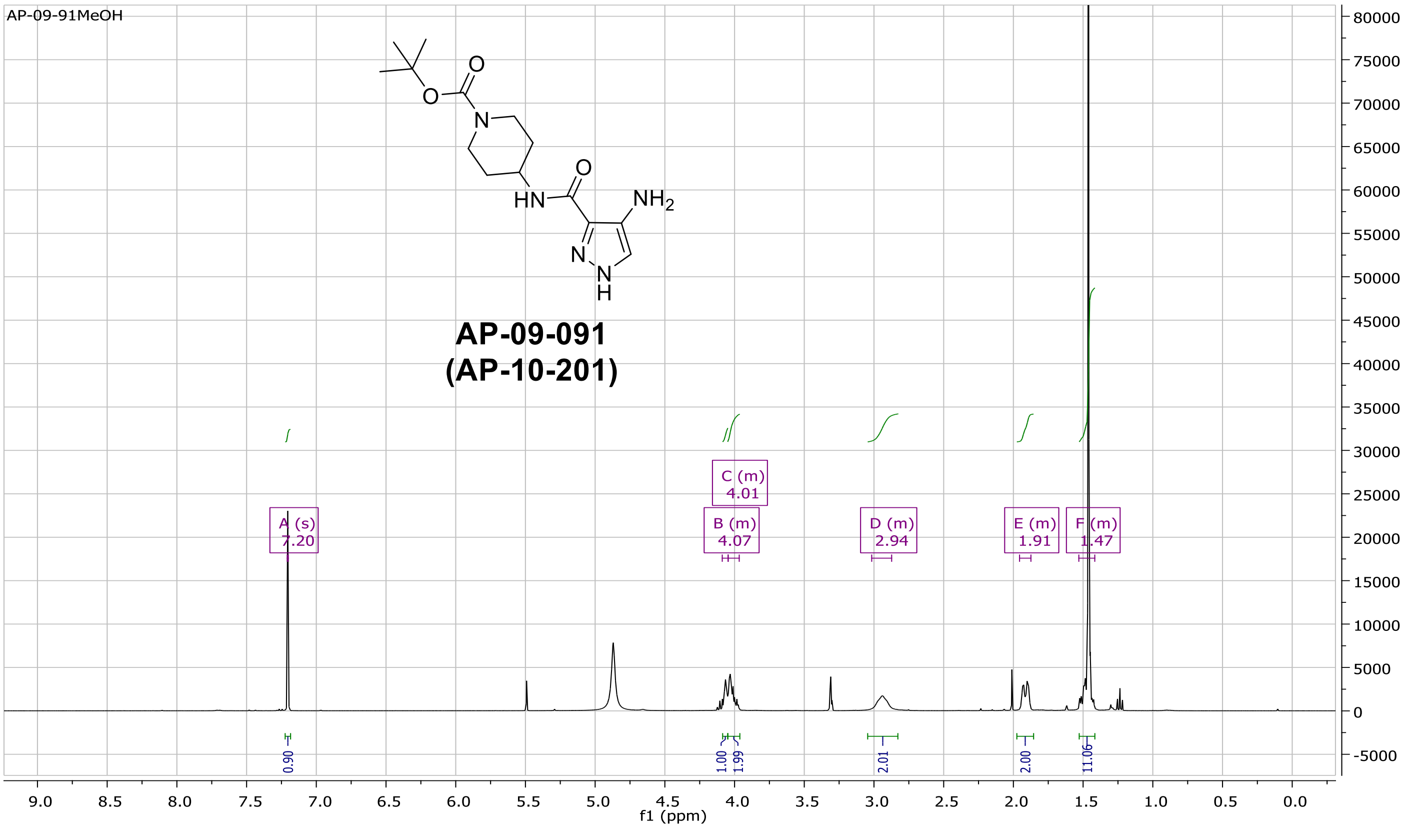

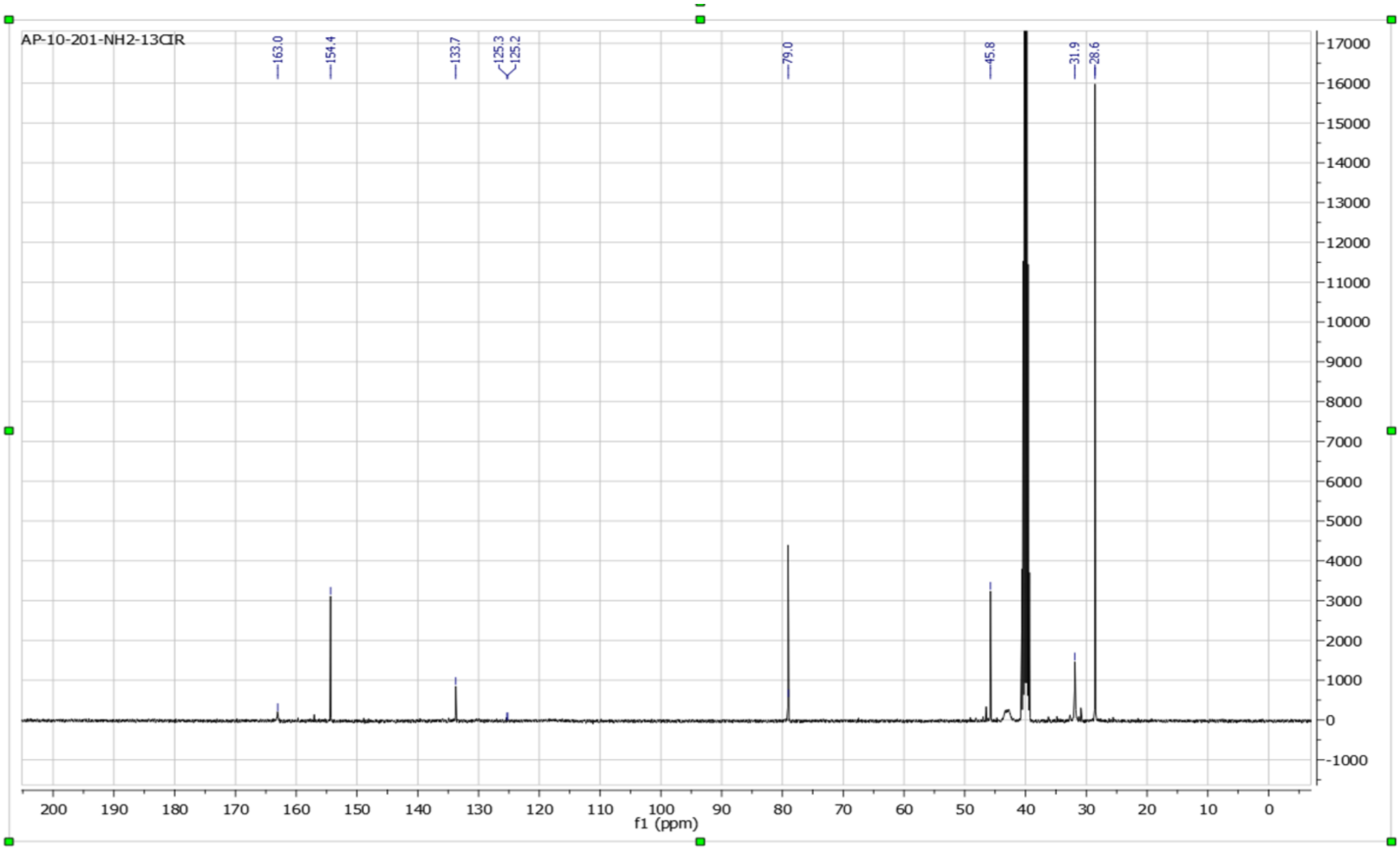
AP-10-201-NH2-13CIR
163.0
154.4
133.7
125.3
125.2
79.0
45.8
31.9
28.6
f1 (ppm)

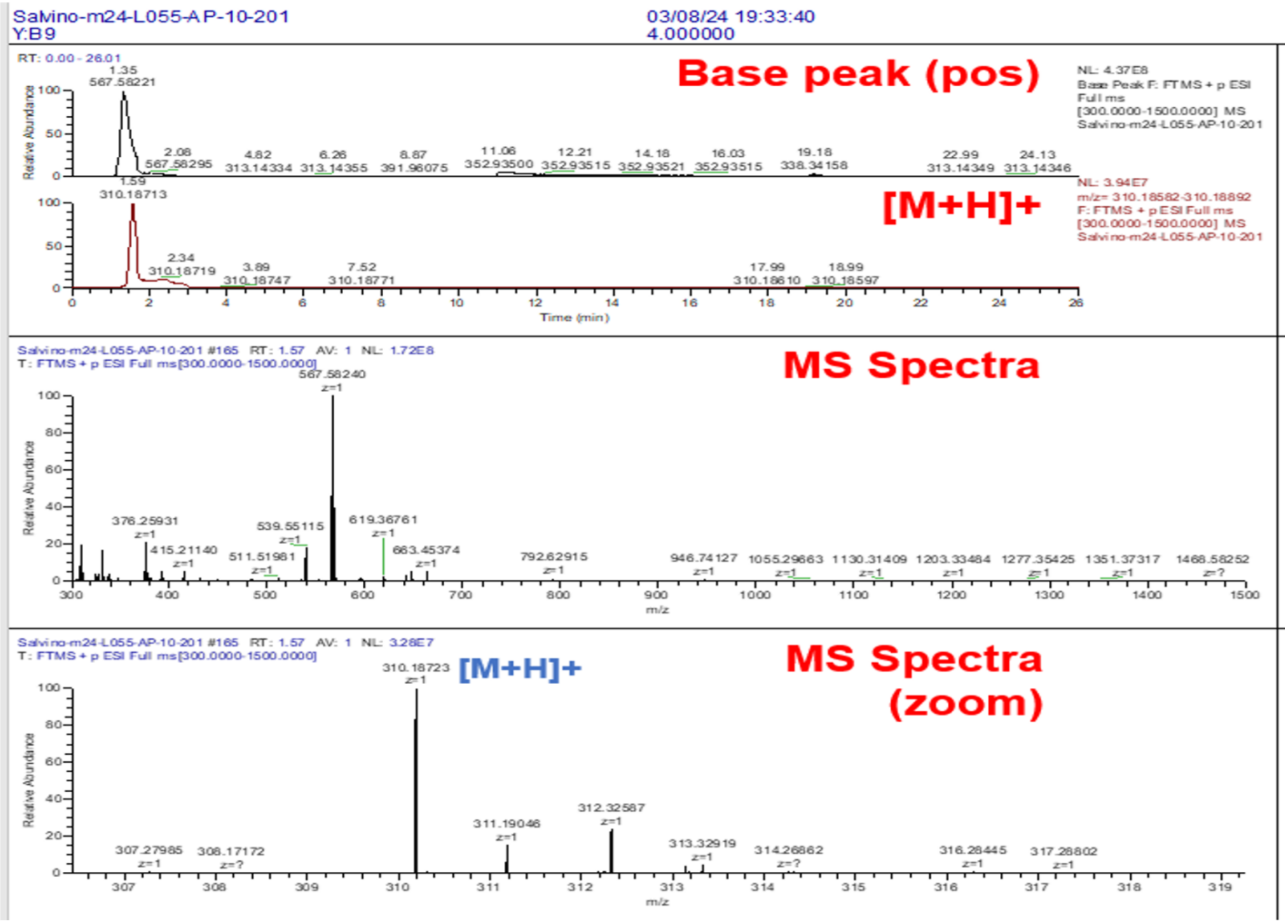
Salvino-m24-L055-AP-10-201
Y:B9
03/08/24 19:33:40
4.000000
Base peak (pos)
NL: 4.37E8
Base Peak F: FTMS + p ESI Full ms [300.0000-1500.0000] MS Salvino-m24-L055-AP-10-201
[M+H]+
NL: 3.94E7
m/z= 310.18582-310.18892
F: FTMS + p ESI Full ms [300.0000-1500.0000] MS Salvino-m24-L055-AP-10-201
Time (min)
MS Spectra
Salvino-m24-L055-AP-10-201 #165 RT: 1.57 AV: 1 NL: 1.72E8
T: FTMS + p ESI Full ms[300.0000-1500.0000]
567.58240
m/z
MS Spectra (zoom)
Salvino-m24-L055-AP-10-201 #165 RT: 1.57 AV: 1 NL: 3.28E7
T: FTMS + p ESI Full ms[300.0000-1500.0000]
310.18723
[M+H]+
311.19046
312.32587
m/z

Tert-butyl 4-(4-(2,6-dichlorobenzamido)-1-(2,6-dichlorobenzoyl)-1H-pyrazole-3-carboxamido) piperidine -1-carboxylate: AP-09-120B (AP-10-202)

Tert-butyl 4-(4-(2,6-dichlorobenzamido)-1H-pyrazole-3-carboxamido)piperidine-1-carboxylate (AP-10-202A):

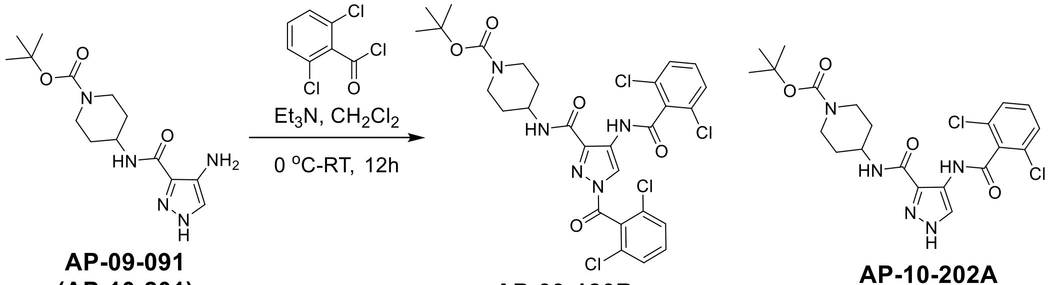

AP-09-091 (AP-10-201) **AP-09-120B (AP-10-202)** **AP-10-202A**

To a stirred solution of tert-butyl 4-(4-amino-1H-pyrazole-3-carboxamido)piperidine-1-carboxylate (300 mg; 0.97 mmol) in 15 mL of dry CH_2Cl_2 at 0 °C was added triethyl amine (0.7 mL; 4.85 mmol) and 2,6-dichlorobenzoyl chloride (608 mg; 2.91 mmol) simultaneously dropwise. The reaction mixture was slowly brought to room temperature and stirred for 16 hours. Completion of the reaction was confirmed by LC−MS. The reaction mixture was quenched with 10 mL of cold water. The product was extracted with CH_2Cl_2 and washed with 1N *aq* HCl (10 mL), saturated *aq* $NaHCO_3$ (10 mL), brine solution (10 mL) and dried over anhydrous Na_2SO_4. The solvent was evaporated under reduced pressure to yield the crude product, which was purified by flash column chromatography to afford AP-10-202 as a white solid (100 mg; 0.15 mmol) and AP-10-202A as a white solid (202 mg; 42 mmol). The products were confirmed by ^1H, ^{13}C NMR and HRMS.

^1H NMR (400 MHz, CD_3OD) *δ* 8.99 (s, 1H), 7.52 – 7.43 (m, 3H), 7.43 – 7.34 (m, 3H), 4.00 – 3.90 (m, 2H), 3.90 – 3.82 (m, 1H), 2.80 – 2.60 (m, 2H), 1.77-1.64 (m, 2H), 1.43 – 1.35 (m, 2H), 1.33 (s, 9H), 1.27 – 1.12 (pentane), 0.80 (pentane).

^{13}C NMR (101 MHz, CDCl3) δ 162.0, 161.7, 154.5, 134.9, 132.7, 132.5, 131.3, 128.2, 128.0, 125.1, 79.8, 47.1, 42.5, 31.7, 28.4.

HRMS: Calcd m/z for $C_{28}H_{28}Cl_4N_5O_5^+$ [M + H] $^+$, 654.0839; found, 654.0838.

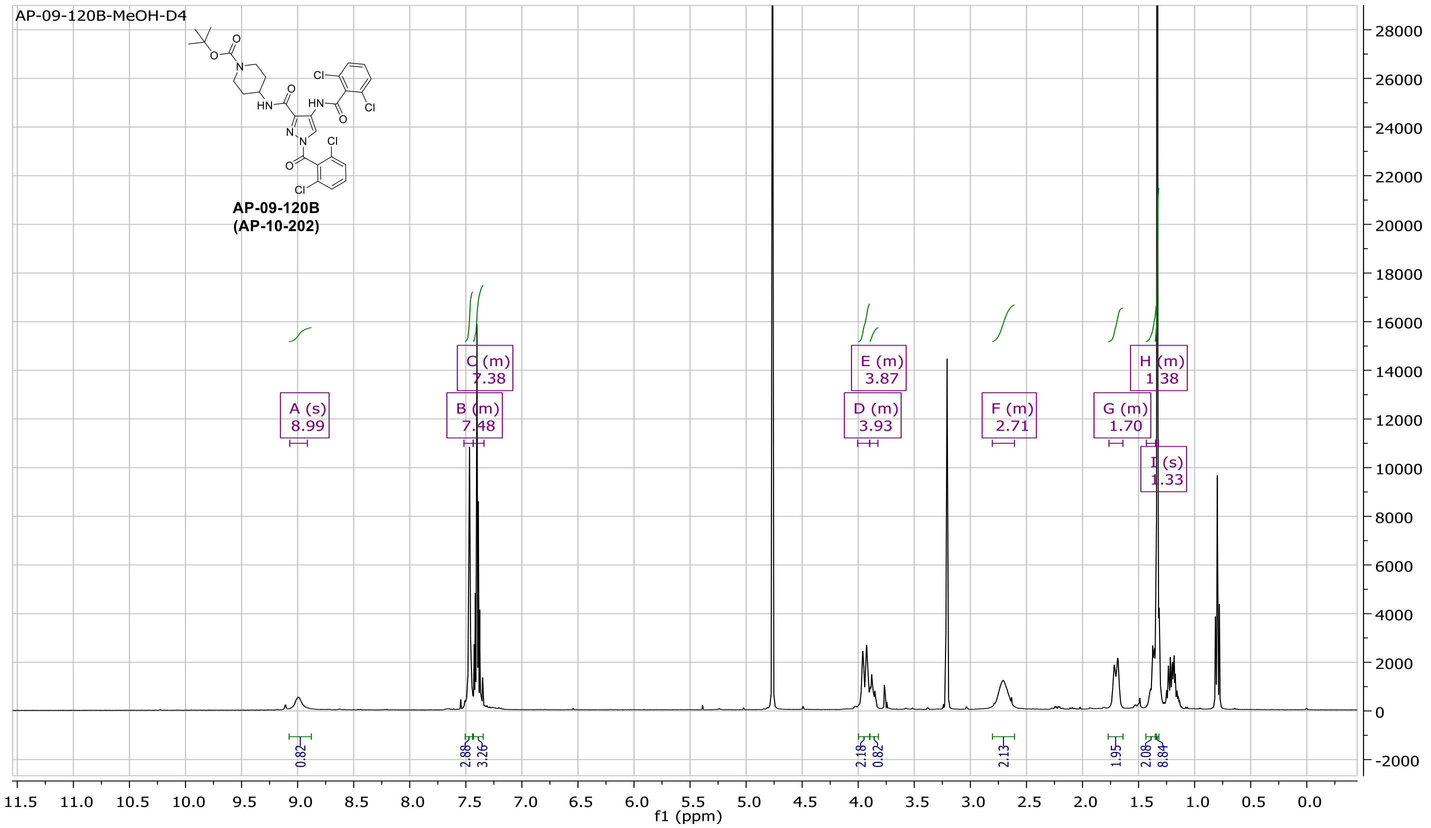
AP-09-120B-MeOH-D4
AP-09-120B
(AP-10-202)
A (s) 8.99
B (m) 7.48
C (m) 7.38
D (m) 3.93
E (m) 3.87
F (m) 2.71
G (m) 1.70
H (m) 1.38
I (s) 1.33
0.82
2.88
3.26
2.18
0.82
2.13
1.95
2.08
8.84
f1 (ppm)

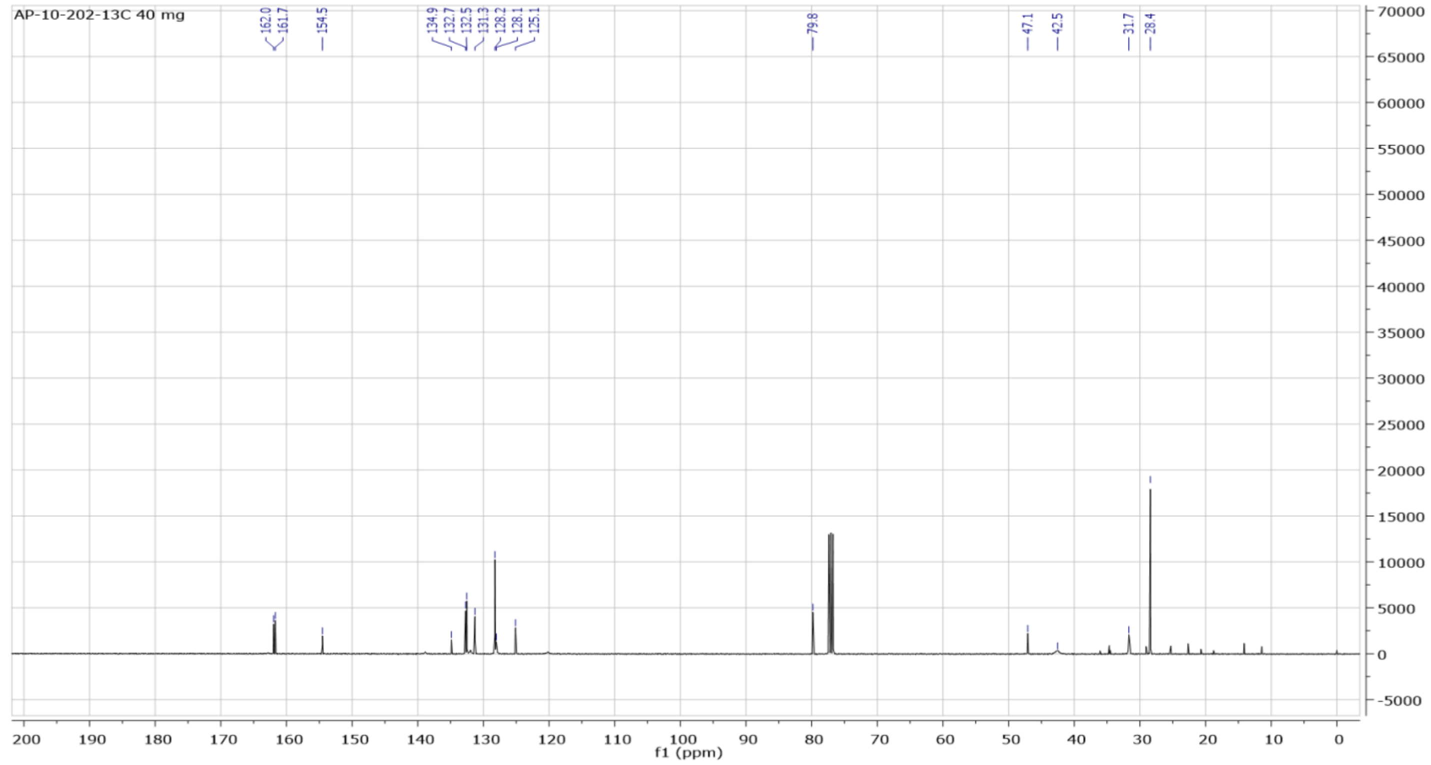
AP-10-202-13C 40 mg
162.0
161.7
154.5
134.9
132.7
132.5
131.3
128.2
128.1
125.1
79.8
47.1
42.5
31.7
28.4
f1 (ppm)

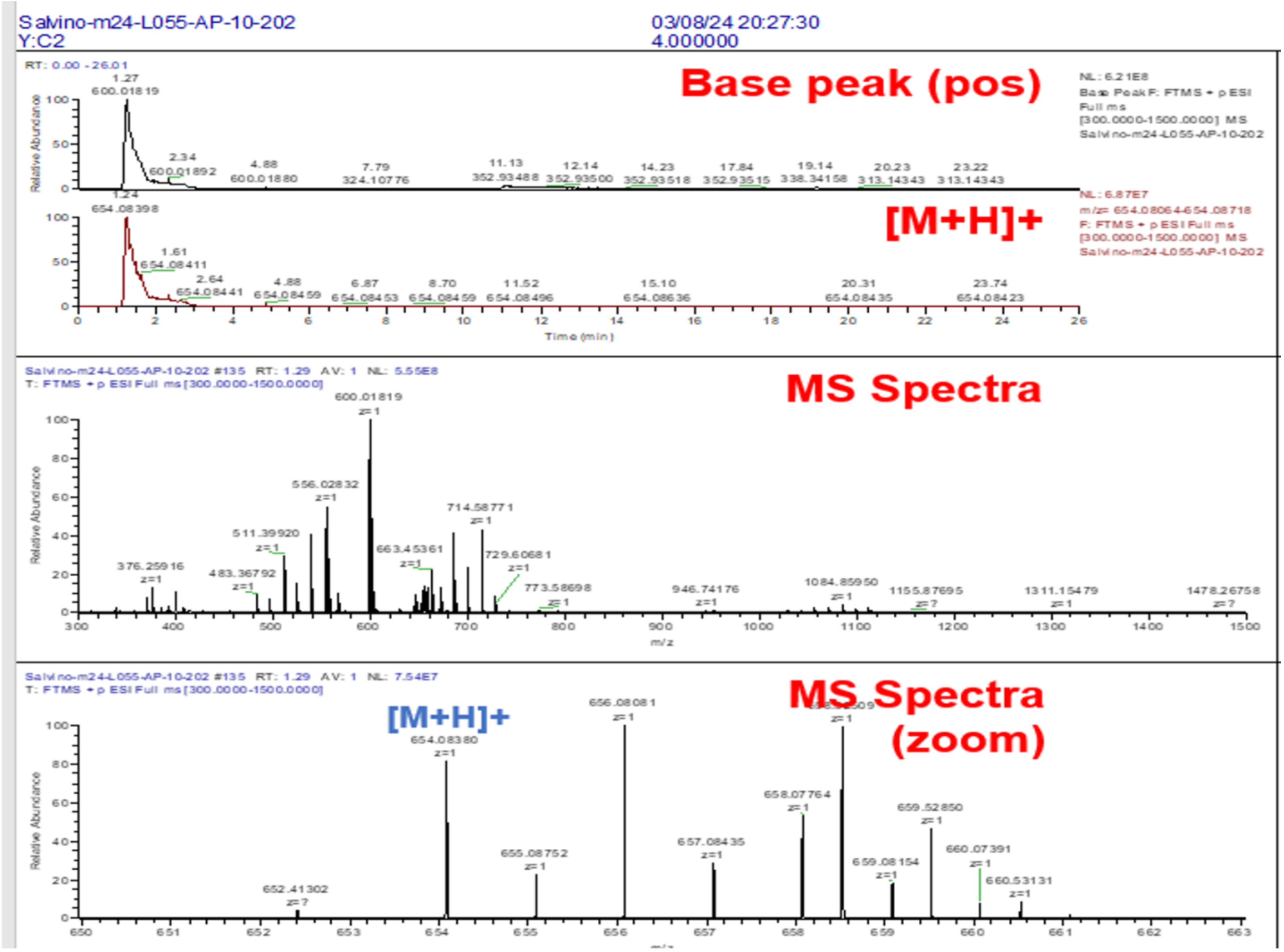

Tert-butyl 4-(4-(2,6-dichlorobenzamido)-1H-pyrazole-3-carboxamido)piperidine-1-carboxylate (AP-10-202A):

^{1}H NMR (400 MHz, DMSO) δ 13.40 (d, *J* = 22.3 Hz, 1H), 10.18 (s, 1H), 8.42 (d, *J* = 8.4 Hz, 1H), 8.36 (d, *J* = 1.3 Hz, 1H), 7.62 – 7.50 (m, 10H), 4.04 – 3.81 (m, 11H), 3.32 (s, 1H), 2.71 (d, J = 23.7 Hz, 8H), 1.75 – 1.61 (m, 7H), 1.59 – 1.42 (m, 8H), 1.40 (s, 29H).

^{13}C NMR (101 MHz, DMSO) δ 163.1, 160.7, 154.3, 135.8, 133.3, 132.3, 131.7, 128.9, 122.0, 121.7, 79.1, 46.3, 31.5, 28.5.

HRMS: Calcd m/z for $C_{21}H_{26}Cl_2N_5O_4^+$ [M + H] $^+$, 482.1356; found, 482.1355.

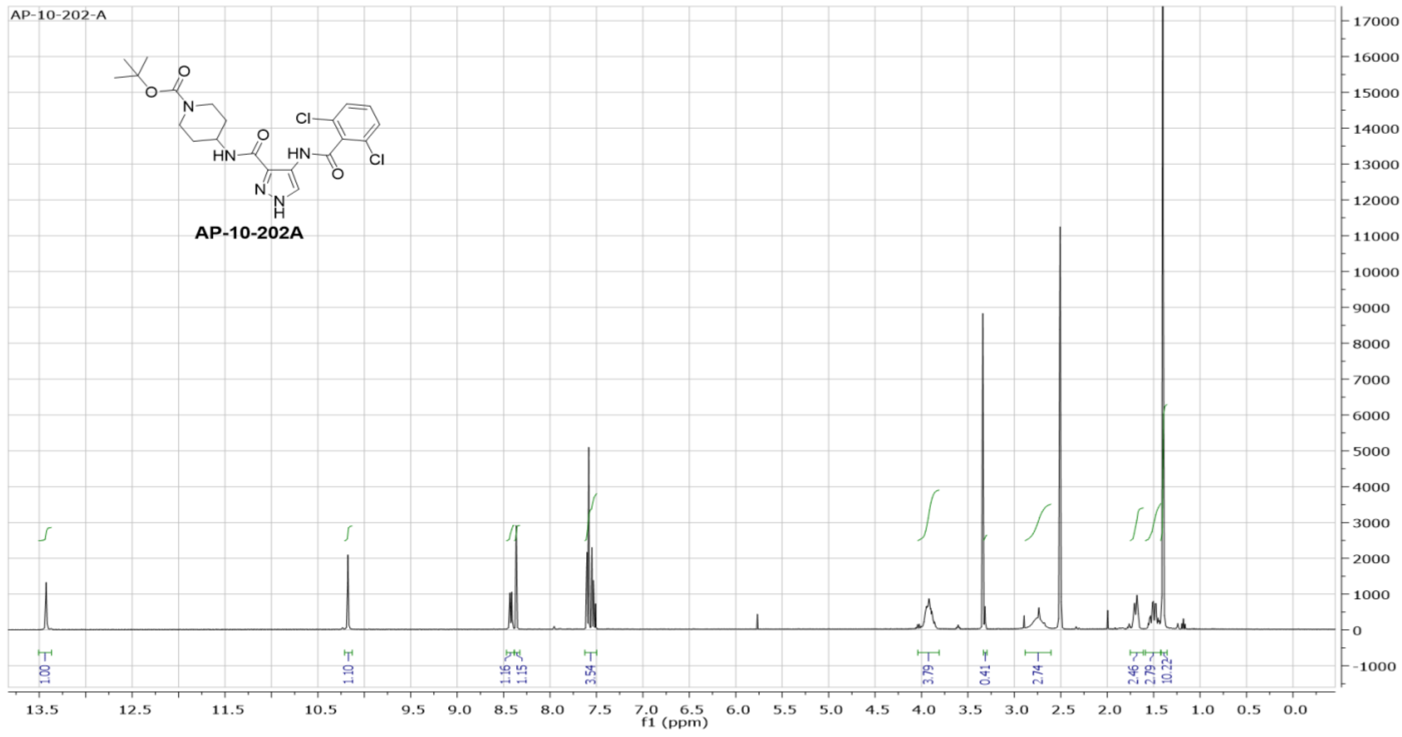
AP-10-202-A
AP-10-202A
1.00
1.10
1.16
1.15
3.54
3.79
0.41
2.74
2.46
2.79
10.22
f1 (ppm)

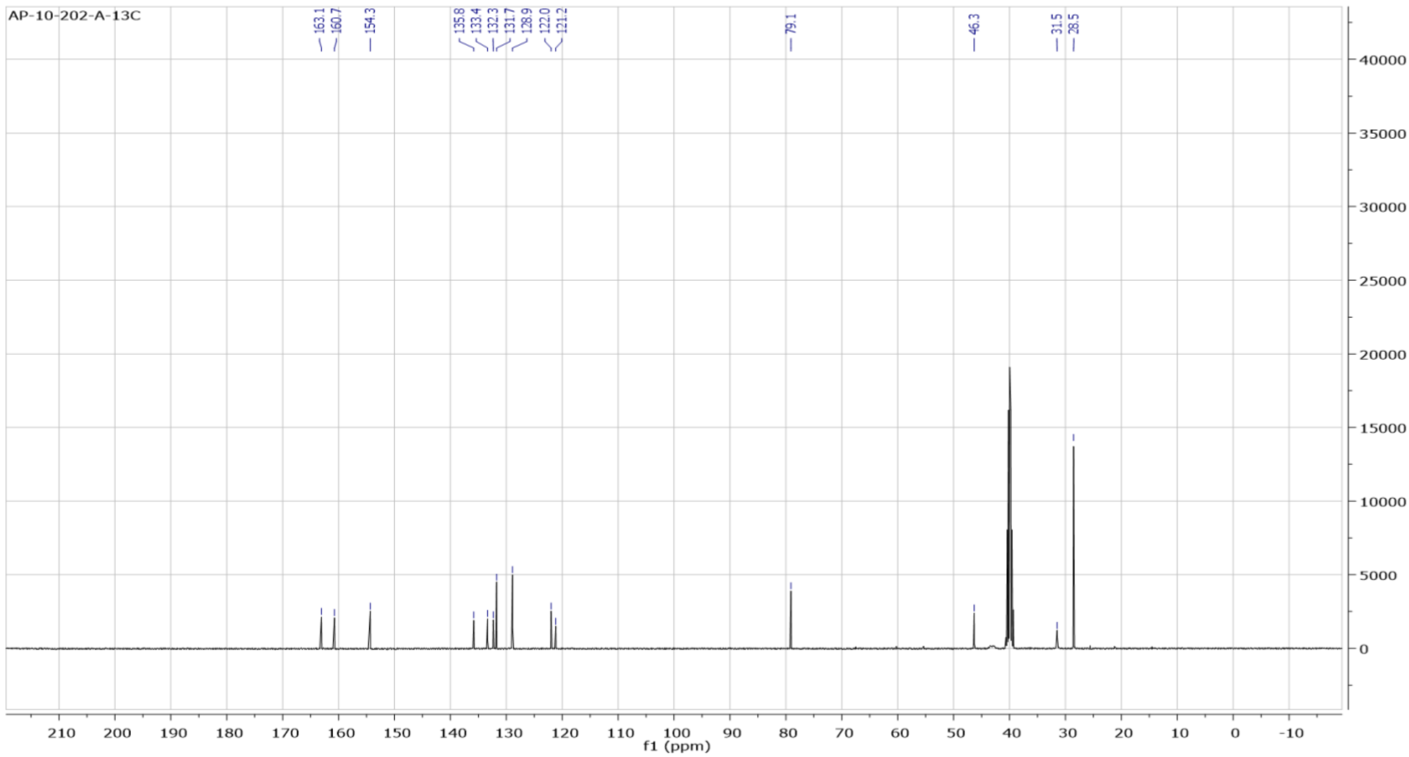
AP-10-202-A-13C
163.1
160.7
154.3
135.8
133.4
132.3
131.7
128.9
122.0
121.2
79.1
46.3
31.5
28.5
f1 (ppm)

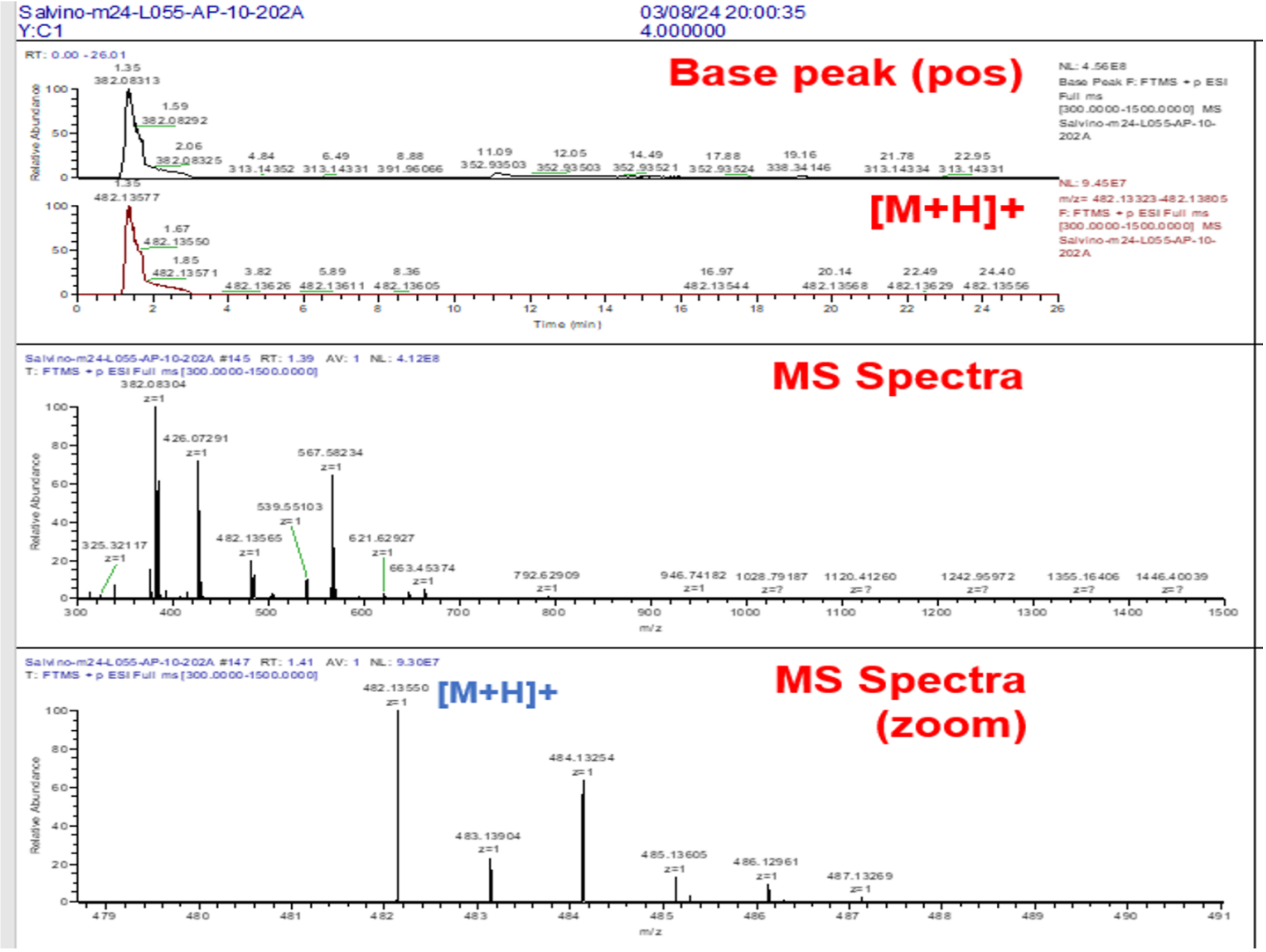

4-(2,6-dichlorobenzamido)-1-(2,6-dichlorobenzoyl)-N-(piperidin-4-yl)-1H-pyrazole-3-carboxamide: JMS-175-2 (AP-09-126)

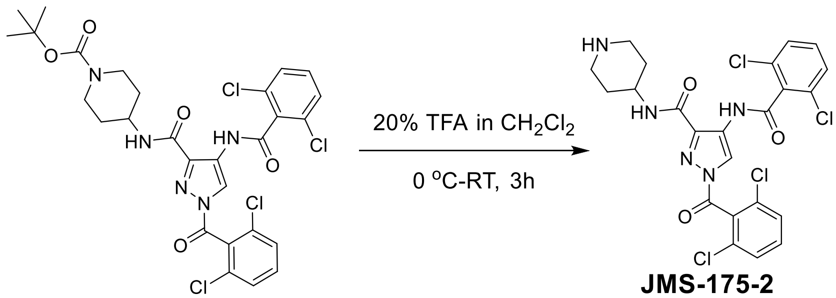

JMS-175-2 (AP-09-126)

AP-09-120B (AP-10-202)

To a stirred solution of tert-butyl 4-(4-(2,6-dichlorobenzamido)-1-(2,6-dichlorobenzoyl)-1H-pyrazole-3-carboxamido)piperidine-1-carboxylate (100 mg; 0.152 mmol) in 4 mL CH_2Cl_2 at 0 °C was added 1 mL of TFA. The reaction mixture was then warmed to room temperature and stirred for 3 hours. Completion of the reaction was confirmed by LC−MS. Volatiles were evaporated under reduced pressure to yield the crude product which was purified by flash column chromatography to afford the title compound as a white solid (76 mg; 0.14 mmol, 90%) which was confirmed by ^{1}H, ^{13}C NMR and HRMS.

^{1}H NMR (400 MHz, CD_3OD) *δ* 9.00 (s, 1H), 7.49 (s, 3H), 7.45 – 7.28 (m, 3H), 4.07 – 3.95 (m, 1H), 3.36 – 3.26 (m, 2H), 3.07 – 2.85 (m, 2H), 2.05 – 1.94 (m, 2H), 1.79 – 1.65 (m, 2H).

^{13}C NMR (101 MHz, CDCl3) δ 162.0, 161.9, 134.8, 132.6, 132.5, 131.4, 128.3, 128.1, 125.1, 44.2, 43.1, 28.3.

HRMS: Calcd m/z for $C_{23}H_{20}Cl_4N_5O_3^+$ [M + H] $^+$, 554.0315; found, 554.0316.

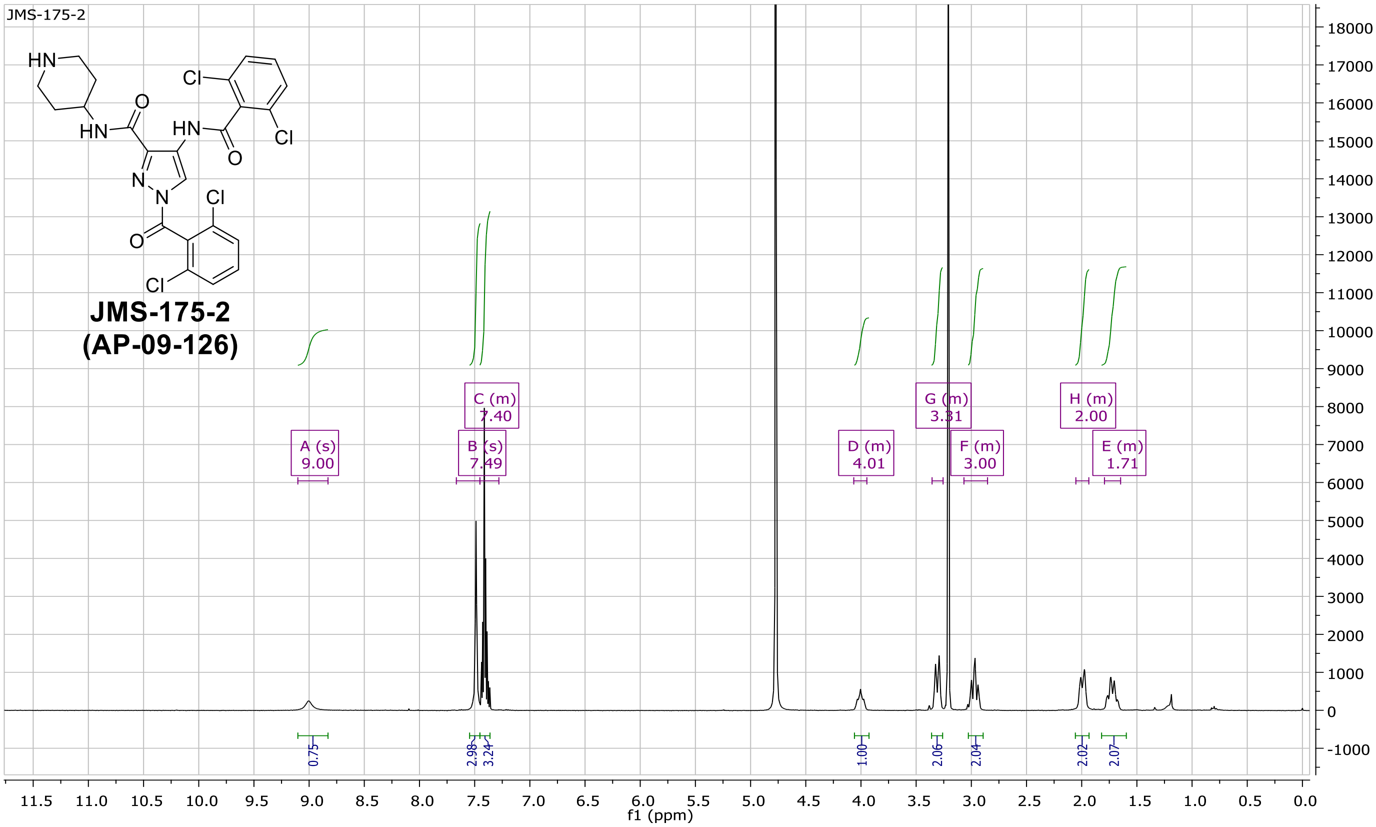

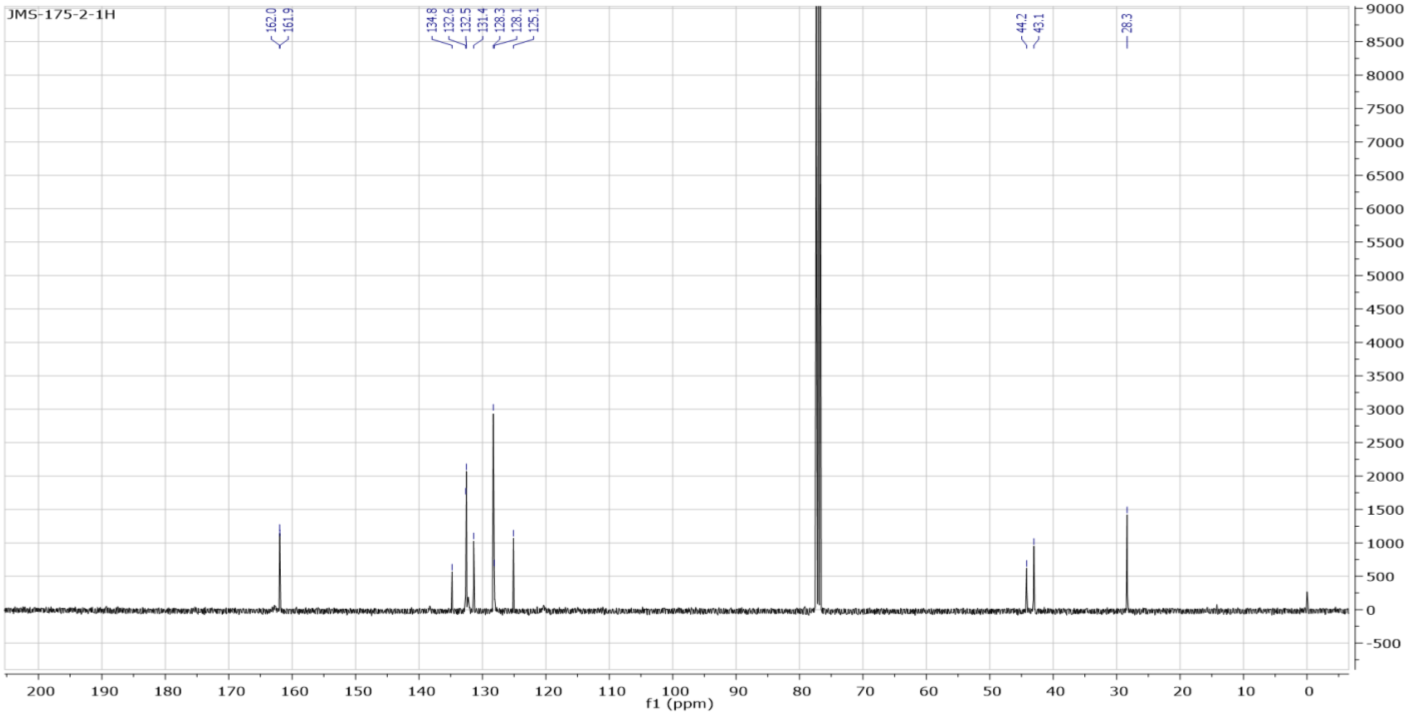

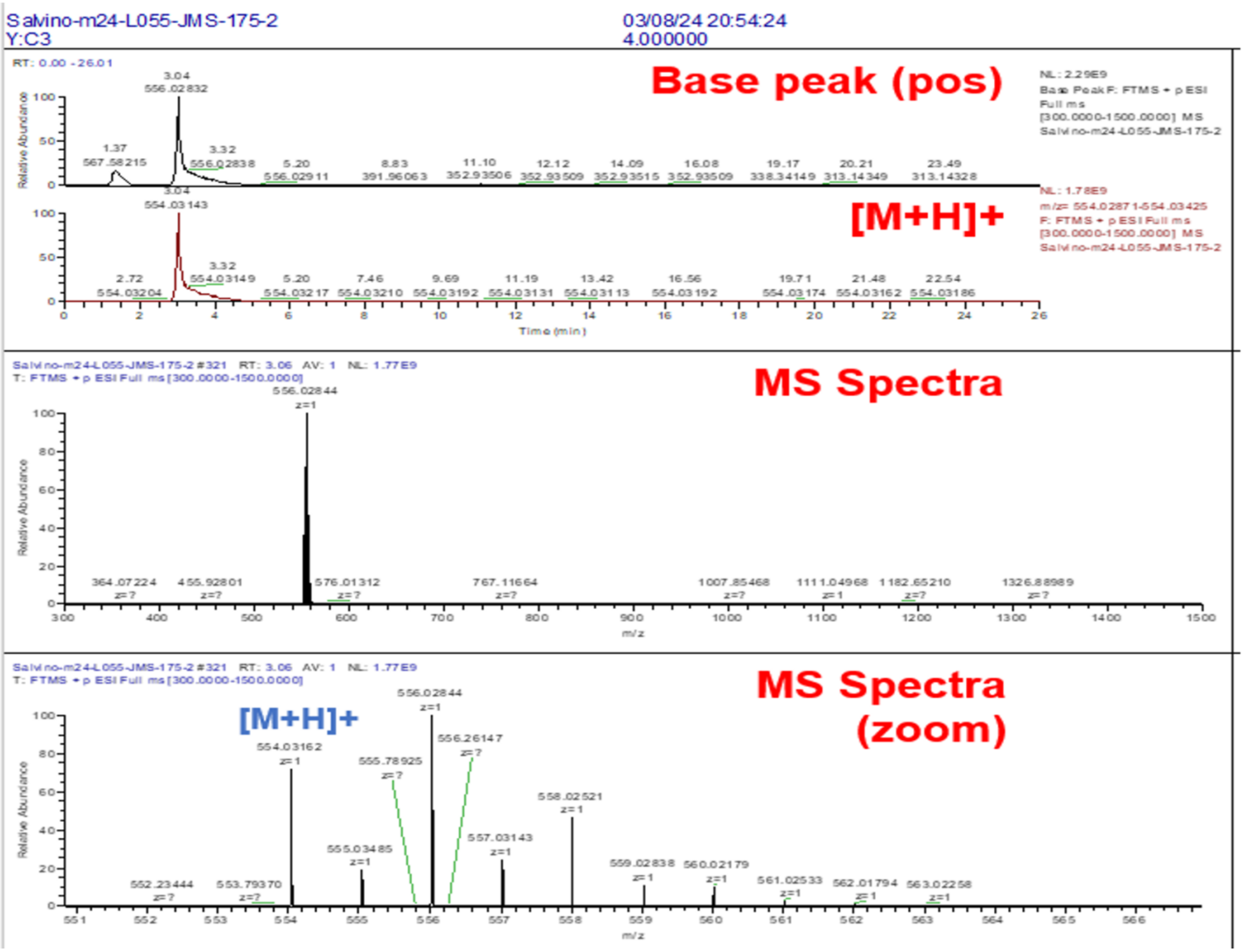
Salvino-m24-L055-JMS-175-2
Y:C3
03/08/24 20:54:24
4.000000
Base peak (pos)
NL: 2.29E9
Base Peak F: FTMS + p ESI Full ms [300.0000-1500.0000] MS Salvino-m24-L055-JMS-175-2
[M+H]+
NL: 1.78E9
m/z= 554.02871-554.03425 F: FTMS + p ESI Full ms [300.0000-1500.0000] MS Salvino-m24-L055-JMS-175-2
Time (min)
MS Spectra
Salvino-m24-L055-JMS-175-2 #321 RT: 3.06 AV: 1 NL: 1.77E9
T: FTMS + p ESI Full ms [300.0000-1500.0000]
556.02844
m/z
MS Spectra (zoom)
[M+H]+
554.03162
556.02844
558.02521
Relative Abundance

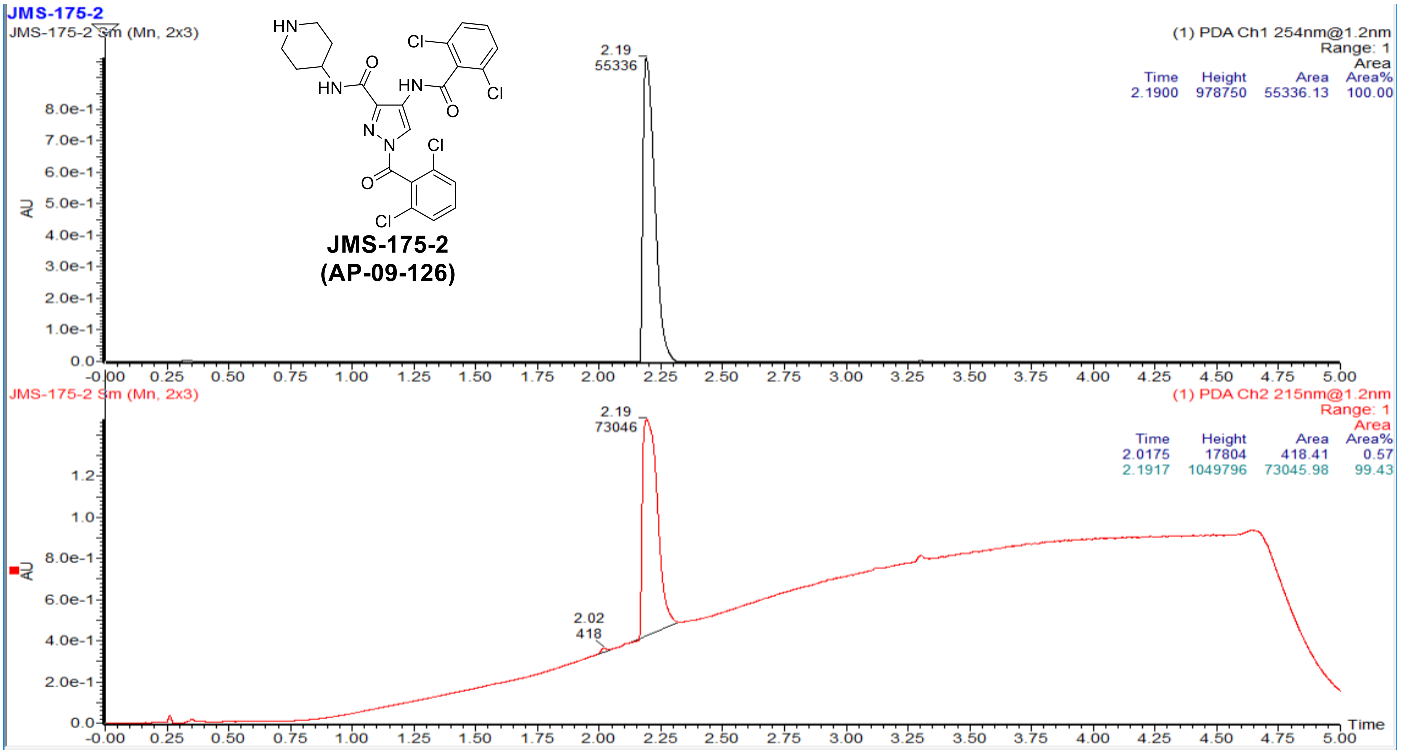
JMS-175-2
JMS-175-2 Sm (Mn, 2x3)
(1) PDA Ch1 254nm@1.2nm
Range: 1
Time Height Area Area%
2.1900 978750 55336.13 100.00
2.19
55336
JMS-175-2
(AP-09-126)
JMS-175-2 Sm (Mn, 2x3)
(1) PDA Ch2 215nm@1.2nm
Range: 1
Time Height Area Area%
2.0175 17804 418.41 0.57
2.1917 1049796 73045.98 99.43
2.19
73046
2.02
418
AU
Time

Tert-butyl 4-(N-methyl-4-nitro-1H-pyrazole-3-carboxamido)piperidine-1-carboxylate: (AP-10-177)

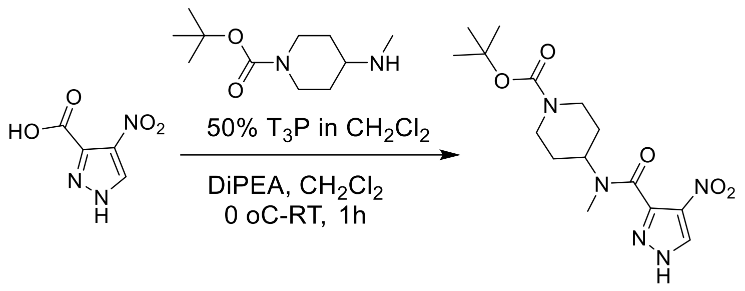

AP-10-177

To a stirred solution of 4-nitro-1H-pyrazole-3-carboxylic acid (1.0 g; 6.36 mmol) and tert-butyl 4-(methylamino)piperidine-1-carboxylate (1.5 g; 7.00 mmol) in 20 mL of dry CH_2Cl_2 at 0 °C was added diisopropyl ethyl amine (4.56 mL; 25.44 mmol) and T3P 50% solution in CH_2Cl_2 by weight (5.26 g; 8.23 mmol) simultaneously dropwise. The reaction mixture was stirred for an additional 1 hour at 0 °C. Completion of the reaction was confirmed by LC−MS. The product was extracted with CH_2Cl_2 (10 mL X 2) and washed with 1N *aq* HCl (20 mL), saturated *aq* $NaHCO_3$ (20 mL), brine solution (20 mL) and dried over anhydrous Na_2SO_4. The solvent was evaporated under reduced pressure to yield the crude product, which was purified by flash column chromatography to afford the title compound as a white solid (1.9 g; 4.90 mmol, 77%) which was confirmed by ^1H, ^{13}C NMR and HRMS.

(Mixture of Isomers) ^1H NMR (400 MHz, CDCl3) δ 13.17 (m, 1H) 8.19-8.10 (m, 2H), 4.83 (m, 1H), 4.42-4.02 (m,3H), 3.46 – 3.31 (m, 1H), 2.98 – 2.80 (m, 2H), 2.76 (m, 2H), 2.57 – 2.38 (m, 2H), 1.92-1.79 (m, 2H), 1.76 – 1.57 (m, 9H).

^{13}C NMR (101 MHz, CDCl3) δ 163.2, 154.8, 154.6, 141.6, 141.3, 132.5, 132.4, 129.4, 80.3, 80.1, 57.4, 52.05, 43.1, 31.00, 29.5, 28.4, 28.4, 28.3, 27.9.

HRMS: Calcd (m/z) for $C_{15}H_{24}N_5O_5^+$ $[M + H]^+$, 354.1772; found, 354.1768.

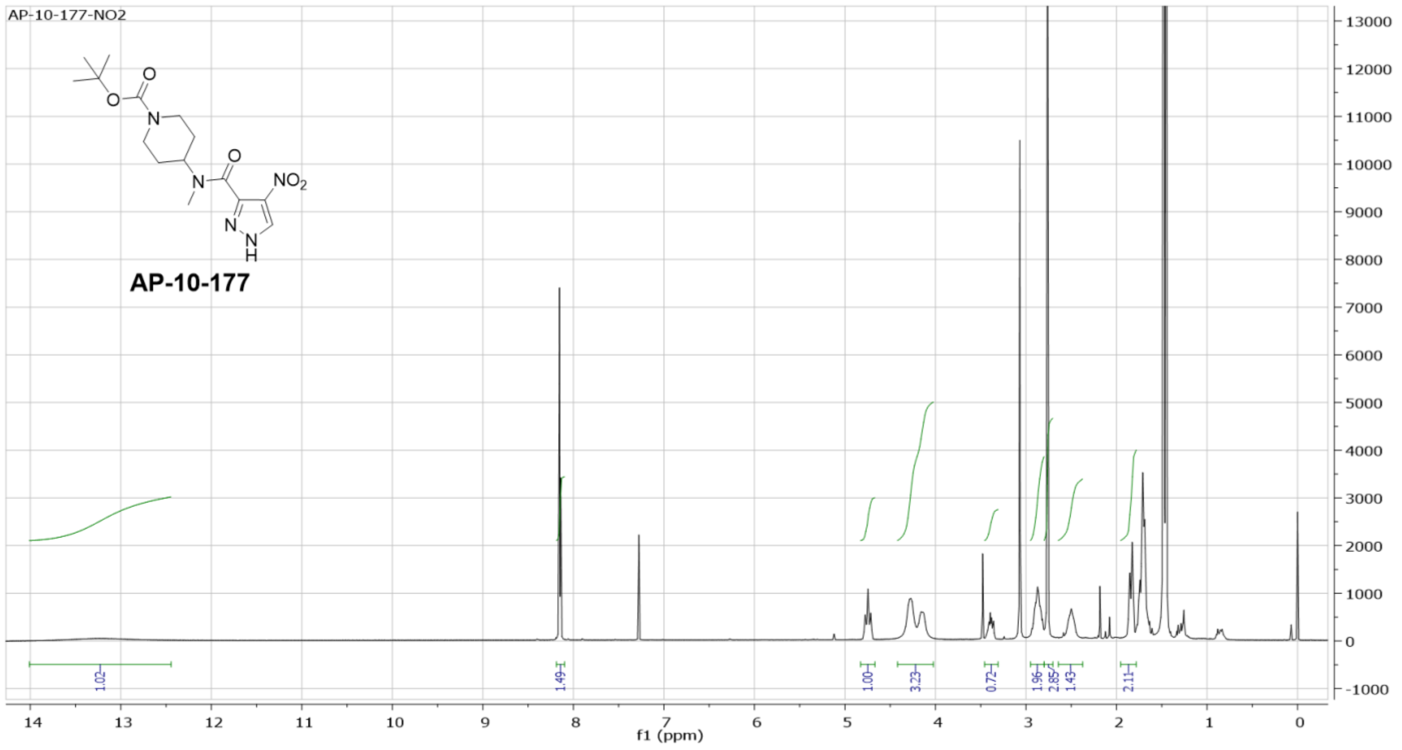

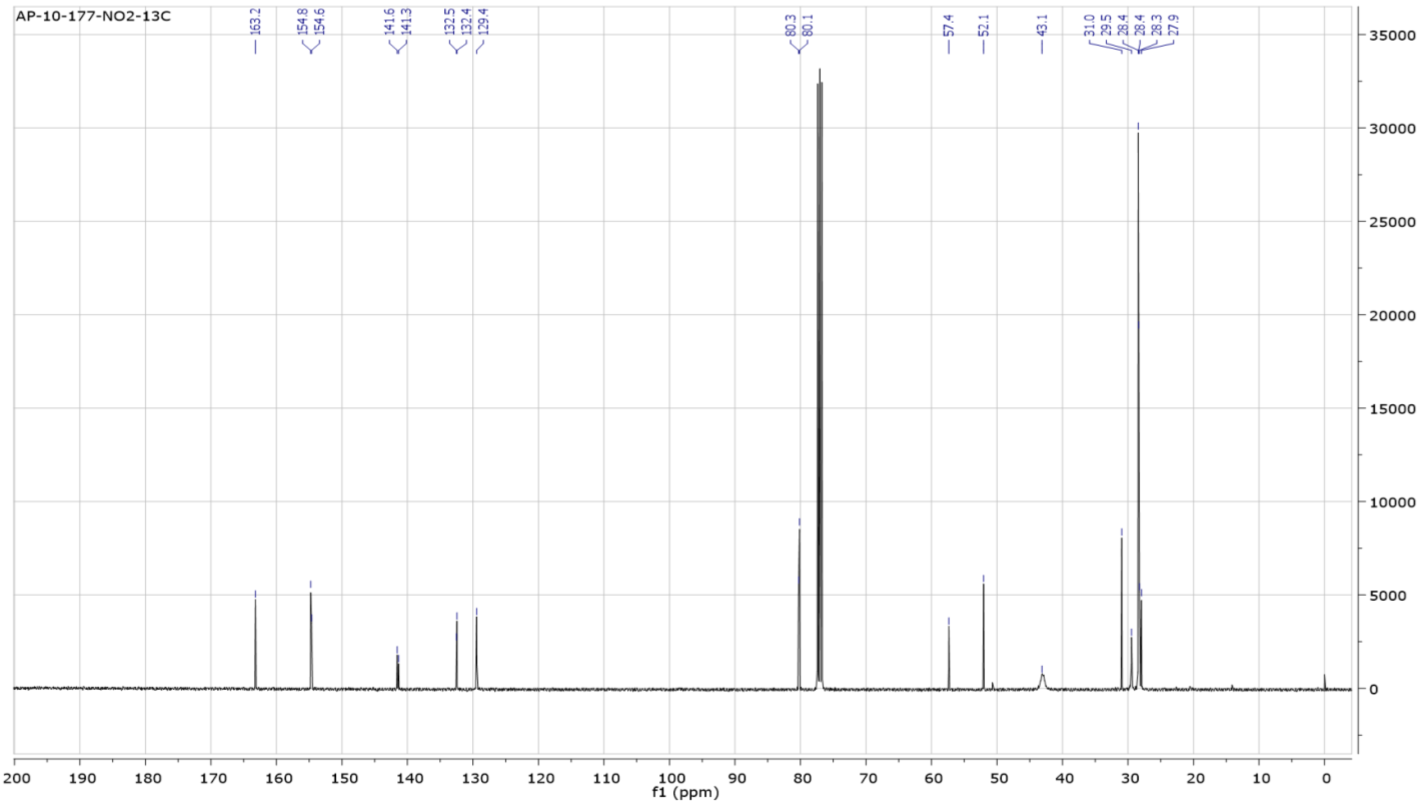
AP-10-177-NO2-13C
163.2
154.8
154.6
141.6
141.3
132.5
132.4
129.4
80.3
80.1
57.4
52.1
43.1
31.0
29.5
28.4
28.4
28.3
27.9
35000
30000
25000
20000
15000
10000
5000
0
200 190 180 170 160 150 140 130 120 110 100 90 80 70 60 50 40 30 20 10 0
f1 (ppm)

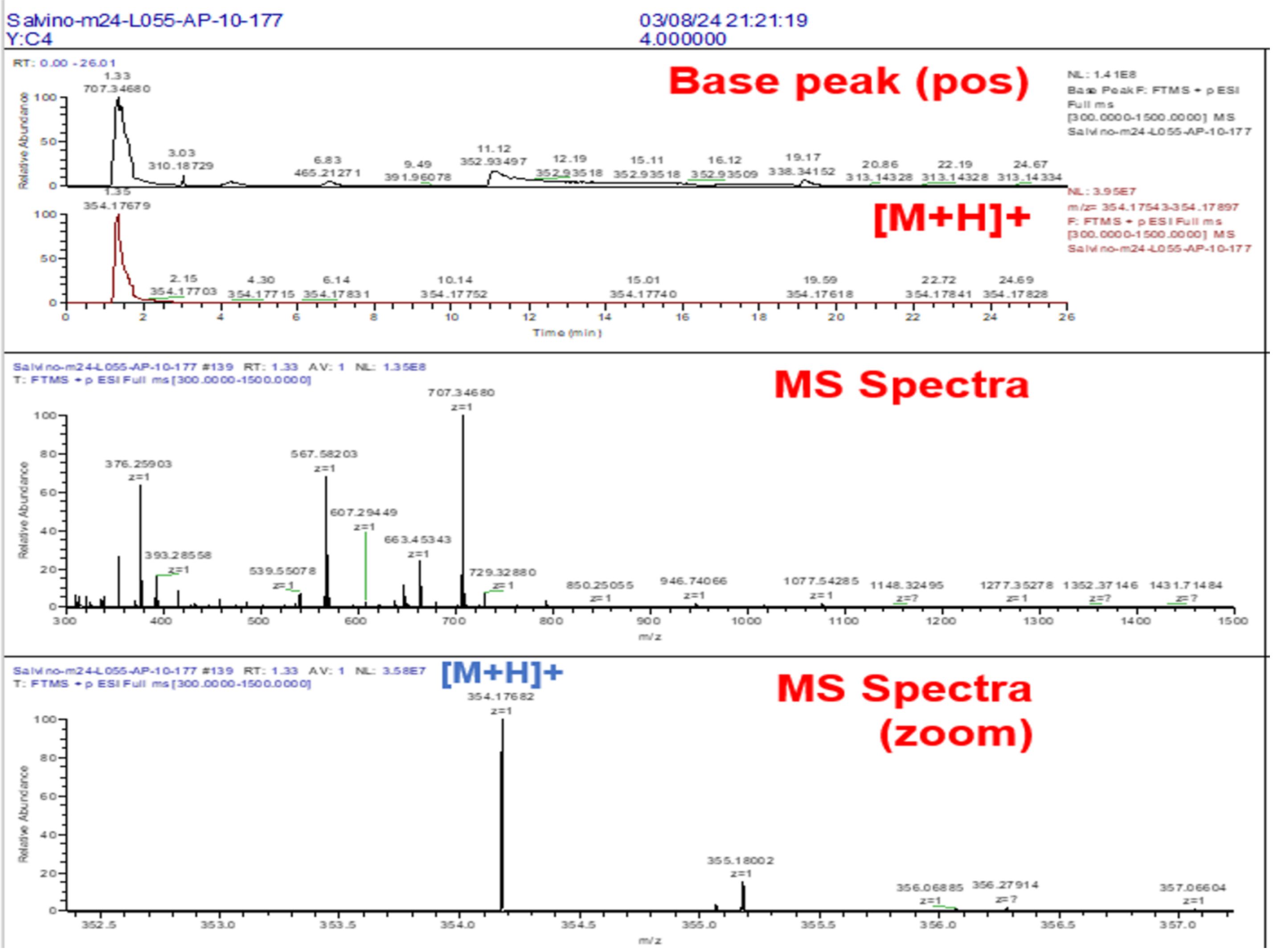
Salvino-m24-L055-AP-10-177
Y:C4
03/08/24 21:21:19
4.000000
Base peak (pos)
[M+H]+
MS Spectra
MS Spectra (zoom)
[M+H]+
NL: 1.41E8
Base Peak F: FTMS + p ESI Full ms [300.0000-1500.0000] MS Salvino-m24-L055-AP-10-177
NL: 3.95E7
m/z= 354.17543-354.17897 F: FTMS + p ESI Full ms [300.0000-1500.0000] MS Salvino-m24-L055-AP-10-177
RT: 0.00 - 26.01
1.33
707.34680
1.35
354.17679
Time (min)
Relative Abundance
Salvino-m24-L055-AP-10-177 #139 RT: 1.33 AV: 1 NL: 1.35E8
T: FTMS + p ESI Full ms [300.0000-1500.0000]
707.34680
z=1
376.25903
z=1
567.58203
z=1
607.29449
z=1
663.45343
z=1
m/z
Salvino-m24-L055-AP-10-177 #139 RT: 1.33 AV: 1 NL: 3.58E7
T: FTMS + p ESI Full ms [300.0000-1500.0000]
354.17682
z=1
355.18002
z=1
m/z

Tert-butyl 4-(4-amino-N-methyl-1H-pyrazole-3-carboxamido)piperidine-1-carboxylate: AP-10-165 (AP-10-183)

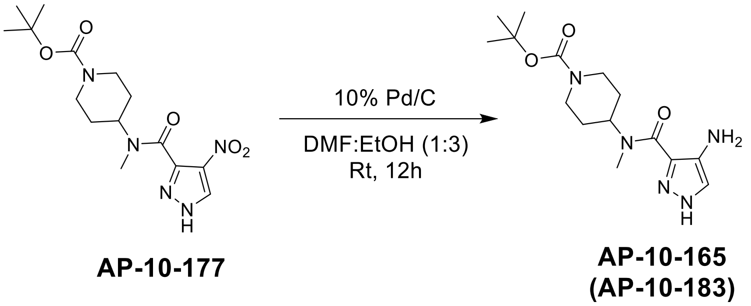

A suspension of tert-butyl 4-(N-methyl-4-nitro-1H-pyrazole-3-carboxamido)piperidine-1-carboxylate (1.1 g, 3.11 mmol) and 10% Pd/C (160 mg) in DMF (10 mL) and EtOH (30 mL) was stirred for 2 h under H_2 at room temperature. Completion of the reaction confirmed by TLC, the reaction mixture was filtered using Celite, the filtrate was concentrated under reduced pressure to obtain a residue which was diluted in ethyl acetate and washed with cold brine solution, then dried over anhydrous Na_2SO_4. The solvent was evaporated under reduced pressure to yield the crude product, which was purified by flash column chromatography to afford the title compound as a light brown solid (0.95 g, 2.95 mmol, 95%). The product was confirmed by ^{1}H, ^{13}C NMR and HRMS.

(Mixture of Isomers) ^{1}H NMR (400 MHz, CDCl3) δ 11.50 (m, 1H), 7.06 (m, 1H), 4.81 (m, 1H), 4.39 – 4.00 (m, 2H), 3.26 (m, 2H), 2.76 (m, 3H), 1.68 (m, 4H), 1.47 (m, 9H).

^{13}C NMR (101 MHz, CDCl3) δ 165.2, 154.8, 133.6, 133.4, 116.5, 79.8, 53.4, 43.2, 29.1, 28.4.

HRMS: Calcd m/z for $C_{15}H_{25}N_5O5^+$ [M + H] $^+$, 324.2030; found, 324.2027.

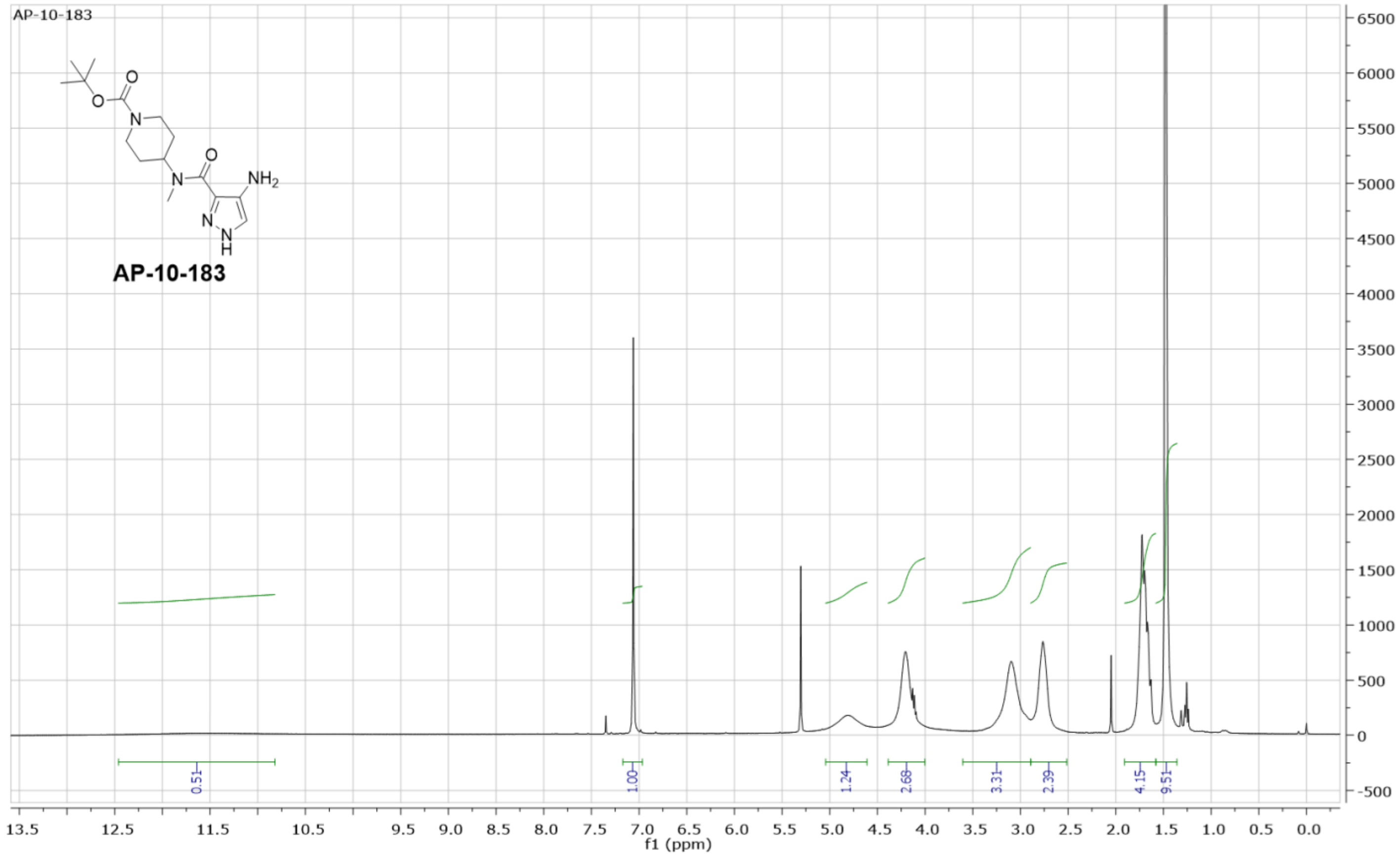

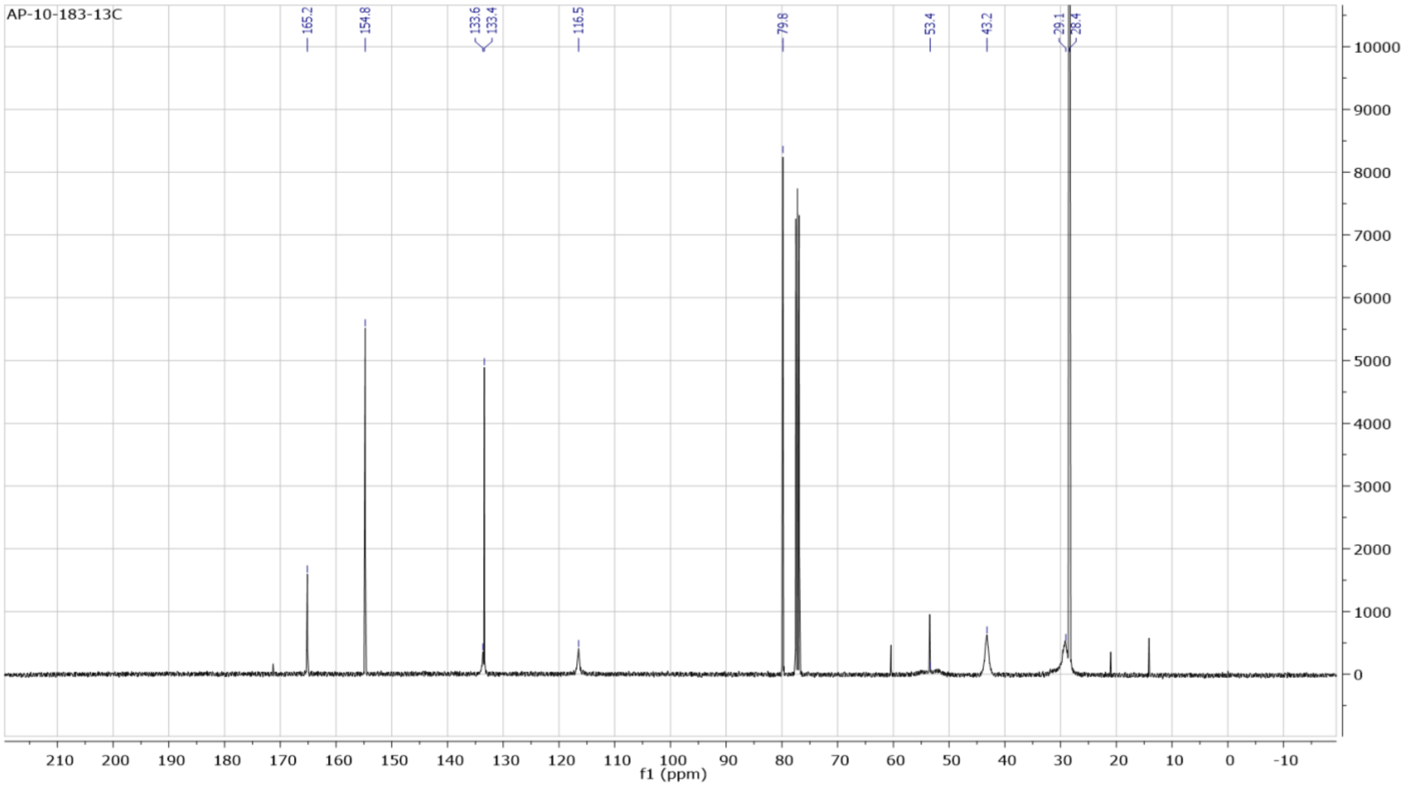

AP-10-183-13C
165.2
154.8
133.6
133.4
116.5
79.8
53.4
43.2
29.1
28.4
10000
9000
8000
7000
6000
5000
4000
3000
2000
1000
0
210
200
190
180
170
160
150
140
130
120
110
100
90
80
70
60
50
40
30
20
10
0
-10
f1 (ppm)

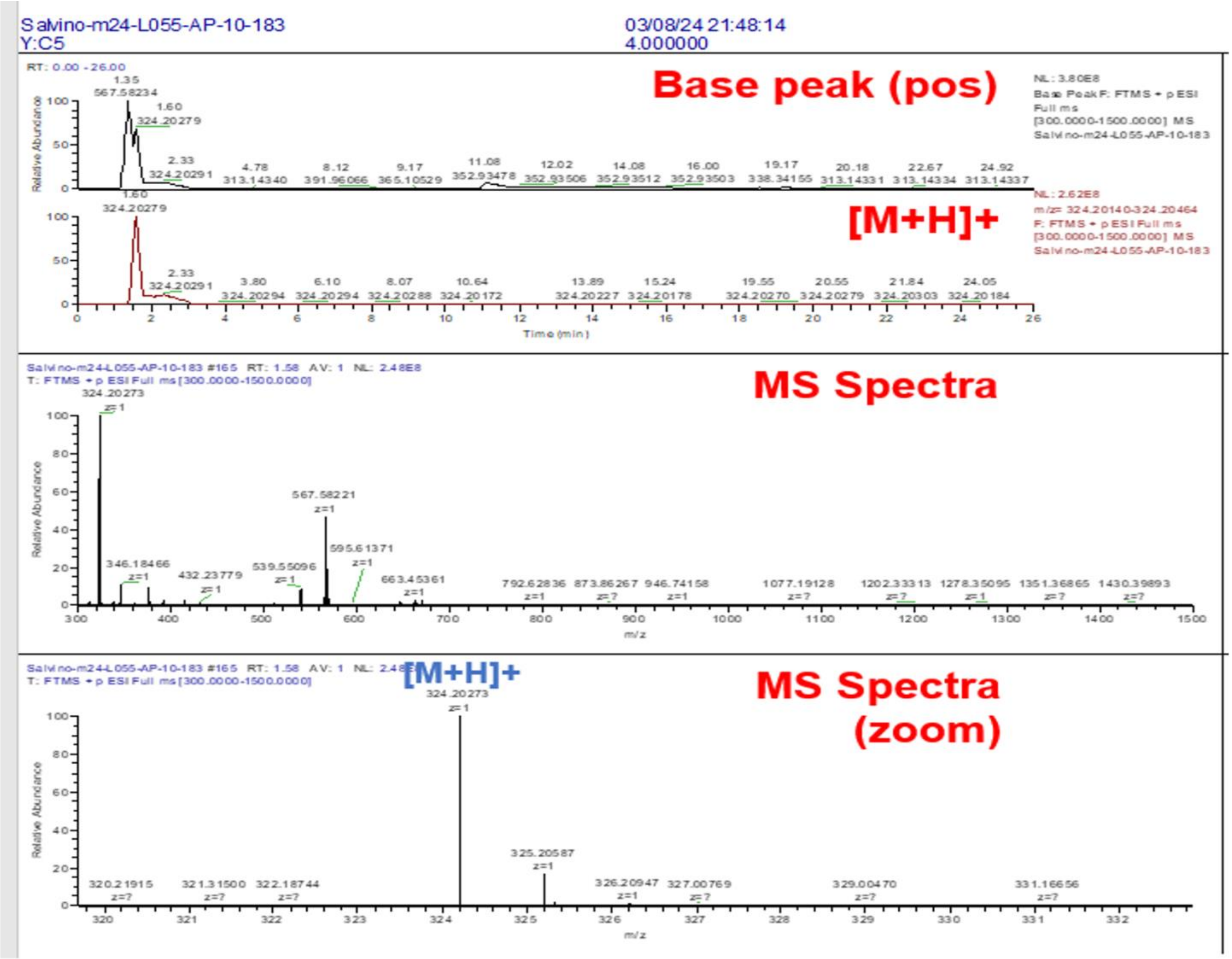

Tert-butyl 4-(4-(2,6-dichlorobenzamido)-1-(2,6-dichlorobenzoyl)-N-methyl-1H-pyrazole-3-carboxamido) piperidine-1-carboxylate: AP-10-169 (AP-10-185)

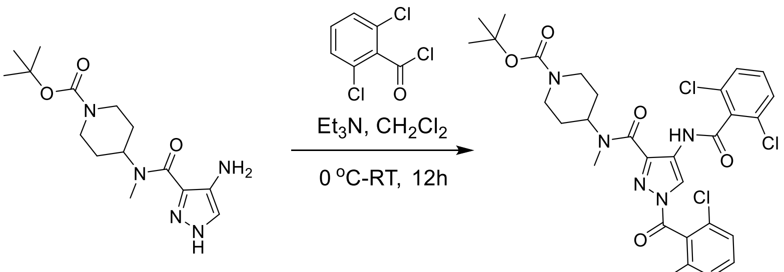

AP-10-165
(AP-10-183)

AP-10-169
(AP-10-185)

To a stirred solution of tert-butyl 6-(4-amino-1H-pyrazole-3-carboxamido)-3-azabicyclo[3.1.0]hexane-3-carboxylate (178 mg; 0.58 mmol) in 5 mL of dry CH_2Cl_2 at 0 °C was added triethyl amine (0.81 mL; 5.79 mmol) and 2,6-dichlorobenzoyl chloride (727 mg; 3.47 mmol) simultaneously dropwise. The reaction mixture was slowly brought to room temperature and stirred for 16 hours. Completion of the reaction was confirmed by LC−MS. The reaction mixture was quenched with 10 mL of cold water. The product was extracted with CH_2Cl_2 and washed with 1N *aq* HCl (10 mL), saturated *aq* $NaHCO_3$ (10 mL),

brine solution (10 mL) and dried over anhydrous Na_2SO_4. The solvent was evaporated under reduced pressure to yield the crude product, which was purified by flash column chromatography to afford the title compound as a white solid (1.1 g; 1.64 mmol, 55%) which was confirmed by ^{1}H, ^{13}C NMR and HRMS.

(Mixture of Isomers) ^{1}H NMR (400 MHz, $CDCl_3$) δ 10.06 (s, 1H), 9.34 (s, 1H), 7.44 – 7.28 (m, 6H), 4.95 – 4.81 (m, 1H), 4.66 – 4.54 (m, 1H), 4.32 – 4.14 (m, 1H), 4.05 – 3.86 (m, 2H), 3.12 – 2.94 (m, 1H), 2.88 (s, 3H), 2.81 – 2.67 (m, 1H), 2.31 – 2.01 (m, 2H), 1.60 – 1.49 (m, 2H), 1.61(H_2O) 1.47 (s, 9H). 1.44(cyclohexnae)

^{13}C NMR (101 MHz, CDCl3) δ 162.75, 162.66, 162.00, 161.93, 154.56, 154.49, 139.97, 135.17, 133.32, 132.59, 132.57, 132.55, 132.53, 131.76, 131.18, 127.86, 127.69, 126.88, 126.75, 79.89, 55.24, 52.42, 31.39, 29.86, 28.44(3C), 28.41.

HRMS: Calcd m/z for $C_{29}H_{29}Cl_4N_5O_5^+$ [M + H] $^+$, 668.0996; found, 668.0997.

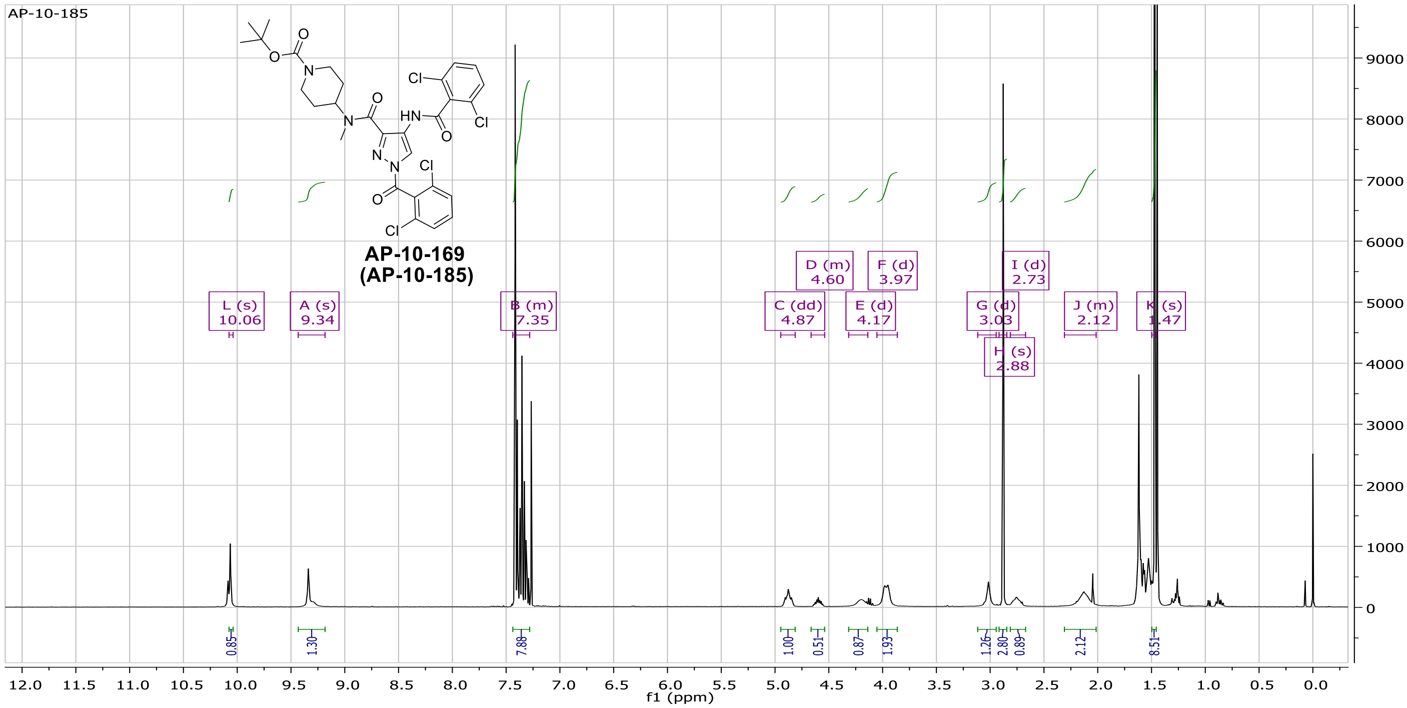

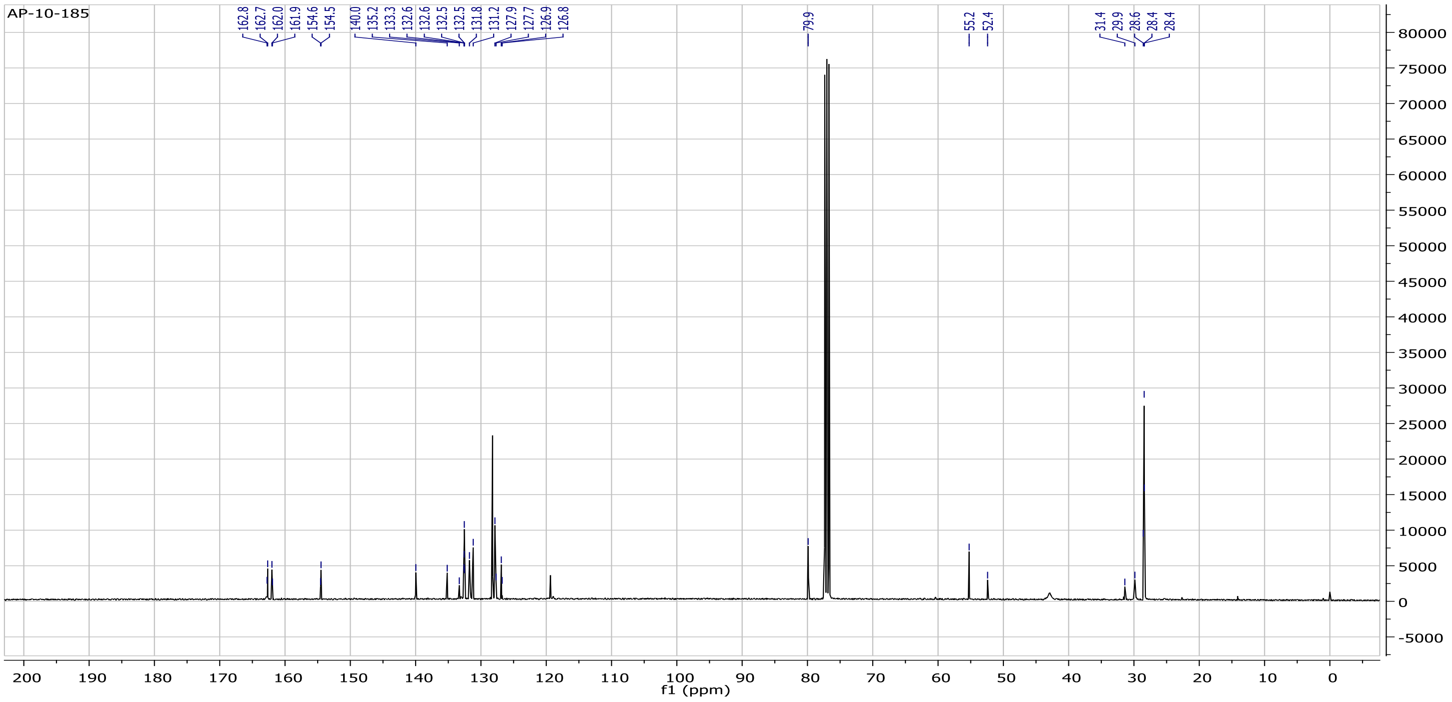

AP-10-185
162.8
162.7
162.0
161.9
154.6
154.5
140.0
135.2
133.3
132.6
132.6
132.5
132.5
131.8
131.2
127.9
127.7
126.9
126.8
79.9
55.2
52.4
31.4
29.9
28.6
28.4
28.4
f1 (ppm)

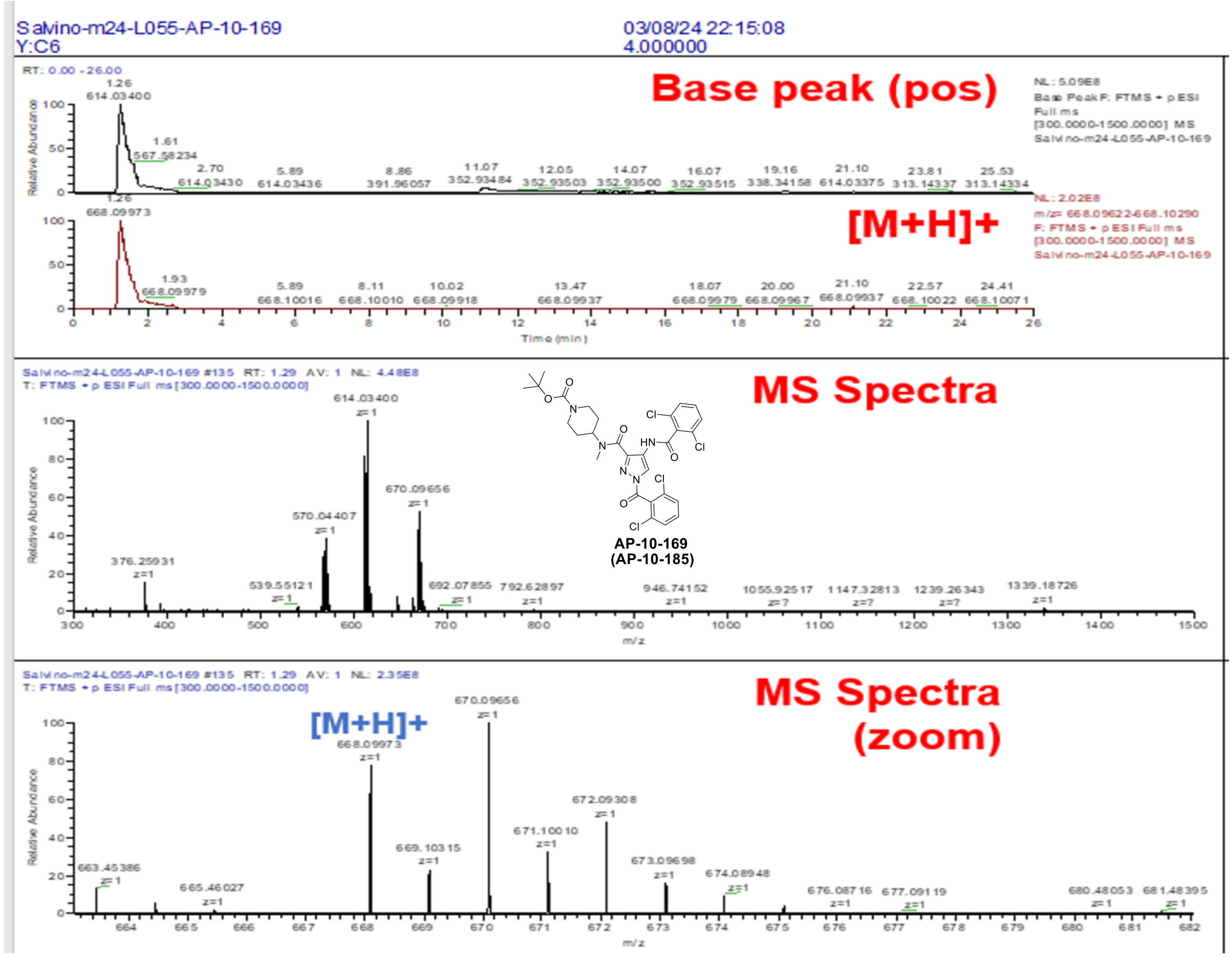

Salvino-m24-L055-AP-10-169
Y:C6
03/08/24 22:15:08
4.000000
Base peak (pos)
[M+H]+
MS Spectra
MS Spectra (zoom)
AP-10-169
(AP-10-185)
614.03400
670.09656
570.04407
376.25931
668.09973
672.09308
671.10010
669.10315
673.09698
674.08948
Time (min)
m/z
Relative Abundance

4-(2,6-dichlorobenzamido)-1-(2,6-dichlorobenzoyl)-N-methyl-N-(piperidin-4-yl)-1H-pyrazole-3-carboxamide: AP-10-197 (FX-171A)

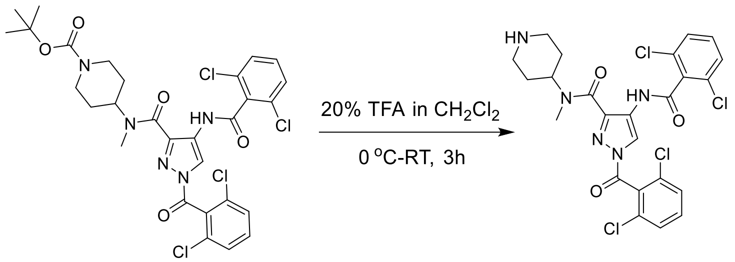

AP-10-169

FX-171-A (AP-10-197)

To a stirred solution of tert-butyl 6-(4-(2,6-dichlorobenzamido)-1-(2,6-dichlorobenzoyl)-1H-pyrazole-3-carboxamido)-3-azabicyclo[3.1.0]hexane-3-carboxylate (60 mg; 0.09 mmol) in 2 mL CH_2Cl_2 at 0 °C was added 0.5 mL of TFA. The reaction mixture was then warmed to room temperature and stirred for 3 hours. Completion of the reaction was confirmed by LC− MS. Volatiles were evaporated under reduced pressure to yield the crude product which was purified by flash column chromatography to afford the title compound as a white solid (51 mg; 0.08 mmol, 85 %) which was confirmed by ^{1}H, ^{13}C NMR and HRMS.

(Mixture of Isomers) ^{1}H NMR (400 MHz, MeOD) δ 9.10-8.87 (m, 1H), 7.68 – 7.44 (m, 6H), 4.69 – 4.26 (m, 2H), 3.54-3.36 (m, 2H), 3.11 (td, J = 12.8, 4.1 Hz, 3H), 3.18-3.06 (m, 2H), 3.05-2.89 (m, 1H), 2.71 (m, 1H) 2.10 – 1.79 (m, 4H).

^{13}C NMR (101 MHz, D2O) δ 165.4, 165.0, 164.1, 164.0, 161.7, 161.3, 161.0, 160.72, 147.5, 147.0, 138.0, 137.9, 135.8, 135.0, 134.0, 134.1, 133.9, 133.8, 130.9, 126.4, 126.1, 121.7, 121.4, 55.6, 51.3, 45.3, 44.9, 33.3, 29.8, 28.5, 27.4.

HRMS: Calcd m/z for $C_{24}H_{21}Cl_4N_5O_3^+$ [M + H] $^+$, 568.0471; found, 568.0473.

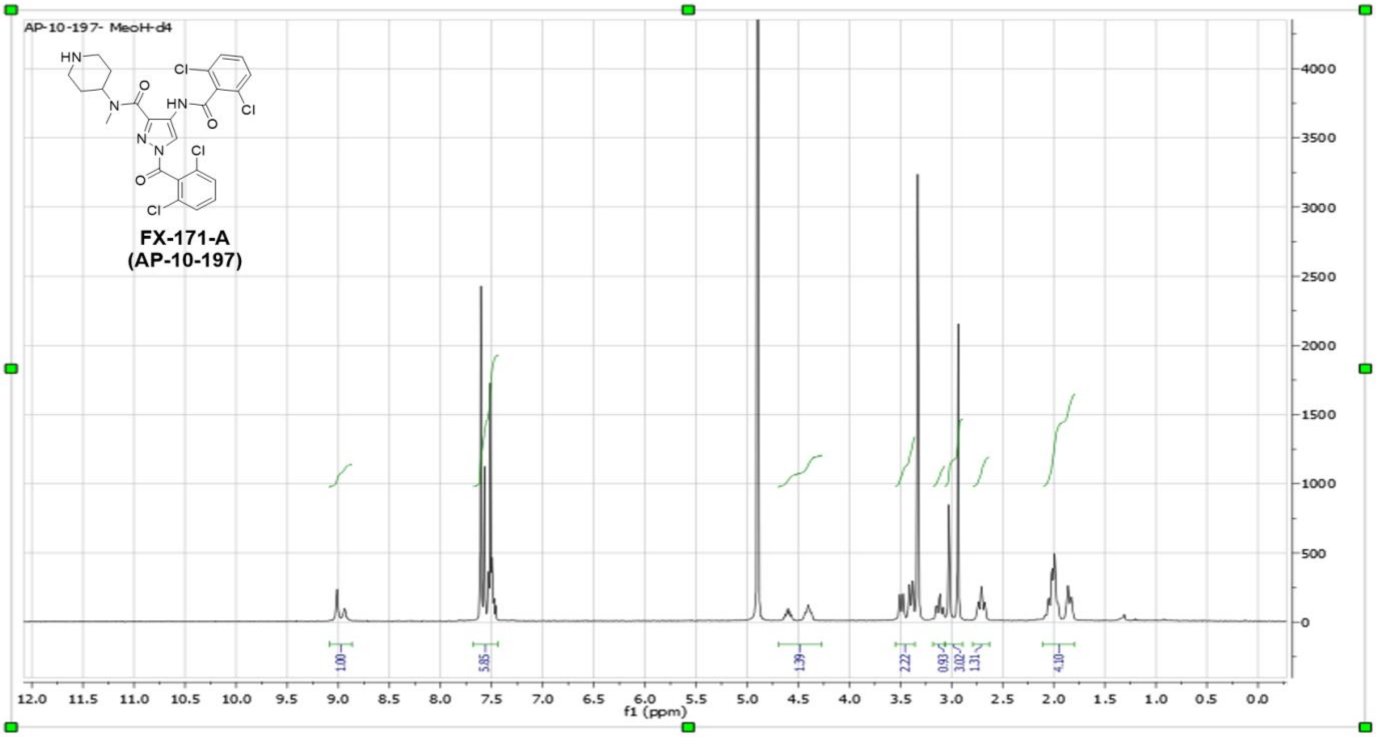

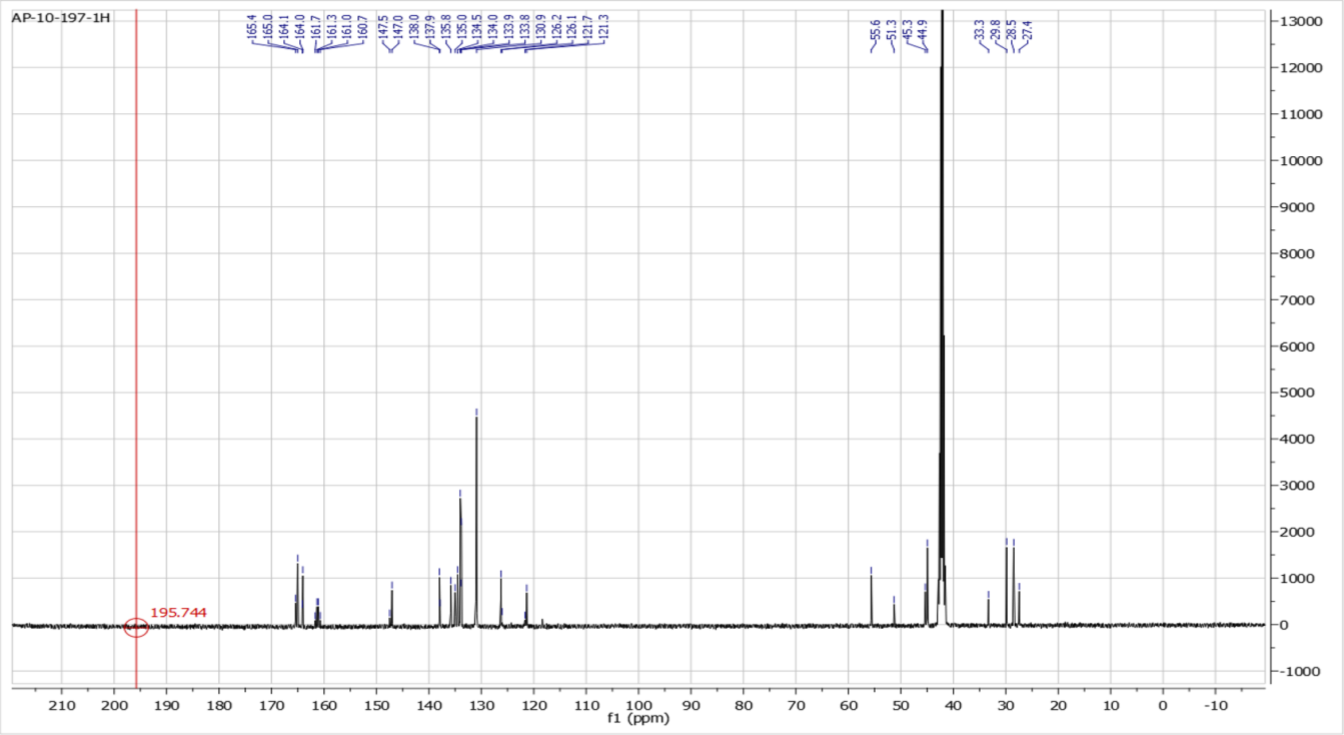
AP-10-197-1H
165.4
165.0
164.1
164.0
161.7
161.3
161.0
160.7
147.5
147.0
138.0
137.9
135.8
135.0
134.5
134.0
133.9
133.8
130.9
126.2
126.1
121.7
121.3
55.6
51.3
45.3
44.9
33.3
29.8
28.5
27.4
195.744
f1 (ppm)

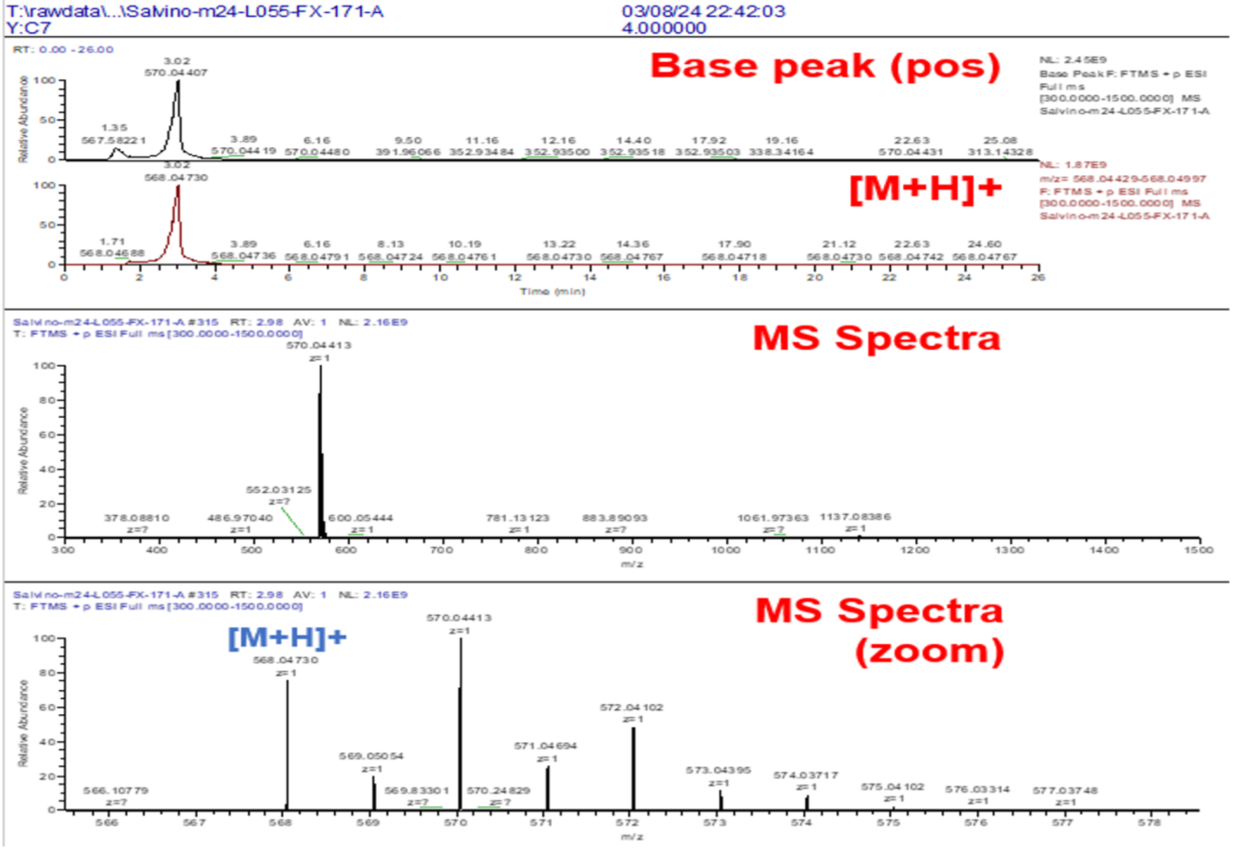
T:\rawdata\...\Salvino-m24-L055-FX-171-A
Y:C7
03/08/24 22:42:03
4.000000
Base peak (pos)
[M+H]+
RT: 0.00 - 26.00
3.02
570.04407
3.02
568.04730
Time (min)
MS Spectra
570.04413
z=1
552.03125
600.05444
Relative Abundance
m/z
MS Spectra
(zoom)
[M+H]+
568.04730
z=1
570.04413
z=1
572.04102
z=1
571.04694
z=1
569.05054
z=1

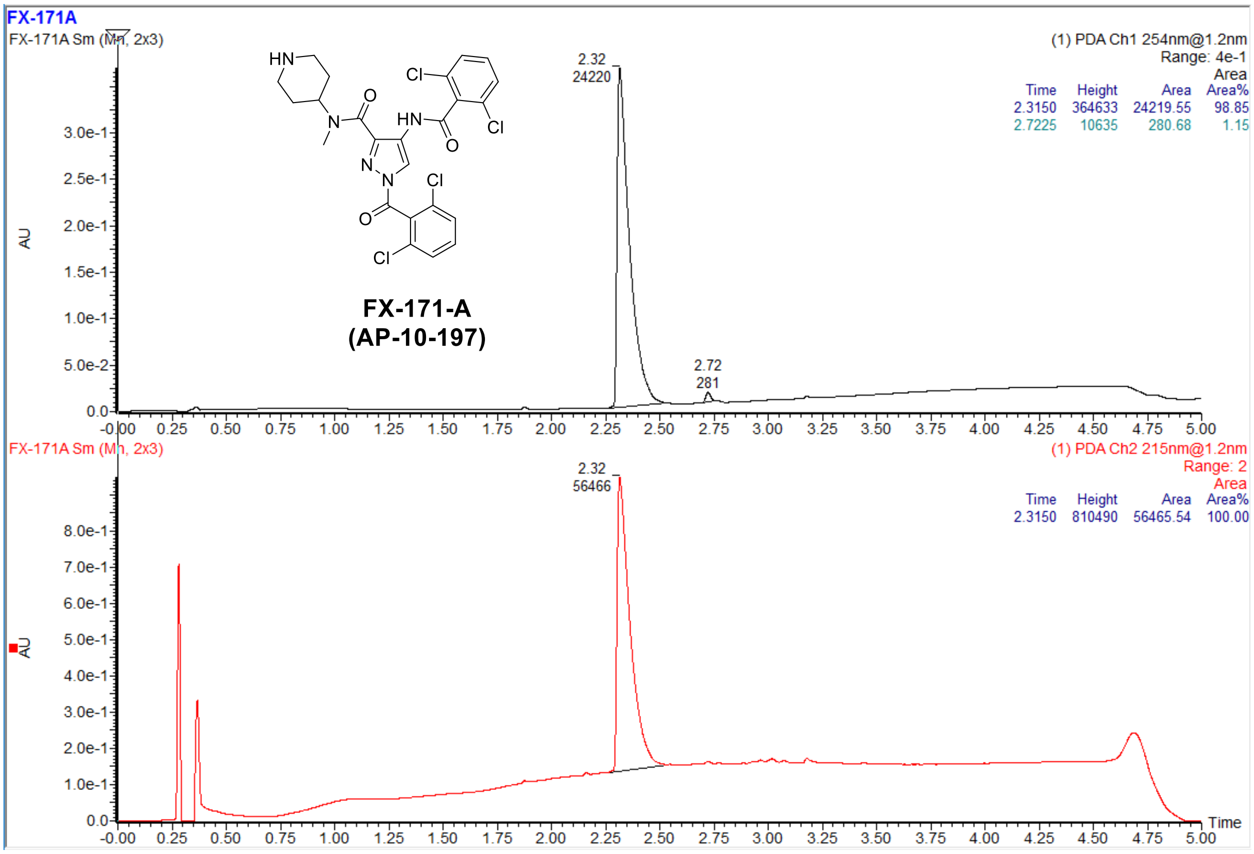

Tert-butyl 4-(4-(2,6-dichlorobenzamido)-1-methyl-1H-pyrazole-3-carboxamido)piperidine-1-carboxylate: AP-10-170

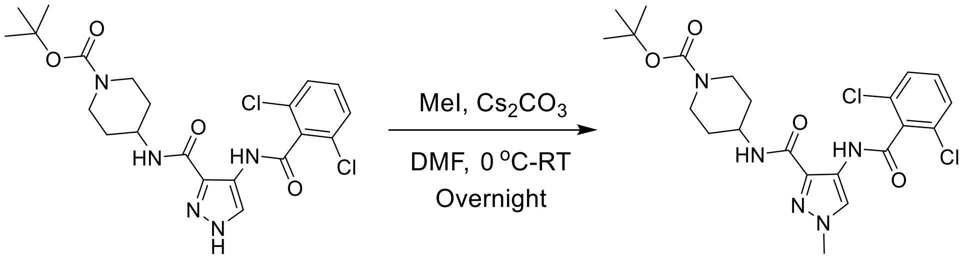

AP-10-170

To a stirred solution of tert-butyl 4-(4-(2,6-dichlorobenzamido)-1H-pyrazole-3-carboxamido)piperidine-1-carboxylate (200 mg; 0.41 mmol) in 10 mL of dry DMF at 0 °C was added Cs_2CO_3 (399.75; 1.23 mmol) and iodomethane (59 mg; 0.41 mmol) simultaneously slowly. The reaction mixture was slowly brought to room temperature and stirred for 16 hours. Completion of the reaction was confirmed by LC−MS. The reaction mixture was quenched with 10 mL of cold water. The product was extracted with CH_2Cl_2 and washed with cold water and brine solution (10 mL) and dried over anhydrous Na_2SO_4. The solvent was evaporated under reduced pressure to yield the crude product, which was purified by flash column chromatography to afford the title compound as a white solid (150 mg; 030 mmol) which was confirmed by ^{1}H, ^{13}C NMR and HRMS.

^{1}H NMR (400 MHz, CDCl3) δ 9.83 (s, 1H), 8.35 (s, 1H), 7.37-7.25 (m, 3H), 6.71 (d, *J* = 8.1 Hz, 1H), 4.23 – 3.96 (m, 3H), 3.92 (s, 3H), 2.88 (t, J = 12.0 Hz, 2H), 1.96 (dd, J = 12.6, 2.8 Hz, 2H), 1.80-1.38 (m, 2H), 1.46 (s, 9H).

^{13}C NMR (101 MHz, CDCl3) δ 162.9, 161.5, 154.7, 135.4, 132.6, 132.3, 131.0, 128.1, 123.5, 123.1, 79.7, 46.4, 39.8, 32.0, 28.4.

HRMS: Calcd m/z for $C_{14}H_{28}Cl_2N_5O_4^+$ [M + H] $^+$, 496.1513; found, 496.1512.

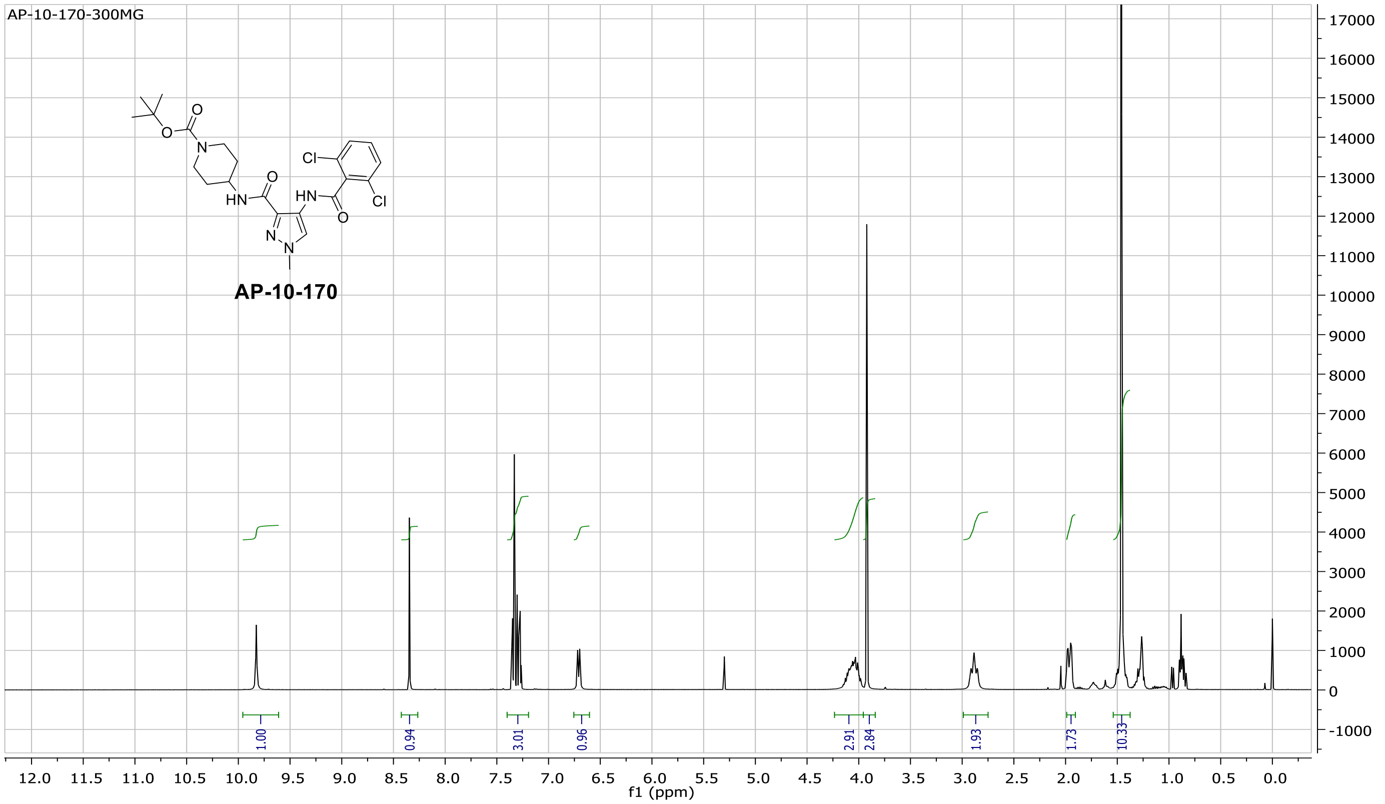

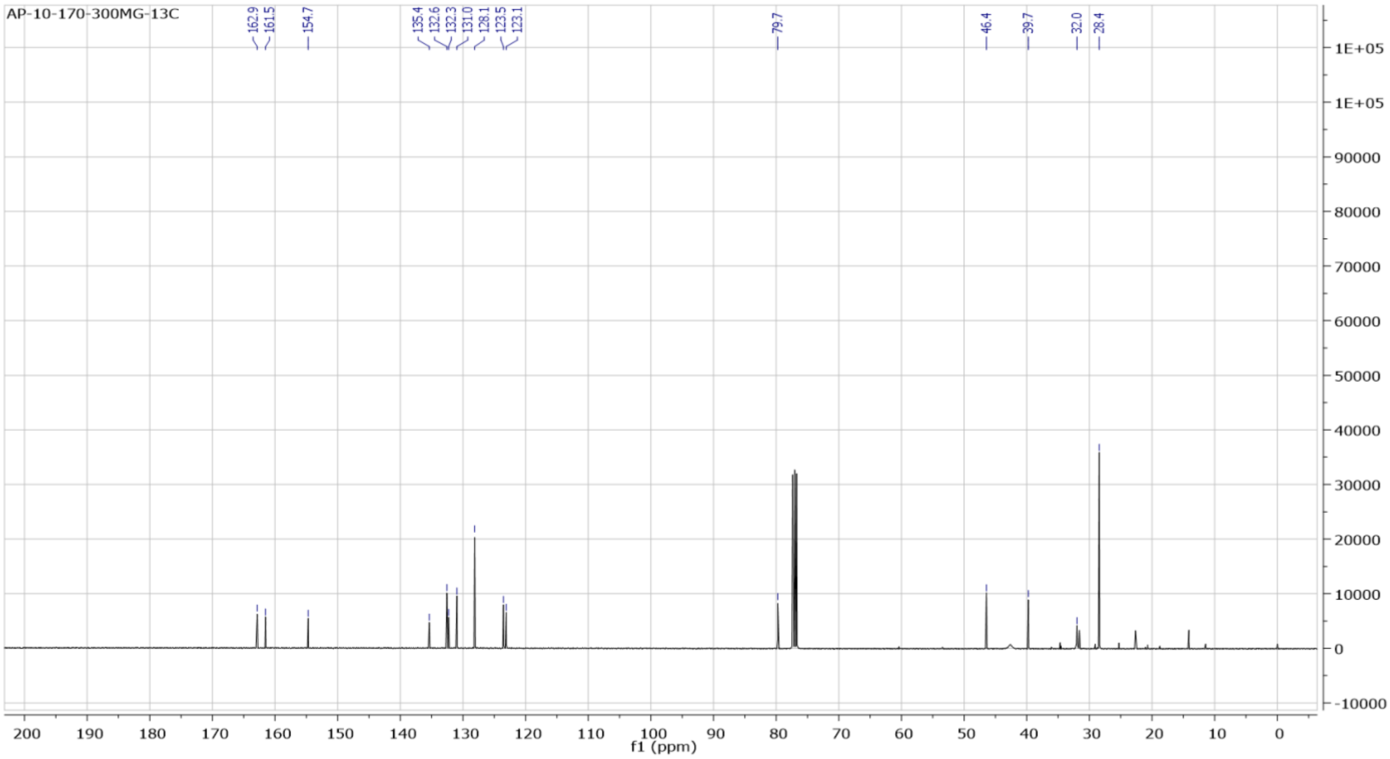

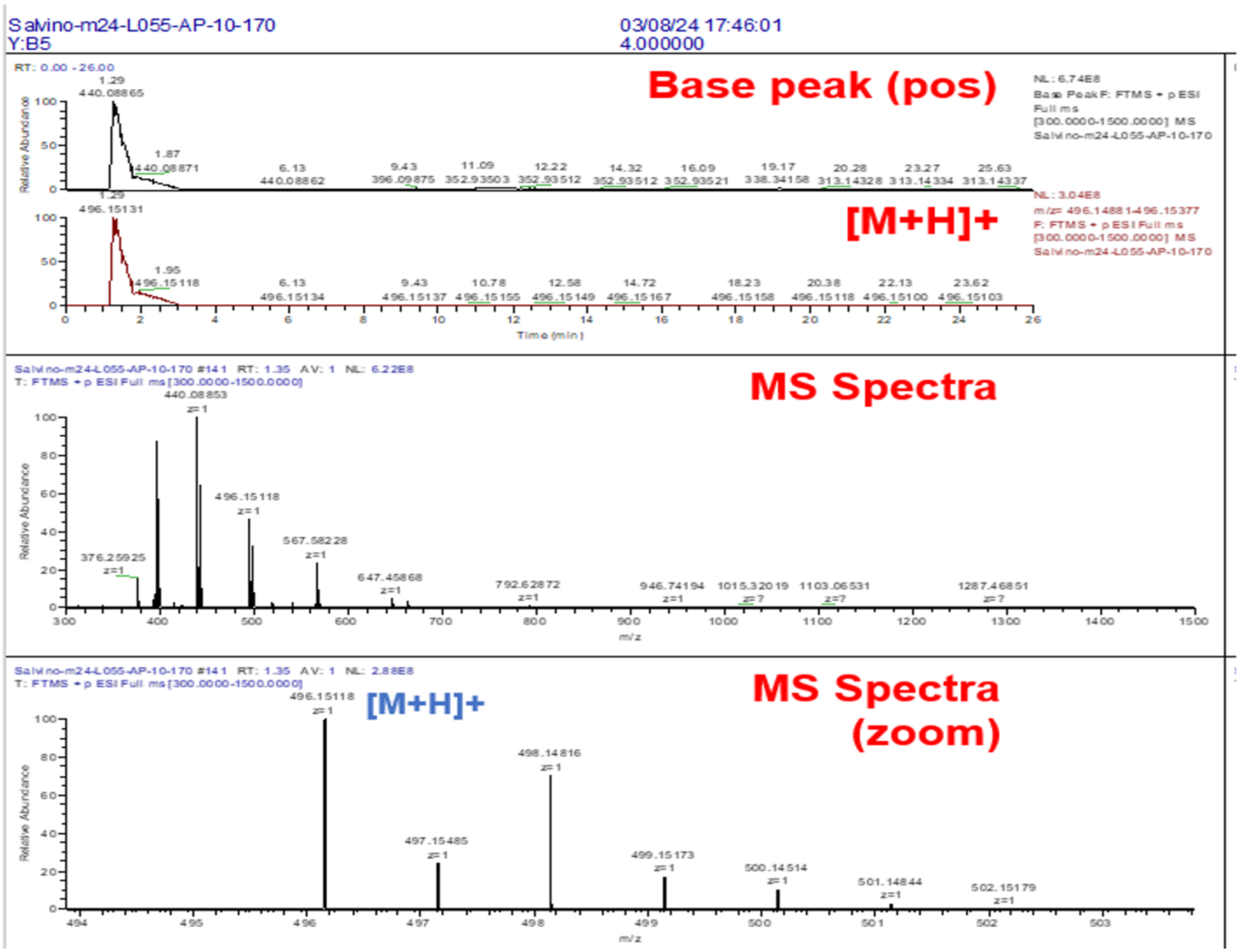

4-(2,6-Dichlorobenzamido)-1-methyl-N-(piperidin-4-yl)-1H-pyrazole-3-carboxamide: (AP-10-186)

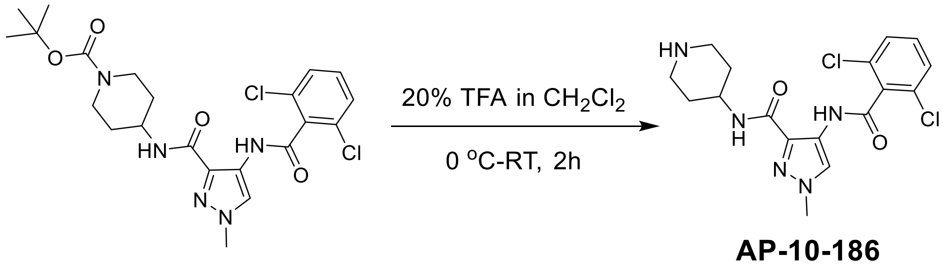

AP-10-170 **AP-10-186**

To a stirred solution of tert-butyl 4-(4-(2,6-dichlorobenzamido)-1-(2,6-dichlorobenzoyl)-1H-pyrazole-3-carboxamido)piperidine-1-carboxylate (100 mg; 0.20 mmol) in 6 mL CH_2Cl_2 at 0 °C was added 2 mL of TFA. The reaction mixture was then warmed to room temperature and stirred for 3 hours. Completion of the reaction was confirmed by LC−MS. Volatiles were evaporated under reduced pressure to yield the crude product which was purified by flash column chromatography to afford the title compound as a white solid (65 mg; 0.15 mmol, 82%) which was confirmed by ^{1}H, ^{13}C NMR and HRMS.

^{1}H NMR (400 MHz, DMSO) δ 10.08 (s, 1H), 9.40 (d, *J* = 9.5 Hz, 1H), 9.01 (d, *J* = 9.1 Hz, 2H), 8.53 (t, *J* = 16.3 Hz, 1H), 8.34 (s, 1H), 7.64 – 7.27 (m, 3H), 3.97-3.78 (m, 4H), 3.72 – 3.34 (m, 1H), 3.17 (d, *J* = 12.5 Hz, 4H), 2.83 (d, *J* = 10.3 Hz, 2H), 2.48 – 2.33 (m, 1H), 1.91 – 1.59 (m, 4H).

^{13}C NMR (101 MHz, DMSO) δ 162.9, 160.8, 135.7, 132.7, 132.4, 131.7, 128.9, 124.1, 122.4, 66.8, 44.3, 42.5, 39.8, 28.3.

HRMS: Calcd m/z for $C_{17}H_{20}Cl_2N_5O_2^+$ [M + H] $^+$, 396.0989; found, 396.0987.

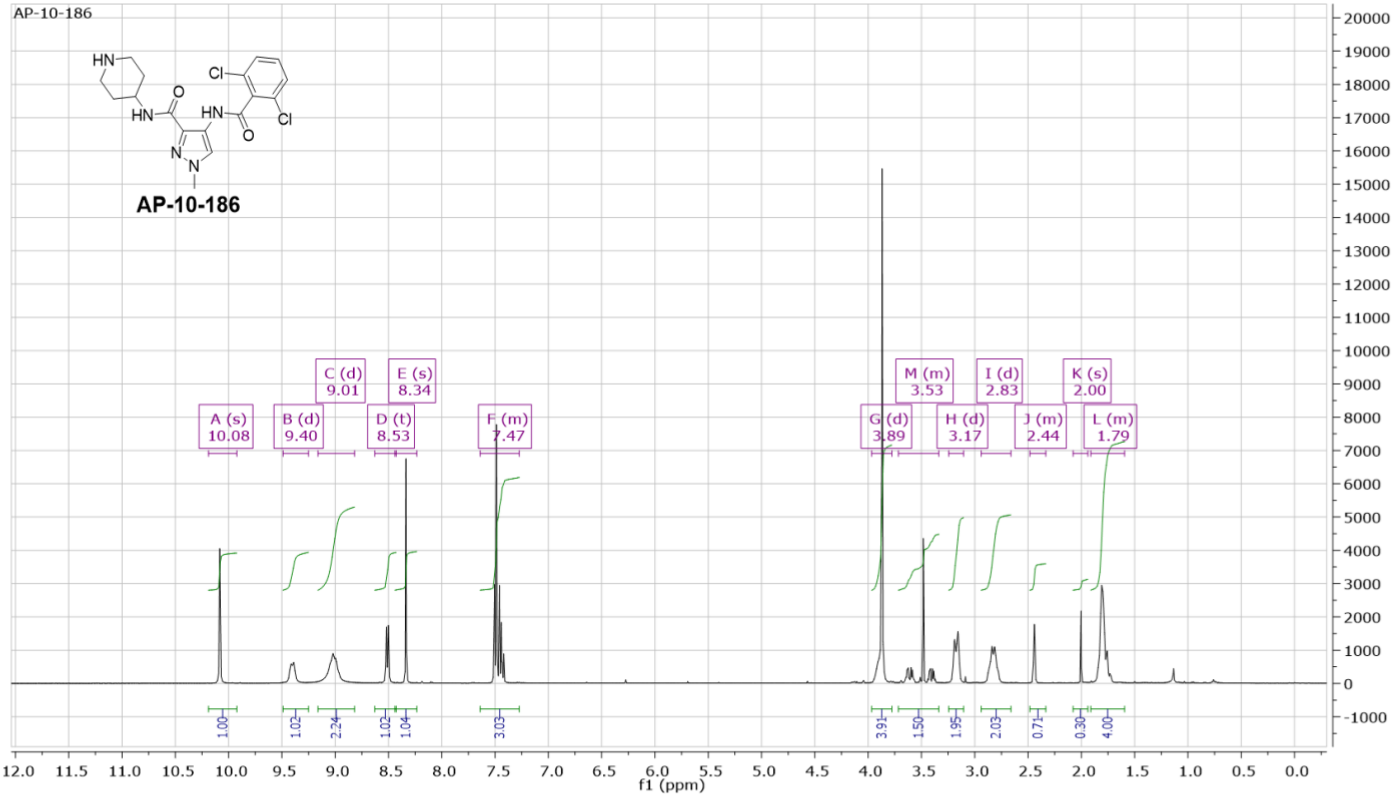

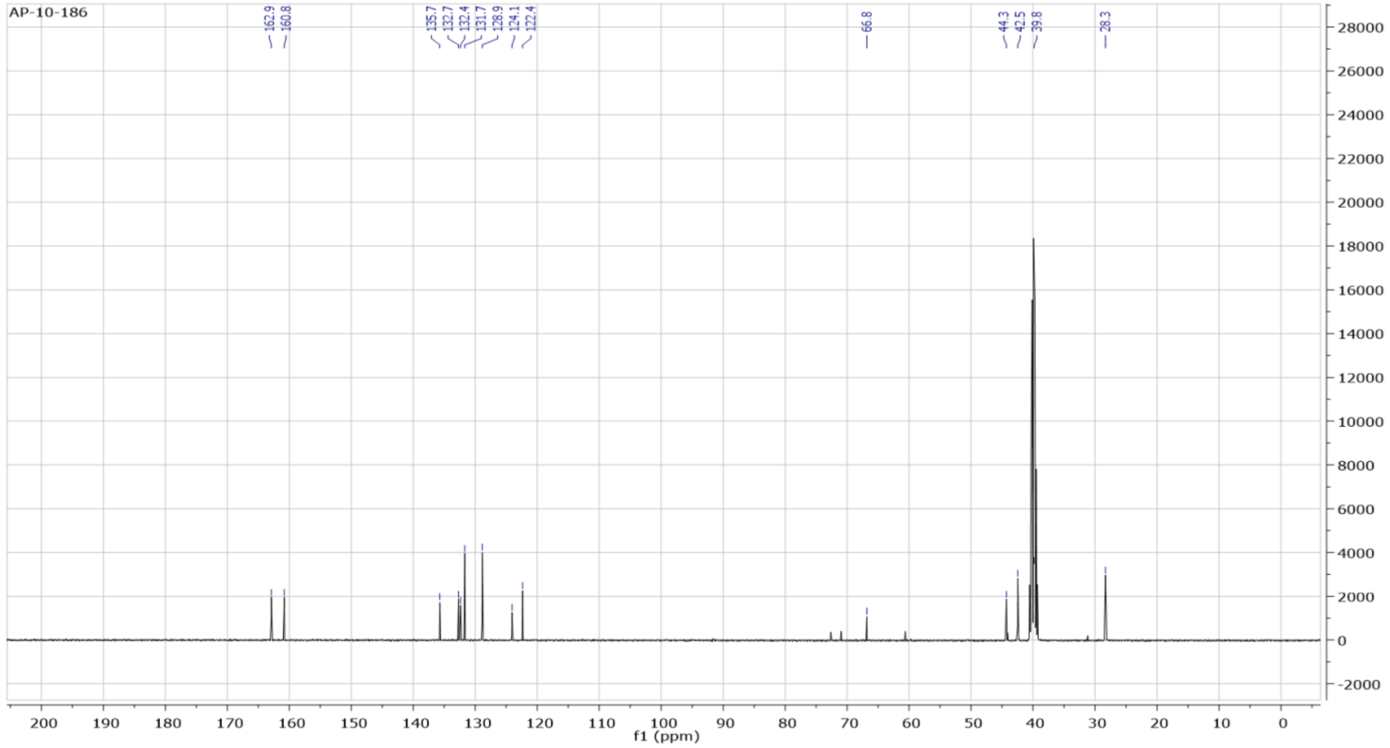

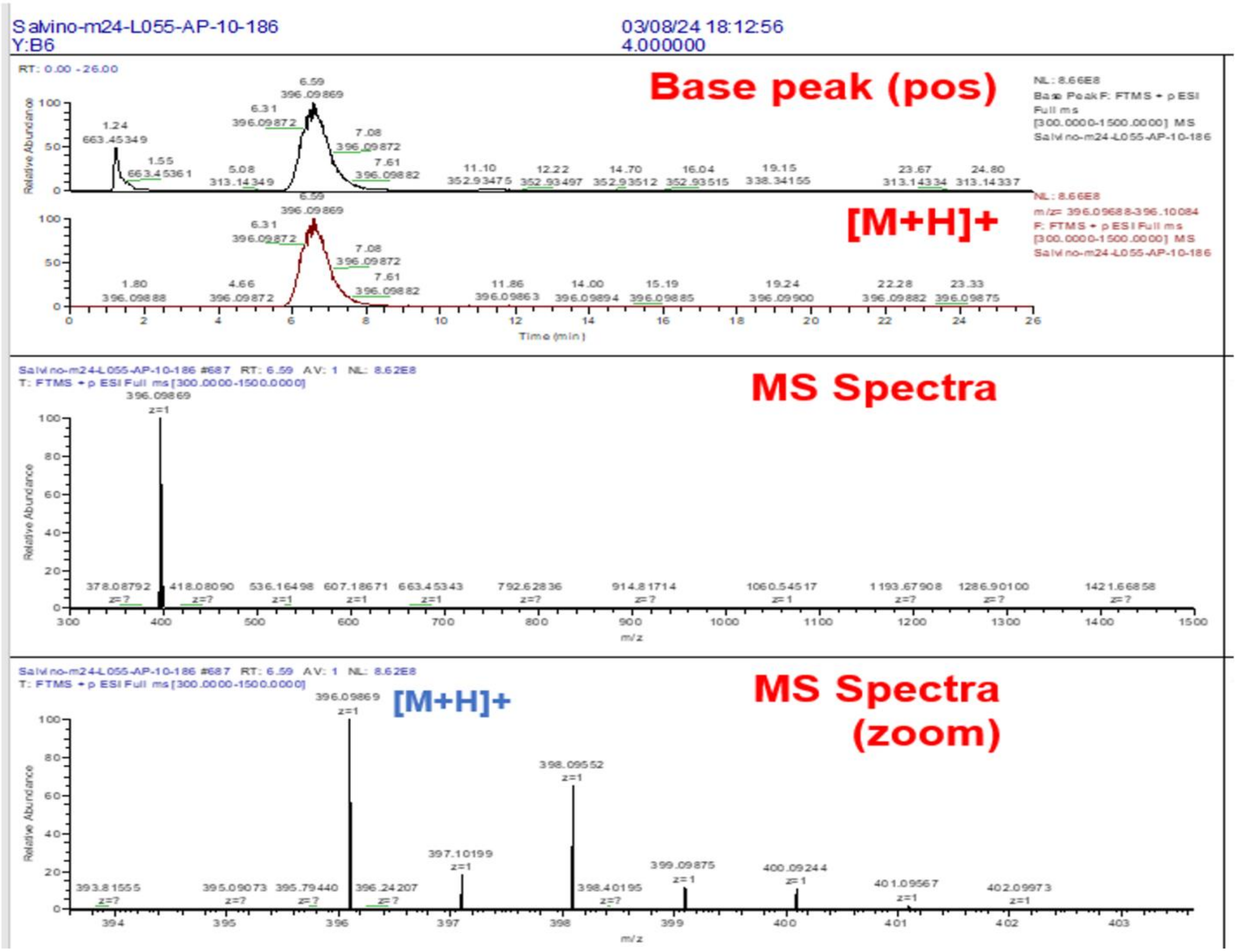

4-(2,6-Dichlorobenzamido)-N-(1-(2,6-dichlorobenzoyl)piperidin-4-yl)-1H-pyrazole-3-carboxamide: AP-05-145 (AP-10-173)

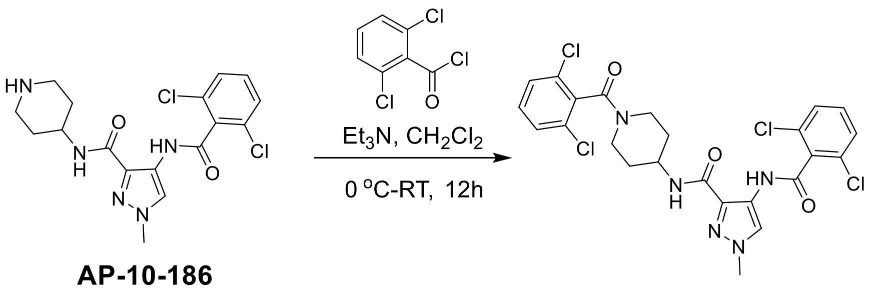

To a stirred solution of tert-butyl 4-(4-amino-1H-pyrazole-3-carboxamido)piperidine-1-carboxylate (50 mg; 0.13 mmol) in 10 mL of dry CH_2Cl_2 at 0 °C was added triethyl amine (65 mg; 0.65mmol) and 2,6-dichlorobenzoyl chloride (34 mg; 0.16 mmol) simultaneously dropwise. The reaction mixture was slowly brought to room temperature and stirred for 16 hours. Completion of the reaction was confirmed by LC−MS. The reaction mixture was quenched with 10 mL of cold water. The product was extracted with CH_2Cl_2 and washed with 1N *aq* HCl (10 mL), saturated *aq* $NaHCO_3$ (10 mL), brine solution (10 mL), and dried over anhydrous Na_2SO_4. The solvent was evaporated under reduced pressure to yield the crude

product, which was purified by flash column chromatography to afford the AP-05-145 (AP-10-173) as a white solid (50 mg; 0.09 mmol). The product was confirmed by ^{1}H, ^{13}C NMR and HRMS.

^{1}H NMR (400 MHz, CDCl3) δ 9.77 (s, 1H), 8.36 (s, 1H), 7.41 – 7.20 (m, 6H), 6.73 (d, *J* = 8.1 Hz, 1H), 4.77 (d, *J* = 13.7 Hz, 1H), 4.23 – 4.06 (m, 1H), 3.93 (s, 3H), 3.42 (d, *J* = 14.3 Hz, 1H), 3.25 – 3.12 (m, 1H), 3.12 – 2.97 (m, 1H), 2.22 – 2.08 (m, 1H), 2.00 (dd, *J* = 9.1, 3.6 Hz, 1H), 1.92 – 1.38 (m, 2H).

^{13}C NMR (101 MHz, CDCl3) δ 163.6, 162.9, 161.5, 135.3, 135.0, 132.5, 132.1, 131.8, 131.6, 131.0, 130.4, 128.1, 128.1, 123.6, 123.1, 46.3, 45.1, 40.3, 39.8, 32.4, 31.6.

HRMS: Calcd m/z for $C_{24}H_{21}Cl_4N_5O3^+$ [M + H] $^+$, 568.1471; found, 568.1472.

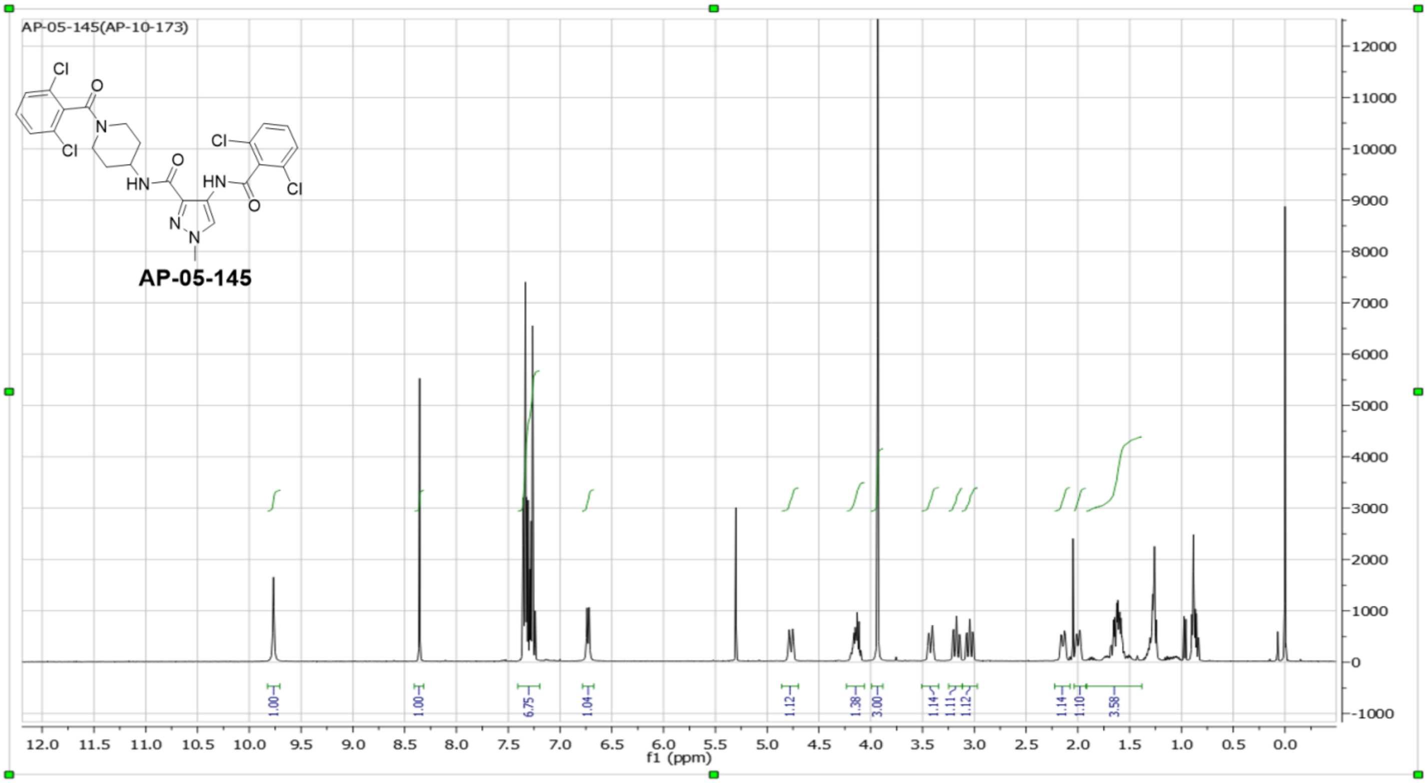

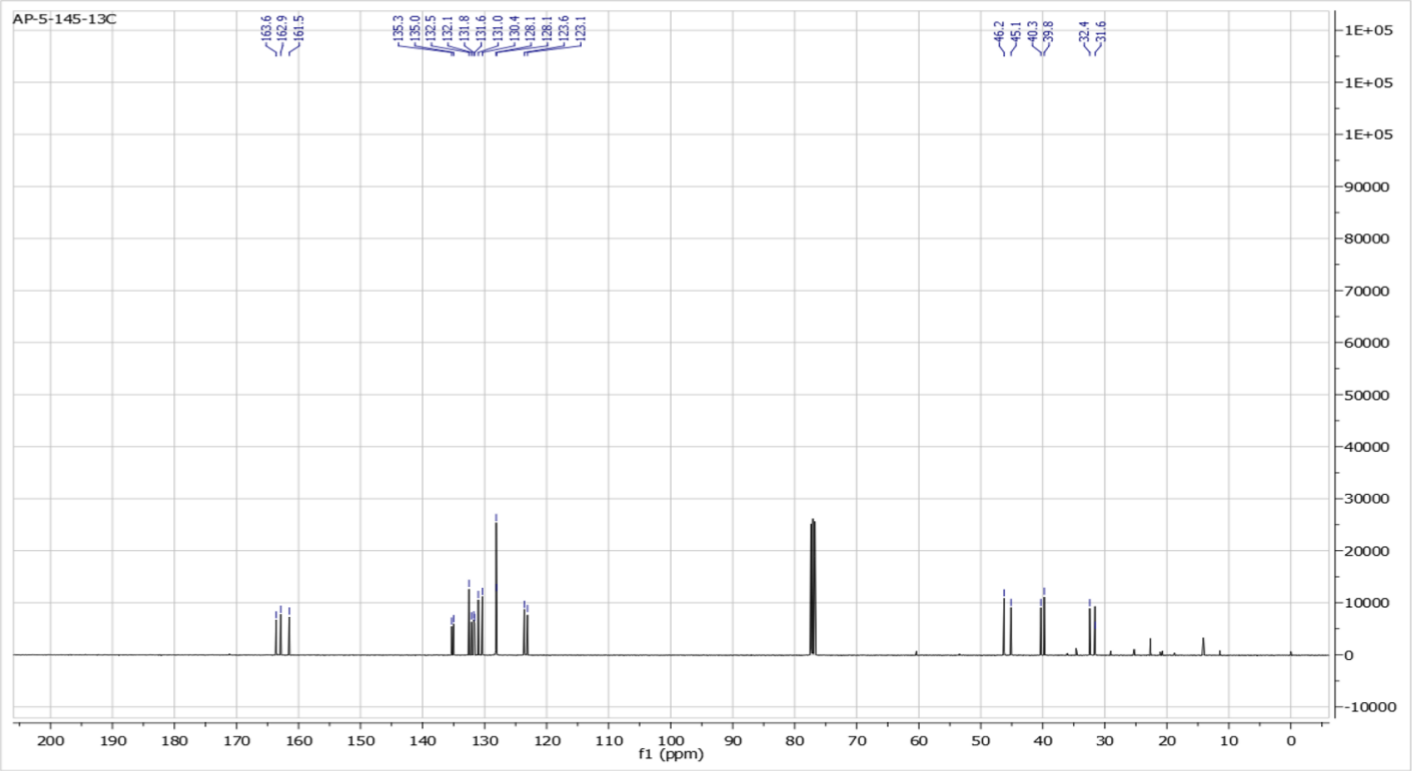

AP-5-145-13C
163.6
162.9
161.5
135.3
135.0
132.5
132.1
131.8
131.6
131.0
130.4
128.1
128.1
123.6
123.1
46.2
45.1
40.3
39.8
32.4
31.6
f1 (ppm)

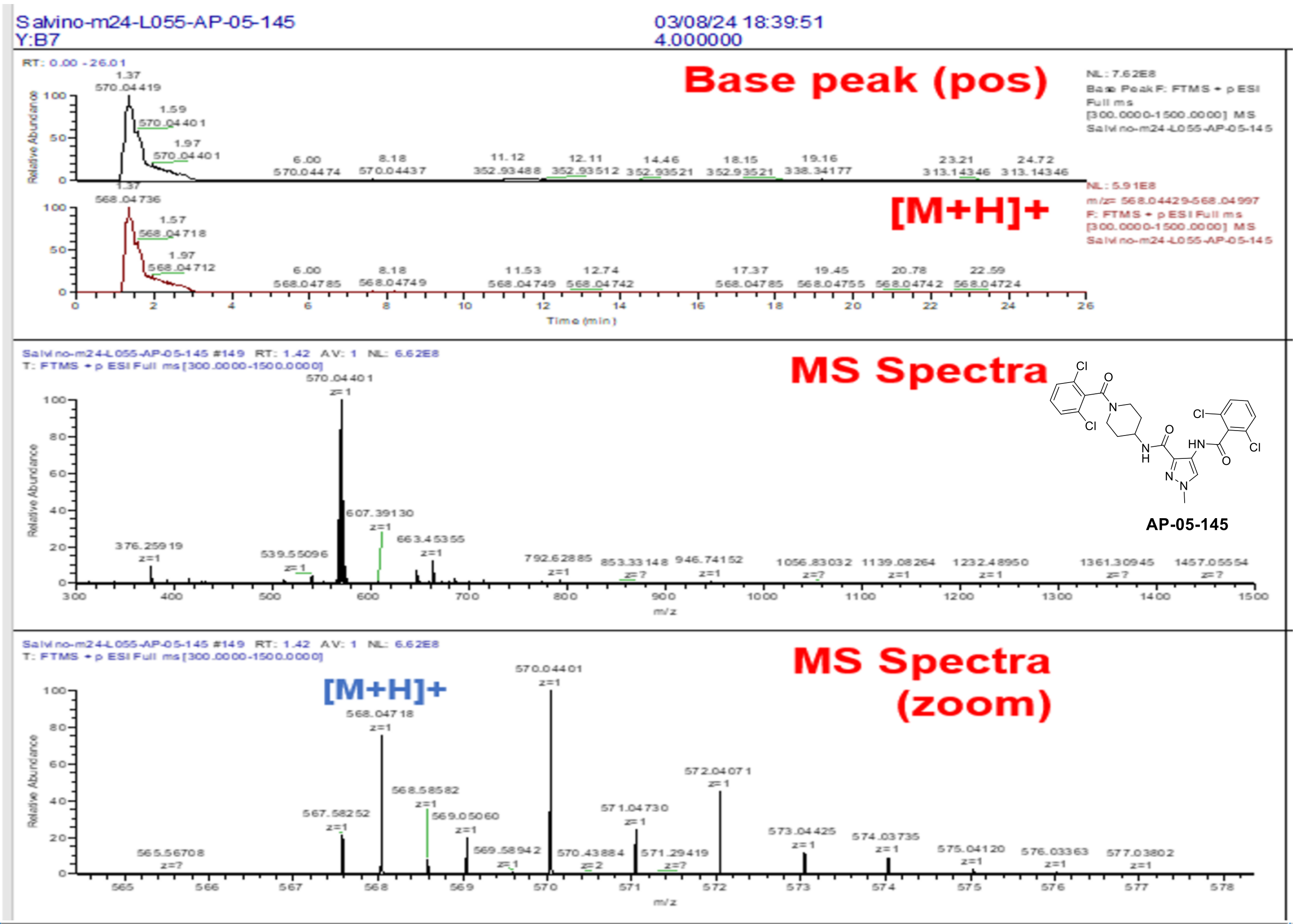

Salvino-m24-L055-AP-05-145
Y:B7
03/08/24 18:39:51
4.000000
Base peak (pos)
[M+H]+
MS Spectra
MS Spectra (zoom)
AP-05-145
RT: 0.00 - 26.01
NL: 7.62E8
Base Peak F: FTMS + p ESI Full ms [300.0000-1500.0000] MS Salvino-m24-L055-AP-05-145
NL: 5.91E8
m/z= 568.04429-568.04997 F: FTMS + p ESI Full ms [300.0000-1500.0000] MS Salvino-m24-L055-AP-05-145
Time (min)
Relative Abundance
Salvino-m24-L055-AP-05-145 #149 RT: 1.42 AV: 1 NL: 6.62E8
T: FTMS + p ESI Full ms [300.0000-1500.0000]
570.04401
568.04718
m/z

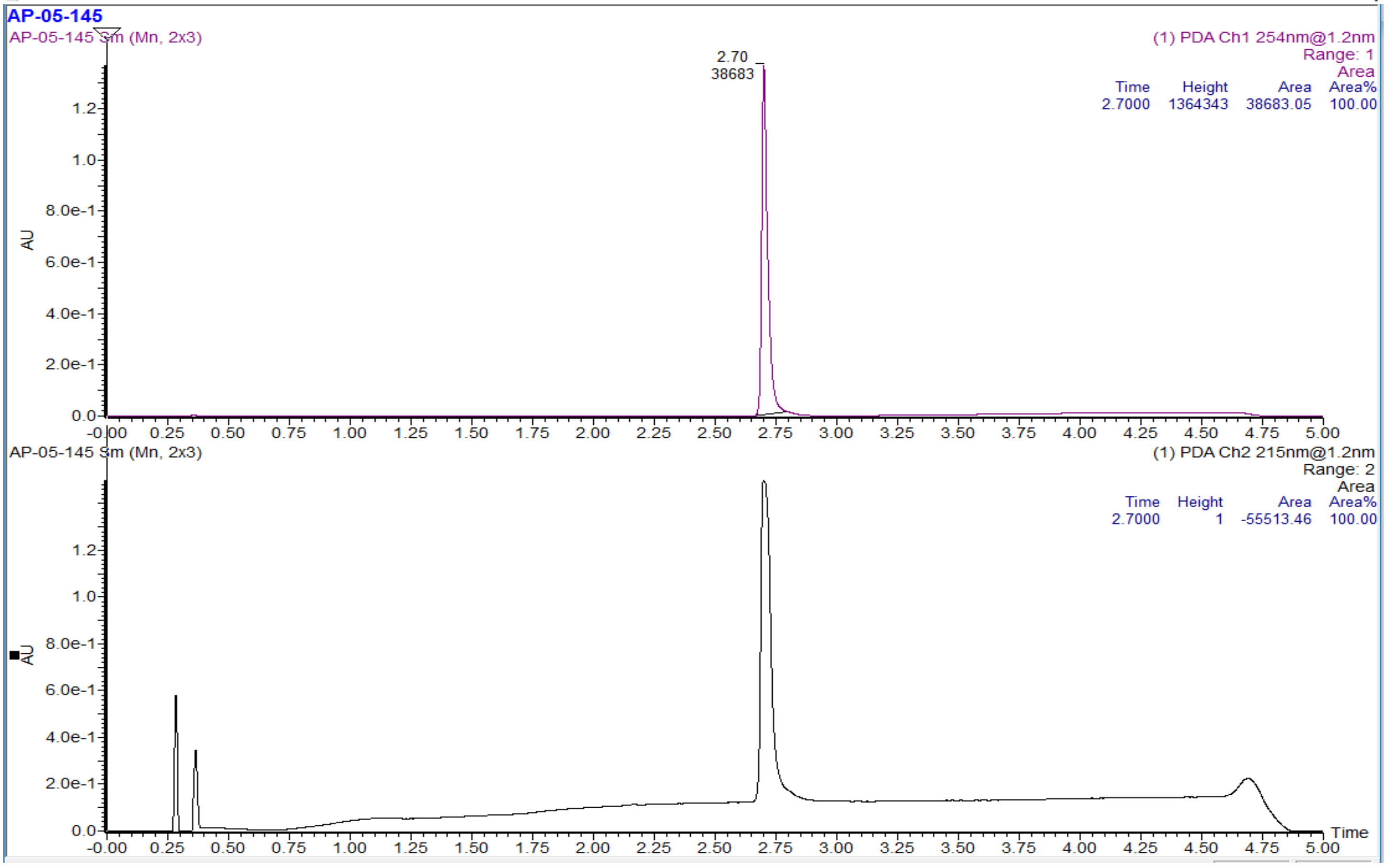

AP-05-145
AP-05-145 Sm (Mn, 2x3)
(1) PDA Ch1 254nm@1.2nm
Range: 1
Time Height Area Area%
2.7000 1364343 38683.05 100.00
2.70
38683
AU
(1) PDA Ch2 215nm@1.2nm
Range: 2
Time Height Area Area%
2.7000 1 -55513.46 100.00
Time
