# Supplementary material for: Molecular glues that inhibit deubiquitylase activity and inflammatory signaling
Source: Nat Struct Mol Biol. 2025 Mar 17;32(9):1812–24. doi: 10.1038/s41594-025-01517-5 (PMC7617869; doi:10.1038/s41594-025-01517-5)

Source Data Extended Data Figure 7

Extended Data Fig. 7a (*left*)

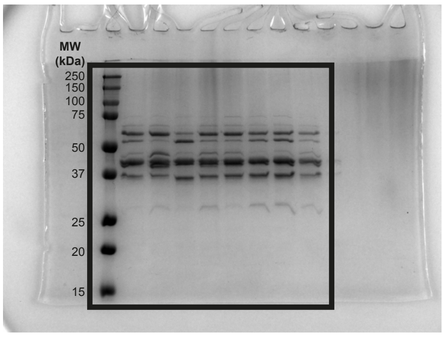

Extended Data Fig. 7a (*middle*)

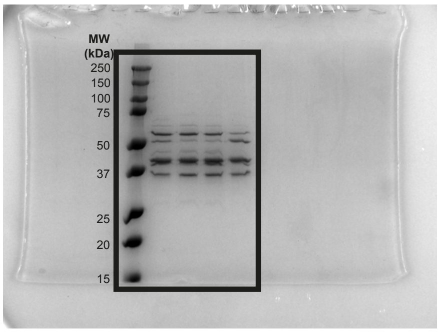

Extended Data Fig. 7a (*right*)

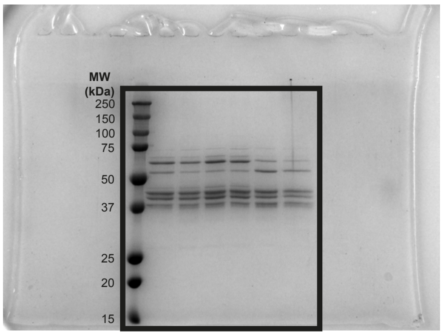

Supplement: Supplementary file 13 — Uncropped gels. [file 41594_2025_1517_MOESM13_ESM.pdf]
