# Supplementary material for: Molecular glues that inhibit deubiquitylase activity and inflammatory signaling
Source: Nat Struct Mol Biol. 2025 Mar 17;32(9):1812–24. doi: 10.1038/s41594-025-01517-5 (PMC7617869; doi:10.1038/s41594-025-01517-5)

Source Data Extended Data Figure 8

Extended Data Fig. 8g

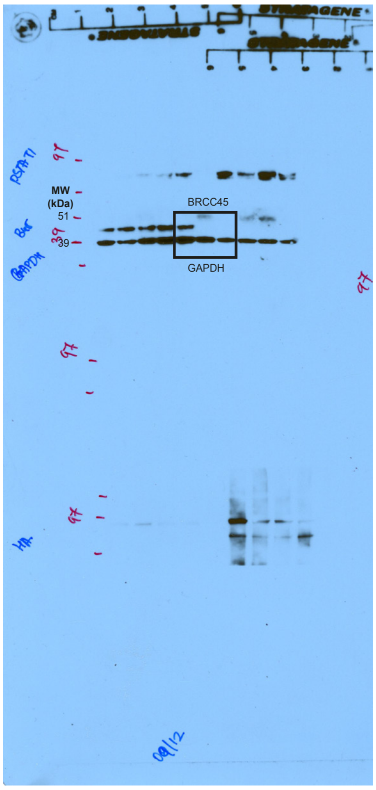

Extended Data Fig. 8h (*top*)

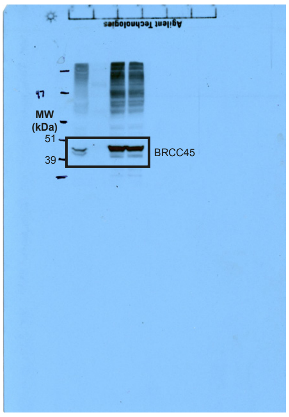

Extended Data Fig. 8h (*bottom*)

)

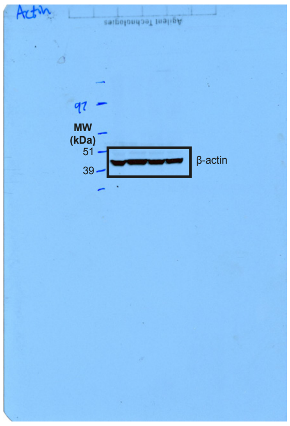

Extended Data Fig. 8i

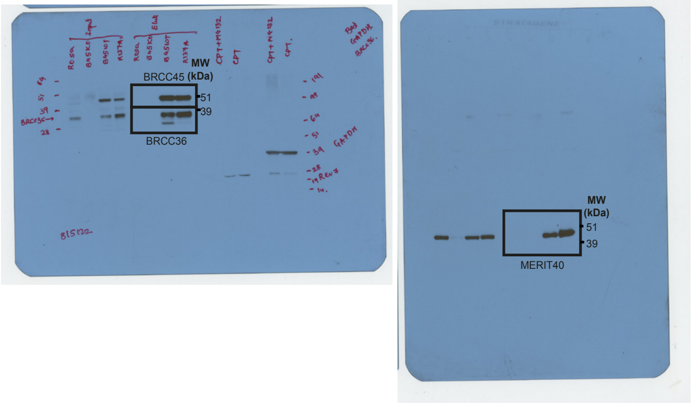

Extended Data Fig. 8j

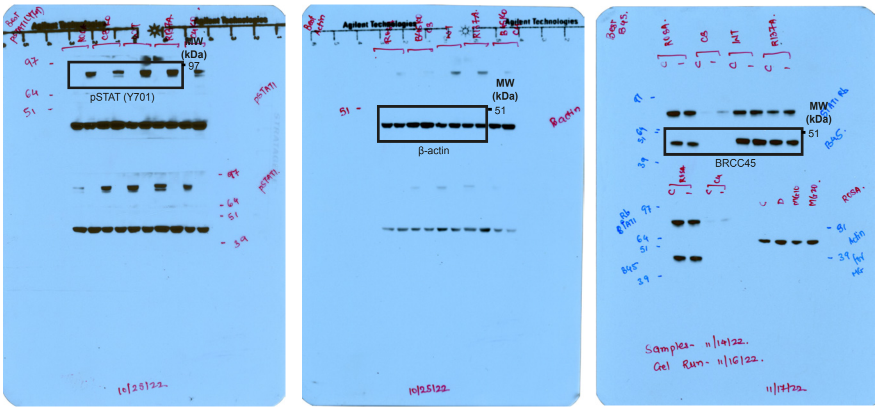

Extended Data Fig. 8n

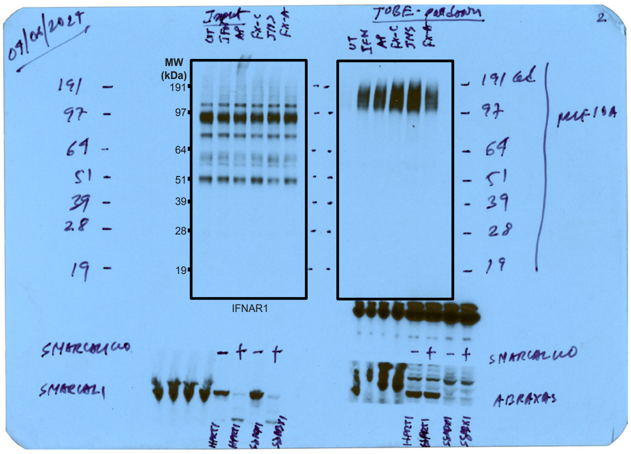

Extended Data Fig. 8o

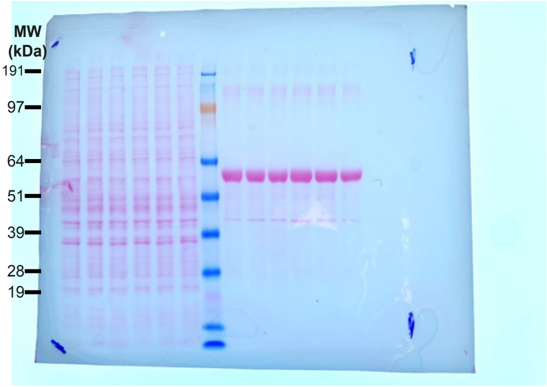

Supplement: Supplementary file 15 — Uncropped western blots. [file 41594_2025_1517_MOESM15_ESM.pdf]
