# Supplementary material for: Molecular glues that inhibit deubiquitylase activity and inflammatory signaling
Source: Nat Struct Mol Biol. Author manuscript; Available in PMC 2025 Jul 7. (PMC7617869; doi:10.1038/s41594-025-01517-5)
Supplement: Supplementary file 1 [file EMS206745-supplement-Reporting_Summary.pdf]

nature portfolio

Corresponding author(s): Elton Zeqiraj

Last updated by author(s): 08/01/2025

Reporting Summary

Nature Portfolio wishes to improve the reproducibility of the work that we publish. This form provides structure for consistency and transparency in reporting. For further information on Nature Portfolio policies, see our Editorial Policies and the Editorial Policy Checklist.

Statistics

For all statistical analyses, confirm that the following items are present in the figure legend, table legend, main text, or Methods section.

n/a	Confirmed	
☐	☒	The exact sample size (*n*) for each experimental group/condition, given as a discrete number and unit of measurement
☐	☒	A statement on whether measurements were taken from distinct samples or whether the same sample was measured repeatedly
☐	☒	The statistical test(s) used AND whether they are one- or two-sided<br>*Only common tests should be described solely by name; describe more complex techniques in the Methods section.*
☒	☐	A description of all covariates tested
☒	☐	A description of any assumptions or corrections, such as tests of normality and adjustment for multiple comparisons
☐	☒	A full description of the statistical parameters including central tendency (e.g. means) or other basic estimates (e.g. regression coefficient) AND variation (e.g. standard deviation) or associated estimates of uncertainty (e.g. confidence intervals)
☐	☒	For null hypothesis testing, the test statistic (e.g. *F*, *t*, *r*) with confidence intervals, effect sizes, degrees of freedom and *P* value noted<br>*Give P values as exact values whenever suitable.*
☒	☐	For Bayesian analysis, information on the choice of priors and Markov chain Monte Carlo settings
☒	☐	For hierarchical and complex designs, identification of the appropriate level for tests and full reporting of outcomes
☒	☐	Estimates of effect sizes (e.g. Cohen's *d*, Pearson's *r*), indicating how they were calculated

Our web collection on statistics for biologists contains articles on many of the points above.

Software and code

Policy information about availability of computer code

Data collection	Cryo-EM data were collected using ThermoFisher EPU software (v3.5.1). Negative stain EM data were collected using ThermoFisher EM imaging and analysis (TIA). Mass photometry data were collected using Refeyn AcquireMP (v2.2.0).
Data analysis	Cryo-EM data were analysed using: Relion (v3.0, v3.1.1), cryoSPARC (v4.2.1), crYOLO (v1.6.1), MotionCor2 (v1.2.1), gCTF (v1.18), CTFFIND (v4.1), Coot (v0.9.1-pre), Phenix(v1.20). Mass spectrometry data were analysed using MassLynx (v4.1). HDX data were analysed using PLGS (v3.0.2), and DynamX (v3.0.0) software. Mass photometry data were analysed using Refeyn DiscoverMP (v2.2.0). Native mass spectrometry data were processed with QualBrowser (v4.2.28.14) and UniDec (v5.0.0). Statistical analysis was performed using GraphPad Prism (v9.0, v9.5.1, v10.1.0, v10.3.1). Cryo-EM maps were visualised in Chimera (v1.12) and ChimeraX (v.1.2.3). Sequence alignments were performed using MUSCLE and edited using ALINE (v1.0.025). Spectral shift assays were carried out using DI.Control software (v2.1.1) and analysed using DI.Screening Analysis software (v2.1.1). Gene expression heatmaps were generated in R (v4.0.2) and RStudio (v1.3.1093).

For manuscripts utilizing custom algorithms or software that are central to the research but not yet described in published literature, software must be made available to editors and reviewers. We strongly encourage code deposition in a community repository (e.g. GitHub). See the Nature Portfolio guidelines for submitting code & software for further information.

Data

Policy information about availability of data

All manuscripts must include a data availability statement. This statement should provide the following information, where applicable:

- Accession codes, unique identifiers, or web links for publicly available datasets
- A description of any restrictions on data availability
- For clinical datasets or third party data, please ensure that the statement adheres to our policy

Cryo-EM maps have been deposited in the Electron Microscopy Data bank under the accession codes EMD-17980 and EMD-18009. Model coordinates have been deposited in the Protein Data Bank under the accession codes 8PVY, 8PY2. HDX data are available via ProteomeXchange (identifier: PXD044584). PDB models 6H3C, 6R8F, 2ZNV, 5J0G, 4ONN, 4ONM, and 3RZ3 were also used for model building or figure generation.

Research involving human participants, their data, or biological material

Policy information about studies with human participants or human data. See also policy information about sex, gender (identity/presentation), and sexual orientation and race, ethnicity and racism.

Reporting on sex and gender	Sex and gender details are reported in Supplementary Table 2. The higher proportion of women in our cohort reflects the well-documented female predominance of systemic sclerosis (SSc), which is consistent with its epidemiological profile. Furthermore, this was a cohort of incident cases of consecutive patients enrolled in our centre, which minimizes selection bias and ensures that the population reflects real-world clinical practice.<br><br>Regarding the distribution of disease subsets, the smaller number of patients with diffuse cutaneous SSc compared to those with limited cutaneous SSc is partly due to random variation and higher prevalence of the limited subset but also reflects a deliberate effort to minimise the potential interference of immunosuppressive therapies in our analysis. Diffuse cutaneous SSc patients are commonly treated with strong immune suppression, and we aimed at reducing such confounding factors.
Reporting on race, ethnicity, or other socially relevant groupings	*Please specify the socially constructed or socially relevant categorization variable(s) used in your manuscript and explain why they were used. Please note that such variables should not be used as proxies for other socially constructed/relevant variables (for example, race or ethnicity should not be used as a proxy for socioeconomic status).*<br>*Provide clear definitions of the relevant terms used, how they were provided (by the participants/respondents, the researchers, or third parties), and the method(s) used to classify people into the different categories (e.g. self-report, census or administrative data, social media data, etc.)*<br>*Please provide details about how you controlled for confounding variables in your analyses.*
Population characteristics	SSc patient samples were taken from the Leeds Scleroderma cohort, a tertiary centre which treating patients from the Yorkshire and the Humber area.
Recruitment	Due to the nature of collecting fresh blood and PBMCs on a weekly basis, patients were randomly selected from the weekly clinics based on those attending clinic.
Ethics oversight	All participants enrolled provided written informed consent according to a protocol approved by the Medicine and Healthy Regulatory agency (STRIKE NRES-011NE to FDG, IRAS 15/NE/2011). Reviewing body: North East - Newcastle & North Tyneside 2 Research Ethics Committee. Sponsor: University of Leeds.

Note that full information on the approval of the study protocol must also be provided in the manuscript.

Field-specific reporting

Please select the one below that is the best fit for your research. If you are not sure, read the appropriate sections before making your selection.

☒ Life sciences ☐ Behavioural & social sciences ☐ Ecological, evolutionary & environmental sciences

For a reference copy of the document with all sections, see nature.com/documents/nr-reporting-summary-flat.pdf

Life sciences study design

All studies must disclose on these points even when the disclosure is negative.

Sample size	Cryo-EM datasets were collected for a total of approximately 5 days instrument time. Sample size for cryo-EM data was arbitrarily chosen depending on sample, instrument availability and the number of particles required to obtain a structure at sufficient resolution to identify the inhibitor binding site.
Data exclusions	During cryo-EM processing, particles are omitted at the 2D or 3D classification stage in line with standard practice. Full details of particle classification are provided in the manuscript.
Replication	Triplicate repeats were formed for in vitro DUB activity assays, RT-qPCR experiments, mass photometry, and HDX-MS. For some in vitro DUB activity assays, two repeats in duplicate were performed. The number of repeats for each assay are outlined in the Figure legends. Biological replicate n numbers are described in the text. Where required, cross-validation was perfomed by rational mutagenesis, suitable controls and

orthogonal assays. In PBMC experiments, healthy and patient donors were used, 3 healthy donors were selected for triplicate biological analysis and 20 SSc patients were selected. A higher number of patient samples was selected to account for heterogeneous populations within the variable disease of SSc.

Randomization

No randomization during allocation into experimental groups was performed. Covariates were controlled by the addition of inactive control compounds as negative controls, and site-specific mutants in enzyme activity assays and cell signalling experiments.

Blinding

Structure determinations were performed without blinding. The sample identity was known to the investigators as this was required for appropriate interpretation of electron microscopy maps and model building. For cell biology experiments, blinding was not required and it was necessary for the investigator to know the sample identity.

Reporting for specific materials, systems and methods

We require information from authors about some types of materials, experimental systems and methods used in many studies. Here, indicate whether each material, system or method listed is relevant to your study. If you are not sure if a list item applies to your research, read the appropriate section before selecting a response.

Materials & experimental systems

n/a	Involved in the study
☐	☒ Antibodies
☐	☒ Eukaryotic cell lines
☒	☐ Palaeontology and archaeology
☒	☐ Animals and other organisms
☒	☐ Clinical data
☒	☐ Dual use research of concern
☒	☐ Plants

Methods

n/a	Involved in the study
☒	☐ ChIP-seq
☒	☐ Flow cytometry
☒	☐ MRI-based neuroimaging

Antibodies

Antibodies used

1. Anti-BRCC45 (Abcam, #ab177960) (1:1000 dilution)
2. Anti-GAPDH (Cell Signaling Technology, #2118S) (1:1000 dilution)
3. Anti-Merit40 (Cell Signaling Technology, #12711S) (1:1000 dilution)
4. Anti-BRCC36 (Abcam, #ab108411) (1:1000 dilution)
5. Anti-Phospho-Stat1 (Tyr701) (Cell Signaling Technology, #9167S) (1:1000 dilution)
6. Anti-beta Actin (Santa Cruz Biotechnology, SC-#47778) (1:1000 dilution)
7. Anti-Interferon alpha/beta receptor 1 antibody (Abcam #ab124764)
8. Human IFN-alpha/beta R1 antibody (R&D Systems, #MAB245) (2.5 μg/10^6 cells)
9. Mouse IgG1 Isotype control (R&D Systems, #MAB002) (2.5 μg/10^6 cells)
10. Human IFN-alpha / beta R1 APC-conjugated Antibody (Biotechne, FAB245A) (1 μg/mL)
11. Mouse IgG1 APC-conjugated Antibody (Biotechne, #IC002A) (1 μg/mL)
12. Amersham ECL Rabbit IgG, HRP-linked whole Ab (#NA934) (1:4000 dilution)
13. Amersham ECL Mouse IgG, HRP-linked whole Ab (#NA931) (1:4000 dilution)
14. Biotin-SP-conjugated AffiniPure Donkey Anti-mouse IgG (H+L) (Jackson Immunoresearch Laboratories #715-065-150) (1:750 dilution)
15. R-Phycoerythrin Streptavidin (Jackson Immunoresearch Laboratories, #016-110-084) (1:1000 dilution)

Validation

Validation of all the commercially available antibodies can be found on their respective websites.
1. https://www.abcam.com/products/primary-antibodies/brcc45bre-antibody-epr11858-ab177960.html
2. https://www.cellsignal.com/product/productDetail.jsp?productId=2118&utm_medium=b2b&utm_campaign=general
3. https://www.cellsignal.com/products/primary-antibodies/merit40-d7y5k-rabbit-mab/12711
4. https://www.abcam.com/products/primary-antibodies/brcc36-antibody-epr4366-ab108411.html
5. https://www.cellsignal.com/products/primary-antibodies/phospho-stat1-tyr701-58d6-rabbit-mab/9167
6. https://www.scbt.com/p/beta-actin-antibody-c4
7. https://www.abcam.com/en-gb/products/primary-antibodies/interferon-alpha-beta-receptor-1-antibody-epr6244-ab124764210
8. https://www.rndsystems.com/products/human-ifn-alpha-beta-r1-antibody-85228_mab245
9. https://www.rndsystems.com/products/mouse-igg-1-isotype-control_mab002
10. https://www.bio-techne.com/p/antibodies/human-ifn-alpha-beta-r1-apc-conjugated-antibody-85228_fab245a
11. https://www.bio-techne.com/p/isotype-controls/mouse-igg-1-apc-conjugated-antibody_ic002a
12. https://www.fishersci.co.uk/shop/products/100ul-amersham-ecl-rabbit-igg-hrp-linked-whole-ab/15417424
13. https://www.fishersci.co.uk/shop/products/1ml-amersham-ecl-mouse-igg-hrp-linked-whole-ab-f/15407424
14. https://www.jacksonimmuno.com/catalog/products/715-065-150
15. https://www.jacksonimmuno.com/catalog/products/016-110-084

Eukaryotic cell lines

Policy information about cell lines and Sex and Gender in Research

Cell line source(s)	Sf9 and Tni cells were used for protein expression and were obtained from Invitrogen (ThermoFisher). MCF10A cells were purchased from ATCC (https://www.atcc.org/products/crl-10317).
Authentication	No authentication of Sf9 or Tni cell lines were performed in our laboratory.
Mycoplasma contamination	For Sf9 and Tni cells, mycoplasma testing is carried out every 2 months, no mycoplasma contamination was detected. For the MCF10A cell lines, mycoplasma testing was carried out for the first three passages and no mycoplasmas contamination was detected.
Commonly misidentified lines (See ICLAC register)	Commonly misidentified cell lines were not used.
