# Supplementary material for: Molecular glues that inhibit deubiquitylase activity and inflammatory signaling
Source: Nat Struct Mol Biol. Author manuscript; Available in PMC 2025 Jul 7. (PMC7617869; doi:10.1038/s41594-025-01517-5)
Supplement: Supplementary file 2 [file EMS206745-supplement-supp_11.pdf]

	Dimer fraction				
[Inhibitor (μM)]		JMS-175-2			FX-171-C
10000	0.35	0.32	0.21	0.31	0.43
5000	0.32	0.27	0.22	0.24	0.56
2500	0.41	0.08	0.22	0.36	0.38
1250	0.29	0.08	0	0.29	0.39
625	0.14	0.06	0.2	0.06	0.22
312.5	0.06	0	0.08	0.08	0.13
156	0.13	0.08	0.06	0.06	0.14
80	0	0.06	0.05	0.03	0.18
40	0.04	0.07	0	0	0.17
20	0	0.18	0	0	0.07
0	0.05	0	0.03	0	0.12

0.4
0.3
0.35
0.17
0.14
0.17
0.03
0
0.11
0.06
0.07
