# Supplementary material for: Molecular glues that inhibit deubiquitylase activity and inflammatory signaling
Source: Nat Struct Mol Biol. Author manuscript; Available in PMC 2025 Jul 7. (PMC7617869; doi:10.1038/s41594-025-01517-5)
Supplement: Supplementary file 3 [file EMS206745-supplement-supp_12.pdf]

[FX-171-C (μM)]		HsBRISC			HsBRISCΔLo[illegible]
199.9216	0.0311856	0.1486176	0.1231551	0.0474499	-0.079079
125.99038	0.162229	0.2082059	0.2077005	0.0950334	-0.008285
79.760148	0.2227304	0.2163529	0.143877	0.1824737	0.1266371
50.146965	0.0569106	0.1394706	0.2566845	0.2434575	0.2223085
31.499918	0.1886856	0.2420588	0.2131283	0.4543457	0.3801332
19.960041	0.1876355	0.174	0.161016	0.5066858	0.5049945
12.783867	0.5596206	0.3111765	0.2491711	0.6513849	0.4754717
7.9936445	0.4918699	0.2335882	0.2962567	0.6972302	0.709212
5.1973933	0.6151762	0.3188235	0.3117647	0.8896848	0.7130966
3.1590005	0.4027778	0.2970588	0.328877	0.8204394	0.9567148
1.9996009	0.7381436	0.4197059	0.4125668	0.9331423	0.8962264
1.2598414	0.7743902	0.5964706	0.5179144	0.8619866	0.9567148
0.7999365	0.820122	0.7670588	0.7221925	0.9837631	0.981687
0.4999749	0.8336721	0.8541176	0.8390374	0.9904489	0.9306326
0.3199899	0.8780488	1.045	0.8398396	1.2239733	0.9478357
0.199996	0.9109079	1.1	0.9713904	0.8409742	1.0166482
0.1199985	0.8990515	1.2973529	1.1131016	1.0176695	0.9561598
0.0799994	0.9329268	1.2152941	0.9839572	0.9355301	0.9389567
0.0599997	1.0193089	1.1785294	1.0066845	1.0157593	0.9467259
0.0399999	0.8326558	1.2123529	1.0320856	0.904489	0.9217536
0.02	0.8163957	1.3588235	1.0272727	1.0329513	0.8668147
0.013	0.9661247	1.2585294	1.0283422	0.8758357	0.8940067
0.013	1.1063686	0.9508824	0.8874332	0.9594078	0.8823529
0	1	1	1	1	1

		**Normalised V0**				
**op**		**MmBRISC**			**DrBRISCΔNΔC**	
-0.02609	0.0753169	0.0224259	-0.019681	0.3129969	0.2853129	0.3043376
-0.024848	0.1198669	0.0863032	0.0743528	0.4738532	0.365118	0.426214
0.1423536	0.2258555	0.1417717	0.1329922	0.6671254	0.5065568	0.3962753
0.1232104	0.2436629	0.1321976	0.1837447	0.6952599	0.6277632	0.7138142
0.3980477	0.3403042	0.2592504	0.2598435	0.7519878	0.7817535	0.7739274
0.4112256	0.4312421	0.3645656	0.4124022	0.6504587	0.8984638	0.8710514
0.6301518	0.5773131	0.5291312	0.5270921	0.9357798	1.0243537	0.883074
0.6659436	0.6821926	0.6463373	0.6195063	0.9454128	0.9127014	0.9516737
0.7640998	0.8710393	0.8367973	0.8088501	0.6448012	0.9953166	0.8470061
0.8834056	0.9249049	0.9175468	1.0225768	1.0415902	0.9998127	0.8592645
0.9251627	0.9762357	0.9243612	0.8829019	0.9181957	0.9951293	0.8213107
0.7933839	0.8979721	1.0057922	0.922637	0.9862385	1.092544	0.9450731
0.8145336	0.9803549	1.0003407	0.9196267	1.0090214	0.9333084	0.8976898
1.0146421	0.9489861	0.9710392	0.9656833	0.9714067	1.0520794	0.9507308
0.89859	0.9942966	1.0160136	0.948224	0.9730887	1.1657924	0.8821311
0.9354664	1.0570342	1.0821124	0.9581577	1.0444954	1.0230423	0.9962282
0.8275488	1.0662231	1.0132879	0.9623721	0.9666667	1.1183964	0.9768977
0.7814534	1.0196451	1.0078365	1.0168573	1.2573394	1.0717497	1.0139085
0.8736443	1.0611534	0.9751278	0.8991571	1.0001529	1.042338	0.9509665
0.9322126	0.893853	0.913799	0.846177	0.9720183	1.0863619	0.9938708
0.9186551	1.0126743	1.0197615	0.9048766	0.9073394	0.9512926	0.9153701
0.9224512	0.8922687	0.932879	0.8269115	0.9830275	1.0453353	1.0073079
0.8649675	1.0475285	1.0344123	0.9087899	1.0755352	0.9046459	1
1	1	1	1	1	1	1

Cf	BRISC∆N	∆C
1.0479369	0.9667093	0.9356775
0.806432	1.0083227	0.9331046
0.9478155	0.9801536	1.2941681
0.8822816	1.099872	0.9082333
0.9199029	1.1370038	0.9150943
1.0479369	1.1286812	1.0394511
1.0303398	1.0448143	0.7909091
0.9320388	1.0640205	1.2692967
0.9696602	1.084507	0.6282161
0.9417476	1.1331626	0.9365352
0.9132282	0.9122919	0.8399657
0.9211165	1.1222791	1.0145798
0.848301	0.918694	0.8447684
0.8932039	0.9705506	1.1603774
0.8853155	0.9321383	0.7921098
0.9393204	1.0294494	0.8090051
0.9120146	0.9545455	0.690223
0.9599515	1.0211268	0.8317324
0.9599515	0.9545455	0.8432247
0.8373786	1.0851472	0.9219554
0.8822816	0.9398207	0.9125214
0.8962379	0.9603073	0.9451115
1.0097087	0.9065301	0.9614065
1	1	1
