# Supplementary material for: Molecular glues that inhibit deubiquitylase activity and inflammatory signaling
Source: Nat Struct Mol Biol. Author manuscript; Available in PMC 2025 Jul 7. (PMC7617869; doi:10.1038/s41594-025-01517-5)
Supplement: Supplementary file 4 [file EMS206745-supplement-supp_14.pdf]

[BRISC (nM)]		WT		BRCC36 T128	
40	1067	1241	1530	263.7	269.7
20	604.3	1115	999.5	159.1	169.2
10	433.6	509.8	492.5	82.82	98.95
5	252	295.7	277.5	38.19	33.52
2.5	128.4	75.45	166.1	14.18	0.6624
1.25	73.8	48.72	88.59	1.329	0.02481
0	-7.273	-24.29	-17.2	-9.221	-16.45

[BRISC (nM)]		WT		Abraxas2 I133	
40	1209	972	783.8	1065	1029
20	603	680.7	659.2	818.3	946
10	417.8	551	633.7	611.3	559.3
5	240.5	325.2	323.9	410.1	361
2.5	161.2	163.8	183.8	288.2	202.8
1.25	70.74	82.11	125.4	174.7	143.7
0	-10.2	-13.22	-12.98	-5.856	-6.711

[BRISC (nM)]		WT		BRCC45 R137	
40	1356	1562	977.5	1405	1817
20	945.1	1152	923.4	1158	1199
10	718.1	784	922.1	832.4	888
5	469.4	486.3	513.2	570.3	610.5
2.5	251.3	301.2	325.9	356.6	343.5
1.25	150.3	198.8	181.3	218.4	228.6
0	1.048	17.94	-25.32	7.721	1.409

Initial veloci

**3P**		**BRCC36 W130A**			**BRCC36 I158K**	
264.3	2187	2281	2915	47.15	50.97	55.02
159.8	1697	1296	1628	14.82	34.38	37.4
88.22	990.4	1264	912.6	8.897	10.58	8.163
40.74	758.2	623.5	637.1	-4.444	12.8	-2.541
5.379	362.6	396.3	295.1	2.54	-8.075	-14.17
3.544	224.1	207.7	187.1	-15.36	-7.072	-19.08
-22.89	-33.06	-15.81	-11.62	-13.74	-7.81	-23.1
**3W**		**Abraxas2 T135K**			**Abraxas2 T140A**	
1206	1203	1279	1326	1537	1339	1258
929.3	686.7	1022	941.3	674.3	1202	621.6
458.9	454	578.3	613.4	296.1	685.7	316.3
363.2	318.6	442.4	376.1	246.2	364.1	223.9
186.8	209.5	273.7	270.5	148.1	245.8	106.9
120.5	124.1	142.6	155	78.12	127.6	57.68
-12.73	-7.474	-5.177	-4.342	-13.82	-16.98	-7.851
**7A**		**BRCC45 F140A**			**BRCC45 R137A+F140A**	
1548	1051	1269	1598	1530	2146	1744
1166	1437	1115	1349	1273	1359	1467
968.1	1272	947.8	1075	920.6	900.3	1137
645.7	787.2	618.6	783.7	549.5	600.5	654.2
363.5	448.4	354	419.4	341.7	324.3	401
257.8	301.4	237.9	277.6	214.8	267.8	245.3
-10.94	28.29	-7.094	-14.22	26.83	-10.97	-9.509

ity (nM/min)

BRCC36 R167A			BRCC36 L169R			Bl
1722	1627	1721	1334	1553	2228	2138
1217	1139	1777	1361	1825	2085	1694
1014	873.8	815.6	1200	1199	1247	1280
686.1	611.4	580.8	1004	983.6	839.4	853.5
352.1	344.4	294.8	542.3	578.1	574.4	451.7
216.5	179.9	166.9	353.4	361.2	317	289.4
-9.408	-8.198	-6.341	-8.181	-11.47	-15.76	-7.541

BRCC45 C245A			BRCC45 D248R		
1405	2395	1425	1856	2089	2388
1453	1699	1719	1287	1364	1493
941	1085	1098	880.5	801.2	1059
647.1	643.1	709.9	519.6	459.7	553.1
371.4	372.9	415.6	306.1	289.6	271
208.1	225.6	255.3	174.1	174.1	137.4
13.87	-9.667	-15.43	7.473	-20.61	-15.82

RCC36 L169A		BRCC36 L169W		
1751	2340	2546	2258	2109
1481	1695	1636	1548	1897
970.6	1236	1165	1126	1060
701.6	712.8	770.2	582.2	559.7
387	393.9	416.1	318.3	343.9
219.1	223.5	90.15	197.4	184.6
-11.67	-14.84	-9.765	-17.65	-7.886
