# Supplementary material for: Molecular glues that inhibit deubiquitylase activity and inflammatory signaling
Source: Nat Struct Mol Biol. Author manuscript; Available in PMC 2025 Jul 7. (PMC7617869; doi:10.1038/s41594-025-01517-5)
Supplement: Supplementary file 5 [file EMS206745-supplement-supp_16.pdf]

**Biological replicate 1**			**Blot**	
		Mean	IntDen	RawIntDen
**DMSO**	1	120.482	909641	909641
**AP-5-145**	2	130.366	984263	984263
**FX-171-C**	3	153.776	1161009	1161009
**JMS-175-2**	4	160.529	1211992	1211992
**FX-171-A**	5	122.155	922270	922270
**background**	6	57.92	437294	437294

**Biological replicate 2**			**Blot**	
		Mean	IntDen	RawIntDen
**DMSO**	1	118.053	832273	832273
**AP-5-145**	2	126.343	890715	890715
**FX-171-C**	3	129.75	914735	914735
**JMS-175-2**	4	140.9	993345	993345
**FX-171-A**	5	113.378	799312	799312
**background**	6	77.601	547088	547088

**Biological replicate 3**			**Blot**	
		Mean	IntDen	RawIntDen
**DMSO**	1	87.504	537627	537627
**AP-5-145**	2	100.076	614865	614865
**FX-171-C**	3	106.409	653778	653778
**JMS-175-2**	4	114.675	704566	704566
**FX-171-A**	5	95.594	587332	587332
**background**	6	57.068	350625	350625

**DMSO**	**AP-5-145**	**FX-171-C**	**JMS-175-2**	**FX-171-A**
1	1.157987277	1.532176081	1.640117004	1.026741472
1	1.204934	1.289158	1.564793	0.884431
1	1.413063	1.621139	1.892726	1.265804

Background substracted Fold Change

62.562	1
72.446	1.157987277
95.856	1.532176081
102.609	1.640117004
64.235	1.026741472
0	0

Background substracted Fold Change

40.452	1
48.742	1.204934243
52.149	1.28915752
63.299	1.564792841
35.777	0.88443093
0	0

Background substracted Fold Change

30.436	1
43.008	1.413063477
49.341	1.62113944
57.607	1.89272572
38.526	1.265803654
0	0
