# Supplementary material for: Molecular glues that inhibit deubiquitylase activity and inflammatory signaling
Source: Nat Struct Mol Biol. Author manuscript; Available in PMC 2025 Jul 7. (PMC7617869; doi:10.1038/s41594-025-01517-5)
Supplement: Supplementary file 6 [file EMS206745-supplement-supp_17.pdf]

gene	JMS-175-2_HC1	JMS-175-2_HC2	JMS-175-2_HC3	AP-5-145_HC1
ADAR	-1.488587726	-0.333820558	-0.008200031	-0.08177522
BST2	-0.085941765	-1.597253597	-2.57710907	0.149920742
CASP1	-1.294270183	-1.029545015	-2.745366487	-0.124935676
CD86	-0.64261137	-1.639475202	-2.556616675	0.002980136
CXCL10	-6.186761658	-1.53780549	-3.495082963	0.301288848
DDX58	-1.851351162	-0.821198994	-2.710115466	0.522104345
GBP1	0.09720766	-0.449458172	-1.939610645	-0.113377833
IFI16	-0.175050033	0.866334135	0.334138662	-0.097605526
IFI6	-2.955996208	-0.54320504	-2.689437513	-0.525808702
IFIH1	-3.260609239	-0.70410107	-0.908589543	0.510419268
IFIT1	-1.070672698	0.87852547	-0.672208003	0.087792809
IFIT2	-1.436519106	0.029482062	0.25350559	0.571431401
IFIT3	-4.323755511	0.103842657	0.051434185	0.183552996
IFITM1	-2.905710272	-0.219540104	0.428212423	-0.125961766
IFITM2	1.300095957	-1.146670875	0.156983652	0.391376464
IFITM3	0.882764131	-0.738147701	-1.006658174	0.448110637
IRF1	-0.274214229	1.56951494	0.582739467	0.173892278
IRF7	0.126010665	0.429875833	-0.57795264	-0.065586829
IRF9	0.953285607	0.165433775	-0.126524698	0.291465113
ISG15	-1.258632073	-1.625371905	-0.881028377	-0.221048566
ISG20	-0.375970352	-0.827306184	-1.540814657	0.361824155
MX1	-3.694269178	-1.51822701	-2.394238483	0.309247328
MX2	-2.830119073	-0.964417904	-2.371548377	-0.801492566
MYD88	-0.873035273	-0.552056105	-1.228005578	0.040490233
NMI	-0.671293172	0.190663996	-0.598647476	0.325235335
OAS1	0.107518045	-1.171060787	-2.39289126	0.151439551
OAS2	-1.514620068	-1.3551239	-1.162928373	-0.185599562
PML	1.88536966	0.764907829	1.236976356	0.297329167
SOCS1	1.327848531	-1.0924863	-0.842549773	0.935180038
STAT1	-0.660307948	-0.80911578	-1.057255253	-0.096452442
STAT2	-2.070324795	-1.602179627	-2.2508771	0.377518711
STAT3	-0.541311874	-0.272152706	-0.061486179	0.253009632
TAP1	-1.623517458	-1.02370529	-0.432204762	0.503147049
TNFSF10	-0.037405635	-1.430639467	-1.67497694	0.633692871

AP-5-145_HC2	AP-5-145_HC3	FX-171-C_HC1	FX-171-C_HC2	FX-171-C_HC3
-0.144692623	0.202303201	-0.723506897	0.024578377	0.532293988
0.524350339	-1.144948837	0.414058065	0.540797338	-0.974751051
0.328858921	-0.274153255	-0.203763353	1.084358921	-0.256092469
-0.183359266	0.159993557	0.598990459	-0.822089267	-0.065356656
-1.144517555	0.395517269	1.361020171	0.951079445	2.017287056
-0.015960058	-0.798483234	-0.719844332	-0.232246059	-0.632195448
0.295758764	-0.237781412	0.76181349	0.196713763	-0.220521626
0.330360071	-0.307412105	-0.215150203	0.33047507	0.381887681
0.634155896	-0.408510281	-1.004725379	1.626423895	-0.074951494
-0.427912135	-0.264999311	-1.142792409	-0.381838135	0.137858475
0.144502406	-0.265504771	-1.083406868	-0.190548595	-0.789141984
-0.202648002	-0.632520178	-0.328677276	-0.625617002	-0.852249392
-0.229235407	0.016119417	-0.779213681	-0.368103407	0.079057203
-0.589546169	0.504930655	-0.250196443	-1.332784169	0.012287442
-0.342358939	-0.152636116	0.016377787	-1.28715094	-0.347657329
-0.021630765	-0.620092942	0.14512496	-0.013171766	0.398373845
0.369972875	-0.786936301	0.109437601	0.943573875	0.490359485
0.256345769	-0.234475408	-0.170177506	1.296098768	0.041372379
0.11971271	-0.536170466	-0.009269564	-0.42416729	0.108971321
0.282629031	-0.111463145	-0.769620243	-0.429841969	-0.234841359
-0.227243248	-0.216443425	-0.052817522	-0.814606249	-0.345329638
0.261187925	-0.830142251	-0.952423349	-0.597012075	0.513538536
0.662524031	-0.245719145	-1.596639243	1.367663031	0.839228641
0.31857483	-0.465064346	0.680956556	0.81499283	-0.052930559
0.073068932	-0.524543244	-0.428470342	0.292896932	-0.213762458
0.332239148	-0.665759028	0.026388874	0.583581148	0.056324759
-0.319652964	0.399940859	-0.494897239	-0.377191965	-0.033771354
-0.205699236	-0.145582412	0.50604749	0.279549764	0.700940374
-1.089098365	-0.694751541	1.892487361	0.018810635	-0.554219755
0.119742155	-0.031903021	-0.567944119	0.326375155	0.858062766
-0.445814691	-0.048822868	0.508670035	-0.680477692	0.701810919
-0.186042771	-0.105789947	-0.220361045	-0.146064771	0.14068584
-1.045955354	0.13536947	0.052481372	-0.183563354	-0.110545744
-0.747628532	-0.229573708	0.216689194	-0.625863532	0.160126079
