# Supplementary material for: Molecular glues that inhibit deubiquitylase activity and inflammatory signaling
Source: Nat Struct Mol Biol. Author manuscript; Available in PMC 2025 Jul 7. (PMC7617869; doi:10.1038/s41594-025-01517-5)
Supplement: Supplementary file 7 [file EMS206745-supplement-supp_2.pdf]

Supplementary Table 1 - HDX-MS Data Summary Table

Data Set	BRISC + DMSO	BRISC + FX-171-C
**HDX reaction details**	50 mM potassium phosphate, 200 mM NaCl, pD 7.5, 95 % D2O, 4ºC	50 mM potassium phosphate, 200 mM NaCl, pD 7.5, 95 % D2O, 4ºC
**HDX time course (min)**	0, 0.5, 1, 10, 60	
**HDX control samples**	Maximally-labelled controls were not performed.	
**Back exchange**	Not applicable	
**Number of peptides**	BRCC36: 110<br>Abraxas2ΔC: 71<br>BRCC45: 110<br>MERIT40ΔN: 89	BRCC36: 111<br>Abraxas2ΔC: 71<br>BRCC45: 109<br>MERIT40ΔN: 89
**Sequence coverage (%)**	BRCC36: 94.30<br>Abraxas2ΔC: 86.89<br>BRCC45: 88.37<br>MERIT40ΔN: 92.66	BRCC36: 94.30<br>Abraxas2ΔC: 86.89<br>BRCC45: 88.37<br>MERIT40ΔN: 92.66
**Average peptide length / Redundancy**	BRCC36: 9.87/3.64<br>Abraxas2ΔC: 11.76/3.60<br>BRCC45: 9.99/3.21<br>MERIT40ΔN: 9.21/3.42	BRCC36: 9.88/3.68<br>Abraxas2ΔC: 11.76/3.60<br>BRCC45: 10.00/3.19<br>MERIT40ΔN: 9.21/3.42
**Replicates (biological or technical)**	3 technical	3 technical
**Repeatability (average SD)**	BRCC36: 0.1185<br>Abraxas2ΔC: 0.1186<br>BRCC45: 0.0861<br>MERIT40ΔN: 0.0927	BRCC36: 0.0578<br>Abraxas2ΔC: 0.0623<br>BRCC45: 0.0620<br>MERIT40ΔN: 0.0584
**Significant differences in HDX (delta HDX > X D)**	Reference	99% CI in summed data
