# Supplementary material for: Molecular glues that inhibit deubiquitylase activity and inflammatory signaling
Source: Nat Struct Mol Biol. Author manuscript; Available in PMC 2025 Jul 7. (PMC7617869; doi:10.1038/s41594-025-01517-5)
Supplement: Supplementary file 8 [file EMS206745-supplement-supp_3.pdf]

[FX-171-C (μM)]	BRISC			
199.921599	0.0311856	0.1486176	0.1231551	0.7440395
125.990382	0.162229	0.2082059	0.2077005	0.9989038
79.7601479	0.2227304	0.2163529	0.143877	0.9819129
50.1469648	0.0569106	0.1394706	0.2566845	0.9345026
31.4999183	0.1886856	0.2420588	0.2131283	0.9912305
19.9600414	0.1876355	0.174	0.161016	0.9551932
12.7838675	0.5596206	0.3111765	0.2491711	1.0132913
7.99364453	0.4918699	0.2335882	0.2962567	0.9509455
5.19739325	0.6151762	0.3188235	0.3117647	0.9682105
3.15900052	0.4027778	0.2970588	0.328877	0.8584544
1.9996009	0.7381436	0.4197059	0.4125668	1.0416552
1.25984138	0.7743902	0.5964706	0.5179144	0.9788983
0.79993647	0.820122	0.7670588	0.7221925	0.959715
0.49997495	0.8336721	0.8541176	0.8390374	0.4557413
0.31998989	0.8780488	1.045	0.8398396	1.0016443
0.19999601	0.9109079	1.1	0.9713904	1.0187723
0.11999849	0.8990515	1.2973529	1.1131016	0.9409427
0.07999937	0.9329268	1.2152941	0.9839572	1.022746
0.0599997	1.0193089	1.1785294	1.0066845	1.0275418
0.03999987	0.8326558	1.2123529	1.0320856	1.0872842
0.01999996	0.8163957	1.3588235	1.0272727	1.1224993
0.01299998	0.9661247	1.2585294	1.0283422	1.0908468
0.01299999	1.1063686	0.9508824	0.8874332	1.1009866
0	1	1	1	1

				**Normalised V0**		
**ARISC**			**Trypsin**			**USP2**
0.5764004	0.7980119	0.4321911	0.4467054	0.4965628	1.0470373	0.8290398
0.9715752	0.9683234	0.6817085	0.8207364	0.673923	1.048259	0.979313
0.9177759	0.8967528	0.7444498	0.9554264	0.754583	1.0995724	0.8637783
0.8542707	0.966998	0.8122587	0.9452519	0.8817599	1.086744	1.0722092
0.9889074	0.9685885	0.9263996	1.0801841	0.8455545	1.0714722	0.9617486
0.8844981	0.9378396	0.9382239	0.9859496	0.9653987	1.2816127	0.8934426
0.9875208	0.9597084	1.1066602	1.0954457	0.9651696	1.1667685	1.0269321
0.8953134	0.9840954	1.0359556	1.1419574	1.0531622	1.2687844	0.9668228
0.9452302	1.0636183	1.0125483	1.1184593	1.0815765	1.2058644	0.9941452
0.9100111	0.9811796	0.9589768	1.0753391	1.1191567	1.32865	0.7564403
0.9796173	1.0145792	1.031612	1.1363857	0.9683776	1.2107514	0.9570648
0.9338602	0.9579854	1.0830116	0.9949128	1.0687443	1.2535125	0.9797034
0.9608985	1.0535454	0.9633205	1.092781	0.8806141	1.1667685	0.952381
0.9876595	0.9695162	0.9676641	1.1218508	0.9917507	1.3225412	0.7864949
0.9219357	1.067727	0.9473938	1.1031977	1.0213107	1.1753207	0.9406714
0.9437049	0.9098741	1.0482625	1.0750969	0.9569203	1.1624924	0.9867291
0.9459235	1.0337972	0.9090251	1.1017442	1.1372594	1.1563836	1.1202186
0.9475874	0.8942346	0.9908301	1.0850291	0.9855637	1.2620648	0.9465262
0.9352468	0.9673956	0.9939672	1.1736919	1.0048121	1.101405	1.0347385
0.984609	0.8910537	0.8957529	1.0721899	0.9550871	1.1808186	1.0542545
0.9660288	1.020941	0.9753861	1.0816376	1.0368928	1.1527184	1.0046838
0.9198558	0.9932406	1.027027	1.030281	1.0332264	1.144777	0.9761905
0.879645	1.0394964		1.1293605	1.2108158	1.06292	1.0886027
1	1	1	1	1	1	1

		AMSH*		HsBRCC36-Abraxas2		
0.8108478	0.8791541	0.889559	0.9451986	1.1273396	0.8107112	0.639201
0.8997265	1.097281	0.9973615	1.1121166	1.275234	1.0294883	0.9008739
0.9120328	1.0954683	0.9276291	1.1578683	1.111631	1.2751518	1.168789
0.8163172	1.1141994	0.9321523	1.2533937	1.2177473	1.1136167	0.9053683
0.9489517	1.1123867	1.038824	1.0020111	1.1493984	0.9767997	0.8461923
0.9216044	0.7794562	0.7410479	1.2800402	1.0337567	1.0626626	0.8933833
0.9175023	1.170997	0.9747456	1.2101559	1.0778743	0.9312663	0.7822722
0.7862352	1.1377644	1.0655861	1.1613876	1.1412099	1	1.011985
0.9284412	0.9975831	1.1059178	1.1603821	1.0534759	0.9453599	1.0299625
0.9480401	1.0809668	1.0644553	1.2182001	1.0541444	1.0318734	0.8521848
0.9503191	1.1401813	1.1500188	1.1910508	1.0573195	0.8883348	0.8901373
0.8377393	1.058006	1.09951	1.1427853	1.1923463	1.0058543	0.8037453
0.937557	1.2199396	0.889559	1.1860231	1.0157086	0.9325672	0.9280899
0.9402917	0.9945619	0.8997361	1.1623932	1.2762366	0.885516	0.9133583
0.9193254	1.0586103	1.0162081	1.1256913	1.0444519	1.0325239	1.0476904
0.9384686	1.1770393	1.0648323	1.2538964	1.0860628	0.937771	0.7275905
1.0154968	1.2356495	1.1074256	1.1422826	1.0890709	1.0542064	0.9805243
0.9639927	1.155287	1.0463626	1.0336853	1.2996324	1.0735039	0.9036205
0.8564266	1.1655589	0.9973615	1.025641	1.0203877	1.1192541	0.8586767
0.8345488	1.0755287	0.9687147	1.1176471	1.0218917	1.1006071	0.8289638
0.8910665	1.1015106	0.9958538	1.1221719	1.2999666	1.0082394	1.0204744
0.8523245	1.1450151	1.0354316	1.0889894	1.0344251	1.089549	1.0594257
0.8281677	0.9268882	0.9739917	0.7692308	1.0606618	0.9804857	1.0536829
1	1	1	1	1	1	1
