# Supplementary material for: Molecular glues that inhibit deubiquitylase activity and inflammatory signaling
Source: Nat Struct Mol Biol. Author manuscript; Available in PMC 2025 Jul 7. (PMC7617869; doi:10.1038/s41594-025-01517-5)
Supplement: Supplementary file 9 [file EMS206745-supplement-supp_4.pdf]

[FX-171-C (μM)]	WT			BRCC36 W13(	
200	-0.056281	-0.016297	0.0042245	0.1415236	-0.007729
100	0.0172844	0.1268761	0.1129566	0.2098712	0.2432087
50	0.0476834	0.2324607	0.0726254	0.4035944	0.2879921
25	0.0848134	0.2354276	0.2155869	0.5324571	0.288189
12.5	0.0438674	0.1770681	0.2379932	0.6754292	0.5915354
6.25	0.1300515	0.1609773	0.1849489	0.8320815	0.5661417
3.1	0.0678893	0.2540663	0.1668777	0.7966738	0.6612205
1.04	0.2670528	0.5033159	0.3779834	0.873927	0.782185
0.35	0.5423423	0.7232112	0.6751096	0.9667382	0.7732283
0.035	0.997426	0.7919721	0.9381393	0.9651288	0.9302165
0.0035	0.956242	0.7724258	0.9883098	0.9522532	0.9525591
0	1	1	1	1	1

[FX-171-C (μM)]	WT			Abraxas2 I133	
200	-0.056281	-0.016297	0.0042245	-0.017573	0.1299411
100	0.0172844	0.1268761	0.1129566	-0.05203	0.1559186
50	0.0476834	0.2324607	0.0726254	0.1012087	0.2459025
25	0.0848134	0.2354276	0.2155869	0.0541454	0.2117836
12.5	0.0438674	0.1770681	0.2379932	0.0953919	0.2465988
6.25	0.1300515	0.1609773	0.1849489	0.1792257	0.4617033
3.1	0.0678893	0.2540663	0.1668777	0.2779981	0.4702196
1.04	0.2670528	0.5033159	0.3779834	0.8517469	0.6089984
0.35	0.5423423	0.7232112	0.6751096	1.0485364	0.8211034
0.035	0.997426	0.7919721	0.9381393	1.1886686	0.9453669
0.0035	0.956242	0.7724258	0.9883098	1.1416431	0.8826995
0	1	1	1	1	1

[FX-171-C (μM)]	WT			BRCC45 R137	
200	-0.056281	-0.016297	0.0042245	0.3375704	0.4043257
100	0.0172844	0.1268761	0.1129566	0.5239212	1.0653944
50	0.0476834	0.2324607	0.0726254	0.6984053	1.0966921
25	0.0848134	0.2354276	0.2155869	0.8006567	0.9452926
12.5	0.0438674	0.1770681	0.2379932	0.8752345	0.9587786
6.25	0.1300515	0.1609773	0.1849489	0.9268293	1.0750636
3.1	0.0678893	0.2540663	0.1668777	0.772045	1.0178117
1.04	0.2670528	0.5033159	0.3779834	0.8433396	1.0178117
0.35	0.5423423	0.7232112	0.6751096	0.8414634	1.0071247
0.035	0.997426	0.7919721	0.9381393	0.8592871	0.9961832
0.0035	0.956242	0.7724258	0.9883098	0.7378049	0.9251908
0	1	1	1	1	1

Normalised V0

)A	BRCC36 R167A			BRCC36 L169A		
0.0583791	0.0315687	-0.011106	0.0082031	0.104628	0.175309	0.131516
0.1251374	0.0489616	0.0599416	0.0644423	0.2210363	0.1824123	0.1666667
0.2071429	0.0877154	0.0256406	0.1249285	0.4061116	0.3534593	0.2295552
0.314881	0.0948299	0.0798411	0.185367	0.8376882	0.5364398	0.5420851
0.5535714	0.1495802	0.1377717	0.2655863	0.9362267	0.6885921	0.7433046
0.7788462	0.2083076	0.1432695	0.2477598	1.0482728	0.8249751	0.5297704
0.8992674	0.2550155	0.2708401	0.379266	0.9840567	0.8675948	0.8238881
1.0393773	0.4684048	0.5325981	0.6806482	0.9612489	0.8705782	1.2003826
1.081044	0.7839152	0.6796951	0.8484271	0.9313552	0.9390538	1.2482066
1.0068681	0.9845338	0.8352254	0.9985701	0.9466342	0.9295354	1.036944
1.0554029	0.9301812	0.757217	1.0414681	0.9995571	0.8978548	1.1189622
1	1	1	1	1	1	1

'W	Abraxas2 T135K			Abraxas2 T140A		
0.0783114	-0.013741	0.0391456	0.0408525	-0.150175	0.1434007	-0.026812
0.1360718	-0.015592	0.0283101	0.106623	-0.028281	0.3536266	0.1450647
0.1725228	0.0363854	0.0911392	0.1380328	-0.044703	0.5008323	0.2190129
0.2045501	0.0578841	0.1752532	0.1706557	0.026178	0.2897741	0.3135113
0.2425935	0.0768766	0.1805063	0.2120984	0.0308028	0.5136742	0.4015372
0.312184	0.0395718	0.2786076	0.2820656	0.3289703	0.7109394	0.4592233
0.6385238	0.2093199	0.3898101	0.3174098	0.5342059	0.505113	0.6279126
0.9547523	0.4473552	0.6658228	0.6311475	0.5766143	0.9907253	0.9142395
0.9951972	0.734131	0.821519	0.7993443	0.9947644	0.943044	1.1407767
1.0490394	0.8100756	0.8670886	1.077377	0.7625654	1.0117717	1.0291262
1.0381699	0.7641058	0.9018987	1.0006557	0.890925	0.924019	1.0461165
1	1	1	1	1	1	1

'A	BRCC45 F140A			BRCC45 R137A+F140A		
0.0591934	0.0015831	0.0353622	0.0392304	0.076737	0.2358605	0.0245061
0.3223804	0.0505947	0.0814889	0.0937242	0.1783839	0.3294898	0.1016291
0.562673	0.0895532	0.0676056	0.1692665	0.2374678	1.0677429	0.1595841
0.9300119	0.1420122	0.161167	0.2166322	0.3697375	0.6281349	0.2327903
0.9331752	0.268531	0.2798793	0.2660124	0.5229027	0.7549726	0.3889081
0.491894	0.4390871	0.3435614	0.244344	0.6670098	0.8227155	0.2939688
1.0177936	0.512054	0.5186117	0.5529442	0.6505404	0.8757567	0.6381282
1.1399763	0.7470267	0.7252515	0.8021694	0.7498713	1.0054771	0.9362218
1.166469	0.8987464	0.7579477	0.8664773	0.7493567	0.9331219	0.9667244
1.1763543	0.9251045	0.8270624	0.9522211	0.7987648	0.991352	0.9764298
1.1020166	0.9601414	0.783501	0.8331612	0.4872877	1.0328625	1.0117851
1	1	1	1	1	1	1

BRCC36 L169R			BRCC36 L169W			BRCC
0.0974723	0.8436019	0.0332306	0.0594858	0.7259878	0.0442276	0.2033871
0.361261	0.9330806	0.197586	0.4037526	0.9144377	0.2558915	0.5690323
0.8216416	0.9683412	0.5891115	0.8482743	0.9533435	0.8183766	0.7958065
0.8804317	0.9281517	0.923472	0.9379199	0.96231	0.9157343	0.856129
0.8881	1.0130806	1.1111967	0.898309	0.912462	1.037134	0.8351613
0.8906561	0.974218	1.0937339	0.9031735	0.956535	0.9902404	0.9625806
0.8952002	0.9584834	0.8875193	0.9346769	0.8963526	0.9414425	0.8645161
0.9602386	0.9636019	1.007961	0.8899699	0.8901216	0.7967151	0.7632258
0.9369497	0.9791469	1.0639445	0.9402363	0.9516717	0.9419186	0.9858065
0.9681909	0.997346	1.0608629	0.9193885	0.9077508	0.9352535	0.8680645
0.9875036	1.0200948	1.0005136	0.9404679	0.8589666	0.9983337	0.7548387
1	1	1	1	1	1	1

BRCC45 C245A			BRCC45 D248R		
-0.012052	0.163424	0.0959223	0.1175042	0.33106	0.1560671
0.0569095	0.2386151	0.2675243	0.2878921	0.5541883	0.682494
0.1628559	0.3901869	0.3091262	0.482968	0.8228317	0.6446043
0.4664992	0.3911215	0.4218447	0.6505902	0.9229059	0.940048
0.4334171	0.6091759	0.5674757	0.6546374	0.8013343	1.0158273
0.6637353	0.7056075	0.668932	0.8647555	0.95404	1.1352518
0.4832496	0.7931181	0.7033981	0.8775717	0.8591549	1.146283
0.8844221	0.8228547	0.826699	0.9281619	0.8717569	1.2196643
0.9468174	0.8300765	0.9563107	0.9166948	0.9844329	1.0076739
0.7462312	0.9090909	0.9446602	0.9743676	0.9117865	1.0551559
0.9765494	0.9354291	0.9135922	0.917032	0.9555226	1.0666667
1	1	1	1	1	1

**:36 R167A+L169A**
0.8120314
0.883871
1.0252833
1.0528335
1.0725371
1.1858762
1.0786399
1.0798605
1.0524847
1.0714908
1.1635571
1
