# Supplementary material for: Molecular glues that inhibit deubiquitylase activity and inflammatory signaling
Source: Nat Struct Mol Biol. Author manuscript; Available in PMC 2025 Jul 7. (PMC7617869; doi:10.1038/s41594-025-01517-5)
Supplement: Supplementary file 10 [file EMS206745-supplement-supp_5.pdf]

No IFN	IFN	IFN + AP-5-145	IFN + JMS-175-2	IFN + FX-171-C
100	83.24729675	65.89835645	48.45665243	56.26509181
100	88.93228899	82.46681605	45.75649802	56.96400304
100	76.31421036	66.48118171	33.06493253	56.05262423

IFN + FX-171-A	IFN + Tofacitinib*
59.94802399	59.2689369
80.39868703	88.38887404
71.13133033	79.9065714
