# Supplementary material for: Molecular glues that inhibit deubiquitylase activity and inflammatory signaling
Source: Nat Struct Mol Biol. Author manuscript; Available in PMC 2025 Jul 7. (PMC7617869; doi:10.1038/s41594-025-01517-5)
Supplement: Supplementary file 11 [file EMS206745-supplement-supp_6.pdf]

Type I IFN signalling array raw data - analysis of 34 ISGs from unstimulated PBMCs from SSc pati

gene	AP-5-145_P1	AP-5-145_P6	AP-5-145_P8	JMS-175-2_P1	JMS-175-2_P6	JMS-175-2_P8
ADAR	3.58461789	4.69931195	3.55935004	3.10069671	4.47344584	4.36301021
BST2	3.18448489	1.59531495	1.65414304	4.63266271	2.60919884	2.48283621
CASP1	1.62813789	2.36119495	1.18273004	2.74661971	2.11351084	1.95828821
CD86	4.78534489	3.55207495	3.54958004	6.49274471	4.68479184	4.19599121
CXCL10	2.86429989	4.52919495	3.68795604	5.78244671	5.51476884	4.58536521
DDX58	2.45950089	1.78703495	1.17462204	3.51922071	2.66473584	2.26886521
GBP1	0.14743589	1.75372895	-0.462083	1.14256571	2.38259184	0.77993621
IFI16	1.82995789	1.29156495	1.16580404	0.87214971	1.74352284	1.34449321
IFI6	2.46467389	0.80326595	1.08720304	4.37943671	1.89459084	1.40311021
IFIH1	3.17396589	3.04197695	2.58205904	3.58711271	3.86474984	3.37566721
IFIT1	4.23989489	1.39717295	1.66989604	4.64488071	3.60702884	3.51101821
IFIT2	3.35329289	0.07596795	0.85096604	2.22884771	3.19012684	2.83774921
IFIT3	2.46344789	1.24797595	1.26274704	2.49596671	2.53006184	2.34274221
IFITM1	-0.2471271	0.31684495	-0.828512	1.50895571	1.10538684	0.55869221
IFITM2	3.99901389	5.10823995	4.19061704	5.15946471	5.09526884	4.82396821
IFITM3	3.00636089	-0.1661911	-0.202598	4.50767571	1.71863584	2.21105621
IRF1	1.05965989	2.06702195	0.52422404	1.15930571	2.10465984	0.76887321
IRF7	5.40716989	3.93263495	9.93296104	4.91169571	4.79813784	3.67601321
IRF9	2.06622489	1.10607695	0.83657704	2.00123871	1.77817184	1.07405821
ISG15	2.59590689	0.36668995	0.10793904	3.54031671	2.47481784	2.53976621
ISG20	2.43159289	3.17404495	1.32154004	3.11483471	4.07035284	2.36154921
MX1	4.42387789	2.39894295	2.67902404	4.68139171	3.29673884	2.96026621
MX2	4.17302089	2.07445295	2.35476904	3.94660471	3.18141584	2.65183621
MYD88	3.56256289	1.99349395	2.17998204	4.18781171	3.51414684	3.44597921
NMI	1.72974789	1.72385195	1.09858604	1.86096471	2.10652384	1.94494821
OAS1	3.53675089	2.39252995	2.84801404	4.75309471	3.12183384	2.56164321
OAS2	3.25593389	2.62215195	2.25726004	4.56821671	3.26888684	1.99042321
PML	3.76948189	3.58074495	2.63229204	3.58277371	3.77355584	3.13702621
SOCS1	4.27619189	2.28801095	1.78938204	3.78422371	3.31398384	2.26111721
STAT1	-1.1712571	1.68928495	-1.283489	0.47348671	0.15696184	-0.9535318
STAT2	3.54306989	3.23608795	2.12891404	3.74160271	3.17802884	2.42035821
STAT3	1.89754689	1.51959995	1.32991704	1.39985671	1.71836284	1.63708821
TAP1	1.43634389	1.83615895	0.95571404	1.44703771	2.05340184	1.01428921
TNFSF10	2.31792489	2.43409495	2.17731004	3.85989671	3.83918184	2.70612521

ents

FX-171-C_P1	FX-171-C_P6	FX-171-C_P8	AP-5-145_P4	JMS-175-2_P4	AP-5-145_P9	JMS-175-2_P9
3.33068577	4.91346025	3.91817901	4.48511211	3.81157178	4.33423915	3.74277031
3.46838477	1.59071425	2.87296801	2.45803211	2.63376878	3.30210815	3.88860631
2.04551977	2.57415025	1.86687401	0.98154511	1.21275778	2.95035515	2.81227431
6.16868477	4.49895125	4.77952501	2.78303611	3.22765078	4.58520415	5.90588831
4.48312877	4.87069725	3.92272301	6.70758311	8.70974878	5.45318615	7.70629231
1.97373677	1.82917225	2.96761201	2.54337911	2.74299178	3.02553515	2.93992931
0.72059777	2.17874325	0.56726901	2.14483211	2.15802178	2.62187115	2.72775731
1.06403077	1.53258325	1.82068201	1.64380711	2.04907678	2.04915915	1.79364631
3.39419677	1.43525725	1.65674901	2.22676911	2.42500778	2.93611515	3.94747131
3.84879077	3.94867725	3.41735901	3.64233511	4.06680578	3.69321915	4.07210831
4.25233577	2.84362025	3.47530601	3.64253911	6.81265178	3.98928015	5.48136031
3.48558877	3.02836025	3.26868701	3.20168611	3.87681678	2.78009615	4.27635331
3.50313077	5.87707225	2.48592301	3.28073611	4.82099478	3.06390915	4.96752831
1.14394677	1.62930225	0.71254901	0.12428511	1.01825478	0.27155815	0.95929631
4.84533977	5.36691725	4.76243101	3.47138511	4.11645578	4.22008515	4.70012031
3.90207777	1.13071125	2.53569901	2.31983111	2.91555678	3.24385415	4.16474231
0.66485377	1.92723225	1.20726501	1.22444011	1.52600778	2.23167015	2.16541831
5.37200277	4.28309825	3.54699001	5.20628211	5.38456478	4.57592615	5.09617631
1.69021377	1.21402925	1.52141501	2.06456611	1.63011678	2.24089415	2.36346931
2.50806777	1.71127325	2.31134101	6.31154711	7.82827278	3.35450315	5.15740631
2.59486677	4.03628225	2.58499901	2.47946611	2.45405878	2.54728315	3.35977131
3.86572777	4.59083025	2.85357801	4.22633811	4.39015478	3.57986015	3.31669131
3.31786477	2.65281225	2.69101801	3.65825211	4.11923478	2.95183115	3.61590631
3.07445477	1.90815425	3.45387701	2.29544211	2.46999878	2.99177115	3.65990931
1.34621977	2.49728025	2.32019301	1.40646011	1.56201478	2.20547615	2.47276831
3.49763977	2.18671625	2.86294101	3.85190111	3.99460178	3.95765015	5.08033531
2.50385177	2.94131525	2.82137601	3.66498611	4.01168378	3.69563615	3.31459931
3.33489877	3.80691925	3.40742001	4.45087311	3.46353878	4.45711815	4.08170031
3.41590577	2.79255225	2.57257701	3.14450711	3.52737578	2.94300215	3.23127631
-1.1658792	0.22008725	-0.838889	0.55066211	0.65796178	0.70399015	1.17138631
2.88156677	3.30650525	2.68340801	4.37268911	3.70216478	3.59684615	3.43783131
1.35333477	3.43545725	1.67275701	1.63611111	1.85005278	1.63261315	1.46669331
0.81815677	1.84415425	1.13653101	1.64863111	1.73913178	2.57570215	1.98837631
3.10280177	5.01537325	2.88162301	2.98938411	2.97058378	3.02799615	3.81011631

FX-171-C_P9	AP-5-145_P2	JMS-175-2_P2	FX-171-C_P2	FX-171-C_P3	AP-5-145_P3	JMS-175-2_P3
3.85821497	4.35271932	4.1126124	4.27364038	4.00083744	5.31334562	4.43467244
3.57303597	3.67192032	3.0720774	2.85910138	2.17695044	2.30861362	2.87045044
2.54831697	3.66436532	2.3984704	2.00221838	1.94128744	1.94812062	2.03901444
6.16547497	3.18969632	3.9008554	2.64977838	5.64975344	4.31400362	5.36128944
5.47085397	2.73368032	4.2051124	2.91735538	2.58181744	4.39563662	4.63516044
3.35000397	3.20935632	3.0215934	3.30913038	2.13540444	2.76285462	3.29826644
2.68803597	1.45943232	1.9795704	1.36730838	1.42223744	0.85446462	1.48026244
1.76503397	2.36208532	2.2144244	1.77476838	1.07693444	1.33058462	1.76947144
3.89341097	2.72984432	3.0757944	2.68061338	0.95812744	1.77206862	2.46782244
4.42334697	3.98577432	3.5554014	3.59972038	2.51053544	3.42878862	2.79278944
4.28967997	4.29271232	4.3874344	4.38486138	1.62591544	2.48843862	3.43115344
4.66417397	3.71852632	4.3302764	3.90754938	1.67815744	2.32684062	2.58782944
3.79917897	3.57488932	3.5709364	3.67540838	1.20941744	3.04255862	2.47828644
0.94883497	1.17798332	1.4215564	1.17809538	0.39541244	0.81827062	1.40265044
4.64755397	5.17980332	5.1920114	5.17255438	4.78986144	5.26169062	4.82393844
3.96763797	3.78552932	3.8372934	3.91553538	1.58964544	1.43368262	2.08017544
2.54694497	2.14360732	1.9225314	1.82071538	1.07305144	1.14996562	0.67145544
6.80236997	6.17761532	5.8854414	6.58600838	3.98566944	4.45485362	3.92465144
2.19006397	2.26919532	2.2294794	2.33504538	0.97500744	1.29411162	1.68844344
3.98578797	3.69303632	3.9137764	3.92233738	0.69199344	0.96213462	1.96995144
2.62027497	3.37468132	3.2663194	3.22426338	2.38462244	2.84268462	3.42005144
3.10711997	4.63393132	4.4781634	4.40516838	1.26113844	2.27994662	2.34318644
4.12450597	4.35697732	4.1616994	4.08179838	1.60711644	2.35798762	3.30268644
3.73815097	3.31689632	3.4388294	3.07474938	2.51047344	2.32138562	3.35938944
2.14330797	1.95766632	2.0432094	1.85037138	1.49120844	1.87810562	2.15688144
4.42362897	4.26027932	3.9747414	3.83587338	1.73726644	2.48875462	3.10251244
4.60764697	4.93047432	4.4689234	3.61538838	1.64228644	3.21637462	3.57544844
4.55248397	4.68165532	4.5783594	5.07469038	2.98803844	3.16862762	3.20587644
3.57792197	3.91463632	4.3511524	4.26593038	3.58168744	3.67169662	3.05514844
0.97695897	-0.4525667	-0.3774296	-0.7577086	-0.7275296	-0.2783044	-0.0354656
3.96763997	3.34396332	3.1482684	3.15866838	2.80380044	3.62694262	3.83377444
6.67566097	5.44597532	4.5243574	4.39006638	1.89688744	2.41156262	1.72762444
2.35910397	2.25459832	2.5942454	1.92569638	1.56300944	2.07466362	1.81919844
3.90221597	3.37409332	3.8304174	3.28499438	2.14084744	2.09211462	3.39341144

AP-5-145_P7	JMS-175-2_P7	FX-171-C_P7	AP-5-145_P5	JMS-175-2_P5	FX-171-C_P5	FX-171-C_P4
4.41488995	3.83200141	4.68033699	5.11068711	4.96505736	4.77706122	4.20806636
4.59012595	3.49444141	3.61394699	3.68485711	3.79494036	3.67171122	2.32662736
2.03320995	1.15647641	1.13368599	2.20602711	2.51987736	2.88795622	1.29637336
4.03429595	5.14864241	3.48501599	4.01315711	4.52053236	4.72902722	3.41914936
5.67985895	4.65861941	4.74218299	6.07818511	4.11079736	4.87746522	6.90451736
3.54297595	2.83918441	2.91253799	4.17263511	3.97968636	3.78476722	3.50162536
2.93918595	2.26066141	2.89499799	2.17660111	1.75920036	1.58310522	2.08555636
2.49156195	1.84640741	1.99192399	2.95058711	2.52036436	2.37722522	1.62229036
3.20665695	2.75502441	2.49029299	3.66379711	3.75323036	3.89724322	2.02613436
4.01090195	3.20489541	3.46780899	5.19250711	5.37665136	5.09699222	3.97967136
4.57454295	4.75784441	3.83025299	7.13808411	6.99256636	6.55329922	6.51835036
4.72796995	4.40332441	5.24817199	5.59162711	4.74526536	5.23485222	3.76383736
5.13463595	4.36500241	4.92766699	5.47554311	4.92282736	5.41449322	5.43569336
1.19617195	1.36921941	0.89939999	1.70467511	1.66185836	1.97139722	0.87677536
4.67335095	4.73677941	4.97366499	5.08983011	4.67785936	4.48075822	3.98609736
3.42017095	3.73263841	3.56762699	4.49002711	4.35173136	4.15118822	2.87717836
2.58531895	1.65422141	2.33502899	1.97299111	1.16082136	1.15753722	0.99081836
5.88977995	5.47784141	5.79761899	5.93541811	5.12638036	5.44296222	4.60082036
2.97286395	2.54465041	2.22906799	2.80945111	2.71651536	2.46052022	1.89630336
4.12440295	4.25177441	4.26192699	6.01496711	5.48828036	5.47735422	7.49532036
3.39273795	2.94994441	3.29992799	3.46356311	3.39495936	3.62282922	2.61771336
4.39507295	4.00165441	5.21032499	5.55837611	5.29865036	5.06152122	3.69247436
3.74486595	3.80743341	3.74052199	4.85057611	5.10397236	5.02129522	4.03215236
3.13559495	3.39676741	2.92499699	2.86932111	3.68126036	2.92709322	2.37593636
2.63287695	2.03714641	1.97347399	2.76247311	2.36708036	3.71565522	1.26429136
5.00967795	3.83206241	5.32741299	4.92522911	4.43911836	4.79327422	3.41253236
4.72453995	4.21452441	3.79761699	4.97111311	3.93791136	4.11858022	3.65525836
5.04809195	4.34434241	4.36110499	5.21048711	4.48018436	4.17723522	3.43604636
4.38087595	4.32105941	3.84660099	4.14897911	4.16104736	3.63229522	2.69956436
0.55334095	0.50324741	0.78982699	0.24229211	0.15773336	-0.2010878	0.56368436
4.14778295	3.16559441	5.57296299	3.98632011	3.67587436	3.71226722	3.00144036
2.39959895	2.04976141	3.49405699	2.60996711	2.15066736	2.05283922	1.77306036
3.73366095	2.31577041	2.71239799	2.30877811	2.65911236	1.57777022	1.33581336
3.55842695	3.71205441	3.03103999	3.76843611	3.50625336	3.78912122	2.99486536
