# Supplementary material for: Molecular glues that inhibit deubiquitylase activity and inflammatory signaling
Source: Nat Struct Mol Biol. Author manuscript; Available in PMC 2025 Jul 7. (PMC7617869; doi:10.1038/s41594-025-01517-5)
Supplement: Supplementary file 12 [file EMS206745-supplement-supp_7.pdf]

Compound name
JMS-175-2

DUB
Activity (% control)

Screening concentration
5 µM

Activity (% control) = ((sample - mean no enzyme)/mean plus enzyme - mean no enzyme)*100

USP1/UAF1		USP2		USP4		US
81	82	86	84	101	101	103

P5		USP6		USP7		USP8	
	105	92	95	116	121	88	84

USP9X		USP10		USP11		USP12/UA
103	98	95	102	102	100	96

F1/WDR20	USP14		USP15		USP16	
96	85	95	111	115	79	78

USP17		USP19		USP20		US[illegible]
89	83	100	99	93	101	93

P21		USP24		USP25		USP27X	
	93	98	99	93	94	102	97

USP28		USP30		USP35		USP[illegible]
107	96	89	89	96	94	108

P36		USP38		USP45		USP46/UAF1/WDR20	
	117	94	92	112	107	95	99

USP48		CYLD		UCHL1		UC[illegible]
99	103	104	112	104	112	95

HL3		UCHL5		BAP1		OTU1	
	96	90	98	92	96	84	84

OTUB2		OTUD1		OTUD3		OTUD5[illegible]
97	96	100	102	95	93	96

(p177S)		OTUD6A		OTUD6B		Cezanne	
	99	100	103	102	101	88	90

VCPIP		AMSH-LP		AMSH-LP(+Zn^{2+})		Ata[illegible]
93	93	98	90	100	99	87

xin3		Ataxin3L		JOSD1		JOSD2	
	85	98	95	67	67	101	102
