# Supplementary material for: Molecular glues that inhibit deubiquitylase activity and inflammatory signaling
Source: Nat Struct Mol Biol. Author manuscript; Available in PMC 2025 Jul 7. (PMC7617869; doi:10.1038/s41594-025-01517-5)
Supplement: Supplementary file 13 [file EMS206745-supplement-supp_8.pdf]

[di-Ub-TAMRA (nM)]	[JMS-175-2 (µM)]		50 µM	
31.2		31.39	49.47	41.04
62.5		43.48	66.23	64.63
125		67.12	88.75	84.05
250		119.1	114.6	160
500		187.5	427.2	246.3
1000		371.3	640.2	667.9

[di-Ub-TAMRA (nM)]	[FX-171-C (µM)]		50 µM	
31.2		14.21	35.58	38.22
62.5		28.22	40.49	46.74
125		36.3	46.67	63.46
250		67.25	84.89	62.1
500		109.3	143.3	196.9
1000		213.3	360.7	415.9

Initial

	**12.5 µM**			**3.12 µM**		
62.2	65.87	55.25	77.42	88.2	80.48	114
80.26	123.7	100.7	117.4	160.5	160.2	172.4
183.3	175.6	208.5	277.7	256.8	310.9	402.8
283.5	316	354.2	456.6	483.6	573.6	740.9
579	1146	683.3	881.4	1578	1143	1362
1009	1193	1032	1418	1905	1731	2371

	**12.5 µM**			**3.12 µM**		
38.22	44.65	65.69	46.27	64.26	58.53	76.24
34.93	52.19	58.9	76.58	69.67	70.11	126.7
92.02	100.3	111.4	175.1	170.4	173.8	304.6
178.9	174	179.5	322.6	305.6	309.4	548.6
332.6	297.9	255.2	642.2	551.1	624.9	1085
493.9	641.6	679	979	990.8	1134	1629

l velocity (FU/min)

781 nM			195 nM			48 nM
123.2	122.2	137.4	132.5	144.8	124.7	137.4
226.1	228.4	205.5	232	265.7	222.1	242.3
468.4	474	474.4	538.4	481.2	476.9	540.8
834.7	869	866.4	900.9	960.9	886	905.7
2989	1732	1677	3345	1765	1705	3322
2828	2787	3093	3236	3210	3019	3362

781 nM			195 nM			48 nM
88.16	98.75	104.4	118.9	118.7	121.5	126.3
95.78	99.98	166.2	105.9	106.4	182.7	137.5
276.4	303.4	414.6	331.3	418.4	467.2	386.6
487.2	518.3	732.8	688	641.2	794.1	722.2
985.1	1129	1442	1370	1434	1729	1553
1647	1858	2231	2296	2566	2734	2460

		0 nM	
146.6	137.8	176.5	143.2
243.8	235.9	275.7	311.3
503.9	493.6	586.2	532.5
906.9	1069	984.5	925.2
1624	1658	3479	1856
3486	3184	4018	3437

		0 nM	
146.6	131.2	130.2	136.8
123.2	211.1	145.7	132.4
434.5	518	429.9	490.1
770.5	902.4	758.6	800
1273	1822	1734	1625
2836	2993	2747	2929
